# Genetic Diversity and Molecular Evolution of Chinese Waxy Maize Germplasm

**DOI:** 10.1371/journal.pone.0066606

**Published:** 2013-06-20

**Authors:** Hongjian Zheng, Hui Wang, Hua Yang, Jinhong Wu, Biao Shi, Run Cai, Yunbi Xu, Aizhong Wu, Lijun Luo

**Affiliations:** 1 Shanghai Academy of Agricultural Sciences, Shanghai, China; 2 Shanghai Agrobiological Gene Center, Shanghai, China; 3 School of Agriculture and Biology, Shanghai Jiaotong University, Shanghai, China; 4 Institute of Crop Science, Chinese Academy of Agricultural Sciences, Beijing, China; 5 International Maize and Wheat Improvement Center (CIMMYT), Texcoco, Mexico; CNR, Italy

## Abstract

Waxy maize (*Zea mays* L. var. *certaina* Kulesh), with many excellent characters in terms of starch composition and economic value, has grown in China for a long history and its production has increased dramatically in recent decades. However, the evolution and origin of waxy maize still remains unclear. We studied the genetic diversity of Chinese waxy maize including typical landraces and inbred lines by SSR analysis and the results showed a wide genetic diversity in the Chinese waxy maize germplasm. We analyzed the origin and evolution of waxy maize by sequencing 108 samples, and downloading 52 sequences from GenBank for the waxy locus in a number of accessions from genus *Zea*. A sharp reduction of nucleotide diversity and significant neutrality tests (Tajima’s *D* and Fu and Li’s F*) were observed at the waxy locus in Chinese waxy maize but not in nonglutinous maize. Phylogenetic analysis indicated that Chinese waxy maize originated from the cultivated flint maize and most of the modern waxy maize inbred lines showed a distinct independent origin and evolution process compared with the germplasm from Southwest China. The results indicated that an agronomic trait can be quickly improved to meet production demand by selection.

## Introduction

Waxy maize (*Zea mays* L. var. *certain* Kulesh) is a special cultivated type of maize, and was first discovered in China in 1908 and then in other Asian countries [1]–[4]. Waxy maize, with nearly 100% amylopectin in endosperm, is mainly used as food in Asia, and is also an important raw material for food industries, textiles, paper-making and feedstuff worldwide because of its excellent characters in terms of starch composition and economic value [5]. Despite waxy maize was first discovered in China, the origin and evolution of waxy maize is still an enigma [6]–[11].

There are abundant waxy maize landraces in China, most of which distribute in Southwestern China, particularly in Yunnan, Guizhou and Guangxi [5], [7]. Yunnan is an original area of many important plant species with high genetic diversity. Several studies have suggested that Chinese waxy maize originated from Yunnan and Guangxi according to morphology, karotype, isozymes and DNA markers [3], [10], [12]–[14]. There is a wide genetic diversity of waxy maize in agronomic traits such as plant height, maturity, economic characters, resistance to insects and diseases and yield components in those regions [5]. With simple sequence repeats (SSR) markers, comparative analysis of genetic diversity in landraces of waxy maize from Yunnan and Guizhou concluded that both Yunnan and Guizhou would be the center of diversity and origin for waxy maize [10].

According to plant morphology, geographical distribution and biological characters, combined with historical data and folklore, waxy maize was considered to be derived from one single gene mutant from flint corn [5]. However, in 1970, a landrace (termed Four-row Wax due to only four rows of seed set in the cob) was collected from Menghai County, Yunnan Province, which has been planted by the local Dai minority since 1890 and is a primitive cultivar with many characters similar to that of wild species [3], [15]. The C-band pattern and karyotype of waxy maize were similar to popcorn, and the latter was considered as the oldest type of maize [16]. In the isozyme patterns of malic dehydrogenase, Chinese waxy maize has six bands as the same as that of Coix, suggesting that the origin of waxy maize might be related to Coix [6]. All these studies have made the origin and evolution of waxy maize more mysterious.

**Figure 1:**
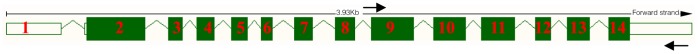
Gene structure of waxy gene in maize ( http://www.maizesequence.org/Zea_mays/GRMZM2G024993_T01 **).** Boxes represent exons. Lines between exons represent introns. The region between the two arrows is the fragment used in this study.

The glutinous phenotype in maize has been shown to be resulted from a dramatic reduction in synthesis of amylose because of mutations or insertions in the waxy gene, which locates on the short arm of chromosome 9 and encodes a granule-bound starch synthase in maize [11], [17]–[18]. The DNA sequence of the wild type waxy locus was determined in 1980s in maize and is composed of 14 exons (Fig. 1) [18]. Recently, waxy sequences have been determined in a number of accessions from *Zea* lineage [4], [19]–[22].

In maize, several genes such as *ae1*, *tb1* and *Y1* have been shown to be under strong selection. In rice, the origin of glutinous rice is associated with reduced genetic variation [23], and a selective sweep of about 250 kb in the waxy genomic region was observed [24]. However, there were several contrary reports on selection of waxy gene in maize [11], [20]–[22]. Further studies are needed to elucidate whether a same strong selection in waxy genomic region has happened under domestication in Chinese waxy maize.

With the development of molecular systematics, comparison of DNA sequence variation between closely related species has provided insight into the amount of divergence between sibling species, and the ancestral population size of sibling species [25]. Tian et al determined the systematical position of waxy maize in genus *Zea* and confirmed the relationship among waxy maize, normal maize and progenitor of maize [22].

In recent decades, waxy maize production has increased dramatically especially in the developed areas in Southeast China. It was difficult for breeders to utilize the waxy maize landraces because of their undesirable agronomic traits. Many adapted waxy maize lines have been developed for hybrid production through different selection methods [26]. The unclear relationship between these elite waxy maize lines and old landraces makes the understanding of origin and evolution of waxy maize more complicated.

In this study, we first used SSR markers to study the genetic diversity of Chinese waxy maize including landraces and inbred lines; we then sampled waxy sequences from 108 maize accessions including 89 waxy and 19 nonglutinous ones and compared with data of waxy sequences downloaded from GenBank to study the systematic position of Chinese waxy maize in genus *Zea*, and investigate the origin and dynamics of population evolution for Chinese waxy maize.

## Materials and Methods

### SSR Analyses

A set of 165 accessions of Chinese waxy maize including typical landraces and inbred lines were chosen at random and analyzed for the genetic diversity with SSR markers. DNA was extracted employing a modified CTAB procedure [27]. We used the set of 20 SSR markers downloaded from MaizeGDB (http://www.maizegdb.org), two from each chromosome (Table 1). PCR conditions and protocols were according to Bassam et al [28]. SSRs were multiplexed for a maximum efficiency. Fragments were separated using acrylamide gels run. The PIC for each marker was determined as described by Smith et al [29]. Allele identity was used for cluster analysis using NTSYS-pc Version 2.1 with UPGMA method to evaluate the genetic diversity [30].

**Table 1:** Chromosome locations of SSR primers and primer sequences.

No.	Primer Name	Bin	Forward chain	Reverse chain
1	bnlg439	1.03	TTGACATCGCCATCTTGGTGACCA	TCTTAATGCGATCGTACGAAGTTGTGGAA
2	phi011	1.09	TGTTGCTCGGTCACCATACC	GCACACACACAGGACGACAGT
3	bnlg381	2.04	TCCCTCTTGAGTGTTTATCACAAA	GTTTCCATGGGCAGGTGTAT
4	umc1551	2.09	CACCGGAACACCTTCTTACAGTTT	CGAAACCTTCTCGTGATGAGC
5	umc2101	3.00	CCCGGCTAGAGCTATAAAGCAAGT	CTAGCTAGTTTGGTGCGTGGTGAT
6	bnlg197	3.06	GCGAGAAGAAAGCGAGCAGA	CGCCAAGAAGAAACACATCACA
7	phi072	4.01	ACCGTGCATGATTAATTTCTCCAGCCTT	GACAGCGCGCAAATGGATTGAACT
8	bnlg2162	4.08	GTCTGCTGCTAGTGGTGGTG	CACCGGCATTCGATATCTTT
9	umc1705	5.03	ATGCGTCTTTCACAAAGCATTACA	AGGTGCAGTTCATAGACTTCCTGG
10	umc1153	5.09	CAGCATCTATAGCTTGCTTGCATT	TGGGTTTTGTTTGTTTGTTTGTTG
11	phi126	6.00	TCCTGCTTATTGCTTTCGTCAT	GAGCTTGCATATTTCTTGTGGACA
12	mmc0241	6.05	TATATCCGTGCATTTACGTTT	CATCGCTTGTCTGTCGA
13	phi112	7.01	TGCCCTGCAGGTTCACATTGAGT	AGGAGTACGCTTGGATGCTCTTC
14	phi328175	7.04	GGGAAGTGCTCCTTGCAG	CGGTAGGTGAACGCGGTA
15	bnlg2181	8.00	CCAATTCACCAATCATGCAA	TTGGGGTGAAGCAATGTGTA
16	phi080	8.08	CACCCGATGCAACTTGCGTAGA	TCGTCACGTTCCACGACATCAC
17	bnlg244	9.02	GATGCTACTACTGGTCTAGTCCAGA	CTCCTCCACTCATCAGCCTTGA
18	umc1277	9.08	TTTGAGAACGGAAGCAAGTACTCC	ACCAACCAACCACTCCCTTTTTAG
19	umc1380	10.00	CTGCTGATGTCTGGAAGAACCCT	AGCATCATGCCAGCAGGTTTT
20	umc1196	10.07	CGTGCTACTACTGCTACAAAGCGA	AGTCGTTCGTGTCTTCCGAAACT

### Amylopectin Content Determination

Amylopectin content was determined according to the National Standards of the People’s Republic of China, GB 7648–87 (China Standard Press 1987).

### DNA Sequences for Waxy Gene

Eighty-nine diverse accessions of Chinese waxy maize, 16 accessions of flint maize and 3 accessions of sweet maize, including landraces and inbred lines, were selected for amplification and determination of the sequences of the waxy genes (Table 2). The landraces were mainly collected from Yunnan, Guizhou and Guangxi, where Chinese waxy maize was believed to originate. The inbred lines were mainly collected from Shandong, Beijing, Shanghai and Zhejiang where waxy maize production has increased quickly in recent decades. Accessions were sequenced at waxy locus. Primers for two overlapping regions for exon 8–12 (P-F1: 5′ GATTTCATCGACGGGTCTGT -3′ and P-R1: 5′- TCTGTCCCTCTCGTCAGGAT -3′) and exon 11–14 (P-F2: 5′- ATCCTGACGAGAGGGACAGA -3′ and P-R2: 5′- CACCGAACAGCAGGGATTAT -3′) were designed based on conserved regions of the B73 genomic sequence (AF488416). All primers were designed using Primer3 [31]. PCR products were purified with glass milk kit (BioDev Company, China) and were sequenced on both strands using an Applied Biosystems 3730 sequencer with the forward and reverse primers. The alignments were done with DNASTAR (DNASTAR Inc., 2001) and checked manually.

**Table 2:** Summary of accessions sampled for*Waxy* gene analysis.

Population/taxon	Sample name		Type	Origin	Sequence or GenBank no.	Seed source or Reference	
Waxy maize from Southwest China	Banqiaohuangnuo		LR	Guizhou, China	CWM050	CAAS	
	Chaoyangbainuo		LR	Guizhou, China	CWM052	CAAS	
	Diannuobaogu		LR	Yunnan, China	CWM056	CAAS	
	Bainuoyumi		LR	Yunnan, China	CWM057	CAAS	
	Huangnuobaogu		LR	Yunnan, China	CWM069	CAAS	
	Bendinuobaogu		LR	Yunnan, China	CWM074	CAAS	
	Nuobaogu		LR	Shanxi, China	CWM080	CAAS	
	Liuchengnuo		LR	Guangxi, China	DQ369863	Tian,M.L. et. al 2006	
	Dalinuoyumi		LR	Guangxi, China	DQ369864	Tian,M.L. et. al 2006	
	Laonuo		LR	Guangxi, China	DQ369865	Tian,M.L. et. al 2006	
	Wunonglaonuo		LR	Guangxi, China	DQ369866	Tian,M.L. et. al 2006	
	Zaibainuo		LR	Yunnan, China	DQ369886	Tian,M.L. et. al 2006	
	Piantounuo		LR	Yunnan, China	DQ369887	Tian,M.L. et. al 2006	
	Huanuoyumi		LR	Yunnan, China	DQ369888	Tian,M.L. et. al 2006	
	Landihuanuo		LR	Yunnan, China	DQ369891	Tian,M.L. et. al 2006	
	Changchongbainuo		LR	Guizhou, China	DQ369895	Tian,M.L. et. al 2006	
	Huangyounuo		LR	Guizhou, China	DQ369897	Tian,M.L. et. al 2006	
	Jinhuangnuo		LR	Guizhou, China	DQ369899	Tian,M.L. et. al 2006	
	Xiaobainuo		LR	Guizhou, China	DQ369901	Tian,M.L. et. al 2006	
Waxy maize from East China	Baigengyumi		LR	Shanghai, China	SWM011	SAGC	
	Huangnuoyumi		LR	Shanghai, China	SWM012	SAGC	
	Gengbaidayumi		LR	Shanghai, China	SWM017	SAGC	
	SWL089		IL	Shandong, China	SWL089	SAAS	
	SWL094		IL	Shandong, China	SWL094	SAAS	
	SWL097		IL	Shandong, China	SWL097	SAAS	
	SWL102		IL	Shandong, China	SWL102	SAAS	
	SWL105		IL	Shandong, China	SWL105	SAAS	
	SWL107		IL	Shandong, China	SWL107	SAAS	
	SWL108		IL	Shandong, China	SWL108	SAAS	
	SWL124		IL	Shanghai,China	SWL124	SAAS	
	SWL125		IL	Shanghai,China	SWL125	SAAS	
	SWL127		IL	Shanghai,China	SWL127	SAAS	
	SWL129		IL	Shanghai,China	SWL129	SAAS	
	SWL130		IL	Shandong,China	SWL130	SAAS	
	SWL131		IL	Shandong,China	SWL131	SAAS	
	SWL132		IL	Guangdong, China	SWL132	SAAS	
	SWL135		IL	Shandong, China	SWL135	SAAS	
	SWL138		IL	Shandong, China	SWL138	SAAS	
	SWL155		IL	Shanghai, China	SWL155	SAAS	
	SWL161		IL	Shandhai, China	SWL161	SAAS	
	SWL162		IL	Shandong, China	SWL162	SAAS	
	SWL165	IL		Beijing, China	SWL165		SAAS
	SWL166	IL		Jiangsu, China	SWL166		SAAS
	SWL169	IL		Guizhou, China	SWL169		SAAS
	SWL170	IL		Guizhou, China	SWL170		SAAS
	SWL171	IL		Guizhou, China	SWL171		SAAS
Waxy maize from East China	SWL172	IL		Shanghai, China	SWL172		SAAS
	SWL174	IL		Shanghai, China	SWL174		SAAS
	SWL175	IL		Shanghai, China	SWL175		SAAS
	SWL176	IL		Shandong, China	SWL176		SAAS
	SWL177	IL		Shandong, China	SWL177		SAAS
	SWL178	IL		Shandong, China	SWL178		SAAS
	SWL179	IL		Shandong, China	SWL179		SAAS
	SWL180	IL		Shandong, China	SWL180		SAAS
	SWL182	IL		Hubei, China	SWL182		SAAS
	SWL183	IL		Shandong, China	SWL183		SAAS
	SWL190	IL		Shanghai, China	SWL190		SAAS
	SWL191	IL		Shanghai, China	SWL191		SAAS
	SWL193	IL		Shanghai, China	SWL193		SAAS
	SWL194	IL		Shanghai, China	SWL194		SAAS
	SWL196	IL		Shanghai, China	SWL196		SAAS
	SWL197	IL		Shandong, China	SWL197		SAAS
	SWL198	IL		Shanghai, China	SWL198		SAAS
	SWL200	IL		Shanghai, China	SWL200		SAAS
	SWL201	IL		Shanghai, China	SWL201		SAAS
	SWL202	IL		Shanghai, China	SWL202		SAAS
	SWL231	IL		Shanghai, China	SWL231		SAAS
	SWL258	IL		Shanghai, China	SWL258		SAAS
	SWL269	IL		Shanghai, China	SWL269		SAAS
	SWL271	IL		Shanghai, China	SWL271		SAAS
	SWL273	IL		Shanghai, China	SWL273		SAAS
	SWL276	IL		Beijing, China	SWL276		SAAS
	SWL277	IL		Beijing, China	SWL277		SAAS
	SWL278	IL		Jiangsu, China	SWL278		SAAS
	SWL283	IL		Shanghai, China	SWL283		SAAS
	SWL284	IL		Shanghai, China	SWL284		SAAS
	SWL285	IL		Shanghai, China	SWL285		SAAS
	SWL286	IL		Shanghai, China	SWL286		SAAS
	SWL287	IL		Shanghai, China	SWL287		SAAS
	SWL290	IL		Shanghai, China	SWL290		SAAS
	SWL294	IL		Shanghai, China	SWL294		SAAS
	SWL310	IL		Shanghai, China	SWL310		SAAS
	SWL312	IL		Shanghai, China	SWL312		SAAS
	SWL324	IL		Shanghai, China	SWL324		SAAS
	SWL326	IL		Shanghai, China	SWL326		SAAS
	SWL328	IL		Shanghai, China	SWL328		SAAS
	SWL329	IL		Shanghai, China	SWL329		SAAS
	SWL333	IL		Shanghai, China	SWL333		SAAS
	SWL335	IL		Shanghai, China	SWL335		SAAS
	SWL337	IL		Shanghai, China	SWL337		SAAS
	SWL339	IL		Shanghai, China	SWL339		SAAS
	SWL343	IL		Shanghai, China	SWL343		SAAS
	SWL345	IL		Shanghai, China	SWL345		SAAS
	SWL348	IL		Shanghai, China	SWL348		SAAS
Waxy maize from East China	SWL359	IL		Jiangsu, China	SWL359		SAAS
	SWL362	IL		Shanghai, China	SWL362		SAAS
	SWL370	IL		Shanghai, China	SWL370		SAAS
	SWL371	IL		Shanghai, China	SWL371		SAAS
	SWL378	IL		Shanghai, China	SWL378		SAAS
	SWL385	IL		Shanghai, China	SWL385		SAAS
	SWL386	IL		Shanghai, China	SWL386		SAAS
Waxy maize from America	915B	IL		Argentina	EU747862		Zhao,Y. et al 2008
	923A	IL		Argentina	EU747863		Zhao,Y. et al 2008
Flint maize from China	Laorenya	LR		Shanghai, China	SMV002		SAGC
	Ziyuya	LR		Shanghai, China	SMV003		SAGC
	Jinmeihuang	LR		Shanghai, China	SMV004		SAGC
	Matuan	LR		Shanghai, China	SMV005		SAGC
	Zaobai	LR		Shanghai, China	SMV006		SAGC
	Xiaojinhuang	LR		Shanghai, China	SMV015		SAGC
	Huangnuo	LR		Guizhou, China	CMV054		CAAS
	Bainuoyumai	LR		Yunnan, China	CMV065		CAAS
	Heinuobaogu	LR		Yunnan, China	CMV075		CAAS
	SML090	IL		Shandong, China	SML090		SAAS
	SML136	IL		Shandong, China	SML136		SAAS
	SML137	IL		Shandong, China	SML137		SAAS
	SML173	IL		Jiangsu, China	SML173		SAAS
	SML280	IL		Shanghai, China	SML280		SAAS
	SML306	IL		Shanghai, China	SML306		SAAS
	SML383	IL		Shangdong, China	SML383		SAAS
Sweet maize from China	SHL391	IL		Zhejiang, China	SHL391		SAAS
	SHL403	IL		Zhejiang, China	SHL403		SAAS
	SHL408	IL		Zhejiang, China	SHL408		SAAS
Flint maze from America	EP1		IL	America	AF544080		Whitt,S.R., et al. 2002
	Mo17		IL	America	AF544090		Whitt,S.R., et al. 2002
	M162W		IL	America	AF544089		Whitt,S.R., et al. 2002
	Oh43		IL	America	AF544094		Whitt,S.R., et al. 2002
	Ki21		IL	America	AF544087		Whitt,S.R., et al. 2002
	P39		IL	America	AF544095		Whitt,S.R., et al. 2002
	IL101		IL	America	AF544085		Whitt,S.R., et al. 2002
	A6		IL	America	AF544069		Whitt,S.R., et al. 2002
	B37		IL	America	AF544072		Whitt,S.R., et al. 2002
	Pa91		IL	America	AF544096		Whitt,S.R., et al. 2002
	Tx601		IL	America	AF544098		Whitt,S.R., et al. 2002
	NC348		IL	America	AF544093		Whitt,S.R., et al. 2002
	CML254		IL	America	AF544076		Whitt,S.R., et al. 2002
	T232		IL	America	AF544097		Whitt,S.R., et al. 2002
	Ky21		IL	America	AF544088		Whitt,S.R., et al. 2002
	B97		IL	America	AF544074		Whitt,S.R., et al. 2002
	CI187		IL	America	AF544075		Whitt,S.R., et al. 2002
	CML333		IL	America	AF544078		Whitt,S.R., et al. 2002
	W153R		IL	America	AF544099		Whitt,S.R., et al. 2002
	I205		IL	America	AF544083		Whitt,S.R., et al. 2002
	B14A		IL	America	AF544071		Whitt,S.R., et al. 2002
	N28Ht		IL	America	AF544091		Whitt,S.R., et al. 2002
	NC260		IL	America	AF544092		Whitt,S.R., et al. 2002
	IDS28		IL	America	AF544084		Whitt,S.R., et al. 2002
	a2132		IL	America	AF544082		Whitt,S.R., et al. 2002
*Zea mays* ssp.	PI566683		LR	America	mAF292525		Gaut,B.S. et al 2000
*Mexicana*	PI566691		LR	America	mAF292530		Gaut,B.S. et al 2000
	PI566685		LR	America	mAF292524		Gaut,B.S. et al 2000
	PI566673		LR	America	mAF292528		Gaut,B.S. et al 2000
	PI566682		LR	America	mAF292526		Gaut,B.S. et al 2000
	-		LR	America	mAF079260		Mason-Gamer, 1998
*Zea mays* ssp.	PI331786		LR	America	pAF292518		Gaut,B.S. et al 2000
*Parviglumis*	M106		LR	America	pAF292523		Gaut,B.S. et al 2000
	PI331785		LR	America	pAF292517		Gaut,B.S. et al 2000
	PI384061		LR	America	pAF292520		Gaut,B.S. et al 2000
	PI331783		LR	America	pAF292516		Gaut,B.S. et al 2000
	PI384062		LR	America	pAF292521		Gaut,B.S. et al 2000
	PI331787		LR	America	pAF292519		Gaut,B.S. et al 2000

LR: Landrace; IL: Inbred line; CAAS: Chinese Academy of Agricultural Sciences; SAGC: Shanghai Agrobiological Gene Center; SAAS: Shanghai Academy of Agricultural Sciences.

Genetic diversity at waxy gene locus from a wide range of maize (*Zea mays* ssp. *mays*) and its wild relatives (*Z. mays* ssp. *mexicana*, hereafter *mexicana*, and *Z. mays* ssp *parviglumis*, hereafter *parviglumis*) had been investigated in previous studies [20], [32]–[35], and their sequences were downloaded from GenBank (http://blast.ncbi.nlm.nih.gov/Blast.cgi) as comparison in this study. The sampled accessions were classified into eight populations according to their endosperm texture, geographical origins or taxa (Table 2).

### Sequence Variation Analysis for Waxy Gene

Sequence analysis was performed, in part, with the program DNASP version 5.10.01 [36], [37]. The average pairwise nucleotide diversity *π* and Watterson’s estimator of *θ*, an estimate of 4*N_e_μ*, where *N_e_* is the effective population size and *μ* is the mutation rate per nucleotide, were calculated for genetic diversity [38], [39]. The Tajima’s D and Li & Fu’s D* and F* were tested for deviation from the neutral equilibrium model of evolution using the tests of Tajima, and Fu and Li [40], [41]. Recombination rates were estimated with the methods developed by Hudson et al and Hey et al [42], [43].

### Phylogenetic Reconstruction

Neighbour-joining (NJ) phylogenies based on the Kimura 2-parameter distance matrix were generated by MEGA version 4.0 to reconstruct the gene tree using waxy gene data [44]. One thousand bootstrap replicates were used to assess confidence in the phylogeny.

## Results

### Genetic Analyses of Chinese Waxy Maize

We chose 165 accessions of Chinese waxy maize including landraces and inbred lines and analyzed for the genetic diversity with SSR markers. A total of 104 alleles were found at the 20 SSR loci, with a range of 2 to 8 alleles per marker. The average alleles per marker across genotypes were 5.2, which were higher than that (3.7) reported by Wu et al using 16 waxy maize landraces and 61 SSR markers [9]. The PIC values for the 20 SSR loci ranged from 0.41 to 0.84, with an average of 0.70. This is consistent with the results of Xue et al., who reported an average PIC value of 0.64 for 184 maize inbred lines including 111 common and 73 waxy inbreds [45]. The average PIC value in combination with the high number of alleles indicates presence of a wide genetic diversity in the Chinese waxy maize germplasm used in this study.

**Figure 2:**
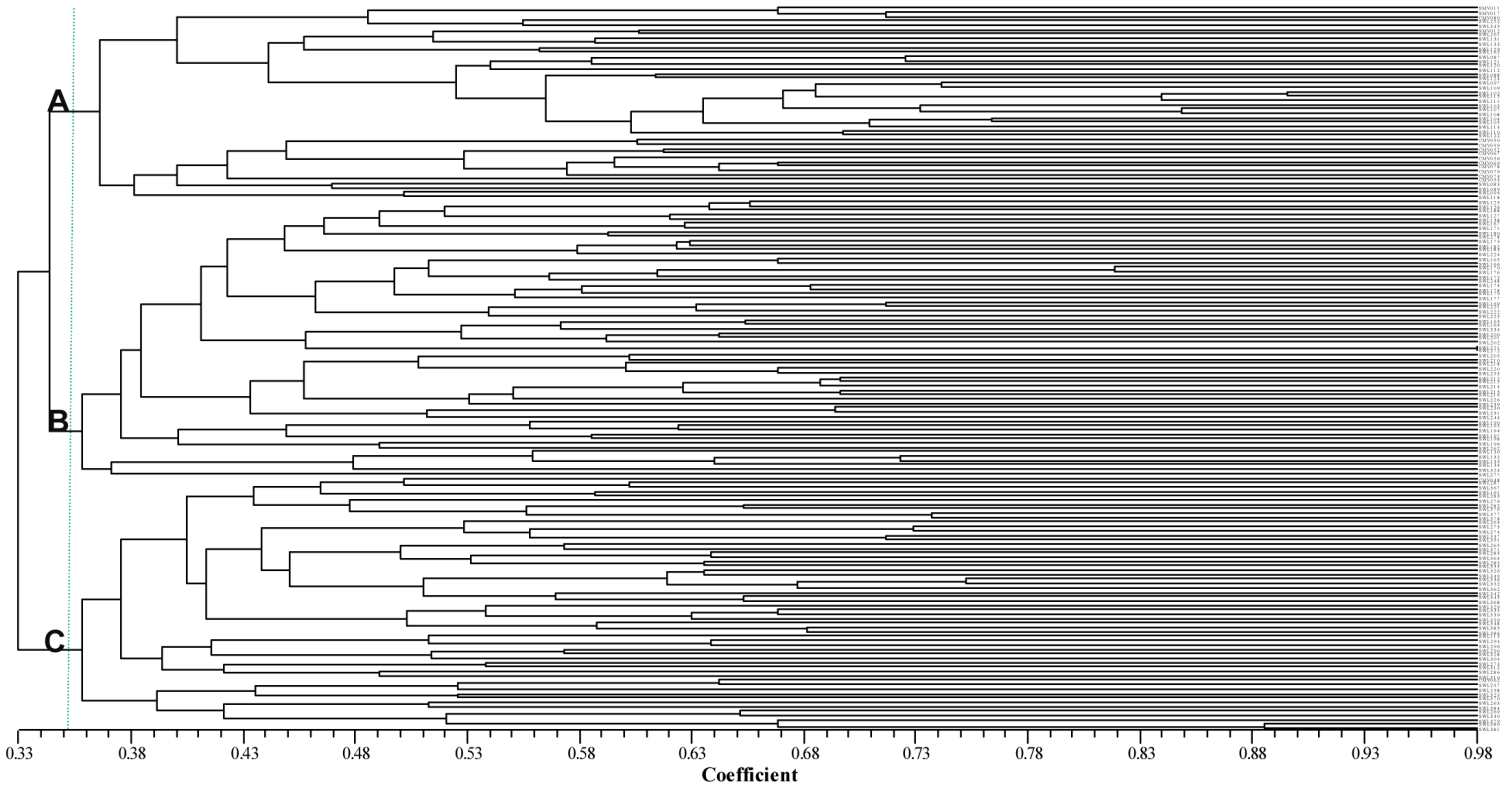
Dendrogram for 165 maize accessions based on a cluster analysis of genetic similarities from SSR data.

Cluster analysis of 165 waxy maize lines was conducted based on genetic similarities from SSR data with UPGMA method. UPGMA analysis grouped the 165 lines into groups A, B and C (Fig. 2), which was generally consistent with their known pedigree information and breeder’s experience. The landraces collected from Southwest China such as Yunnan, Guizhou and Guangxi, and some of inbred lines collected from Northern China formed one group (group A), of which the Southwest Chinese waxy maize land races formed a subgroup. Most of the inbred lines collected from Yangtse River delta including Shanghai and Zhejiang formed one group (group B). Most of the inbred lines collected from North and East China including Shandong, Henan and Beijing formed the group C. The results indicated that most of the modern waxy inbred lines seemed to have little relationship with the Southwest Chinese waxy maize landraces, which were regarded as the origin of Chinese waxy maize [3], [12]–[14]. It would be very helpful to choose the most genetically distant lines for waxy maize genetic study and hybridization breeding based on grouping of waxy maize germplasm.

### DNA Sequence Variation and Test for Deviation from Neutrality

We examined DNA sequence variation in about 1,750 bp region of waxy gene in 89 Chinese waxy, 16 flint and 3 sweet maize accessions, of which 72 nucleotides were variable (Table 3). High amylopectin content (>95%) was observed in the 89 Chinese waxy accessions (Table S1). Waxy sequence data for 52 accessions from maize (*Zea mays* ssp. *mays*) and its wild relatives (*mexicana* and *parviglumis*) were downloaded from GenBank and used as comparison in this study [20], [32]–[35]. Different accessions within each taxon or population showed an apparent difference in genetic variation at waxy locus (Table 3). Average pairwise nucleotide diversity, ***π***, in the nonglutinous maize was more than 3-fold higher than that in the waxy accessions. The waxy maize from East China, mainly being modern waxy maize inbred lines, contained the minimum level of variation among all sampled taxa or populations. The estimate of *π* for waxy maize from East China was 66.0%, 12.7%, 19.9%, 13.1%, 6.1% and 7.0% of that for waxy maize accessions from Southwest China, flint maize from China, sweet maize from China, flint maize from America, *parviglumis* and *mexicana*, respectively. Similarly, *θ* and ***k*** were lower in the waxy maize compared to others samples. The reduction in genetic diversity at waxy locus in waxy maize suggested that Chinese waxy maize experienced a genetic bottleneck during its improvement, especially in modern waxy maize breeding.

**Table 3:** Summary of nucleotide diversity within maize taxon or population.

Taxon or Population	*N* b	Site^b^	*S* b	*H* b	*π* b	*θ* b	*k* b	*D*	D*	F*	*Rm*
Waxy maize from Southwest China^a^	19	1775	12	6	0.00103	0.00260	1.357	−2.20087**	−2.97230*	−3.18559*	0
Waxy maize from East China	82	1791	26	8	0.00068	0.00204	1.170	−2.38092**	−4.10102*	−4.12647*	0
Flint maize from China	16	1793	27	11	0.00535	0.00472	9.225	0.54969	0.86751	0.86751	6
Sweet maize from China	3	1763	9	4	0.00341	0.00341	6.000	-	-	-	-
Flint maize from America^a^	25	1788	23	15	0.00521	0.00634	5.481	−0.68060	−0.92668	−0.99414	5
*parviglumis* a	7	1388	37	7	0.01106	0.01139	14.667	−0.16551	−0.13036	−0.15287	8
*mexicana* a	6	1373	29	6	0.00974	0.01067	12.400	−0.54951	−0.49755	−0.55619	2

a Some sequence data were from Mason et al (1998), Gaut et al (2000); Whitt et al. (2002), Tian et al. (2006), and Zhao et al. (2008).

b *n*, number of samples; *S* : number of polymorphic (segregating) sites; *H*, haploid; *Hd*: haplotype (gene) diversity; *π*, The average pairwise nucleotide diversity (Tajima 1983); *θ*, an estimate of 4N_e_μ, where N_e_ is the effective population size and μ is the mutation rate per nucleotide (Watterson 1975); *K*, average number of nucleotide differences; *D:* Tajima’s D (1989); D*:Fu and Li’s D (1993); F*: Fu and Li’s F (1993); *Rm:* Minimum number of recombination events.

** *P*<0.01; * *P*<0.05.

The Tajima’s *D* and Li & Fu’s D* and F* were tested for deviation from the neutral equilibrium model of evolution based on Tajima, and Fu and Li [40], [41]. Estimates of D, D* and F* were different within each taxon or population (Table 3). All three tests identified a significantly negative selection on the waxy gene in both waxy subpopulations, but not in any nonglutinous subpopulation or taxon, which suggests that strong selection in improvement has acted on the locus in the Chinese waxy population. The negative selection in East Chinese waxy maize accessions, which mainly consist of modern waxy maize inbred lines, was stronger than that in Southwest Chinese waxy maize accessions, which are landraces. The significant neutral deviation at this locus in the waxy population compared to the nonglutinous population is consistent with the sharp reduction in polymorphism.

We estimated the minimum number of recombination events (*Rm*) within the samples of waxy maize sequences (Table 3). The recombination events were 0, 0, 6, 5, 8 and 2 for the waxy maize from Southwest China, waxy maize from East China, Chinese flint maize, American flint, *parviglumis* and *mexicana* accessions. High-frequency recombination appears to somewhat increase the sequence diversity at waxy locus.

### Phylogenetics for Chinese Waxy Maize Based on Sequence Polymorphisms

**Figure 3:**
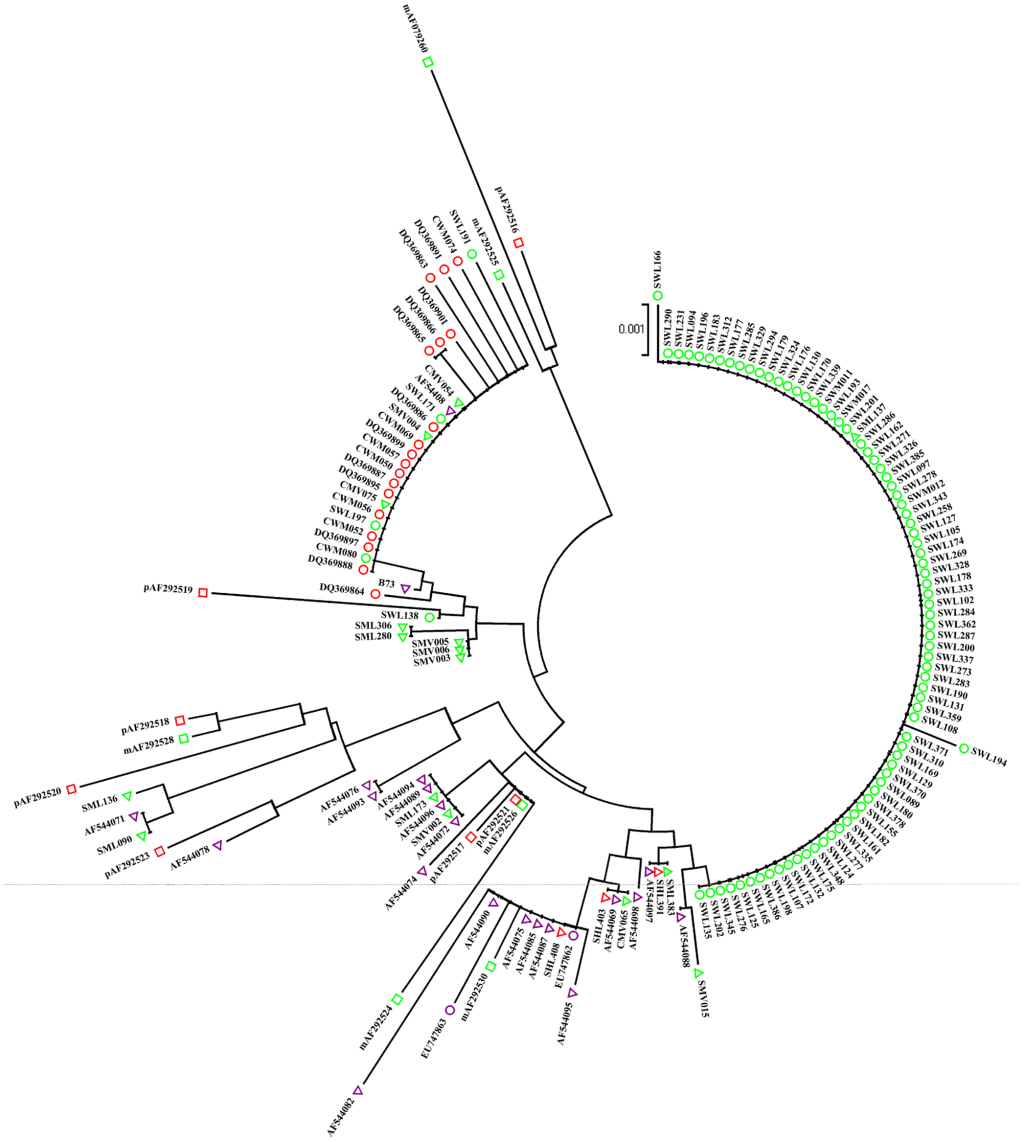
Phylogenetic tree of the Chinese waxy maize accessions based on waxy gene. The waxy maize from Southwest China, East China and America are shown with red, green and purple circles, respectively. The sweet maize, flint maize from China and flint maize from America are shown with red, green and purple triangles, respectively. Wild maize *Zea mays* spp. *parviglumis* and *Zea mays* spp. *mexicana* are shown with red and green squares, respectively.

Based on the waxy sequences, we constructed a phylogenetic tree including Chinese waxy and other nonglutinous maize accessions by neighbor joint method (Fig. 3), which is helpful to elucidate their origins. The tree indicated that Chinese waxy maize were grouped two distinct independent branches, one mainly containing Southwest Chinese waxy maize and the other mainly East Chinese waxy maize. It suggests that the Chinese waxy maize should have two independent origins and evolution processes.

One subset of waxy maize sequences forms one distinct branch except one Chinese flint maize sequence SML137. This group contains most of the waxy maize inbred lines from East China and three landraces Baigengyumi, Huangnuoyumi and Gengbaidayumi. The remaining waxy maize accessions, which contained all other Southwest Chinese waxy landraces and some East Chinese waxy accessions, were mixed with several Chinese flint and America flint accessions. Two American waxy maize accessions (915B and 923A) formed a distinct group mixed with some American flint maize, which was different from Chinese waxy maize. The intermixing is reasonable and essential if waxy maize might be domesticated from flint maize.

Three wild maize accessions (*parviglumis* AF292516, and *mexicana* AF292525 and AF079260) formed a distinct branch which is basal to the flint and waxy maize groups. On the other hand, two different branches were formed with flint maize, which were basal to the two different Chinese waxy maize groups. The results suggest that flint maize maybe be the ancestor of waxy maize, and wild maize relatives *parviglumis* or *mexicana* maybe be the ancestor of flint maize. This suggestion was consistent with the low nucleotide diversity in waxy maize compared to flint maize, *parviglumis* and *mexicana*.

## Discussion

Due to the special dietary habit for glutinous food in China, selection in maize has been made for waxy phenotype with high amylopectin since the maize was introduced into China from the new world about 400 years ago [3]. Although there are abundant waxy maize landraces in south China such as Yunnan, Guizhou and Guangxi, where were regarded as the origin of Chinese waxy maize [3], [12]–[14], they have seldom been used directly or indirectly in modern waxy maize breeding in recent decades [25]. Our study was a try to elucidate the relationship between modern waxy inbred lines and waxy maize landraces.

The waxy mutants give rise to the waxy maize phenotype because the mutants hinder amylase synthesis leading to accumulate nearly 100% amylopectin in starch [25]. In our collection, high amylopectin content (>95%) was observed in the 89 Chinese waxy accessions sequenced by ourselves (Table S1). We tried to include the main typical landraces and inbred lines currently used for commercial production in China to reveal the genetic diversity of Chinese waxy maize in our study.

### Genetic Diversity of Chinese Waxy Maize

Our SSR analysis have shown a wide genetic diversity in Chinese waxy maize accessions including landraces and inbred lines. It is also very interesting to find that the landraces collected from Southwest China formed a subgroup, indicating that most of the modern waxy inbred lines seemed to have little relationship with the Southwest Chinese landraces. A further study on DNA sequence variation at waxy locus for Chinese waxy maize and its wild relatives provided insight to the relationship between modern waxy inbred lines and waxy maize landraces, and information on the origin and dynamics of population evolution for Chinese waxy maize in genus *Zea*.

It is commonly thought that crops are bereft of genetic variation compared to their wild relatives [46]. In our study, the Chinese waxy maize only contained 16.0%, 16.3%, 7.7% and 8.8% of sequence diversity in flint maize accessions from China, flint maize from America, *parviglumis*, and *mexicana*, respectively (Table 3). The decrease of genetic diversity at *waxy* locus was caused by only one possible reason that waxy gene experienced a genetic bottleneck and strong selection during its improvement, especially in modern waxy maize breeding, for waxy maize from Eastern China contained 66.0% of the sequence diversity in waxy maize from Southwest.

Our test for deviation from neutrality revealed a significantly negative selection on the waxy gene in waxy maize, but not in normal maize and their wild relatives. On the other hand, the negative selection in Eastern Chinese waxy maize was stronger than that in Southwest Chinese waxy maize. The neutral test result is consistent with the sharp reduction in polymorphism at this locus in waxy maize compared to the normal maize and their wild relatives. The results suggest that strong selection in improvement has acted on the locus in the Chinese waxy maize.

### Origin and Evolution of Chinese Waxy Maize

Resolving the issues related to the origin and evolution for a domesticated crop is a fascinating and challenging endeavor that requires the integration of botanical, archeological and genetic evidence [47]–[49]. Our data have provided us an explicit relationship among waxy maize, flint maize and the wild relatives for two reasons. First, in the phylogenetic tree, the sequences of Chinese waxy maize formed two groups which are mixed with several Chinese flint maize sequences, and two distinct branches formed with flint maize were basal to the two Chinese waxy maize groups. It showed that waxy maize undergoes the most recent divergence event from flint maize. Second, three wild maize accessions (*parviglumis* AF292516, and *mexicana* AF292525 and AF079260) formed a distinct branch which is basal to the flint and waxy maize groups. The results suggest that wild maize relatives *parviglumis* or *mexicana* maybe be the ancestor of flint maize.

It seems difficult to explain the observation that the intermixture of two subspecies (*parviglumis* and *mexicana*) formed a basal branch in the phylogenetic tree, given that these two subspecies do not grow sympatrically [49], [50]–[52]. There are two possible explanations: (1) there is a long distance dispersal from *parviglumis* to *mexicana* populations; (2) the gene pools of the two subspecies were diverged too late to be fully differentiated. The first explanation seems unlikely but cannot be excluded. The second explanation seems more likely, given that the gene pools of *mexicana*, *parviglumis* and maize differ more in allele frequencies than by allele presence/absence. The result is consistent with the observation of Fukunaga et al [51]. Of course, this viewpoint needs to be supported by more molecular evidences.

Tian et al concluded that Southwest China origin for waxy maize is consistent with local cultural practices [5]. In the past, only in Southwest China does waxy maize attain the importance of a staple crop. Ethnographic studies suggest that waxy maize cultivation is associated with upland agriculture in Southwest China. Indeed, Yunnan, Guizhou and Guangxi in Southwest China are referred to as “the genetic diversity centre” for waxy maize, reflecting the importance of waxy maize to the economy and culture of the area. In recent decades, waxy maize production has increased dramatically especially in the developed areas of Southeast China. Many waxy maize germplasm and new inbred lines have been developed for hybrid production through genetic improvement [26]. Our study suggests that most of the modern waxy maize germplasm showed a distinct, independent origin and evolution compared with the germplasm in Southwest China. The results indicate that an agronomic trait can be quickly improved to meet production demand by artificial selection. It is reasonable to speculate that production demand plays a critical role in crop genetic improvement and can speed the artificial selection and evolution at a target gene locus.

## Supporting Information

Table S1 ***Waxy*** **gene structure of maize accessions sequenced by Shanghai Academy of Agricultural Sciences.**
(DOC)
