# Supplementary material for: Genetic Diversity and Molecular Evolution of Chinese Waxy Maize Germplasm
Source: PLoS One. 2013 Jun 20;8(6):e66606. doi: 10.1371/journal.pone.0066606 (PMC3688585; doi:10.1371/journal.pone.0066606)
Supplement: Table S1 — Waxy gene structure of maize accessions sequenced by Shanghai Academy of Agricultural Sciences. (DOC) [file pone.0066606.s001.doc]

**Table S1. *Waxy* gene structure of maize accessions sequenced by Shanghai Academy of Agricultural Sciences**

| **Accession** | **AC(%)** | **DNA Sequence** |
| --- | --- | --- |
| CWM050 | 98.30 | CCACAACTGTTCGCGTCCTGCTGGTTCATTATCTGACCTTGATTGCATTGCAGCTACGAGAAGCCCGTGGAAGGCCGGAAGATCAACTGGATGAAGGCCGGGATCCTCGAGGCCGACAGGGTCCTCACCGTCAGCCCCTACTACGCCGAGGAGCTCATCTCCGGCATCGCCAGGGGCTGCGAGCTCGACAACATCATGCGCCTCACCGGCATCACCGGCATCGTCAACGGCATGGACGTCAGCGAGTGGGACCCCAGCAGGGACAAGTACATCGCCGTGAAGTACGACGTGTCGACGGTGAGCTGGCTAGCTAGCTGATTCTGCTGCCTGGTCCTCCTGCTCATGCTGGTTCGGTTCTGACGCGGCGAGTGTACGTACGTGCGTGCGACGGTGGTGTGGTGTCCGGTTCAGGCCGTGGAGGCCAAGGCGCTGCAGGCGGAGGTCGGGCTCCCGGTGGACCGGAACATCCCGCTGGTGGCGTTCATCGGCAGGCTGGAAGAGCAGAAGGGACCCGACGTCATGGCGGCCGCCATCCCGCAGCTCATGGAGATGGTGGAGGACGTGCAGATCGTTCTGCTGGTACGTGTGCGCCGCCCGCCACCCGGCTACTACATGCGTGTATCGTTCTACTGGAACATACGTGTGAGCAACGCGATGGATAATGCTGCAGGGCACGGGCAAGAAGAAGTTCGAGCGCATGCTCATGAGCGCCGAGGAGAAGTTCCCAGGCAAGGTGCGCGCCGTGGTCAAGTTCAACGCGGCGCTGGCGCACCACATCATGGCCGGCGCCGACGTGCTCGCCGTCACCAGCCGCTTCGAGCCCTGCGGCCTCATCCAGCTGCAGGGGATGCGATACGGAACGGTACGAGAGAGAAAAAAAAACATCCTGAATCCTGACGAGAGGGACAGAGACAGATTGATTATGAATGCTTCATCGATTTGAATTGATTGATCGATGTCTCCCGCTGCGACTCTTGCAGCCCTGCGCCTGCGCGTCCACCGGTGGACTCGTCGACACCATCATCGAAGGCAAGACCGGGTTCCACATGGGCCGCCTCAGCGTCGACGTAAGCCTAGCTCTGCCATGATCTTTCTTCTTTCTGTATGTATGTATGTATGAATCAGCACCGCCGTTCTTGTTTCGTCGTCCTCTCTTCCCAGTGCAACGTCGTGGAGCCGGCGGACGTCAAGAAGGTGGCCACCACCTTGCAGCGCGCCATCAAGGTGGTCGGCACGCCGGCGTACGAGGAGATGGTGAGGAACTGCATGATCCAGGATCTCTCCTGGAAGGTACGTACGCCCGCCCCGCCAGAGCAGAGCGCCAAGATCGATCGACCGACCGACCACACGTACGCGCCTCGCTCCTGTCGCTGACCGTGGTTTAATTTGCGAAATGCGCAGGGCCCTGCCAAGAACTGGGAGAACGTGCTGCTCAGCCTCGGGGTCGCCGGCGGCGAGCCAGGGGTCGAAGGCGAGGAGATCGCGCCGCTCGCCAAGGAGAACGTGGCCGCGCCCTGAAGAGTTCGGCCTGCAGGCCCCCTGATCTCGCGCGTGGTGCAAACATGTTGGGACATCTTCTTATATATGCTGTTTCGTTTATGTGATATGGACAAGTATGTGTAGCTGCTTGCTTGTGCTAGTGTAATATAATAGTGTAGTGGTGGCCAGTGGCACAACCTAATAAGCGCATGAACTAATTGCTTGCGTGTGTAGTTAAGTACCGATCGGTAATTTTATAT |
| CWM052 | 97.62 | CCACAACTGTTCGCGTCCTGCTGGTTCATTATCTGACCTTGATTGCATTGCAGCTACGAGAAGCCCGTGGAAGGCCGGAAGATCAACTGGATGAAGGCCGGGATCCTCGAGGCCGACAGGGTCCTCACCGTCAGCCCCTACTACGCCGAGGAGCTCATCTCCGGCATCGCCAGGGGCTGCGAGCTCGACAACATCATGCGCCTCACCGGCATCACCGGCATCGTCAACGGCATGGACGTCAGCGAGTGGGACCCCAGCAGGGACAAGTACATCGCCGTGAAGTACGACGTGTCGACGGTGAGCTGGCTAGCTAGCTGATTCTGCTGCCTGGTCCTCCTGCTCATGCTGGTTCGGTTCTGACGCGGCGAGTGTACGTACGTGCGTGCGACGGTGGTGTGGTGTCCGGTTCAGGCCGTGGAGGCCAAGGCGCTGCAGGCGGAGGTCGGGCTCCCGGTGGACCGGAACATCCCGCTGGTGGCGTTCATCGGCAGGCTGGAAGAGCAGAAGGGACCCGACGTCATGGCGGCCGCCATCCCGCAGCTCATGGAGATGGTGGAGGACGTGCAGATCGTTCTGCTGGTACGTGTGCGCCGCCCGCCACCCGGCTACTACATGCGTGTATCGTTCTACTGGAACATACGTGTGAGCAACGCGATGGATAATGCTGCAGGGCACGGGCAAGAAGAAGTTCGAGCGCATGCTCATGAGCGCCGAGGAGAAGTTCCCAGGCAAGGTGCGCGCCGTGGTCAAGTTCAACGCGGCGCTGGCGCACCACATCATGGCCGGCGCCGACGTGCTCGCCGTCACCAGCCGCTTCGAGCCCTGCGGCCTCATCCAGCTGCAGGGGATGCGATACGGAACGGTACGAGAGAGAAAAAAAAACATCCTGAATCCTGACGAGAGGGACAGAGACAGATTGATTATGAATGCTTCATCGATTTGAATTGATTGATCGATGTCTCCCGCTGCGACTCTTGCAGCCCTGCGCCTGCGCGTCCACCGGTGGACTCGTCGACACCATCATCGAAGGCAAGACCGGGTTCCACATGGGCCGCCTCAGCGTCGACGTAAGCCTAGCTCTGCCATGATCTTTCTTCTTTCTGTATGTATGTATGTATGAATCAGCACCGCCGTTCTTGTTTCGTCGTCCTCTCTTCCCAGTGCAACGTCGTGGAGCCGGCGGACGTCAAGAAGGTGGCCACCACCTTGCAGCGCGCCATCAAGGTGGTCGGCACGCCGGCGTACGAGGAGATGGTGAGGAACTGCATGATCCAGGATCTCTCCTGGAAGGTACGTACGCCCGCCCCGCCAGAGCAGAGCGCCAAGATCGATCGACCGACCGACCACACGTACGCGCCTCGCTCCTGTCGCTGACCGTGGTTTAATTTGCGAAATGCGCAGGGCCCTGCCAAGAACTGGGAGAACGTGCTGCTCAGCCTCGGGGTCGCCGGCGGCGAGCCAGGGGTCGAAGGCGAGGAGATCGCGCCGCTCGCCAAGGAGAACGTGGCCGCGCCCTGAAGAGTTCGGCCTGCAGGCCCCCTGATCTCGCGCGTGGTGCAAACATGTTGGGACATCTTCTTATATATGCTGTTTCGTTTATGTGATATGGACAAGTATGTGTAGCTGCTTGCTTGTGCTAGTGTAATATAATAGTGTAGTGGTGGCCAGTGGCACAACCTAATAAGCGCATGAACTAATTGCTTGCGTGTGTAGTTAAGTACCGATCGGTAATTTTATAT |

**Table S1 Continued**

| **Accession** | **AC(%)** | **DNA Sequence** |
| --- | --- | --- |
| CWM056 | 99.07 | CCACAACTGTTCGCGTCCTGCTGGTTCATTATCTGACCTGGATTGCATTGCAGCTACGAGAAGCCCGTGGAAGGCCGGAAGATCAACTGGATGAAGGCCGGGATCCTCGAGGCCGACAGGGTCCTCACCGTCAGCCCCTACTACGCCGAGGAGCTCATCTCCGGCATCGCCAGGGGCTGCGAGCTCGACAACATCATGCGCCTCACCGGCATCACCGGCATCGTCAACGGCATGGACGTCAGCGAGTGGGACCCCAGCAGGGACAAGTACATCGCCGTGAAGTACGACGTGTCGACGGTGAGCTGGCTAGCTAGCTGATTCTGCTGCCTGGTCCTCCTGCTCATGCTGGTTCGGTTCTGACGCGGCGAGTGTACGTACGTGCGTGCGACGGTGGTGTGGTGTCCGGTTCAGGCCGTGGAGGCCAAGGCGCTGCAGGCGGAGGTCGGGCTCCCGGTGGACCGGAACATCCCGCTGGTGGCGTTCATCGGCAGGCTGGAAGAGCAGAAGGGACCCGACGTCATGGCGGCCGCCATCCCGCAGCTCATGGAGATGGTGGAGGACGTGCAGATCGTTCTGCTGGTACGTGTGCGCCGCCCGCCACCCGGCTACTACATGCGTGTATCGTTCTACTGGAACATACGTGTGAGCAACGCGATGGATAATGCTGCAGGGCACGGGCAAGAAGAAGTTCGAGCGCATGCTCATGAGCGCCGAGGAGAAGTTCCCAGGCAAGGTGCGCGCCGTGGTCAAGTTCAACGCGGCGCTGGCGCACCACATCATGGCCGGCGCCGACGTGCTCGCCGTCACCAGCCGCTTCGAGCCCTGCGGCCTCATCCAGCTGCAGGGGATGCGATACGGAACGGTACGAGAGAGAAAAAAAAACATCCTGAATCCTGACGAGAGGGACAGAGACAGATTGATTATGAATGCTTCATCGATTTGAATTGATTGATCGATGTCTCCCGCTGCGACTCTTGCAGCCCTGCGCCTGCGCGTCCACCGGTGGACTCGTCGACACCATCATCGAAGGCAAGACCGGGTTCCACATGGGCCGCCTCAGCGTCGACGTAAGCCTAGCTCTGCCATGATCTTTCTTCTTTCTGTATGTATGTATGTATGAATCAGCACCGCCGTTCTTGTTTCGTCGTCCTCTCTTCCCAGTGCAACGTCGTGGAGCCGGCGGACGTCAAGAAGGTGGCCACCACCTTGCAGCGCGCCATCAAGGTGGTCGGCACGCCGGCGTACGAGGAGATGGTGAGGAACTGCATGATCCAGGATCTCTCCTGGAAGGTACGTACGCCCGCCCCGCCAGAGCAGAGCGCCAAGATCGATCGACCGACCGACCACACGTACGCGCCTCGCTCCTGTCGCTGACCGTGGTTTAATTTGCGAAATGCGCAGGGCCCTGCCAAGAACTGGGAGAACGTGCTGCTCAGCCTCGGGGTCGCCGGCGGCGAGCCAGGGGTCGAAGGCGAGGAGATCGCGCCGCTCGCCAAGGAGAACGTGGCCGCGCCCTGAAGAGTTCGGCCTGCAGGCCCCCTGATCTCGCGCGTGGTGCAAACATGTTGGGACATCTTCTTATATATGCTGTTTCGTTTATGTGATATGGACAAGTATGTGTAGCTGCTTGCTTGTGCTAGTGTAATATAATAGTGTAGTGGTGGCCAGTGGCACAACCTAATAAGCGCATGAACTAATTGCTTGCGTGTGTAGTTAAGTACCGATCGGTAATTTTATAT |
| CWM057 | 97.46 | CCACAACTGTTCGCGTCCTGCTGGTTCATTATCTGACCTGGATTGCATTGCAGCTACGAGAAGCCCGTGGAAGGCCGGAAGATCAACTGGATGAAGGCCGGGATCCTCGAGGCCGACAGGGTCCTCACCGTCAGCCCCTACTACGCCGAGGAGCTCATCTCCGGCATCGCCAGGGGCTGCGAGCTCGACAACATCATGCGCCTCACCGGCATCACCGGCATCGTCAACGGCATGGACGTCAGCGAGTGGGACCCCAGCAGGGACAAGTACATCGCCGTGAAGTACGACGTGTCGACGGTGAGCTGGCTAGCTAGCTGATTCTGCTGCCTGGTCCTCCTGCTCATGCTGGTTCGGTTCTGACGCGGCGAGTGTACGTACGTGCGTGCGACGGTGGTGTGGTGTCCGGTTCAGGCCGTGGAGGCCAAGGCGCTGCAGGCGGAGGTCGGGCTCCCGGTGGACCGGAACATCCCGCTGGTGGCGTTCATCGGCAGGCTGGAAGAGCAGAAGGGACCCGACGTCATGGCGGCCGCCATCCCGCAGCTCATGGAGATGGTGGAGGACGTGCAGATCGTTCTGCTGGTACGTGTGCGCCGCCCGCCACCCGGCTACTACATGCGTGTATCGTTCTACTGGAACATACGTGTGAGCAACGCGATGGATAATGCTGCAGGGCACGGGCAAGAAGAAGTTCGAGCGCATGCTCATGAGCGCCGAGGAGAAGTTCCCAGGCAAGGTGCGCGCCGTGGTCAAGTTCAACGCGGCGCTGGCGCACCACATCATGGCCGGCGCCGACGTGCTCGCCGTCACCAGCCGCTTCGAGCCCTGCGGCCTCATCCAGCTGCAGGGGATGCGATACGGAACGGTACGAGAGAGAAAAAAAAACATCCTGAATCCTGACGAGAGGGACAGAGACAGATTGATTATGAATGCTTCATCGATTTGAATTGATTGATCGATGTCTCCCGCTGCGACTCTTGCAGCCCTGCGCCTGCGCGTCCACCGGTGGACTCGTCGACACCATCATCGAAGGCAAGACCGGGTTCCACATGGGCCGCCTCAGCGTCGACGTAAGCCTAGCTCTGCCATGATCTTTCTTCTTTCTGTATGTATGTATGTATGAATCAGCACCGCCGTTCTTGTTTCGTCGTCCTCTCTTCCCAGTGCAACGTCGTGGAGCCGGCGGACGTCAAGAAGGTGGCCACCACCTTGCAGCGCGCCATCAAGGTGGTCGGCACGCCGGCGTACGAGGAGATGGTGAGGAACTGCATGATCCAGGATCTCTCCTGGAAGGTACGTACGCCCGCCCCGCCAGAGCAGAGCGCCAAGATCGATCGACCGACCGACCACACGTACGCGCCTCGCTCCTGTCGCTGACCGTGGTTTAATTTGCGAAATGCGCAGGGCCCTGCCAAGAACTGGGAGAACGTGCTGCTCAGCCTCGGGGTCGCCGGCGGCGAGCCAGGGGTCGAAGGCGAGGAGATCGCGCCGCTCGCCAAGGAGAACGTGGCCGCGCCCTGAAGAGTTCGGCCTGCAGGCCCCCTGATCTCGCGCGTGGTGCAAACATGTTGGGACATCTTCTTATATATGCTGTTTCGTTTATGTGATATGGACAAGTATGTGTAGCTGCTTGCTTGTGCTAGTGTAATATAATAGTGTAGTGGTGGCCAGTGGCACAACCTAATAAGCGCATGAACTAATTGCTTGCGTGTGTAGTTAAGTACCGATCGGTAATTTTATAT |

**Table S1 Continued**

| **Accession** | **AC(%)** | **DNA Sequence** |
| --- | --- | --- |
| CWM069 | 99.26 | CCACAACTGTTCGCGTCCTGCTGGTTCATTATCTGACCTTGATTGCATTGCAGCTACGAGAAGCCCGTGGAAGGCCGGAAGATCAACTGGATGAAGGCCGGGATCCTCGAGGCCGACAGGGTCCTCACCGTCAGCCCCTACTACGCCGAGGAGCTCATCTCCGGCATCGCCAGGGGCTGCGAGCTCGACAACATCATGCGCCTCACCGGCATCACCGGCATCGTCAACGGCATGGACGTCAGCGAGTGGGACCCCAGCAGGGACAAGTACATCGCCGTGAAGTACGACGTGTCGACGGTGAGCTGGCTAGCTAGCTGATTCTGCTGCCTGGTCCTCCTGCTCATGCTGGTTCGGTTCTGACGCGGCGAGTGTACGTACGTGCGTGCGACGGTGGTGTGGTGTCCGGTTCAGGCCGTGGAGGCCAAGGCGCTGCAGGCGGAGGTCGGGCTCCCGGTGGACCGGAACATCCCGCTGGTGGCGTTCATCGGCAGGCTGGAAGAGCAGAAGGGACCCGACGTCATGGCGGCCGCCATCCCGCAGCTCATGGAGATGGTGGAGGACGTGCAGATCGTTCTGCTGGTACGTGTGCGCCGCCCGCCACCCGGCTACTACATGCGTGTATCGTTCTACTGGAACATACGTGTGAGCAACGCGATGGATAATGCTGCAGGGCACGGGCAAGAAGAAGTTCGAGCGCATGCTCATGAGCGCCGAGGAGAAGTTCCCAGGCAAGGTGCGCGCCGTGGTCAAGTTCAACGCGGCGCTGGCGCACCACATCATGGCCGGCGCCGACGTGCTCGCCGTCACCAGCCGCTTCGAGCCCTGCGGCCTCATCCAGCTGCAGGGGATGCGATACGGAACGGTACGAGAGAGAAAAAAAAACATCCTGAATCCTGACGAGAGGGACAGAGACAGATTGATTATGAATGCTTCATCGATTTGAATTGATTGATCGATGTCTCCCGCTGCGACTCTTGCAGCCCTGCGCCTGCGCGTCCACCGGTGGACTCGTCGACACCATCATCGAAGGCAAGACCGGGTTCCACATGGGCCGCCTCAGCGTCGACGTAAGCCTAGCTCTGCCATGATCTTTCTTCTTTCTGTATGTATGTATGTATGAATCAGCACCGCCGTTCTTGTTTCGTCGTCCTCTCTTCCCAGTGCAACGTCGTGGAGCCGGCGGACGTCAAGAAGGTGGCCACCACCTTGCAGCGCGCCATCAAGGTGGTCGGCACGCCGGCGTACGAGGAGATGGTGAGGAACTGCATGATCCAGGATCTCTCCTGGAAGGTACGTACGCCCGCCCCGCCAGAGCAGAGCGCCAAGATCGATCGACCGACCGACCACACGTACGCGCCTCGCTCCTGTCGCTGACCGTGGTTTAATTTGCGAAATGCGCAGGGCCCTGCCAAGAACTGGGAGAACGTGCTGCTCAGCCTCGGGGTCGCCGGCGGCGAGCCAGGGGTCGAAGGCGAGGAGATCGCGCCGCTCGCCAAGGAGAACGTGGCCGCGCCCTGAAGAGTTCGGCCTGCAGGCCCCCTGATCTCGCGCGTGGTGCAAACATGTTGGGACATCTTCTTATATATGCTGTTTCGTTTATGTGATATGGACAAGTATGTGTAGCTGCTTGCTTGTGCTAGTGTAATATAATAGTGTAGTGGTGGCCAGTGGCACAACCTAATAAGCGCATGAACTAATTGCTTGCGTGTGTAGTTAAGTACCGATCGGTAATTTTATAT |
| CWM074 | 95.45 | CCACAACTGTTCGCGTCCTGCTGGTTCATTATCTGACCTTGATTGCATTGCAGCTACGAGAAGCCCGTGGAAGGCCGGAAGATCAACTGGATGAAGGCCGGGATCCTCGAGGCCGACAGGGTCCTCACCGTCAGCCCCTACTACGCCGAGGAGCTCATCTCCGGCATCGCCAGGGGCTGCGAGCTCGACAACATCATGCGCCTCACCGGCATCACCGGCATCGTCAACGGCATGGACGTCAGCGAGTGGGACCCCAGCAGGGACAAGTACATCGCCGTGAAGTACGACGTGTCGACGGTGAGATGTCTAGCTAGCTGATTCTGCTGCCTGGTCCTCCTGCTCATGCTGGTTCGGTTCTGACGCGGCGAGTGTACGTACGTGCGTGCGACGGTGGTGTGGTGTCCGGTTCAGGCCGTGGAGGCCAAGGCGCTGCAGGCGGAGGTCGGGCTCCCGGTGGACCGGAACATCCCGCTGGTGGCGTTCATCGGCAGGCTGGAAGAGCAGAAGGGACCCGACGTCATGGCGGCCGCCATCCCGCAGCTCATGGAGATGGTGGAGGACGTGCAGATCGTTCTGCTGGTACGTGTGCGCCGCCCGCCACCCGGCTACTACATGCGTGTATCGTTCTACTGGAACATACGTGTGAGCAACGCGATGGATAATGCTGCAGGGCACGGGCAAGAAGAAGTTCGAGCGCATGCTCATGAGCGCCGAGGAGAAGTTCCCAGGCAAGGTGCGCGCCGTGGTCAAGTTCAACGCGGCGCTGGCGCACCACATCATGGCCGGCGCCGACGTGCTCGCCGTCACCAGCCGCTTCGAGCCCTGCGGCCTCATCCAGCTGCAGGGGATGCGATACGGAACGGTACGAGAGAGAAAAAAAAACATCCTGAATCCTGACGAGAGGGACAGAGACAGATTGATTATGAATGCTTCATCGATTTGAATTGATTGATCGATGTCTCCCGCTGCGACTCTTGCAGCCCTGCGCCTGCGCGTCCACCGGTGGACTCGTCGACACCATCATCGAAGGCAAGACCGGGTTCCACATGGGCCGCCTCAGCGTCGACGTAAGCCTAGCTCTGCCATGATCTTTCTTCTTTCTGTATGTATGTATGTATGAATCAGCACCGCCGTTCTTGTTTCGTCGTCCTCTCTTCCCAGTGCAACGTCGTGGAGCCGGCGGACGTCAAGAAGGTGGCCACCACCTTGCAGCGCGCCATCAAGGTGGTCGGCACGCCGGCGTACGAGGAGATGGTGAGGAACTGCATGATCCAGGATCTCTCCTGGAAGGTACGTACGCCCGCCCCGCCAGAGCAGAGCGCCAAGATCGATCGACCGACCGACCACACGTACGCGCCTCGCTCCTGTCGCTGACCGTGGTTTAATTTGCGAAATGCGCAGGGCCCTGCCAAGAACTGGGAGAACGTGCTGCTCAGCCTCGGGGTCGCCGGCGGCGAGCCAGGGGTCGAAGGCGAGGAGATCGCGCCGCTCGCCAAGGAGAACGTGGCCGCGCCCTGAAGAGTTCGGCCTGCAGGCCCCCTGATCTCGCGCGTGGTGCAAACATGTTGGGACATCTTCTTATATATGCTGTTTCGTTTATGTGATATGGACAAGTATGTGTAGCTGCTTGCTTGTGCTAGTGTAATATAATAGTGTAGTGGTGGCCAGTGGCACAACCTAATAAGCGCATGAACTAATTGCTTGCGTGTGTAGTTAAGTACCGATCGGTAATTTTATAT |

**Table S1 Continued**

| **Accession** | **AC(%)** | **DNA Sequence** |
| --- | --- | --- |
| CWM080 | 96.79 | CCACAACTGTTCGCGTCCTGCTGGTTCATTATCTGACCTTGATTGCATTGCAGCTACGAGAAGCCCGTGGAAGGCCGGAAGATCAACTGGATGAAGGCCGGGATCCTCGAGGCCGACAGGGTCCTCACCGTCAGCCCCTACTACGCCGAGGAGCTCATCTCCGGCATCGCCAGGGGCTGCGAGCTCGACAACATCATGCGCCTCACCGGCATCACCGGCATCGTCAACGGCATGGACGTCAGCGAGTGGGACCCCAGCAGGGACAAGTACATCGCCGTGAAGTACGACGTGTCGACGGTGAGCTGGCTAGCTAGCTGATTCTGCTGCCTGGTCCTCCTGCTCATGCTGGTTCGGTTCTGACGCGGCGAGTGTACGTACGTGCGTGCGACGGTGGTGTGGTGTCCGGTTCAGGCCGTGGAGGCCAAGGCGCTGCAGGCGGAGGTCGGGCTCCCGGTGGACCGGAACATCCCGCTGGTGGCGTTCATCGGCAGGCTGGAAGAGCAGAAGGGACCCGACGTCATGGCGGCCGCCATCCCGCAGCTCATGGAGATGGTGGAGGACGTGCAGATCGTTCTGCTGGTACGTGTGCGCCGCCCGCCACCCGGCTACTACATGCGTGTATCGTTCTACTGGAACATACGTGTGAGCAACGCGATGGATAATGCTGCAGGGCACGGGCAAGAAGAAGTTCGAGCGCATGCTCATGAGCGCCGAGGAGAAGTTCCCAGGCAAGGTGCGCGCCGTGGTCAAGTTCAACGCGGCGCTGGCGCACCACATCATGGCCGGCGCCGACGTGCTCGCCGTCACCAGCCGCTTCGAGCCCTGCGGCCTCATCCAGCTGCAGGGGATGCGATACGGAACGGTACGAGAGAGAAAAAAAAACATCCTGAATCCTGACGAGAGGGACAGAGACAGATTGATTATGAATGCTTCATCGATTTGAATTGATTGATCGATGTCTCCCGCTGCGACTCTTGCAGCCCTGCGCCTGCGCGTCCACCGGTGGACTCGTCGACACCATCATCGAAGGCAAGACCGGGTTCCACATGGGCCGCCTCAGCGTCGACGTAAGCCTAGCTCTGCCATGATCTTTCTTCTTTCTGTATGTATGTATGTATGAATCAGCACCGCCGTTCTTGTTTCGTCGTCCTCTCTTCCCAGTGCAACGTCGTGGAGCCGGCGGACGTCAAGAAGGTGGCCACCACCTTGCAGCGCGCCATCAAGGTGGTCGGCACGCCGGCGTACGAGGAGATGGTGAGGAACTGCATGATCCAGGATCTCTCCTGGAAGGTACGTACGCCCGCCCCGCCAGAGCAGAGCGCCAAGATCGATCGACCGACCGACCACACGTACGCGCCTCGCTCCTGTCGCTGACCGTGGTTTAATTTGCGAAATGCGCAGGGCCCTGCCAAGAACTGGGAGAACGTGCTGCTCAGCCTCGGGGTCGCCGGCGGCGAGCCAGGGGTCGAAGGCGAGGAGATCGCGCCGCTCGCCAAGGAGAACGTGGCCGCGCCCTGAAGAGTTCGGCCTGCAGGCCCCCTGATCTCGCGCGTGGTGCAAACATGTTGGGACATCTTCTTATATATGCTGTTTCGTTTATGTGATATGGACAAGTATGTGTAGCTGCTTGCTTGTGCTAGTGTAATATAATAGTGTAGTGGTGGCCAGTGGCACAACCTAATAAGCGCATGAACTAATTGCTTGCGTGTGTAGTTAAGTACCGATCGGTAATTTTATAT |
| SWM011 | 97.29 | CCACAACTGTTCGCGTCCTGCTGGTTCATTATCTGACCTGATTGCATTATTGCAGCTACGAGAAGCCCGTGGAAGGCCGGAAGATCAACTGGATGAAGGCCGGGATCCTCGAGGCCGACAGGGTCCTCACCGTCAGCCCCTACTACGCCGAGGAGCTCATCTCCGGCATCGCCAGGGGCTGCGAGCTCGACAACATCATGCGCCTCACCGGCATCACCGGCATCGTCAACGGCATGGACGTCAGCGAGTGGGACCCCAGCAGGGACAAGTACATCGCCGTGAAGTACGACGTGTCGACGGTGAGCTGGCTAGCTAGCTGATTCTGCTGCCTGGTCCTCCTGCTCATGCTGGTTCGGTTCTGACGCGGCAAGTGTACGTACGTGCGTGCGACGGTGGTGTGGTGTCCGGTTCAGGCCGTGGAGGCCAAGGCGCTGAACAAGGAGGCGCTGCAGGCGGAGGTCGGGCTCCCGGTGGACCGGAACATCCCGCTGGTGGCGTTCATCGGCAGGCTGGAAGAGCAGAAGGGACCCGACGTCATGGCGGCCGCCATCCCGCAGCTCATGGAGATGGTGGAGGACGTGCAGATCGTTCTGCTGGTACGTGTGCGCCGCCCGCCACCCGGCTACTACATGCGTGTATCGTTCGTTCTACTGGAACATGCGTGTGAGCAACGCGATGGATAATGCTGCAGGGCACGGGCAAGAAGAAGTTCGAGCGCATGCTCATGAGCGCCGAGGAGAAGTTCCCAGGCAAGGTGCGCGCCGTGGTCAAGTTCAACGCGGCGCTGGCGCACCACATCATGGCCGGCGCCGACGTGCTCGCCGTCACCAGCCGCTTCGAGCCCTGCGGCCTCATCCAGCTGCAGGGGATGCGATACGGAACGGTACGAGAGAGAAAAAAAAACATCCTGAATCCTGACGAGAGGGACAGAGACAGATTGATTATGAATGCTTCATCGATTTGAATTGATTGATCGATGTCTCCCGCTGCGACTCTTGCAGCCCTGCGCCTGCGCGTCCACCGGTGGACTCGTCGACACCATCATCGAAGGCAAGACCGGGTTCCACATGGGCCGCCTCAGCGTCGACGTAAGCCTACCTCTGCCATGTTCTTTCTTCTTTCTTTCTGTATGTATGTATGTATGTACGAATCAGCACCGCCATTCTTGTTTCGTCGTCCTCTCTTCCCAGTGCAACGTCGTGGAGCCGGCGGACGTCAAGAAGGTGGCCACCACCTTGCAGCGCGCCATCAAGGTGGTCGGCACGCCGGCGTACGAGGAGATGGTGAGGAACTGCATGATCCAGGATCTCTCCTGGAAGGTACGTACGCCCGCCCCGCCAGAGCAGAGCGCCAAGATCGATCGACCGACCGACCACACGTACGCGCCTCGCTCCTGTCGCTGACCGTGGTTTAATTTGCGAAATGCGCAGGGCCCTGCCAAGAACTGGGAGAACGTGCTGCTCAGCCTCGGGGTCGCTGGCGGCGAGCCAGGGGTCGAAGGCGAGGAGATCGCGCCGCTCGCCAAGGAGAACGTGGCCGCGCCCTGAAGAGTTCGGCCTGCAGGGCCCCTGATCTCGCGCGTGGTGCAAAGATGTTGGGACATCTTCTTATATATGCTGTTTCGTTTATGTGATATGGACAAGTATGTGTAGCTGCTTGCTTGTGCTAGTGTAATATAGTGTAGTGGTGGCCAGTGGCACAACCTAATAAGCGCATGAACTAATTGCTTGCGTGTGTAGTTAAGTACCGATCGGTAATTTTATAT |

**Table S1 Continued**

| **Accession** | **AC(%)** | **DNA Sequence** |
| --- | --- | --- |
| SWM012 | 98.57 | CCACAACTGTTCGCGTCCTGCTGGTTCATTATCTGACCTGATTGCATTATTGCAGCTACGAGAAGCCCGTGGAAGGCCGGAAGATCAACTGGATGAAGGCCGGGATCCTCGAGGCCGACAGGGTCCTCACCGTCAGCCCCTACTACGCCGAGGAGCTCATCTCCGGCATCGCCAGGGGCTGCGAGCTCGACAACATCATGCGCCTCACCGGCATCACCGGCATCGTCAACGGCATGGACGTCAGCGAGTGGGACCCCAGCAGGGACAAGTACATCGCCGTGAAGTACGACGTGTCGACGGTGAGCTGGCTAGCTAGCTGATTCTGCTGCCTGGTCCTCCTGCTCATGCTGGTTCGGTTCTGACGCGGCAAGTGTACGTACGTGCGTGCGACGGTGGTGTGGTGTCCGGTTCAGGCCGTGGAGGCCAAGGCGCTGAACAAGGAGGCGCTGCAGGCGGAGGTCGGGCTCCCGGTGGACCGGAACATCCCGCTGGTGGCGTTCATCGGCAGGCTGGAAGAGCAGAAGGGACCCGACGTCATGGCGGCCGCCATCCCGCAGCTCATGGAGATGGTGGAGGACGTGCAGATCGTTCTGCTGGTACGTGTGCGCCGCCCGCCACCCGGCTACTACATGCGTGTATCGTTCGTTCTACTGGAACATGCGTGTGAGCAACGCGATGGATAATGCTGCAGGGCACGGGCAAGAAGAAGTTCGAGCGCATGCTCATGAGCGCCGAGGAGAAGTTCCCAGGCAAGGTGCGCGCCGTGGTCAAGTTCAACGCGGCGCTGGCGCACCACATCATGGCCGGCGCCGACGTGCTCGCCGTCACCAGCCGCTTCGAGCCCTGCGGCCTCATCCAGCTGCAGGGGATGCGATACGGAACGGTACGAGAGAGAAAAAAAAACATCCTGAATCCTGACGAGAGGGACAGAGACAGATTGATTATGAATGCTTCATCGATTTGAATTGATTGATCGATGTCTCCCGCTGCGACTCTTGCAGCCCTGCGCCTGCGCGTCCACCGGTGGACTCGTCGACACCATCATCGAAGGCAAGACCGGGTTCCACATGGGCCGCCTCAGCGTCGACGTAAGCCTACCTCTGCCATGTTCTTTCTTCTTTCTTTCTGTATGTATGTATGTATGTACGAATCAGCACCGCCATTCTTGTTTCGTCGTCCTCTCTTCCCAGTGCAACGTCGTGGAGCCGGCGGACGTCAAGAAGGTGGCCACCACCTTGCAGCGCGCCATCAAGGTGGTCGGCACGCCGGCGTACGAGGAGATGGTGAGGAACTGCATGATCCAGGATCTCTCCTGGAAGGTACGTACGCCCGCCCCGCCAGAGCAGAGCGCCAAGATCGATCGACCGACCGACCACACGTACGCGCCTCGCTCCTGTCGCTGACCGTGGTTTAATTTGCGAAATGCGCAGGGCCCTGCCAAGAACTGGGAGAACGTGCTGCTCAGCCTCGGGGTCGCTGGCGGCGAGCCAGGGGTCGAAGGCGAGGAGATCGCGCCGCTCGCCAAGGAGAACGTGGCCGCGCCCTGAAGAGTTCGGCCTGCAGGGCCCCTGATCTCGCGCGTGGTGCAAAGATGTTGGGACATCTTCTTATATATGCTGTTTCGTTTATGTGATATGGACAAGTATGTGTAGCTGCTTGCTTGTGCTAGTGTAATATAGTGTAGTGGTGGCCAGTGGCACAACCTAATAAGCGCATGAACTAATTGCTTGCGTGTGTAGTTAAGTACCGATCGGTAATTTTATAT |
| SWM017 | 98.22 | CCACAACTGTTCGCGTCCTGCTGGTTCATTATCTGACCTGATTGCATTATTGCAGCTACGAGAAGCCCGTGGAAGGCCGGAAGATCAACTGGATGAAGGCCGGGATCCTCGAGGCCGACAGGGTCCTCACCGTCAGCCCCTACTACGCCGAGGAGCTCATCTCCGGCATCGCCAGGGGCTGCGAGCTCGACAACATCATGCGCCTCACCGGCATCACCGGCATCGTCAACGGCATGGACGTCAGCGAGTGGGACCCCAGCAGGGACAAGTACATCGCCGTGAAGTACGACGTGTCGACGGTGAGCTGGCTAGCTAGCTGATTCTGCTGCCTGGTCCTCCTGCTCATGCTGGTTCGGTTCTGACGCGGCAAGTGTACGTACGTGCGTGCGACGGTGGTGTGGTGTCCGGTTCAGGCCGTGGAGGCCAAGGCGCTGAACAAGGAGGCGCTGCAGGCGGAGGTCGGGCTCCCGGTGGACCGGAACATCCCGCTGGTGGCGTTCATCGGCAGGCTGGAAGAGCAGAAGGGACCCGACGTCATGGCGGCCGCCATCCCGCAGCTCATGGAGATGGTGGAGGACGTGCAGATCGTTCTGCTGGTACGTGTGCGCCGCCCGCCACCCGGCTACTACATGCGTGTATCGTTCGTTCTACTGGAACATGCGTGTGAGCAACGCGATGGATAATGCTGCAGGGCACGGGCAAGAAGAAGTTCGAGCGCATGCTCATGAGCGCCGAGGAGAAGTTCCCAGGCAAGGTGCGCGCCGTGGTCAAGTTCAACGCGGCGCTGGCGCACCACATCATGGCCGGCGCCGACGTGCTCGCCGTCACCAGCCGCTTCGAGCCCTGCGGCCTCATCCAGCTGCAGGGGATGCGATACGGAACGGTACGAGAGAGAAAAAAAAACATCCTGAATCCTGACGAGAGGGACAGAGACAGATTGATTATGAATGCTTCATCGATTTGAATTGATTGATCGATGTCTCCCGCTGCGACTCTTGCAGCCCTGCGCCTGCGCGTCCACCGGTGGACTCGTCGACACCATCATCGAAGGCAAGACCGGGTTCCACATGGGCCGCCTCAGCGTCGACGTAAGCCTACCTCTGCCATGTTCTTTCTTCTTTCTTTCTGTATGTATGTATGTATGTACGAATCAGCACCGCCATTCTTGTTTCGTCGTCCTCTCTTCCCAGTGCAACGTCGTGGAGCCGGCGGACGTCAAGAAGGTGGCCACCACCTTGCAGCGCGCCATCAAGGTGGTCGGCACGCCGGCGTACGAGGAGATGGTGAGGAACTGCATGATCCAGGATCTCTCCTGGAAGGTACGTACGCCCGCCCCGCCAGAGCAGAGCGCCAAGATCGATCGACCGACCGACCACACGTACGCGCCTCGCTCCTGTCGCTGACCGTGGTTTAATTTGCGAAATGCGCAGGGCCCTGCCAAGAACTGGGAGAACGTGCTGCTCAGCCTCGGGGTCGCTGGCGGCGAGCCAGGGGTCGAAGGCGAGGAGATCGCGCCGCTCGCCAAGGAGAACGTGGCCGCGCCCTGAAGAGTTCGGCCTGCAGGGCCCCTGATCTCGCGCGTGGTGCAAAGATGTTGGGACATCTTCTTATATATGCTGTTTCGTTTATGTGATATGGACAAGTATGTGTAGCTGCTTGCTTGTGCTAGTGTAATATAGTGTAGTGGTGGCCAGTGGCACAACCTAATAAGCGCATGAACTAATTGCTTGCGTGTGTAGTTAAGTACCGATCGGTAATTTTATAT |

**Table S1 Continued**

| **Accession** | **AC(%)** | **DNA Sequence** |
| --- | --- | --- |
| SWL089 | 99.83 | CCACAACTGTTCGCGTCCTGCTGGTTCATTATCTGACCTGATTGCATTATTGCAGCTACGAGAAGCCCGTGGAAGGCCGGAAGATCAACTGGATGAAGGCCGGGATCCTCGAGGCCGACAGGGTCCTCACCGTCAGCCCCTACTACGCCGAGGAGCTCATCTCCGGCATCGCCAGGGGCTGCGAGCTCGACAACATCATGCGCCTCACCGGCATCACCGGCATCGTCAACGGCATGGACGTCAGCGAGTGGGACCCCAGCAGGGACAAGTACATCGCCGTGAAGTACGACGTGTCGACGGTGAGCTGGCTAGCTAGCTGATTCTGCTGCCTGGTCCTCCTGCTCATGCTGGTTCGGTTCTGACGCGGCAAGTGTACGTACGTGCGTGCGACGGTGGTGTGGTGTCCGGTTCAGGCCGTGGAGGCCAAGGCGCTGAACAAGGAGGCGCTGCAGGCGGAGGTCGGGCTCCCGGTGGACCGGAACATCCCGCTGGTGGCGTTCATCGGCAGGCTGGAAGAGCAGAAGGGACCCGACGTCATGGCGGCCGCCATCCCGCAGCTCATGGAGATGGTGGAGGACGTGCAGATCGTTCTGCTGGTACGTGTGCGCCGCCCGCCACCCGGCTACTACATGCGTGTATCGTTCGTTCTACTGGAACATGCGTGTGAGCAACGCGATGGATAATGCTGCAGGCACGGGCAAGAAGAAGTTCGAGCGCATGCTCATGAGCGCCGAGGAGAAGTTCCCAGGCAAGGTGCGCGCCGTGgTCAAGTTCAACGCGGCGCTGGCGCACCACATCATGGCGGCGCCGACGTGCTCGCCGTCACCAGCCGCTTCGAGCCCTGCGGCCTCATCCAGCTGCAGGGGATGCGATACGGAACGGTACGAGAGAGAAAAAAAAACATCCTGAATCCTGACGAGAGGGACAGAGACAGATTGATTATGAATGCTTCATCGATTTGAATTGATTGATCGATGTCTCCCGCTGCGACTCTTGCAGCCCTGCGCCTGCGCGTCCACCGGTGGACTCGTCGACACCATCATCGAAGGCAAGACCGGGTTCCACATGGGCCGCCTCAGCGTCGACGTAAGCCTACCTCTGCCATGTTCTTTCTTCTTTCTTTCTGTATGTATGTATGTATGTACGAATCAGCACCGCCATTCTTGTTTCGTCGTCCTCTCTTCCCAGTGCAACGTCGTGGAGCCGGCGGACGTCAAGAAGGTGGCCACCACCTTGCAGCGCGCCATCAAGGTGGTCGGCACGCCGGCGTACGAGGAGATGGTGAGGAACTGCATGATCCAGGATCTCTCCTGGAAGGTACGTACGCCCGCCCCGCCAGAGCAGAGCGCCAAGATCGATCGACCGACCGACCACACGTACGCGCCTCGCTCCTGTCGCTGACCGTGGTTTAATTTGCGAAATGCGCAGGGCCCTGCCAAGAACTGGGAGAACGTGCTGCTCAGCCTCGGGGTCGCTGGCGGCGAGCCAGGGGTCGAAGGCGAGGAGATCGCGCCGCTCGCCAAGGAGAACGTGGCCGCGCCCTGAAGAGTTCGGCCTGCAGGGCCCCTGATCTCGCGCGTGGTGCAAAGATGTTGGGACATCTTCTTATATATGCTGTTTCGTTTATGTGATATGGACAAGTATGTGTAGCTGCTTGCTTGTGCTAGTGTAATATAGTGTAGTGGTGGCCAGTGGCACAACCTAATAAGCGCATGAACTAATTGCTTGCGTGTGTAGTTAAGTACCGATCGGTAATTTTATAT |
| SWL094 | 99.00 | CCACAACTGTTCGCGTCCTGCTGGTTCATTATCTGACCTGATTGCATTATTGCAGCTACGAGAAGCCCGTGGAAGGCCGGAAGATCAACTGGATGAAGGCCGGGATCCTCGAGGCCGACAGGGTCCTCACCGTCAGCCCCTACTACGCCGAGGAGCTCATCTCCGGCATCGCCAGGGGCTGCGAGCTCGACAACATCATGCGCCTCACCGGCATCACCGGCATCGTCAACGGCATGGACGTCAGCGAGTGGGACCCCAGCAGGGACAAGTACATCGCCGTGAAGTACGACGTGTCGACGGTGAGCTGGCTAGCTAGCTGATTCTGCTGCCTGGTCCTCCTGCTCATGCTGGTTCGGTTCTGACGCGGCAAGTGTACGTACGTGCGTGCGACGGTGGTGTGGTGTCCGGTTCAGGCCGTGGAGGCCAAGGCGCTGAACAAGGAGGCGCTGCAGGCGGAGGTCGGGCTCCCGGTGGACCGGAACATCCCGCTGGTGGCGTTCATCGGCAGGCTGGAAGAGCAGAAGGGACCCGACGTCATGGCGGCCGCCATCCCGCAGCTCATGGAGATGGTGGAGGACGTGCAGATCGTTCTGCTGGTACGTGTGCGCCGCCCGCCACCCGGCTACTACATGCGTGTATCGTTCGTTCTACTGGAACATGCGTGTGAGCAACGCGATGGATAATGCTGCAGGGCACGGGCAAGAAGAAGTTCGAGCGCATGCTCATGAGCGCCGAGGAGAAGTTCCCAGGCAAGGTGCGCGCCGTGGTCAAGTTCAACGCGGCGCTGGCGCACCACATCATGGCCGGCGCCGACGTGCTCGCCGTCACCAGCCGCTTCGAGCCCTGCGGCCTCATCCAGCTGCAGGGGATGCGATACGGAACGGTACGAGAGAGAAAAAAAAACATCCTGAATCCTGACGAGAGGGACAGAGACAGATTGATTATGAATGCTTCATCGATTTGAATTGATTGATCGATGTCTCCCGCTGCGACTCTTGCAGCCCTGCGCCTGCGCGTCCACCGGTGGACTCGTCGACACCATCATCGAAGGCAAGACCGGGTTCCACATGGGCCGCCTCAGCGTCGACGTAAGCCTACCTCTGCCATGTTCTTTCTTCTTTCTTTCTGTATGTATGTATGTATGTACGAATCAGCACCGCCATTCTTGTTTCGTCGTCCTCTCTTCCCAGTGCAACGTCGTGGAGCCGGCGGACGTCAAGAAGGTGGCCACCACCTTGCAGCGCGCCATCAAGGTGGTCGGCACGCCGGCGTACGAGGAGATGGTGAGGAACTGCATGATCCAGGATCTCTCCTGGAAGGTACGTACGCCCGCCCCGCCAGAGCAGAGCGCCAAGATCGATCGACCGACCGACCACACGTACGCGCCTCGCTCCTGTCGCTGACCGTGGTTTAATTTGCGAAATGCGCAGGGCCCTGCCAAGAACTGGGAGAACGTGCTGCTCAGCCTCGGGGTCGCTGGCGGCGAGCCAGGGGTCGAAGGCGAGGAGATCGCGCCGCTCGCCAAGGAGAACGTGGCCGCGCCCTGAAGAGTTCGGCCTGCAGGGCCCCTGATCTCGCGCGTGGTGCAAAGATGTTGGGACATCTTCTTATATATGCTGTTTCGTTTATGTGATATGGACAAGTATGTGTAGCTGCTTGCTTGTGCTAGTGTAATATAGTGTAGTGGTGGCCAGTGGCACAACCTAATAAGCGCATGAACTAATTGCTTGCGTGTGTAGTTAAGTACCGATCGGTAATTTTATAT |

**Table S1 Continued**

| **Accession** | **AC(%)** | **DNA Sequence** |
| --- | --- | --- |
| SWL097 | 97.40 | CCACAACTGTTCGCGTCCTGCTGGTTCATTATCTGACCTGATTGCATTATTGCAGCTACGAGAAGCCCGTGGAAGGCCGGAAGATCAACTGGATGAAGGCCGGGATCCTCGAGGCCGACAGGGTCCTCACCGTCAGCCCCTACTACGCCGAGGAGCTCATCTCCGGCATCGCCAGGGGCTGCGAGCTCGACAACATCATGCGCCTCACCGGCATCACCGGCATCGTCAACGGCATGGACGTCAGCGAGTGGGACCCCAGCAGGGACAAGTACATCGCCGTGAAGTACGACGTGTCGACGGTGAGCTGGCTAGCTAGCTGATTCTGCTGCCTGGTCCTCCTGCTCATGCTGGTTCGGTTCTGACGCGGCAAGTGTACGTACGTGCGTGCGACGGTGGTGTGGTGTCCGGTTCAGGCCGTGGAGGCCAAGGCGCTGAACAAGGAGGCGCTGCAGGCGGAGGTCGGGCTCCCGGTGGACCGGAACATCCCGCTGGTGGCGTTCATCGGCAGGCTGGAAGAGCAGAAGGGACCCGACGTCATGGCGGCCGCCATCCCGCAGCTCATGGAGATGGTGGAGGACGTGCAGATCGTTCTGCTGGTACGTGTGCGCCGCCCGCCACCCGGCTACTACATGCGTGTATCGTTCGTTCTACTGGAACATGCGTGTGAGCAACGCGATGGATAATGCTGCAGGGCACGGGCAAGAAGAAGTTCGAGCGCATGCTCATGAGCGCCGAGGAGAGTTCCCAGGCAAGGTGCGCGCCGTGGTCAAGTTCAACGCGGCGCTGGCGCACCACATCATGGCCGGCGCCGACGTGCTCGCCGTCACCAGCCGCTTCGAGCCCTGCGGCCTCATCCAGCTGCAGGGGATGCGATACGGAACGGTACGAGAGAGAAAAAAAAACATCCTGAATCCTGACGAGAGGGACAGAGACAGATTGATTATGAATGCTTCATCGATTTGAATTGATTGATCGATGTCTCCCGCTGCGACTCTTGCAGCCCTGCGCCTGCGCGTCCACCGGTGGACTCGTCGACACCATCATCGAAGGCAAGACCGGGTTCCACATGGGCCGCCTCAGCGTCGACGTAAGCCTACCTCTGCCATGTTCTTTCTTCTTTCTTTCTGTATGTATGTATGTATGTACGAATCAGCACCGCCATTCTTGTTTCGTCGTCCTCTCTTCCCAGTGCAACGTCGTGGAGCCGGCGGACGTCAAGAAGGTGGCCACCACCTTGCAGCGCGCCATCAAGGTGGTCGGCACGCCGGCGTACGAGGAGATGGTGAGGAACTGCATGATCCAGGATCTCTCCTGGAAGGTACGTACGCCCGCCCCGCCAGAGCAGAGCGCCAAGATCGATCGACCGACCGACCACACGTACGCGCCTCGCTCCTGTCGCTGACCGTGGTTTAATTTGCGAAATGCGCAGGGCCCTGCCAAGAACTGGGAGAACGTGCTGCTCAGCCTCGGGGTCGCTGGCGGCGAGCCAGGGGTCGAAGGCGAGGAGATCGCGCCGCTCGCCAAGGAGAACGTGGCCGCGCCCTGAAGAGTTCGGCCTGCAGGGCCCCTGATCTCGCGCGTGGTGCAAAGATGTTGGGACATCTTCTTATATATGCTGTTTCGTTTATGTGATATGGACAAGTATGTGTAGCTGCTTGCTTGTGCTAGTGTAATATAGTGTAGTGGTGGCCAGTGGCACAACCTAATAAGCGCATGAACTAATTGCTTGCGTGTGTAGTTAAGTACCGATCGGTAATTTTATAT |
| SWL102 | 99.33 | CCACAACTGTTCGCGTCCTGCTGGTTCATTATCTGACCTGATTGCATTATTGCAGCTACGAGAAGCCCGTGGAAGGCCGGAAGATCAACTGGATGAAGGCCGGGATCCTCGAGGCCGACAGGGTCCTCACCGTCAGCCCCTACTACGCCGAGGAGCTCATCTCCGGCATCGCCAGGGGCTGCGAGCTCGACAACATCATGCGCCTCACCGGCATCACCGGCATCGTCAACGGCATGGACGTCAGCGAGTGGGACCCCAGCAGGGACAAGTACATCGCCGTGAAGTACGACGTGTCGACGGTGAGCTGGCTAGCTAGCTGATTCTGCTGCCTGGTCCTCCTGCTCATGCTGGTTCGGTTCTGACGCGGCAAGTGTACGTACGTGCGTGCGACGGTGGTGTGGTGTCCGGTTCAGGCCGTGGAGGCCAAGGCGCTGAACAAGGAGGCGCTGCAGGCGGAGGTCGGGCTCCCGGTGGACCGGAACATCCCGCTGGTGGCGTTCATCGGCAGGCTGGAAGAGCAGAAGGGACCCGACGTCATGGCGGCCGCCATCCCGCAGCTCATGGAGATGGTGGAGGACGTGCAGATCGTTCTGCTGGTACGTGTGCGCCGCCCGCCACCCGGCTACTACATGCGTGTATCGTTCGTTCTACTGGAACATGCGTGTGAGCAACGCGATGGATAATGCTGCAGGGCACGGGCAAGAAGAAGTTCGAGCGCATGCTCATGAGCGCCGAGGAGAAGTTCCCAGGCAAGGTGCGCGCCGTGGTCAAGTTCAACGCGGCGCTGGCGCACCACATCATGGCCGGCGCCGACGTGCTCGCCGTCACCAGCCGCTTCGAGCCCTGCGGCCTCATCCAGCTGCAGGGGATGCGATACGGAACGGTACGAGAGAGAAAAAAAAACATCCTGAATCCTGACGAGAGGGACAGAGACAGATTGATTATGAATGCTTCATCGATTTGAATTGATTGATCGATGTCTCCCGCTGCGACTCTTGCAGCCCTGCGCCTGCGCGTCCACCGGTGGACTCGTCGACACCATCATCGAAGGCAAGACCGGGTTCCACATGGGCCGCCTCAGCGTCGACGTAAGCCTACCTCTGCCATGTTCTTTCTTCTTTCTTTCTGTATGTATGTATGTATGTACGAATCAGCACCGCCATTCTTGTTTCGTCGTCCTCTCTTCCCAGTGCAACGTCGTGGAGCCGGCGGACGTCAAGAAGGTGGCCACCACCTTGCAGCGCGCCATCAAGGTGGTCGGCACGCCGGCGTACGAGGAGATGGTGAGGAACTGCATGATCCAGGATCTCTCCTGGAAGGTACGTACGCCCGCCCCGCCAGAGCAGAGCGCCAAGATCGATCGACCGACCGACCACACGTACGCGCCTCGCTCCTGTCGCTGACCGTGGTTTAATTTGCGAAATGCGCAGGGCCCTGCCAAGAACTGGGAGAACGTGCTGCTCAGCCTCGGGGTCGCTGGCGGCGAGCCAGGGGTCGAAGGCGAGGAGATCGCGCCGCTCGCCAAGGAGAACGTGGCCGCGCCCTGAAGAGTTCGGCCTGCAGGGCCCCTGATCTCGCGCGTGGTGCAAAGATGTTGGGACATCTTCTTATATATGCTGTTTCGTTTATGTGATATGGACAAGTATGTGTAGCTGCTTGCTTGTGCTAGTGTAATATAGTGTAGTGGTGGCCAGTGGCACAACCTAATAAGCGCATGAACTAATTGCTTGCGTGTGTAGTTAAGTACCGATCGGTAATTTTATAT |

**Table S1 Continued**

| **Accession** | **AC(%)** | **DNA Sequence** |
| --- | --- | --- |
| SWL105 | 97.39 | CCACAACTGTTCGCGTCCTGCTGGTTCATTATCTGACCTGATTGCATTATGCAGCTACGAGAAGCCCGTGGAAGGCCGGAAGATCAACTGGATGAAGGCCGGGATCCTCGAGGCCGACAGGGTCCTCACCGTCAGCCCCTACTACGCCGAGGAGCTCATCTCCGGCATCGCCAGGGGCTGCGAGCTCGACAACATCATGCGCCTCACCGGCATCACCGGCATCGTCAACGGCATGGACGTCAGCGAGTGGGACCCCAGCAGGGACAAGTACATCGCCGTGAAGTACGACGTGTCGACGGTGAGCTGGCTAGCTAGCTGATTCTGCTGCCTGGTCCTCCTGCTCATGCTGGTTCGGTTCTGACGCGGCAAGTGTACGTACGTGCGTGCGACGGTGGTGTGGTGTCCGGTTCAGGCCGTGGAGGCCAAGGCGCTGAACAAGGAGGCGCTGCAGGCGGAGGTCGGGCTCCCGGTGGACCGGAACATCCCGCTGGTGGCGTTCATCGGCAGGCTGGAAGAGCAGAAGGGACCCGACGTCATGGCGGCCGCCATCCCGCAGCTCATGGAGATGGTGGAGGACGTGCAGATCGTTCTGCTGGTACGTGTGCGCCGCCCGCCACCCGGCTACTACATGCGTGTATCGTTCGTTCTACTGGAACATGCGTGTGAGCAACGCGATGGATAATGCTGCAGGGCACGGGCAAGAAGAAGTTCGAGCGCATGCTCATGAGCGCCGAGGAGAAGTTCCCAGGCAAGGTGCGCGCCGTGGTCAAGTTCAACGCGGCGCTGGCGCACCACATCATGGCCGGCGCCGACGTGCTCGCCGTCACCAGCCGCTTCGAGCCCTGCGGCCTCATCCAGCTGCAGGGGATGCGATACGGAACGGTACGAGAGAGAAAAAAAAACATCCTGAATCCTGACGAGAGGGACAGAGACAGATTGATTATGAATGCTTCATCGATTTGAATTGATTGATCGATGTCTCCCGCTGCGACTCTTGCAGCCCTGCGCCTGCGCGTCCACCGGTGGACTCGTCGACACCATCATCGAAGGCAAGACCGGGTTCCACATGGGCCGCCTCAGCGTCGACGTAAGCCTACCTCTGCCATGTTCTTTCTTCTTTCTTTCTGTATGTATGTATGTATGTACGAATCAGCACCGCCATTCTTGTTTCGTCGTCCTCTCTTCCCAGTGCAACGTCGTGGAGCCGGCGGACGTCAAGAAGGTGGCCACCACCTTGCAGCGCGCCATCAAGGTGGTCGGCACGCCGGCGTACGAGGAGATGGTGAGGAACTGCATGATCCAGGATCTCTCCTGGAAGGTACGTACGCCCGCCCCGCCAGAGCAGAGCGCCAAGATCGATCGACCGACCGACCACACGTACGCGCCTCGCTCCTGTCGCTGACCGTGGTTTAATTTGCGAAATGCGCAGGGCCCTGCCAAGAACTGGGAGAACGTGCTGCTCAGCCTCGGGGTCGCTGGCGGCGAGCCAGGGGTCGAAGGCGAGGAGATCGCGCCGCTCGCCAAGGAGAACGTGGCCGCGCCCTGAAGAGTTCGGCCTGCAGGGCCCCTGATCTCGCGCGTGGTGCAAAGATGTTGGGACATCTTCTTATATATGCTGTTTCGTTTATGTGATATGGACAAGTATGTGTAGCTGCTTGCTTGTGCTAGTGTAATATAGTGTAGTGGTGGCCAGTGGCACAACCTAATAAGCGCATGAACTAATTGCTTGCGTGTGTAGTTAAGTACCGATCGGTAATTTTATAT |
| SWL107 | 97.97 | CCACAACTGTTCGCGTCCTGCTGGTTCATTATCTGACCTGATTGCATTATTGCAGCTACGAGAAGCCCGTGGAAGGCCGGAAGATCAACTGGATGAAGGCCGGGATCCTCGAGGCCGACAGGGTCCTCACCGTCAGCCCCTACTACGCCGAGGAGCTCATCTCCGGCATCGCCAGGGGCTGCGAGCTCGACAACATCATGCGCCTCACCGGCATCACCGGCATCGTCAACGGCATGGACGTCAGCGAGTGGGACCCCAGCAGGGACAAGTACATCGCCGTGAAGTACGACGTGTCGACGGTGAGCTGGCTAGCTAGCTGATTCTGCTGCCTGGTCCTCCTGCTCATGCTGGTTCGGTTCTGACGCGGCAAGTGTACGTACGTGCGTGCGACGGTGGTGTGGTGTCCGGTTCAGGCCGTGGAGGCCAAGGCGCTGAACAAGGAGGCGCTGCAGGCGGAGGTCGGGCTCCCGGTGGACCGGAACATCCCGCTGGTGGCGTTCATCGGCAGGCTGGAAGAGCAGAAGGGACCCGACGTCATGGCGGCCGCCATCCCGCAGCTCATGGAGATGGTGGAGGACGTGCAGATCGTTCTGCTGGTACGTGTGCGCCGCCCGCCACCCGGCTACTACATGCGTGTATCGTTCGTTCTACTGGAACATGCGTGTGAGCAACGCGATGGATAATGCTGCAGGGCACGGGCAAGAAGAAGTTCGAGCGCATGCTCATGAGCGCCGAGGAGAAGTTCCCAGGCAAGGTGCGCGCCGTGGCAAGTTCAACGCGGCGCTGGCGCACCACATCATGGCCGGCGCCGACGTGCTCGCCGTCACCAGCCGCTTCGAGCCCTGCGGCCTCATCCAGCTGCAGGGGATGCGATACGGAACGGTACGAGAGAGAAAAAAAAACATCCTGAATCCTGACGAGAGGGACAGAGACAGATTGATTATGAATGCTTCATCGATTTGAATTGATTGATCGATGTCTCCCGCTGCGACTCTTGCAGCCCTGCGCCTGCGCGTCCACCGGTGGACTCGTCGACACCATCATCGAAGGCAAGACCGGGTTCCACATGGGCCGCCTCAGCGTCGACGTAAGCCTACCTCTGCCATGTTCTTTCTTCTTTCTTTCTGTATGTATGTATGTATGTACGAATCAGCACCGCCATTCTTGTTTCGTCGTCCTCTCTTCCCAGTGCAACGTCGTGGAGCCGGCGGACGTCAAGAAGGTGGCCACCACCTTGCAGCGCGCCATCAAGGTGGTCGGCACGCCGGCGTACGAGGAGATGGTGAGGAACTGCATGATCCAGGATCTCTCCTGGAAGGTACGTACGCCCGCCCCGCCAGAGCAGAGCGCCAAGATCGATCGACCGACCGACCACACGTACGCGCCTCGCTCCTGTCGCTGACCGTGGTTTAATTTGCGAAATGCGCAGGGCCCTGCCAAGAACTGGGAGAACGTGCTGCTCAGCCTCGGGGTCGCTGGCGGCGAGCCAGGGGTCGAAGGCGAGGAGATCGCGCCGCTCGCCAAGGAGAACGTGGCCGCGCCCTGAAGAGTTCGGCCTGCAGGGCCCCTGATCTCGCGCGTGGTGCAAAGATGTTGGGACATCTTCTTATATATGCTGTTTCGTTTATGTGATATGGACAAGTATGTGTAGCTGCTTGCTTGTGCTAGTGTAATATAGTGTAGTGGTGGCCAGTGGCACAACCTAATAAGCGCATGAACTAATTGCTTGCGTGTGTAGTTAAGTACCGATCGGTAATTTTATAT |

**Table S1 Continued**

| **Accession** | **AC(%)** | **DNA Sequence** |
| --- | --- | --- |
| SWL108 | 98.15 | CCACAACTGTTCGCGTCCTGCTGGTTCATTATCTGACCTGATTGCATTATTGCAGCTACGAGAAGCCCGTGGAAGGCCGGAAGATCAACTGGATGAAGGCCGGGATCCTCGAGGCCGACAGGGTCCTCACCGTCAGCCCCTACTACGCCGAGGAGCTCATCTCCGGCATCGCCAGGGGCTGCGAGCTCGACAACATCATGCGCCTCACCGGCATCACCGGCATCGTCAACGGCATGGACGTCAGCGAGTGGGACCCCAGCAGGGACAAGTACATCGCCGTGAAGTACGACGTGTCGACGGTGAGCTGGCTAGCTAGCTGATTCTGCTGCCTGGTCCTCCTGCTCATGCTGGTTCGGTTCTGACGCGGCAAGTGTACGTACGTGCGTGCGACGGTGGTGTGGTGTCCGGTTCAGGCCGTGGAGGCCAAGGCGCTGAACAAGGAGGCGCTGCAGGCGGAGGTCGGGCTCCCGGTGGACCGGAACATCCCGCTGGTGGCGTTCATCGGCAGGCTGGAAGAGCAGAAGGGACCCGACGTCATGGCGGCCGCCATCCCGCAGCTCATGGAGATGGTGGAGGACGTGCAGATCGTTCTGCTGGTACGTGTGCGCCGCCCGCCACCCGGCTACTACATGCGTGTATCGTTCGTTCTACTGGAACATGCGTGTGAGCAACGCGATGGATAATGCTGCAGGGCACGGGCAAGAAGAAGTTCGAGCGCATGCTCATGAGCGCCGAGGAGAAGTTCCCAGGCAAGGTGCGCGCCGTGGTCAAGTTCAACGCGGCGCTGGCGCACCACATCATGGCCGGCGCCGACGTGCTCGCCGTCACCAGCCGCTTCGAGCCCTGCGGCCTCATCCAGCTGCAGGGGATGCGATACGGAACGGTACGAGAGAGAAAAAAAAACATCCTGAATCCTGACGAGAGGGACAGAGACAGATTGATTATGAATGCTTCATCGATTTGAATTGATTGATCGATGTCTCCCGCTGCGACTCTTGCAGCCCTGCGCCTGCGCGTCCACCGGTGGACTCGTCGACACCATCATCGAAGGCAAGACCGGGTTCCACATGGGCCGCCTCAGCGTCGACGTAAGCCTACCTCTGCCATGTTCTTTCTTCTTTCTTTCTGTATGTATGTATGTATGTACGAATCAGCACCGCCATTCTTGTTTCGTCGTCCTCTCTTCCCAGTGCAACGTCGTGGAGCCGGCGGACGTCAAGAAGGTGGCCACCACCTTGCAGCGCGCCATCAAGGTGGTCGGCACGCCGGCGTACGAGGAGATGGTGAGGAACTGCATGATCCAGGATCTCTCCTGGAAGGTACGTACGCCCGCCCCGCCAGAGCAGAGCGCCAAGATCGATCGACCGACCGACCACACGTACGCGCCTCGCTCCTGTCGCTGACCGTGGTTTAATTTGCGAAATGCGCAGGGCCCTGCCAAGAACTGGGAGAACGTGCTGCTCAGCCTCGGGGTCGCTGGCGGCGAGCCAGGGGTCGAAGGCGAGGAGATCGCGCCGCTCGCCAAGGAGAACGTGGCCGCGCCCTGAAGAGTTCGGCCTGCAGGGCCCCTGATCTCGCGCGTGGTGCAAAGATGTTGGGACATCTTCTTATATATGCTGTTTCGTTTATGTGATATGGACAAGTATGTGTAGCTGCTTGCTTGTGCTAGTGTAATATAGTGTAGTGGTGGCCAGTGGCACAACCTAATAAGCGCATGAACTAATTGCTTGCGTGTGTAGTTAAGTACCGATCGGTAATTTTATAT |
| SWL124 | 98.02 | CCACAACTGTTCGCGTCCTGCTGGTTCATTATCTGACCTGATTGCATTATTGCAGCTACGAGAAGCCCGTGGAAGGCCGGAAGATCAACTGGATGAAGGCCGGGATCCTCGAGGCCGACAGGGTCCTCACCGTCAGCCCCTACTACGCCGAGGAGCTCATCTCCGGCATCGCCAGGGGCTGCGAGCTCGACAACATCATGCGCCTCACCGGCATCACCGGCATCGTCAACGGCATGGACGTCAGCGAGTGGGACCCCAGCAGGGACAAGTACATCGCCGTGAAGTACGACGTGTCGACGGTGAGCTGGCTAGCTAGCTGATTCTGCTGCCTGGTCCTCCTGCTCATGCTGGTTCGGTTCTGACGCGGCAAGTGTACGTACGTGCGTGCGACGGTGGTGTGGTGTCCGGTTCAGGCCGTGGAGGCCAAGGCGCTGAACAAGGAGGCGCTGCAGGCGGAGGTCGGGCTCCCGGTGGACCGGAACATCCCGCTGGTGGCGTTCATCGGCAGGCTGGAAGAGCAGAAGGGACCCGACGTCATGGCGGCCGCCATCCCGCAGCTCATGGAGATGGTGGAGGACGTGCAGATCGTTCTGCTGGTACGTGTGCGCCGCCCGCCACCCGGCTACTACATGCGTGTATCGTTCGTTCTACTGGAACATGCGTGTGAGCAACGCGATGGATAATGCTGCAGGGCACGGGCAAGAAGAAGTTCGAGCGCATGCTCATGAGCGCCGAGGAGAAGTTCCCAGGCAAGGTGCGCGCCGTGGTCAAGTTCAACGCGGCGCTGGCGCACCACATCATGGCCGGCGCCGACGTGCTCGCCGTCACCAGCCGCTTCGAGCCCTGCGGCCTCATCCAGCTGCAGGGGATGCGATACGGAACGGTACGAGAGAGAAAAAAAAACATCCTGAATCCTGACGAGAGGGACAGAGACAGATTGATTATGAATGCTTCATCGATTTGAATTGATTGATCGATGTCTCCCGCTGCGACTCTTGCAGCCCTGCGCCTGCGCGTCCACCGGTGGACTCGTCGACACCATCATCGAAGGCAAGACCGGGTTCCACATGGGCCGCCTCAGCGTCGACGTAAGCCTACCTCTGCCATGTTCTTTCTTCTTTCTTTCTGTATGTATGTATGTATGTACGAATCAGCACCGCCATTCTTGTTTCGTCGTCCTCTCTTCCCAGTGCAACGTCGTGGAGCCGGCGGACGTCAAGAAGGTGGCCACCACCTTGCAGCGCGCCATCAAGGTGGTCGGCACGCCGGCGTACGAGGAGATGGTGAGGAACTGCATGATCCAGGATCTCTCCTGGAAGGTACGTACGCCCGCCCCGCCAGAGCAGAGCGCCAAGATCGATCGACCGACCGACCACACGTACGCGCCTCGCTCCTGTCGCTGACCGTGGTTTAATTTGCGAAATGCGCAGGGCCCTGCCAAGAACTGGGAGAACGTGCTGCTCAGCCTCGGGGTCGCTGGCGGCGAGCCAGGGGTCGAAGGCGAGGAGATCGCGCCGCTCGCCAAGGAGAACGTGGCCGCGCCCTGAAGAGTTCGGCCTGCAGGGCCCCTGATCTCGCGCGTGGTGCAAAGATGTTGGGACATCTTCTTATATATGCTGTTTCGTTTATGTGATATGGACAAGTATGTGTAGCTGCTTGCTTGTGCTAGTGTAATATAGTGTAGTGGTGGCCAGTGGCACAACCTAATAAGCGCATGAACTAATTGCTTGCGTGTGTAGTTAAGTACCGATCGGTAATTTTATAT |

**Table S1 Continued**

| **Accession** | **AC(%)** | **DNA Sequence** |
| --- | --- | --- |
| SWL125 | 97.23 | CCACAACTGTTCGCGTCCTGCTGGTTCATTATCTGACCTGATTGCATTATTGCAGCTACGAGAAGCCCGTGGAAGGCCGGAAGATCAACTGGATGAAGGCCGGGATCCTCGAGGCCGACAGGGTCCTCACCGTCAGCCCCTACTACGCCGAGGAGCTCATCTCCGGCATCGCCAGGGGCTGCGAGCTCGACAACATCATGCGCCTCACCGGCATCACCGGCATCGTCAACGGCATGGACGTCAGCGAGTGGGACCCCAGCAGGGACAAGTACATCGCCGTGAAGTACGACGTGTCGACGGTGAGCTGGCTAGCTAGCTGATTCTGCTGCCTGGTCCTCCTGCTCATGCTGGTTCGGTTCTGACGCGGCAAGTGTACGTACGTGCGTGCGACGGTGGTGTGGTGTCCGGTTCAGGCCGTGGAGGCCAAGGCGCTGAACAAGGAGGCGCTGCAGGCGGAGGTCGGGCTCCCGGTGGACCGGAACATCCCGCTGGTGGCGTTCATCGGCAGGCTGGAAGAGCAGAAGGGACCCGACGTCATGGCGGCCGCCATCCCGCAGCTCATGGAGATGGTGGAGGACGTGCAGATCGTTCTGCTGGTACGTGTGCGCCGCCCGCCACCCGGCTACTACATGCGTGTATCGTTCGTTCTACTGGAACATGCGTGTGAGCAACGCGATGGATAATGCTGCAGGGCACGGGCAAGAAGAAGTTCGAGCGCATGCTCATGAGCGCCGAGGAGAAGTTCCCAGGCAAGGTGCGCGCCGTGGTCAAGTTCAACGCGGCGCTGGCGCACCACATCATGGCCGGCGCCGACGTGCTCGCCGTCACCAGCCGCTTCGAGCCCTGCGGCCTCATCCAGCTGCAGGGGATGCGATACGGAACGGTACGAGAGAGAAAAAAAAACATCCTGAATCCTGACGAGAGGGACAGAGACAGATTGATTATGAATGCTTCATCGATTTGAATTGATTGATCGATGTCTCCCGCTGCGACTCTTGCAGCCCTGCGCCTGCGCGTCCACCGGTGGACTCGTCGACACCATCATCGAAGGCAAGACCGGGTTCCACATGGGCCGCCTCAGCGTCGACGTAAGCCTACCTCTGCCATGTTCTTTCTTCTTTCTTTCTGTATGTATGTATGTATGTACGAATCAGCACCGCCATTCTTGTTTCGTCGTCCTCTCTTCCCAGTGCAACGTCGTGGAGCCGGCGGACGTCAAGAAGGTGGCCACCACCTTGCAGCGCGCCATCAAGGTGGTCGGCACGCCGGCGTACGAGGAGATGGTGAGGAACTGCATGATCCAGGATCTCTCCTGGAAGGTACGTACGCCCGCCCCGCCAGAGCAGAGCGCCAAGATCGATCGACCGACCGACCACACGTACGCGCCTCGCTCCTGTCGCTGACCGTGGTTTAATTTGCGAAATGCGCAGGGCCCTGCCAAGAACTGGGAGAACGTGCTGCTCAGCCTCGGGGTCGCTGGCGGCGAGCCAGGGGTCGAAGGCGAGGAGATCGCGCCGCTCGCCAAGGAGAACGTGGCCGCGCCCTGAAGAGTTCGGCCTGCAGGGCCCCTGATCTCGCGCGTGGTGCAAAGATGTTGGGACATCTTCTTATATATGCTGTTTCGTTTATGTGATATGGACAAGTATGTGTAGCTGCTTGCTTGTGCTAGTGTAATATAGTGTAGTGGTGGCCAGTGGCACAACCTAATAAGCGCATGAACTAATTGCTTGCGTGTGTAGTTAAGTACCGATCGGTAATTTTATAT |
| SWL127 | 99.04 | CCACAACTGTTCGCGTCCTGCTGGTTCATTATCTGACCTGATTGCATTATTGCAGCTACGAGAAGCCCGTGGAAGGCCGGAAGATCAACTGGATGAAGGCCGGGATCCTCGAGGCCGACAGGGTCCTCACCGTCAGCCCCTACTACGCCGAGGAGCTCATCTCCGGCATCGCCAGGGGCTGCGAGCTCGACAACATCATGCGCCTCACCGGCATCACCGGCATCGTCAACGGCATGGACGTCAGCGAGTGGGACCCCAGCAGGGACAAGTACATCGCCGTGAAGTACGACGTGTCGACGGTGAGCTGGCTAGCTAGCTGATTCTGCTGCCTGGTCCTCCTGCTCATGCTGGTTCGGTTCTGACGCGGCAAGTGTACGTACGTGCGTGCGACGGTGGTGTGGTGTCCGGTTCAGGCCGTGGAGGCCAAGGCGCTGAACAAGGAGGCGCTGCAGGCGGAGGTCGGGCTCCCGGTGGACCGGAACATCCCGCTGGTGGCGTTCATCGGCAGGCTGGAAGAGCAGAAGGGACCCGACGTCATGGCGGCCGCCATCCCGCAGCTCATGGAGATGGTGGAGGACGTGCAGATCGTTCTGCTGGTACGTGTGCGCCGCCCGCCACCCGGCTACTACATGCGTGTATCGTTCGTTCTACTGGAACATGCGTGTGAGCAACGCGATGGATAATGCTGCAGGGCACGGGCAAGAAGAAGTTCGAGCGCATGCTCATGAGCGCCGAGGAGAAGTTCCCAGGCAAGGTGCGCGCCGTGGTCAAGTTCAACGCGGCGCTGGCGCACCACATCATGGCCGGCGCCGACGTGCTCGCCGTCACCAGCCGCTTCGAGCCCTGCGGCCTCATCCAGCTGCAGGGGATGCGATACGGAACGGTACGAGAGAGAAAAAAAAACATCCTGAATCCTGACGAGAGGGACAGAGACAGATTGATTATGAATGCTTCATCGATTTGAATTGATTGATCGATGTCTCCCGCTGCGACTCTTGCAGCCCTGCGCCTGCGCGTCCACCGGTGGACTCGTCGACACCATCATCGAAGGCAAGACCGGGTTCCACATGGGCCGCCTCAGCGTCGACGTAAGCCTACCTCTGCCATGTTCTTTCTTCTTTCTTTCTGTATGTATGTATGTATGTACGAATCAGCACCGCCATTCTTGTTTCGTCGTCCTCTCTTCCCAGTGCAACGTCGTGGAGCCGGCGGACGTCAAGAAGGTGGCCACCACCTTGCAGCGCGCCATCAAGGTGGTCGGCACGCCGGCGTACGAGGAGATGGTGAGGAACTGCATGATCCAGGATCTCTCCTGGAAGGTACGTACGCCCGCCCCGCCAGAGCAGAGCGCCAAGATCGATCGACCGACCGACCACACGTACGCGCCTCGCTCCTGTCGCTGACCGTGGTTTAATTTGCGAAATGCGCAGGGCCCTGCCAAGAACTGGGAGAACGTGCTGCTCAGCCTCGGGGTCGCTGGCGGCGAGCCAGGGGTCGAAGGCGAGGAGATCGCGCCGCTCGCCAAGGAGAACGTGGCCGCGCCCTGAAGAGTTCGGCCTGCAGGGCCCCTGATCTCGCGCGTGGTGCAAAGATGTTGGGACATCTTCTTATATATGCTGTTTCGTTTATGTGATATGGACAAGTATGTGTAGCTGCTTGCTTGTGCTAGTGTAATATAGTGTAGTGGTGGCCAGTGGCACAACCTAATAAGCGCATGAACTAATTGCTTGCGTGTGTAGTTAAGTACCGATCGGTAATTTTATAT |

**Table S1 Continued**

| **Accession** | **AC(%)** | **DNA Sequence** |
| --- | --- | --- |
| SWL129 | 97.91 | CCACAACTGTTCGCGTCCTGCTGGTTCATTATCTGACCTGATTGCATTATTGCAGCTACGAGAAGCCCGTGGAAGGCCGGAAGATCAACTGGATGAAGGCCGGGATCCTCGAGGCCGACAGGGTCCTCACCGTCAGCCCCTACTACGCCGAGGAGCTCATCTCCGGCATCGCCAGGGGCTGCGAGCTCGACAACATCATGCGCCTCACCGGCATCACCGGCATCGTCAACGGCATGGACGTCAGCGAGTGGGACCCCAGCAGGGACAAGTACATCGCCGTGAAGTACGACGTGTCGACGGTGAGCTGGCTAGCTAGCTGATTCTGCTGCCTGGTCCTCCTGCTCATGCTGGTTCGGTTCTGACGCGGCAAGTGTACGTACGTGCGTGCGACGGTGGTGTGGTGTCCGGTTCAGGCCGTGGAGGCCAAGGCGCTGAACAAGGAGGCGCTGCAGGCGGAGGTCGGGCTCCCGGTGGACCGGAACATCCCGCTGGTGGCGTTCATCGGCAGGCTGGAAGAGCAGAAGGGACCCGACGTCATGGCGGCCGCCATCCCGCAGCTCATGGAGATGGTGGAGGACGTGCAGATCGTTCTGCTGGTACGTGTGCGCCGCCCGCCACCCGGCTACTACATGCGTGTATCGTTCGTTCTACTGGAACATGCGTGTGAGCAACGCGATGGATAATGCTGCAGGGCACGGGCAAGAAGAAGTTCGAGCGCATGCTCATGAGCGCCGAGGAGAAGTTCCCAGGCAAGGTGCGCGCCGTGGTCAAGTTCAACGCGGCGCTGGCGCACCACATCATGGCCGGCGCCGACGTGCTCGCCGTCACCAGCCGCTTCGAGCCCTGCGGCCTCATCCAGCTGCAGGGGATGCGATACGGAACGGTACGAGAGAGAAAAAAAAACATCCTGAATCCTGACGAGAGGGACAGAGACAGATTGATTATGAATGCTTCATCGATTTGAATTGATTGATCGATGTCTCCCGCTGCGACTCTTGCAGCCCTGCGCCTGCGCGTCCACCGGTGGACTCGTCGACACCATCATCGAAGGCAAGACCGGGTTCCACATGGGCCGCCTCAGCGTCGACGTAAGCCTACCTCTGCCATGTTCTTTCTTCTTTCTTTCTGTATGTATGTATGTATGTACGAATCAGCACCGCCATTCTTGTTTCGTCGTCCTCTCTTCCCAGTGCAACGTCGTGGAGCCGGCGGACGTCAAGAAGGTGGCCACCACCTTGCAGCGCGCCATCAAGGTGGTCGGCACGCCGGCGTACGAGGAGATGGTGAGGAACTGCATGATCCAGGATCTCTCCTGGAAGGTACGTACGCCCGCCCCGCCAGAGCAGAGCGCCAAGATCGATCGACCGACCGACCACACGTACGCGCCTCGCTCCTGTCGCTGACCGTGGTTTAATTTGCGAAATGCGCAGGGCCCTGCCAAGAACTGGGAGAACGTGCTGCTCAGCCTCGGGGTCGCTGGCGGCGAGCCAGGGGTCGAAGGCGAGGAGATCGCGCCGCTCGCCAAGGAGAACGTGGCCGCGCCCTGAAGAGTTCGGCCTGCAGGGCCCCTGATCTCGCGCGTGGTGCAAAGATGTTGGGACATCTTCTTATATATGCTGTTTCGTTTATGTGATATGGACAAGTATGTGTAGCTGCTTGCTTGTGCTAGTGTAATATAGTGTAGTGGTGGCCAGTGGCACAACCTAATAAGCGCATGAACTAATTGCTTGCGTGTGTAGTTAAGTACCGATCGGTAATTTTATAT |
| SWL130 | 98.55 | CCACAACTGTTCGCGTCCTGCTGGTTCATTATCTGACCTGATTGCATTATTGCAGCTACGAGAAGCCCGTGGAAGGCCGGAAGATCAACTGGATGAAGGCCGGGATCCTCGAGGCCGACAGGGTCCTCACCGTCAGCCCCTACTACGCCGAGGAGCTCATCTCCGGCATCGCCAGGGGCTGCGAGCTCGACAACATCATGCGCCTCACCGGCATCACCGGCATCGTCAACGGCATGGACGTCAGCGAGTGGGACCCCAGCAGGGACAAGTACATCGCCGTGAAGTACGACGTGTCGACGGTGAGCTGGCTAGCTAGCTGATTCTGCTGCCTGGTCCTCCTGCTCATGCTGGTTCGGTTCTGACGCGGCAAGTGTACGTACGTGCGTGCGACGGTGGTGTGGTGTCCGGTTCAGGCCGTGGAGGCCAAGGCGCTGAACAAGGAGGCGCTGCAGGCGGAGGTCGGGCTCCCGGTGGACCGGAACATCCCGCTGGTGGCGTTCATCGGCAGGCTGGAAGAGCAGAAGGGACCCGACGTCATGGCGGCCGCCATCCCGCAGCTCATGGAGATGGTGGAGGACGTGCAGATCGTTCTGCTGGTACGTGTGCGCCGCCCGCCACCCGGCTACTACATGCGTGTATCGTTCGTTCTACTGGAACATGCGTGTGAGCAACGCGATGGATAATGCTGCAGGGCACGGGCAAGAAGAAGTTCGAGCGCATGCTCATGAGCGCCGAGGAGAAGTTCCCAGGCAAGGTGCGCGCCGTGGTCAAGTTCAACGCGGCGCTGGCGCACCACATCATGGCCGGCGCCGACGTGCTCGCCGTCACCAGCCGCTTCGAGCCCTGCGGCCTCATCCAGCTGCAGGGGATGCGATACGGAACGGTACGAGAGAGAAAAAAAAACATCCTGAATCCTGACGAGAGGGACAGAGACAGATTGATTATGAATGCTTCATCGATTTGAATTGATTGATCGATGTCTCCCGCTGCGACTCTTGCAGCCCTGCGCCTGCGCGTCCACCGGTGGACTCGTCGACACCATCATCGAAGGCAAGACCGGGTTCCACATGGGCCGCCTCAGCGTCGACGTAAGCCTACCTCTGCCATGTTCTTTCTTCTTTCTTTCTGTATGTATGTATGTATGTACGAATCAGCACCGCCATTCTTGTTTCGTCGTCCTCTCTTCCCAGTGCAACGTCGTGGAGCCGGCGGACGTCAAGAAGGTGGCCACCACCTTGCAGCGCGCCATCAAGGTGGTCGGCACGCCGGCGTACGAGGAGATGGTGAGGAACTGCATGATCCAGGATCTCTCCTGGAAGGTACGTACGCCCGCCCCGCCAGAGCAGAGCGCCAAGATCGATCGACCGACCGACCACACGTACGCGCCTCGCTCCTGTCGCTGACCGTGGTTTAATTTGCGAAATGCGCAGGGCCCTGCCAAGAACTGGGAGAACGTGCTGCTCAGCCTCGGGGTCGCTGGCGGCGAGCCAGGGGTCGAAGGCGAGGAGATCGCGCCGCTCGCCAAGGAGAACGTGGCCGCGCCCTGAAGAGTTCGGCCTGCAGGGCCCCTGATCTCGCGCGTGGTGCAAAGATGTTGGGACATCTTCTTATATATGCTGTTTCGTTTATGTGATATGGACAAGTATGTGTAGCTGCTTGCTTGTGCTAGTGTAATATAGTGTAGTGGTGGCCAGTGGCACAACCTAATAAGCGCATGAACTAATTGCTTGCGTGTGTAGTTAAGTACCGATCGGTAATTTTATAT |

**Table S1 Continued**

| **Accession** | **AC(%)** | **DNA Sequence** |
| --- | --- | --- |
| SWL131 | 99.84 | CCACAACTGTTCGCGTCCTGCTGGTTCATTATCTGACCTGATTGCATTATTGCAGCTACGAGAAGCCCGTGGAAGGCCGGAAGATCAACTGGATGAAGGCCGGGATCCTCGAGGCCGACAGGGTCCTCACCGTCAGCCCCTACTACGCCGAGGAGCTCATCTCCGGCATCGCCAGGGGCTGCGAGCTCGACAACATCATGCGCCTCACCGGCATCACCGGCATCGTCAACGGCATGGACGTCAGCGAGTGGGACCCCAGCAGGGACAAGTACATCGCCGTGAAGTACGACGTGTCGACGGTGAGCTGGCTAGCTAGCTGATTCTGCTGCCTGGTCCTCCTGCTCATGCTGGTTCGGTTCTGACGCGGCAAGTGTACGTACGTGCGTGCGACGGTGGTGTGGTGTCCGGTTCAGGCCGTGGAGGCCAAGGCGCTGAACAAGGAGGCGCTGCAGGCGGAGGTCGGGCTCCCGGTGGACCGGAACATCCCGCTGGTGGCGTTCATCGGCAGGCTGGAAGAGCAGAAGGGACCCGACGTCATGGCGGCCGCCATCCCGCAGCTCATGGAGATGGTGGAGGACGTGCAGATCGTTCTGCTGGTACGTGTGCGCCGCCCGCCACCCGGCTACTACATGCGTGTATCGTTCGTTCTACTGGAACATGCGTGTGAGCAACGCGATGGATAATGCTGCAGGGCACGGGCAAGAAGAAGTTCGAGCGCATGCTCATGAGCGCCGAGGAGAAGTTCCCAGGCAAGGTGCGCGCCGTGGTCAAGTTCAACGCGGCGCTGGCGCACCACATCATGGCCGGCGCCGACGTGCTCGCCGTCACCAGCCGCTTCGAGCCCTGCGGCCTCATCCAGCTGCAGGGGATGCGATACGGAACGGTACGAGAGAGAAAAAAAAACATCCTGAATCCTGACGAGAGGGACAGAGACAGATTGATTATGAATGCTTCATCGATTTGAATTGATTGATCGATGTCTCCCGCTGCGACTCTTGCAGCCCTGCGCCTGCGCGTCCACCGGTGGACTCGTCGACACCATCATCGAAGGCAAGACCGGGTTCCACATGGGCCGCCTCAGCGTCGACGTAAGCCTACCTCTGCCATGTTCTTTCTTCTTTCTTTCTGTATGTATGTATGTATGTACGAATCAGCACCGCCATTCTTGTTTCGTCGTCCTCTCTTCCCAGTGCAACGTCGTGGAGCCGGCGGACGTCAAGAAGGTGGCCACCACCTTGCAGCGCGCCATCAAGGTGGTCGGCACGCCGGCGTACGAGGAGATGGTGAGGAACTGCATGATCCAGGATCTCTCCTGGAAGGTACGTACGCCCGCCCCGCCAGAGCAGAGCGCCAAGATCGATCGACCGACCGACCACACGTACGCGCCTCGCTCCTGTCGCTGACCGTGGTTTAATTTGCGAAATGCGCAGGGCCCTGCCAAGAACTGGGAGAACGTGCTGCTCAGCCTCGGGGTCGCTGGCGGCGAGCCAGGGGTCGAAGGCGAGGAGATCGCGCCGCTCGCCAAGGAGAACGTGGCCGCGCCCTGAAGAGTTCGGCCTGCAGGGCCCCTGATCTCGCGCGTGGTGCAAAGATGTTGGGACATCTTCTTATATATGCTGTTTCGTTTATGTGATATGGACAAGTATGTGTAGCTGCTTGCTTGTGCTAGTGTAATATAGTGTAGTGGTGGCCAGTGGCACAACCTAATAAGCGCATGAACTAATTGCTTGCGTGTGTAGTTAAGTACCGATCGGTAATTTTATAT |
| SWL132 | 98.38 | CCACAACTGTTCGCGTCCTGCTGGTTCATTATCTGACCTGATTGCATTATTGCAGCTACGAGAAGCCCGTGGAAGGCCGGAAGATCAACTGGATGAAGGCCGGGATCCTCGAGGCCGACAGGGTCCTCACCGTCAGCCCCTACTACGCCGAGGAGCTCATCTCCGGCATCGCCAGGGGCTGCGAGCTCGACAACATCATGCGCCTCACCGGCATCACCGGCATCGTCAACGGCATGGACGTCAGCGAGTGGGACCCCAGCAGGGACAAGTACATCGCCGTGAAGTACGACGTGTCGACGGTGAGCTGGCTAGCTAGCTGATTCTGCTGCCTGGTCCTCCTGCTCATGCTGGTTCGGTTCTGACGCGGCAAGTGTACGTACGTGCGTGCGACGGTGGTGTGGTGTCCGGTTCAGGCCGTGGAGGCCAAGGCGCTGAACAAGGAGGCGCTGCAGGCGGAGGTCGGGCTCCCGGTGGACCGGAACATCCCGCTGGTGGCGTTCATCGGCAGGCTGGAAGAGCAGAAGGGACCCGACGTCATGGCGGCCGCCATCCCGCAGCTCATGGAGATGGTGGAGGACGTGCAGATCGTTCTGCTGGTACGTGTGCGCCGCCCGCCACCCGGCTACTACATGCGTGTATCGTTCGTTCTACTGGAACATGCGTGTGAGCAACGCGATGGATAATGCTGCAGGGCACGGGCAAGAAGAAGTTCGAGCGCATGCTCATGAGCGCCGAGGAGAAGTTCCCAGGCAAGGTGCGCGCCGTGGTCAAGTTCAACGCGGCGCTGGCGCACCACATCATGGCCGGCGCCGACGTGCTCGCCGTCACCAGCCGCTTCGAGCCCTGCGGCCTCATCCAGCTGCAGGGGATGCGATACGGAACGGTACGAGAGAGAAAAAAAAACATCCTGAATCCTGACGAGAGGGACAGAGACAGATTGATTATGAATGCTTCATCGATTTGAATTGATTGATCGATGTCTCCCGCTGCGACTCTTGCAGCCCTGCGCCTGCGCGTCCACCGGTGGACTCGTCGACACCATCATCGAAGGCAAGACCGGGTTCCACATGGGCCGCCTCAGCGTCGACGTAAGCCTACCTCTGCCATGTTCTTTCTTCTTTCTTTCTGTATGTATGTATGTATGTACGAATCAGCACCGCCATTCTTGTTTCGTCGTCCTCTCTTCCCAGTGCAACGTCGTGGAGCCGGCGGACGTCAAGAAGGTGGCCACCACCTTGCAGCGCGCCATCAAGGTGGTCGGCACGCCGGCGTACGAGGAGATGGTGAGGAACTGCATGATCCAGGATCTCTCCTGGAAGGTACGTACGCCCGCCCCGCCAGAGCAGAGCGCCAAGATCGATCGACCGACCGACCACACGTACGCGCCTCGCTCCTGTCGCTGACCGTGGTTTAATTTGCGAAATGCGCAGGGCCCTGCCAAGAACTGGGAGAACGTGCTGCTCAGCCTCGGGGTCGCTGGCGGCGAGCCAGGGGTCGAAGGCGAGGAGATCGCGCCGCTCGCCAAGGAGAACGTGGCCGCGCCCTGAAGAGTTCGGCCTGCAGGGCCCCTGATCTCGCGCGTGGTGCAAAGATGTTGGGACATCTTCTTATATATGCTGTTTCGTTTATGTGATATGGACAAGTATGTGTAGCTGCTTGCTTGTGCTAGTGTAATATAGTGTAGTGGTGGCCAGTGGCACAACCTAATAAGCGCATGAACTAATTGCTTGCGTGTGTAGTTAAGTACCGATCGGTAATTTTATAT |

**Table S1 Continued**

| **Accession** | **AC(%)** | **DNA Sequence** |
| --- | --- | --- |
| SWL135 | 98.43 | CCACAACTGTTCGCGTCCTGCTGGTTCATTATCTGACCTGATTGCATTATTGCAGCTACGAGAAGCCCGTGGAAGGCCGGAAGATCAACTGGATGAAGGCCGGGATCCTCGAGGCCGACAGGGTCCTCACCGTCAGCCCCTACTACGCCGAGGAGCTCATCTCCGGCATCGCCAGGGGCTGCGAGCTCGACAACATCATGCGCCTCACCGGCATCACCGGCATCGTCAACGGCATGGACGTCAGCGAGTGGGACCCCAGCAGGGACAAGTACATCGCCGTGAAGTACGACGTGTCGACGGTGAGCTGGCTAGCTAGCTGATTCTGCTGCCTGGTCCTCCTGCTCATGCTGGTTCGGTTCTGACGCGGCAAGTGTACGTACGTGCGTGCGACGGTGGTGTGGTGTCCGGTTCAGGCCGTGGAGGCCAAGGCGCTGAACAAGGAGGCGCTGCAGGCGGAGGTCGGGCTCCCGGTGGACCGGAACATCCCGCTGGTGGCGTTCATCGGCAGGCTGGAAGAGCAGAAGGGACCCGACGTCATGGCGGCCGCCATCCCGCAGCTCATGGAGATGGTGGAGGACGTGCAGATCGTTCTGCTGGTACGTGTGCGCCGCCCGCCACCCGGCTACTACATGCGTGTATCGTTCGTTCTACTGGAACATGCGTGTGAGCAACGCGATGGATAATGCTGCAGGGCACGGGCAAGAAGAAGTTCGAGCGCATGCTCATGAGCGCCGAGGAGAAGTTCCCAGGCAAGGTGCGCGCCGTGGTCAAGTTCAACGCGGCGCTGGCGCACCACATCATGGCCGGCGCCGACGTGCTCGCCGTCACCAGCCGCTTCGAGCCCTGCGGCCTCATCCAGCTGCAGGGGATGCGATACGGAACGGTACGAGAGAGAAAAAAAAACATCCTGAATCCTGACGAGAGGGACAGAGACAGATTGATTATGAATGCTTCATCGATTTGAATTGATTGATCGATGTCTCCCGCTGCGACTCTTGCAGCCCTGCGCCTGCGCGTCCACCGGTGGACTCGTCGACACCATCATCGAAGGCAAGACCGGGTTCCACATGGGCCGCCTCAGCGTCGACGTAAGCCTACCTCTGCCATGTTCTTTCTTCTTTCTTTCTGTATGTATGTATGTATGTACGAATCAGCACCGCCATTCTTGTTTCGTCGTCCTCTCTTCCCAGTGCAACGTCGTGGAGCCGGCGGACGTCAAGAAGGTGGCCACCACCTTGCAGCGCGCCATCAAGGTGGTCGGCACGCCGGCGTACGAGGAGATGGTGAGGAACTGCATGATCCAGGATCTCTCCTGGAAGGTACGTACGCCCGCCCCGCCAGAGCAGAGCGCCAAGATCGATCGACCGACCGACCACACGTACGCGCCTCGCTCCTGTCGCTGACCGTGGTTTAATTTGCGAAATGCGCAGGGCCCTGCCAAGAACTGGGAGAACGTGCTGCTCAGCCTCGGGGTCGCTGGCGGCGAGCCAGGGGTCGAAGGCGAGGAGATCGCGCCGCTCGCCAAGGAGAACGTGGCCGCGCCCTGAAGAGTTCGGCCTGCAGGGCCCCTGATCTCGCGCGTGGTGCAAAGATGTTGGGACATCTTCTTATATATGCTGTTTCGTTTATGTGATATGGACAAGTATGTGTAGCTGCTTGCTTGTGCTAGTGTAATATAGTGTAGTGGTGGCCAGTGGCACAACCTAATAAGCGCATGAACTAATTGCTTGCGTGTGTAGTTAAGTACCGATCGGTAATTTTATAT |
| SWL138 | 98.86 | CCACAACTGCTCGCGTCCTGCTGGTTCATTATCTGACCTTGATTGCATTGCAGCTACGAGAAGCCCGTGGAAGGCCGGAAGATCAACTGGATGAAGGCCGGGATCCTCGAGGCCGACAGGGTCCTCACCGTCAGCCCCTACTACGCCGAGGAGCTCATCTCCGGCATCGCCAGGGGCTGCGAGCTCGACAACATCATGCGCCTCACCGGCATCACCGGCATCGTCAACGGCATGGACGTCAGCGAGTGGGACCCCAGCAGGGACAAGTACATCGCCGTGAAGTACGACGTGTCGACGGTGAGCTGGCTAGCTCTGATTCTGCTGCCTGGTCCTCCTGCTCATCATGCTGGTTCGGTACTGACGCGGCAAGTGTACGTACGTGCGTGCGACGGTGGTGTCCGGTTCAGGCCGTGGAGGCCAAGGCGCTGAACAAGGAGGCGCTGCAGGCGGAGGTCGGGCTCCCGGTGGACCGGAACATCCCGCTGGTGGCGTTCATCGGCAGGCTGGAAGAGCAGAAGGGCCCCGACGTCATGGCGGCCGCCATCCCGCAGCTCATGGAGATGGTGGAGGACGTGCAGATCGTTCTGCTGGTACGTGCGCCGCCCGCCACCCGGCTACTACATGCGTGTATAATCGTTCTACTGGAACATACGTGTGAGCAACGCGATGGATAATGCTGCAGGGCACGGGCAAGAAGAAGTTCGAGCGCATGCTCATGAGCGCCGAGGAGAAGTTCCCAGGCAAGGTGCGCGCCGTGGTCAAGTTCAACGCGGCGCTGGCGCACCACATCATGGCCGGCGCCGACGTGCTCGCCGTCACCAGCCGCTTCGAGCCCTGCGGCCTCATCCAGCTGCAGGGGATGCGATACGGAACGGTACGAGAGAGAAAAAAAAACATCCTGAATCCTGACGAGAGGGACAGAGACAGATTGATTATGAATGCTTCATCGATTTGAATTGATTGATCGATGTCTCCCGCTGCGACTCTTGCAGCCCTGCGCCTGCGCGTCCACCGGTGGACTCGTCGACACCATCATCGAAGGCAAGACCGGGTTCCACATGGGCCGCCTCAGCGTCGACGTAAGCCTAGCTCTGCCATGATCTTTCTTCTTTCTGTATGTATGTATGTATGAATCAGCACCGCCATTCTTGTTTCGTCGTCCTCTCTTCCCAGTGCAACGTCGTGGAGCCGGCGGACGTCAAGAAGGTGGCCACCACCTTGCAGCGCGCCATCAAGGTGGTCGGCACGCCGGCGTACGAGGAGATGGTGAGGAACTGCATGATCCAGGATCTCTCCTGGAAGGTACGTACGCCCGCCCCGCCAGAGCAGAGCGCCAAGCCGCCAAGATTGATCGATCGACCGACCACACGTACGCGCCTCGCTCCTGTCGCTGACCGTGGTTTAATTTGCGAAATGCGCAGGGCCCTGCCAAGAACTGGGAGAACGTGCTGCTCAGCCTCGGGGTCGCCGGCGGCGAGCCAGGGGTCGAAGGCGAGGAGATCGCGCCGCTCGCCAAGGAGAACGTGGCCGCGCCCTGAAGAGTTCGGCCTGCAGGGCCCCTGATCTCGCGCGTGGTGCAAAGATGTTGGGACATCTTCTTATATATGCTGTTTCGTTTATGTGATATGGACAAGTATGTGTAGCTGCTTGCTTGTGCTAGTGTAATATAGTGTAGTGGTGGCCAGTGGCACAACCTAATAAGCGCATGAACTAATTGCTTGCGTGTGTAGTTAAGTACCGATCGGTAATTTTATAT |

**Table S1 Continued**

| **Accession** | **AC(%)** | **DNA Sequence** |
| --- | --- | --- |
| SWL155 | 97.04 | CCACAACTGTTCGCGTCCTGCTGGTTCATTATCTGACCTGATTGCATTATTGCAGCTACGAGAAGCCCGTGGAAGGCCGGAAGATCAACTGGATGAAGGCCGGGATCCTCGAGGCCGACAGGGTCCTCACCGTCAGCCCCTACTACGCCGAGGAGCTCATCTCCGGCATCGCCAGGGGCTGCGAGCTCGACAACATCATGCGCCTCACCGGCATCACCGGCATCGTCAACGGCATGGACGTCAGCGAGTGGGACCCCAGCAGGGACAAGTACATCGCCGTGAAGTACGACGTGTCGACGGTGAGCTGGCTAGCTAGCTGATTCTGCTGCCTGGTCCTCCTGCTCATGCTGGTTCGGTTCTGACGCGGCAAGTGTACGTACGTGCGTGCGACGGTGGTGTGGTGTCCGGTTCAGGCCGTGGAGGCCAAGGCGCTGAACAAGGAGGCGCTGCAGGCGGAGGTCGGGCTCCCGGTGGACCGGAACATCCCGCTGGTGGCGTTCATCGGCAGGCTGGAAGAGCAGAAGGGACCCGACGTCATGGCGGCCGCCATCCCGCAGCTCATGGAGATGGTGGAGGACGTGCAGATCGTTCTGCTGGTACGTGTGCGCCGCCCGCCACCCGGCTACTACATGCGTGTATCGTTCGTTCTACTGGAACATGCGTGTGAGCAACGCGATGGATAATGCTGCAGGGCACGGGCAAGAAGAAGTTCGAGCGCATGCTCATGAGCGCCGAGGAGAAGTTCCCAGGCAAGGTGCGCGCCGTGGTCAAGTTCAACGCGGCGCTGGCGCACCACATCATGGCCGGCGCCGACGTGCTCGCCGTCACCAGCCGCTTCGAGCCCTGCGGCCTCATCCAGCTGCAGGGGATGCGATACGGAACGGTACGAGAGAGAAAAAAAAACATCCTGAATCCTGACGAGAGGGACAGAGACAGATTGATTATGAATGCTTCATCGATTTGAATTGATTGATCGATGTCTCCCGCTGCGACTCTTGCAGCCCTGCGCCTGCGCGTCCACCGGTGGACTCGTCGACACCATCATCGAAGGCAAGACCGGGTTCCACATGGGCCGCCTCAGCGTCGACGTAAGCCTACCTCTGCCATGTTCTTTCTTCTTTCTTTCTGTATGTATGTATGTATGTACGAATCAGCACCGCCATTCTTGTTTCGTCGTCCTCTCTTCCCAGTGCAACGTCGTGGAGCCGGCGGACGTCAAGAAGGTGGCCACCACCTTGCAGCGCGCCATCAAGGTGGTCGGCACGCCGGCGTACGAGGAGATGGTGAGGAACTGCATGATCCAGGATCTCTCCTGGAAGGTACGTACGCCCGCCCCGCCAGAGCAGAGCGCCAAGATCGATCGACCGACCGACCACACGTACGCGCCTCGCTCCTGTCGCTGACCGTGGTTTAATTTGCGAAATGCGCAGGGCCCTGCCAAGAACTGGGAGAACGTGCTGCTCAGCCTCGGGGTCGCTGGCGGCGAGCCAGGGGTCGAAGGCGAGGAGATCGCGCCGCTCGCCAAGGAGAACGTGGCCGCGCCCTGAAGAGTTCGGCCTGCAGGGCCCCTGATCTCGCGCGTGGTGCAAAGATGTTGGGACATCTTCTTATATATGCTGTTTCGTTTATGTGATATGGACAAGTATGTGTAGCTGCTTGCTTGTGCTAGTGTAATATAGTGTAGTGGTGGCCAGTGGCACAACCTAATAAGCGCATGAACTAATTGCTTGCGTGTGTAGTTAAGTACCGATCGGTAATTTTATAT |
| SWL161 | 99.11 | CCACAACTGTTCGCGTCCTGCTGGTTCATTATCTGACCTGATTGCATTATTGCAGCTACGAGAAGCCCGTGGAAGGCCGGAAGATCAACTGGATGAAGGCCGGGATCCTCGAGGCCGACAGGGTCCTCACCGTCAGCCCCTACTACGCCGAGGAGCTCATCTCCGGCATCGCCAGGGGCTGCGAGCTCGACAACATCATGCGCCTCACCGGCATCACCGGCATCGTCAACGGCATGGACGTCAGCGAGTGGGACCCCAGCAGGGACAAGTACATCGCCGTGAAGTACGACGTGTCGACGGTGAGCTGGCTAGCTAGCTGATTCTGCTGCCTGGTCCTCCTGCTCATGCTGGTTCGGTTCTGACGCGGCAAGTGTACGTACGTGCGTGCGACGGTGGTGTGGTGTCCGGTTCAGGCCGTGGAGGCCAAGGCGCTGAACAAGGAGGCGCTGCAGGCGGAGGTCGGGCTCCCGGTGGACCGGAACATCCCGCTGGTGGCGTTCATCGGCAGGCTGGAAGAGCAGAAGGGACCCGACGTCATGGCGGCCGCCATCCCGCAGCTCATGGAGATGGTGGAGGACGTGCAGATCGTTCTGCTGGTACGTGTGCGCCGCCCGCCACCCGGCTACTACATGCGTGTATCGTTCGTTCTACTGGAACATGCGTGTGAGCAACGCGATGGATAATGCTGCAGGGCACGGGCAAGAAGAAGTTCGAGCGCATGCTCATGAGCGCCGAGGAGAAGTTCCCAGGCAAGGTGCGCGCCGTGGTCAAGTTCAACGCGGCGCTGGCGCACCACATCATGGCCGGCGCCGACGTGCTCGCCGTCACCAGCCGCTTCGAGCCCTGCGGCCTCATCCAGCTGCAGGGGATGCGATACGGAACGGTACGAGAGAGAAAAAAAAACATCCTGAATCCTGACGAGAGGGACAGAGACAGATTGATTATGAATGCTTCATCGATTTGAATTGATTGATCGATGTCTCCCGCTGCGACTCTTGCAGCCCTGCGCCTGCGCGTCCACCGGTGGACTCGTCGACACCATCATCGAAGGCAAGACCGGGTTCCACATGGGCCGCCTCAGCGTCGACGTAAGCCTACCTCTGCCATGTTCTTTCTTCTTTCTTTCTGTATGTATGTATGTATGTACGAATCAGCACCGCCATTCTTGTTTCGTCGTCCTCTCTTCCCAGTGCAACGTCGTGGAGCCGGCGGACGTCAAGAAGGTGGCCACCACCTTGCAGCGCGCCATCAAGGTGGTCGGCACGCCGGCGTACGAGGAGATGGTGAGGAACTGCATGATCCAGGATCTCTCCTGGAAGGTACGTACGCCCGCCCCGCCAGAGCAGAGCGCCAAGATCGATCGACCGACCGACCACACGTACGCGCCTCGCTCCTGTCGCTGACCGTGGTTTAATTTGCGAAATGCGCAGGGCCCTGCCAAGAACTGGGAGAACGTGCTGCTCAGCCTCGGGGTCGCTGGCGGCGAGCCAGGGGTCGAAGGCGAGGAGATCGCGCCGCTCGCCAAGGAGAACGTGGCCGCGCCCTGAAGAGTTCGGCCTGCAGGGCCCCTGATCTCGCGCGTGGTGCAAAGATGTTGGGACATCTTCTTATATATGCTGTTTCGTTTATGTGATATGGACAAGTATGTGTAGCTGCTTGCTTGTGCTAGTGTAATATAGTGTAGTGGTGGCCAGTGGCACAACCTAATAAGCGCATGAACTAATTGCTTGCGTGTGTAGTTAAGTACCGATCGGTAATTTTATAT |

**Table S1 Continued**

| **Accession** | **AC(%)** | **DNA Sequence** |
| --- | --- | --- |
| SWL162 | 98.66 | CCACAACTGTTCGCGTCCTGCTGGTTCATTATCTGACCTGATTGCATTATTGCAGCTACGAGAAGCCCGTGGAAGGCCGGAAGATCAACTGGATGAAGGCCGGGATCCTCGAGGCCGACAGGGTCCTCACCGTCAGCCCCTACTACGCCGAGGAGCTCATCTCCGGCATCGCCAGGGGCTGCGAGCTCGACAACATCATGCGCCTCACCGGCATCACCGGCATCGTCAACGGCATGGACGTCAGCGAGTGGGACCCCAGCAGGGACAAGTACATCGCCGTGAAGTACGACGTGTCGACGGTGAGCTGGCTAGCTAGCTGATTCTGCTGCCTGGTCCTCCTGCTCATGCTGGTTCGGTTCTGACGCGGCAAGTGTACGTACGTGCGTGCGACGGTGGTGTGGTGTCCGGTTCAGGCCGTGGAGGCCAAGGCGCTGAACAAGGAGGCGCTGCAGGCGGAGGTCGGGCTCCCGGTGGACCGGAACATCCCGCTGGTGGCGTTCATCGGCAGGCTGGAAGAGCAGAAGGGACCCGACGTCATGGCGGCCGCCATCCCGCAGCTCATGGAGATGGTGGAGGACGTGCAGATCGTTCTGCTGGTACGTGTGCGCCGCCCGCCACCCGGCTACTACATGCGTGTATCGTTCGTTCTACTGGAACATGCGTGTGAGCAACGCGATGGATAATGCTGCAGGGCACGGGCAAGAAGAAGTTCGAGCGCATGCTCATGAGCGCCGAGGAGAAGTTCCCAGGCAAGGTGCGCGCCGTGGTCAAGTTCAACGCGGCGCTGGCGCACCACATCATGGCCGGCGCCGACGTGCTCGCCGTCACCAGCCGCTTCGAGCCCTGCGGCCTCATCCAGCTGCAGGGGATGCGATACGGAACGGTACGAGAGAGAAAAAAAAACATCCTGAATCCTGACGAGAGGGACAGAGACAGATTGATTATGAATGCTTCATCGATTTGAATTGATTGATCGATGTCTCCCGCTGCGACTCTTGCAGCCCTGCGCCTGCGCGTCCACCGGTGGACTCGTCGACACCATCATCGAAGGCAAGACCGGGTTCCACATGGGCCGCCTCAGCGTCGACGTAAGCCTACCTCTGCCATGTTCTTTCTTCTTTCTTTCTGTATGTATGTATGTATGTACGAATCAGCACCGCCATTCTTGTTTCGTCGTCCTCTCTTCCCAGTGCAACGTCGTGGAGCCGGCGGACGTCAAGAAGGTGGCCACCACCTTGCAGCGCGCCATCAAGGTGGTCGGCACGCCGGCGTACGAGGAGATGGTGAGGAACTGCATGATCCAGGATCTCTCCTGGAAGGTACGTACGCCCGCCCCGCCAGAGCAGAGCGCCAAGATCGATCGACCGACCGACCACACGTACGCGCCTCGCTCCTGTCGCTGACCGTGGTTTAATTTGCGAAATGCGCAGGGCCCTGCCAAGAACTGGGAGAACGTGCTGCTCAGCCTCGGGGTCGCTGGCGGCGAGCCAGGGGTCGAAGGCGAGGAGATCGCGCCGCTCGCCAAGGAGAACGTGGCCGCGCCCTGAAGAGTTCGGCCTGCAGGGCCCCTGATCTCGCGCGTGGTGCAAAGATGTTGGGACATCTTCTTATATATGCTGTTTCGTTTATGTGATATGGACAAGTATGTGTAGCTGCTTGCTTGTGCTAGTGTAATATAGTGTAGTGGTGGCCAGTGGCACAACCTAATAAGCGCATGAACTAATTGCTTGCGTGTGTAGTTAAGTACCGATCGGTAATTTTATAT |
| SWL165 | 98.82 | CCACAACTGTTCGCGTCCTGCTGGTTCATTATCTGACCTGATTGCATTATTGCAGCTACGAGAAGCCCGTGGAAGGCCGGAAGATCAACTGGATGAAGGCCGGGATCCTCGAGGCCGACAGGGTCCTCACCGTCAGCCCCTACTACGCCGAGGAGCTCATCTCCGGCATCGCCAGGGGCTGCGAGCTCGACAACATCATGCGCCTCACCGGCATCACCGGCATCGTCAACGGCATGGACGTCAGCGAGTGGGACCCCAGCAGGGACAAGTACATCGCCGTGAAGTACGACGTGTCGACGGTGAGCTGGCTAGCTAGCTGATTCTGCTGCCTGGTCCTCCTGCTCATGCTGGTTCGGTTCTGACGCGGCAAGTGTACGTACGTGCGTGCGACGGTGGTGTGGTGTCCGGTTCAGGCCGTGGAGGCCAAGGCGCTGAACAAGGAGGCGCTGCAGGCGGAGGTCGGGCTCCCGGTGGACCGGAACATCCCGCTGGTGGCGTTCATCGGCAGGCTGGAAGAGCAGAAGGGACCCGACGTCATGGCGGCCGCCATCCCGCAGCTCATGGAGATGGTGGAGGACGTGCAGATCGTTCTGCTGGTACGTGTGCGCCGCCCGCCACCCGGCTACTACATGCGTGTATCGTTCGTTCTACTGGAACATGCGTGTGAGCAACGCGATGGATAATGCTGCAGGGCACGGGCAAGAAGAAGTTCGAGCGCATGCTCATGAGCGCCGAGGAGAAGTTCCCAGGCAAGGTGCGCGCCGTGGTCAAGTTCAACGCGGCGCTGGCGCACCACATCATGGCCGGCGCCGACGTGCTCGCCGTCACCAGCCGCTTCGAGCCCTGCGGCCTCATCCAGCTGCAGGGGATGCGATACGGAACGGTACGAGAGAGAAAAAAAAACATCCTGAATCCTGACGAGAGGGACAGAGACAGATTGATTATGAATGCTTCATCGATTTGAATTGATTGATCGATGTCTCCCGCTGCGACTCTTGCAGCCCTGCGCCTGCGCGTCCACCGGTGGACTCGTCGACACCATCATCGAAGGCAAGACCGGGTTCCACATGGGCCGCCTCAGCGTCGACGTAAGCCTACCTCTGCCATGTTCTTTCTTCTTTCTTTCTGTATGTATGTATGTATGTACGAATCAGCACCGCCATTCTTGTTTCGTCGTCCTCTCTTCCCAGTGCAACGTCGTGGAGCCGGCGGACGTCAAGAAGGTGGCCACCACCTTGCAGCGCGCCATCAAGGTGGTCGGCACGCCGGCGTACGAGGAGATGGTGAGGAACTGCATGATCCAGGATCTCTCCTGGAAGGTACGTACGCCCGCCCCGCCAGAGCAGAGCGCCAAGATCGATCGACCGACCGACCACACGTACGCGCCTCGCTCCTGTCGCTGACCGTGGTTTAATTTGCGAAATGCGCAGGGCCCTGCCAAGAACTGGGAGAACGTGCTGCTCAGCCTCGGGGTCGCTGGCGGCGAGCCAGGGGTCGAAGGCGAGGAGATCGCGCCGCTCGCCAAGGAGAACGTGGCCGCGCCCTGAAGAGTTCGGCCTGCAGGGCCCCTGATCTCGCGCGTGGTGCAAAGATGTTGGGACATCTTCTTATATATGCTGTTTCGTTTATGTGATATGGACAAGTATGTGTAGCTGCTTGCTTGTGCTAGTGTAATATAGTGTAGTGGTGGCCAGTGGCACAACCTAATAAGCGCATGAACTAATTGCTTGCGTGTGTAGTTAAGTACCGATCGGTAATTTTATAT |

**Table S1 Continued**

| **Accession** | **AC(%)** | **DNA Sequence** |
| --- | --- | --- |
| SWL166 | 97.85 | CCACAACTGTTCGCGTCCTGCTGGTTCATTATCTGACCTGATTGCATTATTGCAGCTACGAGAAGCCCGTGGAAGGCCGGAAGATCAACTGGATGAAGGCCGGGATCCTCGAGGCCGACAGGGTCCTCACCGTCAGCCCCTACTACGCCGAGGAGCTCATCTCCGGCATCGCCAGGGGCTGCGAGCTCGACAACATCATGCGCCTCACCGGCATCACCGGCATCGTCAACGGCATGGACGTCAGCGAGTGGGACCCCAGCAGGGACAAGTACATCGCCGTGAAGTACGACGTGTCGACGGTGAGCAGGCTAGCTAGCTGATTCTGCTGCCTGGTCCTCCTGCTCATGCTGGTTCGGTTCTGACGCGGCAAGTGTACGTACGTGCGTGCGACGGTGGTGTGGTGTCCGGTTCAGGCCGTGGAGGCCAAGGCGCTGAACAAGGAGGCGCTGCAGGCGGAGGTCGGGCTCCCGGTGGACCGGAACATCCCGCTGGTGGCGTTCATCGGCAGGCTGGAAGAGCAGAAGGGACCCGACGTCATGGCGGCCGCCATCCCGCAGCTCATGGAGATGGTGGAGGACGTGCAGATCGTTCTGCTGGTACGTGTGCGCCGCCCGCCACCCGGCTACTACATGCGTGTATCGTTCGTTCTACTGGAACATGCGTGTGAGCAACGCGATGGATAATGCTGCAGGGCACGGGCAAGAAGAAGTTCGAGCGCATGCTCATGAGCGCCGAGGAGAAGTTCCCAGGCAAGGTGCGCGCCGTGGTCAAGTTCAACGCGGCGCTGGCGCACCACATCATGGCCGGCGCCGACGTGCTCGCCGTCACCAGCCGCTTCGAGCCCTGCGGCCTCATCCAGCTGCAGGGGATGCGATACGGAACGGTACGAGAGAGAAAAAAAAACATCCTGAATCCTGACGAGAGGGACAGAGACAGATTGATTATGAATGCTTCATCGATTTGAATTGATTGATCGATGTCTCCCGCTGCGACTCTTGCAGCCCTGCGCCTGCGCGTCCACCGGTGGACTCGTCGACACCATCATCGAAGGCAAGACCGGGTTCCACATGGGCCGCCTCAGCGTCGACGTAAGCCTACCTCTGCCATGTTCTTTCTTCTTTCTTTCTGTATGTATGTATGTATGTACGAATCAGCACCGCCATTCTTGTTTCGTCGTCCTCTCTTCCCAGTGCAACGTCGTGGAGCCGGCGGACGTCAAGAAGGTGGCCACCACCTTGCAGCGCGCCATCAAGGTGGTCGGCACGCCGGCGTACGAGGAGATGGTGAGGAACTGCATGATCCAGGATCTCTCCTGGAAGGTACGTACGCCCGCCCCGCCAGAGCAGAGCGCCAAGATCGATCGACCGACCGACCACACGTACGCGCCTCGCTCCTGTCGCTGACCGTGGTTTAATTTGCGAAATGCGCAGGGCCCTGCCAAGAACTGGGAGAACGTGCTGCTCAGCCTCGGGGTCGCTGGCGGCGAGCCAGGGGTCGAAGGCGAGGAGATCGCGCCGCTCGCCAAGGAGAACGTGGCCGCGCCCTGAAGAGTTCGGCCTGCAGGGCCCCTGATCTCGCGCGTGGTGCAAAGATGTTGGGACATCTTCTTATATATGCTGTTTCGTTTATGTGATATGGACAAGTATGTGTAGCTGCTTGCTTGTGCTAGTGTAATATAGTGTAGTGGTGGCCAGTGGCACAACCTAATAAGCGCATGAACTAATTGCTTGCGTGTGTAGTTAAGTACCGATCGGTAATTTTATAT |
| SWL169 | 97.97 | CCACAACTGTTCGCGTCCTGCTGGTTCATTATCTGACCTGATTGCATTATTGCAGCTACGAGAAGCCCGTGGAAGGCCGGAAGATCAACTGGATGAAGGCCGGGATCCTCGAGGCCGACAGGGTCCTCACCGTCAGCCCCTACTACGCCGAGGAGCTCATCTCCGGCATCGCCAGGGGCTGCGAGCTCGACAACATCATGCGCCTCACCGGCATCACCGGCATCGTCAACGGCATGGACGTCAGCGAGTGGGACCCCAGCAGGGACAAGTACATCGCCGTGAAGTACGACGTGTCGACGGTGAGCTGGCTAGCTAGCTGATTCTGCTGCCTGGTCCTCCTGCTCATGCTGGTTCGGTTCTGACGCGGCAAGTGTACGTACGTGCGTGCGACGGTGGTGTGGTGTCCGGTTCAGGCCGTGGAGGCCAAGGCGCTGAACAAGGAGGCGCTGCAGGCGGAGGTCGGGCTCCCGGTGGACCGGAACATCCCGCTGGTGGCGTTCATCGGCAGGCTGGAAGAGCAGAAGGGACCCGACGTCATGGCGGCCGCCATCCCGCAGCTCATGGAGATGGTGGAGGACGTGCAGATCGTTCTGCTGGTACGTGTGCGCCGCCCGCCACCCGGCTACTACATGCGTGTATCGTTCGTTCTACTGGAACATGCGTGTGAGCAACGCGATGGATAATGCTGCAGGGCACGGGCAAGAAGAAGTTCGAGCGCATGCTCATGAGCGCCGAGGAGAAGTTCCCAGGCAAGGTGCGCGCCGTGGTCAAGTTCAACGCGGCGCTGGCGCACCACATCATGGCCGGCGCCGACGTGCTCGCCGTCACCAGCCGCTTCGAGCCCTGCGGCCTCATCCAGCTGCAGGGGATGCGATACGGAACGGTACGAGAGAGAAAAAAAAACATCCTGAATCCTGACGAGAGGGACAGAGACAGATTGATTATGAATGCTTCATCGATTTGAATTGATTGATCGATGTCTCCCGCTGCGACTCTTGCAGCCCTGCGCCTGCGCGTCCACCGGTGGACTCGTCGACACCATCATCGAAGGCAAGACCGGGTTCCACATGGGCCGCCTCAGCGTCGACGTAAGCCTACCTCTGCCATGTTCTTTCTTCTTTCTTTCTGTATGTATGTATGTATGTACGAATCAGCACCGCCATTCTTGTTTCGTCGTCCTCTCTTCCCAGTGCAACGTCGTGGAGCCGGCGGACGTCAAGAAGGTGGCCACCACCTTGCAGCGCGCCATCAAGGTGGTCGGCACGCCGGCGTACGAGGAGATGGTGAGGAACTGCATGATCCAGGATCTCTCCTGGAAGGTACGTACGCCCGCCCCGCCAGAGCAGAGCGCCAAGATCGATCGACCGACCGACCACACGTACGCGCCTCGCTCCTGTCGCTGACCGTGGTTTAATTTGCGAAATGCGCAGGGCCCTGCCAAGAACTGGGAGAACGTGCTGCTCAGCCTCGGGGTCGCTGGCGGCGAGCCAGGGGTCGAAGGCGAGGAGATCGCGCCGCTCGCCAAGGAGAACGTGGCCGCGCCCTGAAGAGTTCGGCCTGCAGGGCCCCTGATCTCGCGCGTGGTGCAAAGATGTTGGGACATCTTCTTATATATGCTGTTTCGTTTATGTGATATGGACAAGTATGTGTAGCTGCTTGCTTGTGCTAGTGTAATATAGTGTAGTGGTGGCCAGTGGCACAACCTAATAAGCGCATGAACTAATTGCTTGCGTGTGTAGTTAAGTACCGATCGGTAATTTTATAT |

**Table S1 Continued**

| **Accession** | **AC(%)** | **DNA Sequence** |
| --- | --- | --- |
| SWL170 | 99.38 | CCACAACTGTTCGCGTCCTGCTGGTTCATTATCTGACCTGATTGCATTATTGCAGCTACGAGAAGCCCGTGGAAGGCCGGAAGATCAACTGGATGAAGGCCGGGATCCTCGAGGCCGACAGGGTCCTCACCGTCAGCCCCTACTACGCCGAGGAGCTCATCTCCGGCATCGCCAGGGGCTGCGAGCTCGACAACATCATGCGCCTCACCGGCATCACCGGCATCGTCAACGGCATGGACGTCAGCGAGTGGGACCCCAGCAGGGACAAGTACATCGCCGTGAAGTACGACGTGTCGACGGTGAGCTGGCTAGCTAGCTGATTCTGCTGCCTGGTCCTCCTGCTCATGCTGGTTCGGTTCTGACGCGGCAAGTGTACGTACGTGCGTGCGACGGTGGTGTGGTGTCCGGTTCAGGCCGTGGAGGCCAAGGCGCTGAACAAGGAGGCGCTGCAGGCGGAGGTCGGGCTCCCGGTGGACCGGAACATCCCGCTGGTGGCGTTCATCGGCAGGCTGGAAGAGCAGAAGGGACCCGACGTCATGGCGGCCGCCATCCCGCAGCTCATGGAGATGGTGGAGGACGTGCAGATCGTTCTGCTGGTACGTGTGCGCCGCCCGCCACCCGGCTACTACATGCGTGTATCGTTCGTTCTACTGGAACATGCGTGTGAGCAACGCGATGGATAATGCTGCAGGGCACGGGCAAGAAGAAGTTCGAGCGCATGCTCATGAGCGCCGAGGAGAAGTTCCCAGGCAAGGTGCGCGCCGTGGTCAAGTTCAACGCGGCGCTGGCGCACCACATCATGGCCGGCGCCGACGTGCTCGCCGTCACCAGCCGCTTCGAGCCCTGCGGCCTCATCCAGCTGCAGGGGATGCGATACGGAACGGTACGAGAGAGAAAAAAAAACATCCTGAATCCTGACGAGAGGGACAGAGACAGATTGATTATGAATGCTTCATCGATTTGAATTGATTGATCGATGTCTCCCGCTGCGACTCTTGCAGCCCTGCGCCTGCGCGTCCACCGGTGGACTCGTCGACACCATCATCGAAGGCAAGACCGGGTTCCACATGGGCCGCCTCAGCGTCGACGTAAGCCTACCTCTGCCATGTTCTTTCTTCTTTCTTTCTGTATGTATGTATGTATGTACGAATCAGCACCGCCATTCTTGTTTCGTCGTCCTCTCTTCCCAGTGCAACGTCGTGGAGCCGGCGGACGTCAAGAAGGTGGCCACCACCTTGCAGCGCGCCATCAAGGTGGTCGGCACGCCGGCGTACGAGGAGATGGTGAGGAACTGCATGATCCAGGATCTCTCCTGGAAGGTACGTACGCCCGCCCCGCCAGAGCAGAGCGCCAAGATCGATCGACCGACCGACCACACGTACGCGCCTCGCTCCTGTCGCTGACCGTGGTTTAATTTGCGAAATGCGCAGGGCCCTGCCAAGAACTGGGAGAACGTGCTGCTCAGCCTCGGGGTCGCTGGCGGCGAGCCAGGGGTCGAAGGCGAGGAGATCGCGCCGCTCGCCAAGGAGAACGTGGCCGCGCCCTGAAGAGTTCGGCCTGCAGGGCCCCTGATCTCGCGCGTGGTGCAAAGATGTTGGGACATCTTCTTATATATGCTGTTTCGTTTATGTGATATGGACAAGTATGTGTAGCTGCTTGCTTGTGCTAGTGTAATATAGTGTAGTGGTGGCCAGTGGCACAACCTAATAAGCGCATGAACTAATTGCTTGCGTGTGTAGTTAAGTACCGATCGGTAATTTTATAT |
| SWL171 | 99.81 | CCACAACTGTTCGCGTCCTGCTGGTTCATTATCTGACCTTGATTGCATTGCAGCTACGAGAAGCCCGTGGAAGGCCGGAAGATCAACTGGATGAAGGCCGGGATCCTCGAGGCCGACAGGGTCCTCACCGTCAGCCCCTACTACGCCGAGGAGCTCATCTCCGGCATCGCCAGGGGCTGCGAGCTCGACAACATCATGCGCCTCACCGGCATCACCGGCATCGTCAACGGCATGGACGTCAGCGAGTGGGACCCCAGCAGGGACAAGTACATCGCCGTGAAGTACGACGTGTCGACGGTGAGCTGGCTAGCTAGCTGATTCTGCTGCCTGGTCCTCCTGCTCATGCTGGTTCGGTTCTGACGCGGCGAGTGTACGTACGTGCGTGCGACGGTGGTGTGGTGTCCGGTTCAGGCCGTGGAGGCCAAGGCGCTGCAGGCGGAGGTCGGGCTCCCGGTGGACCGGAACATCCCGCTGGTGGCGTTCATCGGCAGGCTGGAAGAGCAGAAGGGACCCGACGTCATGGCGGCCGCCATCCCGCAGCTCATGGAGATGGTGGAGGACGTGCAGATCGTTCTGCTGGTACGTGTGCGCCGCCCGCCACCCGGCTACTACATGCGTGTATCGTTCTACTGGAACATACGTGTGAGCAACGCGATGGATAATGCTGCAGGGCACGGGCAAGAAGAAGTTCGAGCGCATGCTCATGAGCGCCGAGGAGAAGTTCCCAGGCAAGGTGCGCGCCGTGGTCAAGTTCAACGCGGCGCTGGCGCACCACATCATGGCCGGCGCCGACGTGCTCGCCGTCACCAGCCGCTTCGAGCCCTGCGGCCTCATCCAGCTGCAGGGGATGCGATACGGAACGGTACGAGAGAGAAAAAAAAACATCCTGAATCCTGACGAGAGGGACAGAGACAGATTGATTATGAATGCTTCATCGATTTGAATTGATTGATCGATGTCTCCCGCTGCGACTCTTGCAGCCCTGCGCCTGCGCGTCCACCGGTGGACTCGTCGACACCATCATCGAAGGCAAGACCGGGTTCCACATGGGCCGCCTCAGCGTCGACGTAAGCCTAGCTCTGCCATGATCTTTCTTCTTTCTGTATGTATGTATGTATGAATCAGCACCGCCGTTCTTGTTTCGTCGTCCTCTCTTCCCAGTGCAACGTCGTGGAGCCGGCGGACGTCAAGAAGGTGGCCACCACCTTGCAGCGCGCCATCAAGGTGGTCGGCACGCCGGCGTACGAGGAGATGGTGAGGAACTGCATGATCCAGGATCTCTCCTGGAAGGTACGTACGCCCGCCCCGCCAGAGCAGAGCGCCAAGATCGATCGACCGACCGACCACACGTACGCGCCTCGCTCCTGTCGCTGACCGTGGTTTAATTTGCGAAATGCGCAGGGCCCTGCCAAGAACTGGGAGAACGTGCTGCTCAGCCTCGGGGTCGCCGGCGGCGAGCCAGGGGTCGAAGGCGAGGAGATCGCGCCGCTCGCCAAGGAGAACGTGGCCGCGCCCTGAAGAGTTCGGCCTGCAGGCCCCCTGATCTCGCGCGTGGTGCAAACATGTTGGGACATCTTCTTATATATGCTGTTTCGTTTATGTGATATGGACAAGTATGTGTAGCTGCTTGCTTGTGCTAGTGTAATATAATAGTGTAGTGGTGGCCAGTGGCACAACCTAATAAGCGCATGAACTAATTGCTTGCGTGTGTAGTTAAGTACCGATCGGTAATTTTATAT |

**Table S1 Continued**

| **Accession** | **AC(%)** | **DNA Sequence** |
| --- | --- | --- |
| SWL172 | 98.53 | CCACAACTGTTCGCGTCCTGCTGGTTCATTATCTGACCTGATTGCATTATTGCAGCTACGAGAAGCCCGTGGAAGGCCGGAAGATCAACTGGATGAAGGCCGGGATCCTCGAGGCCGACAGGGTCCTCACCGTCAGCCCCTACTACGCCGAGGAGCTCATCTCCGGCATCGCCAGGGGCTGCGAGCTCGACAACATCATGCGCCTCACCGGCATCACCGGCATCGTCAACGGCATGGACGTCAGCGAGTGGGACCCCAGCAGGGACAAGTACATCGCCGTGAAGTACGACGTGTCGACGGTGAGCTGGCTAGCTAGCTGATTCTGCTGCCTGGTCCTCCTGCTCATGCTGGTTCGGTTCTGACGCGGCAAGTGTACGTACGTGCGTGCGACGGTGGTGTGGTGTCCGGTTCAGGCCGTGGAGGCCAAGGCGCTGAACAAGGAGGCGCTGCAGGCGGAGGTCGGGCTCCCGGTGGACCGGAACATCCCGCTGGTGGCGTTCATCGGCAGGCTGGAAGAGCAGAAGGGACCCGACGTCATGGCGGCCGCCATCCCGCAGCTCATGGAGATGGTGGAGGACGTGCAGATCGTTCTGCTGGTACGTGTGCGCCGCCCGCCACCCGGCTACTACATGCGTGTATCGTTCGTTCTACTGGAACATGCGTGTGAGCAACGCGATGGATAATGCTGCAGGGCACGGGCAAGAAGAAGTTCGAGCGCATGCTCATGAGCGCCGAGGAGAAGTTCCCAGGCAAGGTGCGCGCCGTGGTCAAGTTCAACGCGGCGCTGGCGCACCACATCATGGCCGGCGCCGACGTGCTCGCCGTCACCAGCCGCTTCGAGCCCTGCGGCCTCATCCAGCTGCAGGGGATGCGATACGGAACGGTACGAGAGAGAAAAAAAAACATCCTGAATCCTGACGAGAGGGACAGAGACAGATTGATTATGAATGCTTCATCGATTTGAATTGATTGATCGATGTCTCCCGCTGCGACTCTTGCAGCCCTGCGCCTGCGCGTCCACCGGTGGACTCGTCGACACCATCATCGAAGGCAAGACCGGGTTCCACATGGGCCGCCTCAGCGTCGACGTAAGCCTACCTCTGCCATGTTCTTTCTTCTTTCTTTCTGTATGTATGTATGTATGTACGAATCAGCACCGCCATTCTTGTTTCGTCGTCCTCTCTTCCCAGTGCAACGTCGTGGAGCCGGCGGACGTCAAGAAGGTGGCCACCACCTTGCAGCGCGCCATCAAGGTGGTCGGCACGCCGGCGTACGAGGAGATGGTGAGGAACTGCATGATCCAGGATCTCTCCTGGAAGGTACGTACGCCCGCCCCGCCAGAGCAGAGCGCCAAGATCGATCGACCGACCGACCACACGTACGCGCCTCGCTCCTGTCGCTGACCGTGGTTTAATTTGCGAAATGCGCAGGGCCCTGCCAAGAACTGGGAGAACGTGCTGCTCAGCCTCGGGGTCGCTGGCGGCGAGCCAGGGGTCGAAGGCGAGGAGATCGCGCCGCTCGCCAAGGAGAACGTGGCCGCGCCCTGAAGAGTTCGGCCTGCAGGGCCCCTGATCTCGCGCGTGGTGCAAAGATGTTGGGACATCTTCTTATATATGCTGTTTCGTTTATGTGATATGGACAAGTATGTGTAGCTGCTTGCTTGTGCTAGTGTAATATAGTGTAGTGGTGGCCAGTGGCACAACCTAATAAGCGCATGAACTAATTGCTTGCGTGTGTAGTTAAGTACCGATCGGTAATTTTATAT |
| SWL174 | 97.95 | CCACAACTGTTCGCGTCCTGCTGGTTCATTATCTGACCTGATTGCATTATTGCAGCTACGAGAAGCCCGTGGAAGGCCGGAAGATCAACTGGATGAAGGCCGGGATCCTCGAGGCCGACAGGGTCCTCACCGTCAGCCCCTACTACGCCGAGGAGCTCATCTCCGGCATCGCCAGGGGCTGCGAGCTCGACAACATCATGCGCCTCACCGGCATCACCGGCATCGTCAACGGCATGGACGTCAGCGAGTGGGACCCCAGCAGGGACAAGTACATCGCCGTGAAGTACGACGTGTCGACGGTGAGCTGGCTAGCTAGCTGATTCTGCTGCCTGGTCCTCCTGCTCATGCTGGTTCGGTTCTGACGCGGCAAGTGTACGTACGTGCGTGCGACGGTGGTGTGGTGTCCGGTTCAGGCCGTGGAGGCCAAGGCGCTGAACAAGGAGGCGCTGCAGGCGGAGGTCGGGCTCCCGGTGGACCGGAACATCCCGCTGGTGGCGTTCATCGGCAGGCTGGAAGAGCAGAAGGGACCCGACGTCATGGCGGCCGCCATCCCGCAGCTCATGGAGATGGTGGAGGACGTGCAGATCGTTCTGCTGGTACGTGTGCGCCGCCCGCCACCCGGCTACTACATGCGTGTATCGTTCGTTCTACTGGAACATGCGTGTGAGCAACGCGATGGATAATGCTGCAGGGCACGGGCAAGAAGAAGTTCGAGCGCATGCTCATGAGCGCCGAGGAGAAGTTCCCAGGCAAGGTGCGCGCCGTGGTCAAGTTCAACGCGGCGCTGGCGCACCACATCATGGCCGGCGCCGACGTGCTCGCCGTCACCAGCCGCTTCGAGCCCTGCGGCCTCATCCAGCTGCAGGGGATGCGATACGGAACGGTACGAGAGAGAAAAAAAAACATCCTGAATCCTGACGAGAGGGACAGAGACAGATTGATTATGAATGCTTCATCGATTTGAATTGATTGATCGATGTCTCCCGCTGCGACTCTTGCAGCCCTGCGCCTGCGCGTCCACCGGTGGACTCGTCGACACCATCATCGAAGGCAAGACCGGGTTCCACATGGGCCGCCTCAGCGTCGACGTAAGCCTACCTCTGCCATGTTCTTTCTTCTTTCTTTCTGTATGTATGTATGTATGTACGAATCAGCACCGCCATTCTTGTTTCGTCGTCCTCTCTTCCCAGTGCAACGTCGTGGAGCCGGCGGACGTCAAGAAGGTGGCCACCACCTTGCAGCGCGCCATCAAGGTGGTCGGCACGCCGGCGTACGAGGAGATGGTGAGGAACTGCATGATCCAGGATCTCTCCTGGAAGGTACGTACGCCCGCCCCGCCAGAGCAGAGCGCCAAGATCGATCGACCGACCGACCACACGTACGCGCCTCGCTCCTGTCGCTGACCGTGGTTTAATTTGCGAAATGCGCAGGGCCCTGCCAAGAACTGGGAGAACGTGCTGCTCAGCCTCGGGGTCGCTGGCGGCGAGCCAGGGGTCGAAGGCGAGGAGATCGCGCCGCTCGCCAAGGAGAACGTGGCCGCGCCCTGAAGAGTTCGGCCTGCAGGGCCCCTGATCTCGCGCGTGGTGCAAAGATGTTGGGACATCTTCTTATATATGCTGTTTCGTTTATGTGATATGGACAAGTATGTGTAGCTGCTTGCTTGTGCTAGTGTAATATAGTGTAGTGGTGGCCAGTGGCACAACCTAATAAGCGCATGAACTAATTGCTTGCGTGTGTAGTTAAGTACCGATCGGTAATTTTATAT |

**Table S1 Continued**

| **Accession** | **AC(%)** | **DNA Sequence** |
| --- | --- | --- |
| SWL175 | 98.27 | CCACAACTGTTCGCGTCCTGCTGGTTCATTATCTGACCTGATTGCATTATTGCAGCTACGAGAAGCCCGTGGAAGGCCGGAAGATCAACTGGATGAAGGCCGGGATCCTCGAGGCCGACAGGGTCCTCACCGTCAGCCCCTACTACGCCGAGGAGCTCATCTCCGGCATCGCCAGGGGCTGCGAGCTCGACAACATCATGCGCCTCACCGGCATCACCGGCATCGTCAACGGCATGGACGTCAGCGAGTGGGACCCCAGCAGGGACAAGTACATCGCCGTGAAGTACGACGTGTCGACGGTGAGCTGGCTAGCTAGCTGATTCTGCTGCCTGGTCCTCCTGCTCATGCTGGTTCGGTTCTGACGCGGCAAGTGTACGTACGTGCGTGCGACGGTGGTGTGGTGTCCGGTTCAGGCCGTGGAGGCCAAGGCGCTGAACAAGGAGGCGCTGCAGGCGGAGGTCGGGCTCCCGGTGGACCGGAACATCCCGCTGGTGGCGTTCATCGGCAGGCTGGAAGAGCAGAAGGGACCCGACGTCATGGCGGCCGCCATCCCGCAGCTCATGGAGATGGTGGAGGACGTGCAGATCGTTCTGCTGGTACGTGTGCGCCGCCCGCCACCCGGCTACTACATGCGTGTATCGTTCGTTCTACTGGAACATGCGTGTGAGCAACGCGATGGATAATGCTGCAGGGCACGGGCAAGAAGAAGTTCGAGCGCATGCTCATGAGCGCCGAGGAGAAGTTCCCAGGCAAGGTGCGCGCCGTGGTCAAGTTCAACGCGGCGCTGGCGCACCACATCATGGCCGGCGCCGACGTGCTCGCCGTCACCAGCCGCTTCGAGCCCTGCGGCCTCATCCAGCTGCAGGGGATGCGATACGGAACGGTACGAGAGAGAAAAAAAAACATCCTGAATCCTGACGAGAGGGACAGAGACAGATTGATTATGAATGCTTCATCGATTTGAATTGATTGATCGATGTCTCCCGCTGCGACTCTTGCAGCCCTGCGCCTGCGCGTCCACCGGTGGACTCGTCGACACCATCATCGAAGGCAAGACCGGGTTCCACATGGGCCGCCTCAGCGTCGACGTAAGCCTACCTCTGCCATGTTCTTTCTTCTTTCTTTCTGTATGTATGTATGTATGTACGAATCAGCACCGCCATTCTTGTTTCGTCGTCCTCTCTTCCCAGTGCAACGTCGTGGAGCCGGCGGACGTCAAGAAGGTGGCCACCACCTTGCAGCGCGCCATCAAGGTGGTCGGCACGCCGGCGTACGAGGAGATGGTGAGGAACTGCATGATCCAGGATCTCTCCTGGAAGGTACGTACGCCCGCCCCGCCAGAGCAGAGCGCCAAGATCGATCGACCGACCGACCACACGTACGCGCCTCGCTCCTGTCGCTGACCGTGGTTTAATTTGCGAAATGCGCAGGGCCCTGCCAAGAACTGGGAGAACGTGCTGCTCAGCCTCGGGGTCGCTGGCGGCGAGCCAGGGGTCGAAGGCGAGGAGATCGCGCCGCTCGCCAAGGAGAACGTGGCCGCGCCCTGAAGAGTTCGGCCTGCAGGGCCCCTGATCTCGCGCGTGGTGCAAAGATGTTGGGACATCTTCTTATATATGCTGTTTCGTTTATGTGATATGGACAAGTATGTGTAGCTGCTTGCTTGTGCTAGTGTAATATAGTGTAGTGGTGGCCAGTGGCACAACCTAATAAGCGCATGAACTAATTGCTTGCGTGTGTAGTTAAGTACCGATCGGTAATTTTATAT |
| SWL176 | 97.99 | CCACAACTGTTCGCGTCCTGCTGGTTCATTATCTGACCTGATTGCATTATTGCAGCTACGAGAAGCCCGTGGAAGGCCGGAAGATCAACTGGATGAAGGCCGGGATCCTCGAGGCCGACAGGGTCCTCACCGTCAGCCCCTACTACGCCGAGGAGCTCATCTCCGGCATCGCCAGGGGCTGCGAGCTCGACAACATCATGCGCCTCACCGGCATCACCGGCATCGTCAACGGCATGGACGTCAGCGAGTGGGACCCCAGCAGGGACAAGTACATCGCCGTGAAGTACGACGTGTCGACGGTGAGCTGGCTAGCTAGCTGATTCTGCTGCCTGGTCCTCCTGCTCATGCTGGTTCGGTTCTGACGCGGCAAGTGTACGTACGTGCGTGCGACGGTGGTGTGGTGTCCGGTTCAGGCCGTGGAGGCCAAGGCGCTGAACAAGGAGGCGCTGCAGGCGGAGGTCGGGCTCCCGGTGGACCGGAACATCCCGCTGGTGGCGTTCATCGGCAGGCTGGAAGAGCAGAAGGGACCCGACGTCATGGCGGCCGCCATCCCGCAGCTCATGGAGATGGTGGAGGACGTGCAGATCGTTCTGCTGGTACGTGTGCGCCGCCCGCCACCCGGCTACTACATGCGTGTATCGTTCGTTCTACTGGAACATGCGTGTGAGCAACGCGATGGATAATGCTGCAGGGCACGGGCAAGAAGAAGTTCGAGCGCATGCTCATGAGCGCCGAGGAGAAGTTCCCAGGCAAGGTGCGCGCCGTGGTCAAGTTCAACGCGGCGCTGGCGCACCACATCATGGCCGGCGCCGACGTGCTCGCCGTCACCAGCCGCTTCGAGCCCTGCGGCCTCATCCAGCTGCAGGGGATGCGATACGGAACGGTACGAGAGAGAAAAAAAAACATCCTGAATCCTGACGAGAGGGACAGAGACAGATTGATTATGAATGCTTCATCGATTTGAATTGATTGATCGATGTCTCCCGCTGCGACTCTTGCAGCCCTGCGCCTGCGCGTCCACCGGTGGACTCGTCGACACCATCATCGAAGGCAAGACCGGGTTCCACATGGGCCGCCTCAGCGTCGACGTAAGCCTACCTCTGCCATGTTCTTTCTTCTTTCTTTCTGTATGTATGTATGTATGTACGAATCAGCACCGCCATTCTTGTTTCGTCGTCCTCTCTTCCCAGTGCAACGTCGTGGAGCCGGCGGACGTCAAGAAGGTGGCCACCACCTTGCAGCGCGCCATCAAGGTGGTCGGCACGCCGGCGTACGAGGAGATGGTGAGGAACTGCATGATCCAGGATCTCTCCTGGAAGGTACGTACGCCCGCCCCGCCAGAGCAGAGCGCCAAGATCGATCGACCGACCGACCACACGTACGCGCCTCGCTCCTGTCGCTGACCGTGGTTTAATTTGCGAAATGCGCAGGGCCCTGCCAAGAACTGGGAGAACGTGCTGCTCAGCCTCGGGGTCGCTGGCGGCGAGCCAGGGGTCGAAGGCGAGGAGATCGCGCCGCTCGCCAAGGAGAACGTGGCCGCGCCCTGAAGAGTTCGGCCTGCAGGGCCCCTGATCTCGCGCGTGGTGCAAAGATGTTGGGACATCTTCTTATATATGCTGTTTCGTTTATGTGATATGGACAAGTATGTGTAGCTGCTTGCTTGTGCTAGTGTAATATAGTGTAGTGGTGGCCAGTGGCACAACCTAATAAGCGCATGAACTAATTGCTTGCGTGTGTAGTTAAGTACCGATCGGTAATTTTATAT |

**Table S1 Continued**

| **Accession** | **AC(%)** | **DNA Sequence** |
| --- | --- | --- |
| SWL177 | 98.46 | CCACAACTGTTCGCGTCCTGCTGGTTCATTATCTGACCTGATTGCATTATTGCAGCTACGAGAAGCCCGTGGAAGGCCGGAAGATCAACTGGATGAAGGCCGGGATCCTCGAGGCCGACAGGGTCCTCACCGTCAGCCCCTACTACGCCGAGGAGCTCATCTCCGGCATCGCCAGGGGCTGCGAGCTCGACAACATCATGCGCCTCACCGGCATCACCGGCATCGTCAACGGCATGGACGTCAGCGAGTGGGACCCCAGCAGGGACAAGTACATCGCCGTGAAGTACGACGTGTCGACGGTGAGCTGGCTAGCTAGCTGATTCTGCTGCCTGGTCCTCCTGCTCATGCTGGTTCGGTTCTGACGCGGCAAGTGTACGTACGTGCGTGCGACGGTGGTGTGGTGTCCGGTTCAGGCCGTGGAGGCCAAGGCGCTGAACAAGGAGGCGCTGCAGGCGGAGGTCGGGCTCCCGGTGGACCGGAACATCCCGCTGGTGGCGTTCATCGGCAGGCTGGAAGAGCAGAAGGGACCCGACGTCATGGCGGCCGCCATCCCGCAGCTCATGGAGATGGTGGAGGACGTGCAGATCGTTCTGCTGGTACGTGTGCGCCGCCCGCCACCCGGCTACTACATGCGTGTATCGTTCGTTCTACTGGAACATGCGTGTGAGCAACGCGATGGATAATGCTGCAGGGCACGGGCAAGAAGAAGTTCGAGCGCATGCTCATGAGCGCCGAGGAGAAGTTCCCAGGCAAGGTGCGCGCCGTGGTCAAGTTCAACGCGGCGCTGGCGCACCACATCATGGCCGGCGCCGACGTGCTCGCCGTCACCAGCCGCTTCGAGCCCTGCGGCCTCATCCAGCTGCAGGGGATGCGATACGGAACGGTACGAGAGAGAAAAAAAAACATCCTGAATCCTGACGAGAGGGACAGAGACAGATTGATTATGAATGCTTCATCGATTTGAATTGATTGATCGATGTCTCCCGCTGCGACTCTTGCAGCCCTGCGCCTGCGCGTCCACCGGTGGACTCGTCGACACCATCATCGAAGGCAAGACCGGGTTCCACATGGGCCGCCTCAGCGTCGACGTAAGCCTACCTCTGCCATGTTCTTTCTTCTTTCTTTCTGTATGTATGTATGTATGTACGAATCAGCACCGCCATTCTTGTTTCGTCGTCCTCTCTTCCCAGTGCAACGTCGTGGAGCCGGCGGACGTCAAGAAGGTGGCCACCACCTTGCAGCGCGCCATCAAGGTGGTCGGCACGCCGGCGTACGAGGAGATGGTGAGGAACTGCATGATCCAGGATCTCTCCTGGAAGGTACGTACGCCCGCCCCGCCAGAGCAGAGCGCCAAGATCGATCGACCGACCGACCACACGTACGCGCCTCGCTCCTGTCGCTGACCGTGGTTTAATTTGCGAAATGCGCAGGGCCCTGCCAAGAACTGGGAGAACGTGCTGCTCAGCCTCGGGGTCGCTGGCGGCGAGCCAGGGGTCGAAGGCGAGGAGATCGCGCCGCTCGCCAAGGAGAACGTGGCCGCGCCCTGAAGAGTTCGGCCTGCAGGGCCCCTGATCTCGCGCGTGGTGCAAAGATGTTGGGACATCTTCTTATATATGCTGTTTCGTTTATGTGATATGGACAAGTATGTGTAGCTGCTTGCTTGTGCTAGTGTAATATAGTGTAGTGGTGGCCAGTGGCACAACCTAATAAGCGCATGAACTAATTGCTTGCGTGTGTAGTTAAGTACCGATCGGTAATTTTATAT |
| SWL178 | 98.73 | CCACAACTGTTCGCGTCCTGCTGGTTCATTATCTGACCTGATTGCATTATGCAGCTACGAGAAGCCCGTGGAAGGCCGGAAGATCAACTGGATGAAGGCCGGGATCCTCGAGGCCGACAGGGTCCTCACCGTCAGCCCCTACTACGCCGAGGAGCTCATCTCCGGCATCGCCAGGGGCTGCGAGCTCGACAACATCATGCGCCTCACCGGCATCACCGGCATCGTCAACGGCATGGACGTCAGCGAGTGGGACCCCAGCAGGGACAAGTACATCGCCGTGAAGTACGACGTGTCGACGGTGAGCTGGCTAGCTAGCTGATTCTGCTGCCTGGTCCTCCTGCTCATGCTGGTTCGGTTCTGACGCGGCAAGTGTACGTACGTGCGTGCGACGGTGGTGTGGTGTCCGGTTCAGGCCGTGGAGGCCAAGGCGCTGAACAAGGAGGCGCTGCAGGCGGAGGTCGGGCTCCCGGTGGACCGGAACATCCCGCTGGTGGCGTTCATCGGCAGGCTGGAAGAGCAGAAGGGACCCGACGTCATGGCGGCCGCCATCCCGCAGCTCATGGAGATGGTGGAGGACGTGCAGATCGTTCTGCTGGTACGTGTGCGCCGCCCGCCACCCGGCTACTACATGCGTGTATCGTTCGTTCTACTGGAACATGCGTGTGAGCAACGCGATGGATAATGCTGCAGGGCACGGGCAAGAAGAAGTTCGAGCGCATGCTCATGAGCGCCGAGGAGAAGTTCCCAGGCAAGGTGCGCGCCGTGGTCAAGTTCAACGCGGCGCTGGCGCACCACATCATGGCCGGCGCCGACGTGCTCGCCGTCACCAGCCGCTTCGAGCCCTGCGGCCTCATCCAGCTGCAGGGGATGCGATACGGAACGGTACGAGAGAGAAAAAAAAACATCCTGAATCCTGACGAGAGGGACAGAGACAGATTGATTATGAATGCTTCATCGATTTGAATTGATTGATCGATGTCTCCCGCTGCGACTCTTGCAGCCCTGCGCCTGCGCGTCCACCGGTGGACTCGTCGACACCATCATCGAAGGCAAGACCGGGTTCCACATGGGCCGCCTCAGCGTCGACGTAAGCCTACCTCTGCCATGTTCTTTCTTCTTTCTTTCTGTATGTATGTATGTATGTACGAATCAGCACCGCCATTCTTGTTTCGTCGTCCTCTCTTCCCAGTGCAACGTCGTGGAGCCGGCGGACGTCAAGAAGGTGGCCACCACCTTGCAGCGCGCCATCAAGGTGGTCGGCACGCCGGCGTACGAGGAGATGGTGAGGAACTGCATGATCCAGGATCTCTCCTGGAAGGTACGTACGCCCGCCCCGCCAGAGCAGAGCGCCAAGATCGATCGACCGACCGACCACACGTACGCGCCTCGCTCCTGTCGCTGACCGTGGTTTAATTTGCGAAATGCGCAGGGCCCTGCCAAGAACTGGGAGAACGTGCTGCTCAGCCTCGGGGTCGCTGGCGGCGAGCCAGGGGTCGAAGGCGAGGAGATCGCGCCGCTCGCCAAGGAGAACGTGGCCGCGCCCTGAAGAGTTCGGCCTGCAGGGCCCCTGATCTCGCGCGTGGTGCAAAGATGTTGGGACATCTTCTTATATATGCTGTTTCGTTTATGTGATATGGACAAGTATGTGTAGCTGCTTGCTTGTGCTAGTGTAATATAGTGTAGTGGTGGCCAGTGGCACAACCTAATAAGCGCATGAACTAATTGCTTGCGTGTGTAGTTAAGTACCGATCGGTAATTTTATAT |

**Table S1 Continued**

| **Accession** | **AC(%)** | **DNA Sequence** |
| --- | --- | --- |
| SWL179 | 98.36 | CCACAACTGTTCGCGTCCTGCTGGTTCATTATCTGACCTGATTGCATTATTGCAGCTACGAGAAGCCCGTGGAAGGCCGGAAGATCAACTGGATGAAGGCCGGGATCCTCGAGGCCGACAGGGTCCTCACCGTCAGCCCCTACTACGCCGAGGAGCTCATCTCCGGCATCGCCAGGGGCTGCGAGCTCGACAACATCATGCGCCTCACCGGCATCACCGGCATCGTCAACGGCATGGACGTCAGCGAGTGGGACCCCAGCAGGGACAAGTACATCGCCGTGAAGTACGACGTGTCGACGGTGAGCTGGCTAGCTAGCTGATTCTGCTGCCTGGTCCTCCTGCTCATGCTGGTTCGGTTCTGACGCGGCAAGTGTACGTACGTGCGTGCGACGGTGGTGTGGTGTCCGGTTCAGGCCGTGGAGGCCAAGGCGCTGAACAAGGAGGCGCTGCAGGCGGAGGTCGGGCTCCCGGTGGACCGGAACATCCCGCTGGTGGCGTTCATCGGCAGGCTGGAAGAGCAGAAGGGACCCGACGTCATGGCGGCCGCCATCCCGCAGCTCATGGAGATGGTGGAGGACGTGCAGATCGTTCTGCTGGTACGTGTGCGCCGCCCGCCACCCGGCTACTACATGCGTGTATCGTTCGTTCTACTGGAACATGCGTGTGAGCAACGCGATGGATAATGCTGCAGGGCACGGGCAAGAAGAAGTTCGAGCGCATGCTCATGAGCGCCGAGGAGAAGTTCCCAGGCAAGGTGCGCGCCGTGGTCAAGTTCAACGCGGCGCTGGCGCACCACATCATGGCCGGCGCCGACGTGCTCGCCGTCACCAGCCGCTTCGAGCCCTGCGGCCTCATCCAGCTGCAGGGGATGCGATACGGAACGGTACGAGAGAGAAAAAAAAACATCCTGAATCCTGACGAGAGGGACAGAGACAGATTGATTATGAATGCTTCATCGATTTGAATTGATTGATCGATGTCTCCCGCTGCGACTCTTGCAGCCCTGCGCCTGCGCGTCCACCGGTGGACTCGTCGACACCATCATCGAAGGCAAGACCGGGTTCCACATGGGCCGCCTCAGCGTCGACGTAAGCCTACCTCTGCCATGTTCTTTCTTCTTTCTTTCTGTATGTATGTATGTATGTACGAATCAGCACCGCCATTCTTGTTTCGTCGTCCTCTCTTCCCAGTGCAACGTCGTGGAGCCGGCGGACGTCAAGAAGGTGGCCACCACCTTGCAGCGCGCCATCAAGGTGGTCGGCACGCCGGCGTACGAGGAGATGGTGAGGAACTGCATGATCCAGGATCTCTCCTGGAAGGTACGTACGCCCGCCCCGCCAGAGCAGAGCGCCAAGATCGATCGACCGACCGACCACACGTACGCGCCTCGCTCCTGTCGCTGACCGTGGTTTAATTTGCGAAATGCGCAGGGCCCTGCCAAGAACTGGGAGAACGTGCTGCTCAGCCTCGGGGTCGCTGGCGGCGAGCCAGGGGTCGAAGGCGAGGAGATCGCGCCGCTCGCCAAGGAGAACGTGGCCGCGCCCTGAAGAGTTCGGCCTGCAGGGCCCCTGATCTCGCGCGTGGTGCAAAGATGTTGGGACATCTTCTTATATATGCTGTTTCGTTTATGTGATATGGACAAGTATGTGTAGCTGCTTGCTTGTGCTAGTGTAATATAGTGTAGTGGTGGCCAGTGGCACAACCTAATAAGCGCATGAACTAATTGCTTGCGTGTGTAGTTAAGTACCGATCGGTAATTTTATAT |
| SWL180 | 98.20 | CCACAACTGTTCGCGTCCTGCTGGTTCATTATCTGACCTGATTGCATTATTGCAGCTACGAGAAGCCCGTGGAAGGCCGGAAGATCAACTGGATGAAGGCCGGGATCCTCGAGGCCGACAGGGTCCTCACCGTCAGCCCCTACTACGCCGAGGAGCTCATCTCCGGCATCGCCAGGGGCTGCGAGCTCGACAACATCATGCGCCTCACCGGCATCACCGGCATCGTCAACGGCATGGACGTCAGCGAGTGGGACCCCAGCAGGGACAAGTACATCGCCGTGAAGTACGACGTGTCGACGGTGAGCTGGCTAGCTAGCTGATTCTGCTGCCTGGTCCTCCTGCTCATGCTGGTTCGGTTCTGACGCGGCAAGTGTACGTACGTGCGTGCGACGGTGGTGTGGTGTCCGGTTCAGGCCGTGGAGGCCAAGGCGCTGAACAAGGAGGCGCTGCAGGCGGAGGTCGGGCTCCCGGTGGACCGGAACATCCCGCTGGTGGCGTTCATCGGCAGGCTGGAAGAGCAGAAGGGACCCGACGTCATGGCGGCCGCCATCCCGCAGCTCATGGAGATGGTGGAGGACGTGCAGATCGTTCTGCTGGTACGTGTGCGCCGCCCGCCACCCGGCTACTACATGCGTGTATCGTTCGTTCTACTGGAACATGCGTGTGAGCAACGCGATGGATAATGCTGCAGGGCACGGGCAAGAAGAAGTTCGAGCGCATGCTCATGAGCGCCGAGGAGAAGTTCCCAGGCAAGGTGCGCGCCGTGGTCAAGTTCAACGCGGCGCTGGCGCACCACATCATGGCCGGCGCCGACGTGCTCGCCGTCACCAGCCGCTTCGAGCCCTGCGGCCTCATCCAGCTGCAGGGGATGCGATACGGAACGGTACGAGAGAGAAAAAAAAACATCCTGAATCCTGACGAGAGGGACAGAGACAGATTGATTATGAATGCTTCATCGATTTGAATTGATTGATCGATGTCTCCCGCTGCGACTCTTGCAGCCCTGCGCCTGCGCGTCCACCGGTGGACTCGTCGACACCATCATCGAAGGCAAGACCGGGTTCCACATGGGCCGCCTCAGCGTCGACGTAAGCCTACCTCTGCCATGTTCTTTCTTCTTTCTTTCTGTATGTATGTATGTATGTACGAATCAGCACCGCCATTCTTGTTTCGTCGTCCTCTCTTCCCAGTGCAACGTCGTGGAGCCGGCGGACGTCAAGAAGGTGGCCACCACCTTGCAGCGCGCCATCAAGGTGGTCGGCACGCCGGCGTACGAGGAGATGGTGAGGAACTGCATGATCCAGGATCTCTCCTGGAAGGTACGTACGCCCGCCCCGCCAGAGCAGAGCGCCAAGATCGATCGACCGACCGACCACACGTACGCGCCTCGCTCCTGTCGCTGACCGTGGTTTAATTTGCGAAATGCGCAGGGCCCTGCCAAGAACTGGGAGAACGTGCTGCTCAGCCTCGGGGTCGCTGGCGGCGAGCCAGGGGTCGAAGGCGAGGAGATCGCGCCGCTCGCCAAGGAGAACGTGGCCGCGCCCTGAAGAGTTCGGCCTGCAGGGCCCCTGATCTCGCGCGTGGTGCAAAGATGTTGGGACATCTTCTTATATATGCTGTTTCGTTTATGTGATATGGACAAGTATGTGTAGCTGCTTGCTTGTGCTAGTGTAATATAGTGTAGTGGTGGCCAGTGGCACAACCTAATAAGCGCATGAACTAATTGCTTGCGTGTGTAGTTAAGTACCGATCGGTAATTTTATAT |

**Table S1 Continued**

| **Accession** | **AC(%)** | **DNA Sequence** |
| --- | --- | --- |
| SWL182 | 99.12 | CCACAACTGTTCGCGTCCTGCTGGTTCATTATCTGACCTGATTGCATTATTGCAGCTACGAGAAGCCCGTGGAAGGCCGGAAGATCAACTGGATGAAGGCCGGGATCCTCGAGGCCGACAGGGTCCTCACCGTCAGCCCCTACTACGCCGAGGAGCTCATCTCCGGCATCGCCAGGGGCTGCGAGCTCGACAACATCATGCGCCTCACCGGCATCACCGGCATCGTCAACGGCATGGACGTCAGCGAGTGGGACCCCAGCAGGGACAAGTACATCGCCGTGAAGTACGACGTGTCGACGGTGAGCTGGCTAGCTAGCTGATTCTGCTGCCTGGTCCTCCTGCTCATGCTGGTTCGGTTCTGACGCGGCAAGTGTACGTACGTGCGTGCGACGGTGGTGTGGTGTCCGGTTCAGGCCGTGGAGGCCAAGGCGCTGAACAAGGAGGCGCTGCAGGCGGAGGTCGGGCTCCCGGTGGACCGGAACATCCCGCTGGTGGCGTTCATCGGCAGGCTGGAAGAGCAGAAGGGACCCGACGTCATGGCGGCCGCCATCCCGCAGCTCATGGAGATGGTGGAGGACGTGCAGATCGTTCTGCTGGTACGTGTGCGCCGCCCGCCACCCGGCTACTACATGCGTGTATCGTTCGTTCTACTGGAACATGCGTGTGAGCAACGCGATGGATAATGCTGCAGGGCACGGGCAAGAAGAAGTTCGAGCGCATGCTCATGAGCGCCGAGGAGAAGTTCCCAGGCAAGGTGCGCGCCGTGGTCAAGTTCAACGCGGCGCTGGCGCACCACATCATGGCCGGCGCCGACGTGCTCGCCGTCACCAGCCGCTTCGAGCCCTGCGGCCTCATCCAGCTGCAGGGGATGCGATACGGAACGGTACGAGAGAGAAAAAAAAACATCCTGAATCCTGACGAGAGGGACAGAGACAGATTGATTATGAATGCTTCATCGATTTGAATTGATTGATCGATGTCTCCCGCTGCGACTCTTGCAGCCCTGCGCCTGCGCGTCCACCGGTGGACTCGTCGACACCATCATCGAAGGCAAGACCGGGTTCCACATGGGCCGCCTCAGCGTCGACGTAAGCCTACCTCTGCCATGTTCTTTCTTCTTTCTTTCTGTATGTATGTATGTATGTACGAATCAGCACCGCCATTCTTGTTTCGTCGTCCTCTCTTCCCAGTGCAACGTCGTGGAGCCGGCGGACGTCAAGAAGGTGGCCACCACCTTGCAGCGCGCCATCAAGGTGGTCGGCACGCCGGCGTACGAGGAGATGGTGAGGAACTGCATGATCCAGGATCTCTCCTGGAAGGTACGTACGCCCGCCCCGCCAGAGCAGAGCGCCAAGATCGATCGACCGACCGACCACACGTACGCGCCTCGCTCCTGTCGCTGACCGTGGTTTAATTTGCGAAATGCGCAGGGCCCTGCCAAGAACTGGGAGAACGTGCTGCTCAGCCTCGGGGTCGCTGGCGGCGAGCCAGGGGTCGAAGGCGAGGAGATCGCGCCGCTCGCCAAGGAGAACGTGGCCGCGCCCTGAAGAGTTCGGCCTGCAGGGCCCCTGATCTCGCGCGTGGTGCAAAGATGTTGGGACATCTTCTTATATATGCTGTTTCGTTTATGTGATATGGACAAGTATGTGTAGCTGCTTGCTTGTGCTAGTGTAATATAGTGTAGTGGTGGCCAGTGGCACAACCTAATAAGCGCATGAACTAATTGCTTGCGTGTGTAGTTAAGTACCGATCGGTAATTTTATAT |
| SWL183 | 98.00 | CCACAACTGTTCGCGTCCTGCTGGTTCATTATCTGACCTGATTGCATTATTGCAGCTACGAGAAGCCCGTGGAAGGCCGGAAGATCAACTGGATGAAGGCCGGGATCCTCGAGGCCGACAGGGTCCTCACCGTCAGCCCCTACTACGCCGAGGAGCTCATCTCCGGCATCGCCAGGGGCTGCGAGCTCGACAACATCATGCGCCTCACCGGCATCACCGGCATCGTCAACGGCATGGACGTCAGCGAGTGGGACCCCAGCAGGGACAAGTACATCGCCGTGAAGTACGACGTGTCGACGGTGAGCTGGCTAGCTAGCTGATTCTGCTGCCTGGTCCTCCTGCTCATGCTGGTTCGGTTCTGACGCGGCAAGTGTACGTACGTGCGTGCGACGGTGGTGTGGTGTCCGGTTCAGGCCGTGGAGGCCAAGGCGCTGAACAAGGAGGCGCTGCAGGCGGAGGTCGGGCTCCCGGTGGACCGGAACATCCCGCTGGTGGCGTTCATCGGCAGGCTGGAAGAGCAGAAGGGACCCGACGTCATGGCGGCCGCCATCCCGCAGCTCATGGAGATGGTGGAGGACGTGCAGATCGTTCTGCTGGTACGTGTGCGCCGCCCGCCACCCGGCTACTACATGCGTGTATCGTTCGTTCTACTGGAACATGCGTGTGAGCAACGCGATGGATAATGCTGCAGGGCACGGGCAAGAAGAAGTTCGAGCGCATGCTCATGAGCGCCGAGGAGAAGTTCCCAGGCAAGGTGCGCGCCGTGGTCAAGTTCAACGCGGCGCTGGCGCACCACATCATGGCCGGCGCCGACGTGCTCGCCGTCACCAGCCGCTTCGAGCCCTGCGGCCTCATCCAGCTGCAGGGGATGCGATACGGAACGGTACGAGAGAGAAAAAAAAACATCCTGAATCCTGACGAGAGGGACAGAGACAGATTGATTATGAATGCTTCATCGATTTGAATTGATTGATCGATGTCTCCCGCTGCGACTCTTGCAGCCCTGCGCCTGCGCGTCCACCGGTGGACTCGTCGACACCATCATCGAAGGCAAGACCGGGTTCCACATGGGCCGCCTCAGCGTCGACGTAAGCCTACCTCTGCCATGTTCTTTCTTCTTTCTTTCTGTATGTATGTATGTATGTACGAATCAGCACCGCCATTCTTGTTTCGTCGTCCTCTCTTCCCAGTGCAACGTCGTGGAGCCGGCGGACGTCAAGAAGGTGGCCACCACCTTGCAGCGCGCCATCAAGGTGGTCGGCACGCCGGCGTACGAGGAGATGGTGAGGAACTGCATGATCCAGGATCTCTCCTGGAAGGTACGTACGCCCGCCCCGCCAGAGCAGAGCGCCAAGATCGATCGACCGACCGACCACACGTACGCGCCTCGCTCCTGTCGCTGACCGTGGTTTAATTTGCGAAATGCGCAGGGCCCTGCCAAGAACTGGGAGAACGTGCTGCTCAGCCTCGGGGTCGCTGGCGGCGAGCCAGGGGTCGAAGGCGAGGAGATCGCGCCGCTCGCCAAGGAGAACGTGGCCGCGCCCTGAAGAGTTCGGCCTGCAGGGCCCCTGATCTCGCGCGTGGTGCAAAGATGTTGGGACATCTTCTTATATATGCTGTTTCGTTTATGTGATATGGACAAGTATGTGTAGCTGCTTGCTTGTGCTAGTGTAATATAGTGTAGTGGTGGCCAGTGGCACAACCTAATAAGCGCATGAACTAATTGCTTGCGTGTGTAGTTAAGTACCGATCGGTAATTTTTATAT |

**Table S1 Continued**

| **Accession** | **AC(%)** | **DNA Sequence** |
| --- | --- | --- |
| SWL190 | 98.85 | CCACAACTGTTCGCGTCCTGCTGGTTCATTATCTGACCTGATTGCATTATTGCAGCTACGAGAAGCCCGTGGAAGGCCGGAAGATCAACTGGATGAAGGCCGGGATCCTCGAGGCCGACAGGGTCCTCACCGTCAGCCCCTACTACGCCGAGGAGCTCATCTCCGGCATCGCCAGGGGCTGCGAGCTCGACAACATCATGCGCCTCACCGGCATCACCGGCATCGTCAACGGCATGGACGTCAGCGAGTGGGACCCCAGCAGGGACAAGTACATCGCCGTGAAGTACGACGTGTCGACGGTGAGCTGGCTAGCTAGCTGATTCTGCTGCCTGGTCCTCCTGCTCATGCTGGTTCGGTTCTGACGCGGCAAGTGTACGTACGTGCGTGCGACGGTGGTGTGGTGTCCGGTTCAGGCCGTGGAGGCCAAGGCGCTGAACAAGGAGGCGCTGCAGGCGGAGGTCGGGCTCCCGGTGGACCGGAACATCCCGCTGGTGGCGTTCATCGGCAGGCTGGAAGAGCAGAAGGGACCCGACGTCATGGCGGCCGCCATCCCGCAGCTCATGGAGATGGTGGAGGACGTGCAGATCGTTCTGCTGGTACGTGTGCGCCGCCCGCCACCCGGCTACTACATGCGTGTATCGTTCGTTCTACTGGAACATGCGTGTGAGCAACGCGATGGATAATGCTGCAGGGCACGGGCAAGAAGAAGTTCGAGCGCATGCTCATGAGCGCCGAGGAGAAGTTCCCAGGCAAGGTGCGCGCCGTGGTCAAGTTCAACGCGGCGCTGGCGCACCACATCATGGCCGGCGCCGACGTGCTCGCCGTCACCAGCCGCTTCGAGCCCTGCGGCCTCATCCAGCTGCAGGGGATGCGATACGGAACGGTACGAGAGAGAAAAAAAAACATCCTGAATCCTGACGAGAGGGACAGAGACAGATTGATTATGAATGCTTCATCGATTTGAATTGATTGATCGATGTCTCCCGCTGCGACTCTTGCAGCCCTGCGCCTGCGCGTCCACCGGTGGACTCGTCGACACCATCATCGAAGGCAAGACCGGGTTCCACATGGGCCGCCTCAGCGTCGACGTAAGCCTACCTCTGCCATGTTCTTTCTTCTTTCTTTCTGTATGTATGTATGTATGTACGAATCAGCACCGCCATTCTTGTTTCGTCGTCCTCTCTTCCCAGTGCAACGTCGTGGAGCCGGCGGACGTCAAGAAGGTGGCCACCACCTTGCAGCGCGCCATCAAGGTGGTCGGCACGCCGGCGTACGAGGAGATGGTGAGGAACTGCATGATCCAGGATCTCTCCTGGAAGGTACGTACGCCCGCCCCGCCAGAGCAGAGCGCCAAGATCGATCGACCGACCGACCACACGTACGCGCCTCGCTCCTGTCGCTGACCGTGGTTTAATTTGCGAAATGCGCAGGGCCCTGCCAAGAACTGGGAGAACGTGCTGCTCAGCCTCGGGGTCGCTGGCGGCGAGCCAGGGGTCGAAGGCGAGGAGATCGCGCCGCTCGCCAAGGAGAACGTGGCCGCGCCCTGAAGAGTTCGGCCTGCAGGGCCCCTGATCTCGCGCGTGGTGCAAAGATGTTGGGACATCTTCTTATATATGCTGTTTCGTTTATGTGATATGGACAAGTATGTGTAGCTGCTTGCTTGTGCTAGTGTAATATAGTGTAGTGGTGGCCAGTGGCACAACCTAATAAGCGCATGAACTAATTGCTTGCGTGTGTAGTTAAGTACCGATCGGTAATTTTATAT |
| SWL191 | 98.29 | CCACAACTGTTCGCGTCCTGCTGGTTCATTATCTGACCTTGATTGCATTGCAGCTACAAGAAGCCCGTGGAAGGCCGGAAGATCAACTGGATGAAGGCCGGGATCCTCGAGGCCGACAGGGTCCTCACCGTCAGCCCCTACTACGCCGAGGAGCTCATCTCCGGCATCGCCAGGGGCTGCGAGCTCGACAACATCATGCGCCTCACCGGCATCACCGGCATCGTCAACGGCATGGACGTCAGCGAGTGGGACCCCAGCAGGGACAAGTACATCTCCGTGAAGTACGACATGTCGACGGTGAGCTGGCTAGCTAGCTGATTCTGCTGCCTGGTCCTCCTGCTCATGCTGGTTCGGTTCTGACACGGCGAGTGTACGTACGTGCGTGCGACGGTGGTGTGGTGTCCGGTTCAGGCCGTGGAGGCCAAGGCGCTGCAGGCGGAGGTCGGGCTCCCGGTGGACCGGAACATCCCGCTGGTGGCGTTCATCGGCAGGCTGGAAGAGCAGAAGGGACCCGACGTCATGGCGGCCGCCATCCCGCAGCTCATGGAGATGGTGGAGGACGTGCAGATCGTTCTGCTGGTACGTGTGCGCCGCCCGCCACCCGGCTACTACATGCGTGTATCGTTCTACTGGAACATACGTGTGAGCAACGCGATGGATAATGCTGCAGGGCACGGGCAAGAAGAAGTTCGAGCGCATGCTCATGAGCGCCGAGGAGAAGTTCCCAGGCAAGGTGCGCGCCGTGGTCAAGTTCAACGCGGCGCTGGCGCACCACATCATGGCCGGCGCCGACGTGCTCGCCGTCACCAGCCGCTTCGAGCCCTGCGGCCTCATCCAGCTGCAGGGGATGCGATACGGAACGGTACGAGAGAGAAAAAAAAACATCCTGAATCCTGACGAGAGGGACAGAGACAGATTGATTATGAATGCTTCATCGATTTGAATTGATTGATCGATGTCTCCCGCTGCGACTCTTGCAGCCCTGCGCCTGCGCGTCCACCGGTGGACTCGTCGACACCATCATCGAAGGCAAGACCGGGTTCCACATGGGCCGCCTCAGCGTCGACGTAAGCCTAGCTCTGCCATGATCTTTCTTCTTTCTGTATGTATGTATGTATGAATCAGCACCGCCGTTCTTGTTTCGTCGTCCTCTCTTCCCAGTGCAACGTCGTGGAGCCGGCGGACGTCAAGAAGGTGGCCACCACCTTGCAGCGCGCCATCAAGGTGGTCGGCACGCCGGCGTACGAGGAGATGGTGAGGAACTGCATGATCCAGGATCTCTCCTGGAAGGTACGTACGCCCGCCCCGCCAGAGCAGAGCGCCAAGATCGATCGACCGACCGACCACACGTACGCGCCTCGCTCCTGTCGCTGACCGTGGTTTAATTTGCGAAATGCGCAGGGCCCTGCCAAGAACTGGGAGAACGTGCTGCTCAGCCTCGGGGTCGCCGGCGGCGAGCCAGGGGTCGAAGGCGAGGAGATCGCGCCGCTCGCCAAGGAGAACGTGGCCGCGCCCTGAAGAGTTCGGCCTGCAGGCCCCCTGATCTCGCGCGTGGTGCAAACATGTTGGGACATCTTCTTATATATGCTGTTTCGTTTATGTGATATGGACAAGTATGTGTAGCTGCTTGCTTGTGCTAGTGTAATATAATAGTGTAGTGGTGGCCAGTGGCACAACCTAATAAGCGCATGAACTAATTGCTTGCGTGTGTAGTTAAGTACCGATCGGTAATTTTATAT |

**Table S1 Continued**

| **Accession** | **AC(%)** | **DNA Sequence** |
| --- | --- | --- |
| SWL193 | 98.76 | CCACAACTGTTCGCGTCCTGCTGGTTCATTATCTGACCTGATTGCATTATTGCAGCTACGAGAAGCCCGTGGAAGGCCGGAAGATCAACTGGATGAAGGCCGGGATCCTCGAGGCCGACAGGGTCCTCACCGTCAGCCCCTACTACGCCGAGGAGCTCATCTCCGGCATCGCCAGGGGCTGCGAGCTCGACAACATCATGCGCCTCACCGGCATCACCGGCATCGTCAACGGCATGGACGTCAGCGAGTGGGACCCCAGCAGGGACAAGTACATCGCCGTGAAGTACGACGTGTCGACGGTGAGCTGGCTAGCTAGCTGATTCTGCTGCCTGGTCCTCCTGCTCATGCTGGTTCGGTTCTGACGCGGCAAGTGTACGTACGTGCGTGCGACGGTGGTGTGGTGTCCGGTTCAGGCCGTGGAGGCCAAGGCGCTGAACAAGGAGGCGCTGCAGGCGGAGGTCGGGCTCCCGGTGGACCGGAACATCCCGCTGGTGGCGTTCATCGGCAGGCTGGAAGAGCAGAAGGGACCCGACGTCATGGCGGCCGCCATCCCGCAGCTCATGGAGATGGTGGAGGACGTGCAGATCGTTCTGCTGGTACGTGTGCGCCGCCCGCCACCCGGCTACTACATGCGTGTATCGTTCGTTCTACTGGAACATGCGTGTGAGCAACGCGATGGATAATGCTGCAGGGCACGGGCAAGAAGAAGTTCGAGCGCATGCTCATGAGCGCCGAGGAGAAGTTCCCAGGCAAGGTGCGCGCCGTGGTCAAGTTCAACGCGGCGCTGGCGCACCACATCATGGCCGGCGCCGACGTGCTCGCCGTCACCAGCCGCTTCGAGCCCTGCGGCCTCATCCAGCTGCAGGGGATGCGATACGGAACGGTACGAGAGAGAAAAAAAAACATCCTGAATCCTGACGAGAGGGACAGAGACAGATTGATTATGAATGCTTCATCGATTTGAATTGATTGATCGATGTCTCCCGCTGCGACTCTTGCAGCCCTGCGCCTGCGCGTCCACCGGTGGACTCGTCGACACCATCATCGAAGGCAAGACCGGGTTCCACATGGGCCGCCTCAGCGTCGACGTAAGCCTACCTCTGCCATGTTCTTTCTTCTTTCTTTCTGTATGTATGTATGTATGTACGAATCAGCACCGCCATTCTTGTTTCGTCGTCCTCTCTTCCCAGTGCAACGTCGTGGAGCCGGCGGACGTCAAGAAGGTGGCCACCACCTTGCAGCGCGCCATCAAGGTGGTCGGCACGCCGGCGTACGAGGAGATGGTGAGGAACTGCATGATCCAGGATCTCTCCTGGAAGGTACGTACGCCCGCCCCGCCAGAGCAGAGCGCCAAGATCGATCGACCGACCGACCACACGTACGCGCCTCGCTCCTGTCGCTGACCGTGGTTTAATTTGCGAAATGCGCAGGGCCCTGCCAAGAACTGGGAGAACGTGCTGCTCAGCCTCGGGGTCGCTGGCGGCGAGCCAGGGGTCGAAGGCGAGGAGATCGCGCCGCTCGCCAAGGAGAACGTGGCCGCGCCCTGAAGAGTTCGGCCTGCAGGGCCCCTGATCTCGCGCGTGGTGCAAAGATGTTGGGACATCTTCTTATATATGCTGTTTCGTTTATGTGATATGGACAAGTATGTGTAGCTGCTTGCTTGTGCTAGTGTAATATAGTGTAGTGGTGGCCAGTGGCACAACCTAATAAGCGCATGAACTAATTGCTTGCGTGTGTAGTTAAGTACCGATCGGTAATTTTATAT |
| SWL194 | 97.55 | CCACAACTGTTCGCGTCCTGCTGGTTCATTATCTGACCTGATTGCATTATTGCAGCTACGAGAAGCCCGTGGAAGGCCGGAAGATCAACTGGATGAAGGCCGGGATCCTCGAGGCCGACAGGGTCCTCACCGTCAGCCCCTACTACGCCGAGGAGCTCATCTCCGGCATCGCCAGGGGTTGCGAGCTCGACAACATCATGCGCCTCACCGGCATCACCGGCATCGTCAACGGCATGGACGTCAGCGAGTGGGACCCCAGCAGGGACAAGTACATCGCCGTGAAGTACGACGTGTCGACGGTGAGCTGGCTAGCTAGCTGATTCTGCTGCCTGGTCCTCCTGCTCATGCTGGTTCGGTTCTGACGCGGCAAGTGTACGTACGTGCGTGCGACGGTGGTGTGGTGTCCGGTTCAGGCCGTGGAGGCCAAGGCGCTGAACAAGGAGGCGCTGCAGGCGGAGGTCGGGCTCCCGGTGGACCGGAACATCCCGCTGGTGGCGTTCATCGGCAGGCTGGAAGAGCAGAAGGGACCCGACGTCATGGCGGCCGCCATCCCGCAGCTCATGGAGATGGTGGAGGACGTGCAGATCGTTCTGCTGGTACGTGTGCGCCGCCCGCCACCCGGCTACTACATGCGTGTATCGTTCGTTCTACTGGAACATGCGTGTGAGCAACGCGATGGATAATGCTGCAGGGCACGGGCAAGAAGAAGTTCGAGCGCATGCTCATGAGCGCCGAGGAGAAGTTCCCAGGCAAGGTGCGCGCCGTGGTCAAGTTCGACGCGGCGCTGGCGCACCACATCATGGCCGGCGCCGACGTGCTCGCCGTCACCAGCCGCTTCGAGCCCTGCGGCCTCATCCAGCTGCAGGGGATGCGATACGGAACGGTACGAGAGAGAAAAAAAAACATCCTGAATCCTGACGAGAGGGACAGAGACAGATTGATTATGAATGCTTCATCGATTTGAATTGATTGATCGATGTCTCCCGCTGCGACTCTTGCAGCCCTGCGCCTGCGCGTCCACCGGTGGACTCGTCGACACCATCATCGAAGGCAAGACCGGGTTCCACATGGGCCGCCTCAGCGTCGACGTAAGCCTACCTCTGCCATGTTCTTTCTTCTTTCTTTCTGTATGTATGTATGTATGTACGAATCAGCACCGCCATTCTTGTTTCGTCGTCCTCTCTTCCCAGTGCAACGTCGTGGAGCCGGCGGACGTCAAGAAGGTGGCCACCACCTTGCAGCGCGCCATCAAGGTGGTCGGCACGCCGGCGTACGAGGAGATGGTGAGGAACTGCATGATCCAGGATCTCTCCTGGAAGGTACGTACGCCCGCCCCGCCAGAGCAGAGCGCCAAGATCGATCGACCGACCGACCACACGTACGCGCCTCGCTCCTGTCGCTGACCGTGGTTTAATTTGCGAAATGCGCAGGGCCCTGCCAAGAACTGGGAGAACGTGCTGCTCAGCCTCGGGGTCGCTGGCGGCGAGCCAGGGGTCGAAGGCGAGGAGATCGCGCCGCTCGCCAAGGAGAACGTGGCCGCGCCCTGAAGAGTTCGGCCTGCAGGGCCCCTGATCTCGCGCGTGGTGCAAAGATGTTGGGACATCTTCTTATATATGCTGTTTCGTTTATGTGATATGGACAAGTATGTGTAGCTGCTTGCTTGTGCTAGTGTAATATAGTGTAGTGGTGGCCAGTGGCACAACCTAATAAGCGCATGAACTAATTGCTTGCGTGTGTAGTTAAGTACCGATCGGTAATTTTATAT |

**Table S1 Continued**

| **Accession** | **AC(%)** | **DNA Sequence** |
| --- | --- | --- |
| SWL196 | 98.58 | CCACAACTGTTCGCGTCCTGCTGGTTCATTATCTGACCTGATTGCATTATTGCAGCTACGAGAAGCCCGTGGAAGGCCGGAAGATCAACTGGATGAAGGCCGGGATCCTCGAGGCCGACAGGGTCCTCACCGTCAGCCCCTACTACGCCGAGGAGCTCATCTCCGGCATCGCCAGGGGCTGCGAGCTCGACAACATCATGCGCCTCACCGGCATCACCGGCATCGTCAACGGCATGGACGTCAGCGAGTGGGACCCCAGCAGGGACAAGTACATCGCCGTGAAGTACGACGTGTCGACGGTGAGCTGGCTAGCTAGCTGATTCTGCTGCCTGGTCCTCCTGCTCATGCTGGTTCGGTTCTGACGCGGCAAGTGTACGTACGTGCGTGCGACGGTGGTGTGGTGTCCGGTTCAGGCCGTGGAGGCCAAGGCGCTGAACAAGGAGGCGCTGCAGGCGGAGGTCGGGCTCCCGGTGGACCGGAACATCCCGCTGGTGGCGTTCATCGGCAGGCTGGAAGAGCAGAAGGGACCCGACGTCATGGCGGCCGCCATCCCGCAGCTCATGGAGATGGTGGAGGACGTGCAGATCGTTCTGCTGGTACGTGTGCGCCGCCCGCCACCCGGCTACTACATGCGTGTATCGTTCGTTCTACTGGAACATGCGTGTGAGCAACGCGATGGATAATGCTGCAGGGCACGGGCAAGAAGAAGTTCGAGCGCATGCTCATGAGCGCCGAGGAGAAGTTCCCAGGCAAGGTGCGCGCCGTGGTCAAGTTCAACGCGGCGCTGGCGCACCACATCATGGCCGGCGCCGACGTGCTCGCCGTCACCAGCCGCTTCGAGCCCTGCGGCCTCATCCAGCTGCAGGGGATGCGATACGGAACGGTACGAGAGAGAAAAAAAAACATCCTGAATCCTGACGAGAGGGACAGAGACAGATTGATTATGAATGCTTCATCGATTTGAATTGATTGATCGATGTCTCCCGCTGCGACTCTTGCAGCCCTGCGCCTGCGCGTCCACCGGTGGACTCGTCGACACCATCATCGAAGGCAAGACCGGGTTCCACATGGGCCGCCTCAGCGTCGACGTAAGCCTACCTCTGCCATGTTCTTTCTTCTTTCTTTCTGTATGTATGTATGTATGTACGAATCAGCACCGCCATTCTTGTTTCGTCGTCCTCTCTTCCCAGTGCAACGTCGTGGAGCCGGCGGACGTCAAGAAGGTGGCCACCACCTTGCAGCGCGCCATCAAGGTGGTCGGCACGCCGGCGTACGAGGAGATGGTGAGGAACTGCATGATCCAGGATCTCTCCTGGAAGGTACGTACGCCCGCCCCGCCAGAGCAGAGCGCCAAGATCGATCGACCGACCGACCACACGTACGCGCCTCGCTCCTGTCGCTGACCGTGGTTTAATTTGCGAAATGCGCAGGGCCCTGCCAAGAACTGGGAGAACGTGCTGCTCAGCCTCGGGGTCGCTGGCGGCGAGCCAGGGGTCGAAGGCGAGGAGATCGCGCCGCTCGCCAAGGAGAACGTGGCCGCGCCCTGAAGAGTTCGGCCTGCAGGGCCCCTGATCTCGCGCGTGGTGCAAAGATGTTGGGACATCTTCTTATATATGCTGTTTCGTTTATGTGATATGGACAAGTATGTGTAGCTGCTTGCTTGTGCTAGTGTAATATAGTGTAGTGGTGGCCAGTGGCACAACCTAATAAGCGCATGAACTAATTGCTTGCGTGTGTAGTTAAGTACCGATCGGTAATTTTATAT |
| SWL197 | 98.75 | CCACAACTGTTCGCGTCCTGCTGGTTCATTATCTGACCTTGATTGCATTGCAGCTACGAGAAGCCCGTGGAAGGCCGGAAGATCAACTGGATGAAGGCCGGGATCCTCGAGGCCGACAGGGTCCTCACCGTCAGCCCCTACTACGCCGAGGAGCTCATCTCCGGCATCGCCAGGGGCTGCGAGCTCGACAACATCATGCGCCTCACCGGCATCACCGGCATCGTCAACGGCATGGACGTCAGCGAGTGGGACCCCAGCAGGGACAAGTACATCGCCGTGAAGTACGACGTGTCGACGGTGAGCTGGCTAGCTAGCTGATTCTGCTGCCTGGTCCTCCTGCTCATGCTGGTTCGGTTCTGACGCGGCGAGTGTACGTACGTGCGTGCGACGGTGGTGTGGTGTCCGGTTCAGGCCGTGGAGGCCAAGGCGCTGCAGGCGGAGGTCGGGCTCCCGGTGGACCGGAACATCCCGCTGGTGGCGTTCATCGGCAGGCTGGAAGAGCAGAAGGGACCCGACGTCATGGCGGCCGCCATCCCGCAGCTCATGGAGATGGTGGAGGACGTGCAGATCGTTCTGCTGGTACGTGTGCGCCGCCCGCCACCCGGCTACTACATGCGTGTATCGTTCTACTGGAACATACGTGTGAGCAACGCGATGGATAATGCTGCAGGGCACGGGCAAGAAGAAGTTCGAGCGCATGCTCATGAGCGCCGAGGAGAAGTTCCCAGGCAAGGTGCGCGCCGTGGTCAAGTTCAACGCGGCGCTGGCGCACCACATCATGGCCGGCGCCGACGTGCTCGCCGTCACCAGCCGCTTCGAGCCCTGCGGCCTCATCCAGCTGCAGGGGATGCGATACGGAACGGTACGAGAGAGAAAAAAAAACATCCTGAATCCTGACGAGAGGGACAGAGACAGATTGATTATGAATGCTTCATCGATTTGAATTGATTGATCGATGTCTCCCGCTGCGACTCTTGCAGCCCTGCGCCTGCGCGTCCACCGGTGGACTCGTCGACACCATCATCGAAGGCAAGACCGGGTTCCACATGGGCCGCCTCAGCGTCGACGTAAGCCTAGCTCTGCCATGATCTTTCTTCTTTCTGTATGTATGTATGTATGAATCAGCACCGCCGTTCTTGTTTCGTCGTCCTCTCTTCCCAGTGCAACGTCGTGGAGCCGGCGGACGTCAAGAAGGTGGCCACCACCTTGCAGCGCGCCATCAAGGTGGTCGGCACGCCGGCGTACGAGGAGATGGTGAGGAACTGCATGATCCAGGATCTCTCCTGGAAGGTACGTACGCCCGCCCCGCCAGAGCAGAGCGCCAAGATCGATCGACCGACCGACCACACGTACGCGCCTCGCTCCTGTCGCTGACCGTGGTTTAATTTGCGAAATGCGCAGGGCCCTGCCAAGAACTGGGAGAACGTGCTGCTCAGCCTCGGGGTCGCCGGCGGCGAGCCAGGGGTCGAAGGCGAGGAGATCGCGCCGCTCGCCAAGGAGAACGTGGCCGCGCCCTGAAGAGTTCGGCCTGCAGGCCCCCTGATCTCGCGCGTGGTGCAAACATGTTGGGACATCTTCTTATATATGCTGTTTCGTTTATGTGATATGGACAAGTATGTGTAGCTGCTTGCTTGTGCTAGTGTAATATAATAGTGTAGTGGTGGCCAGTGGCACAACCTAATAAGCGCATGAACTAATTGCTTGCGTGTGTAGTTAAGTACCGATCGGTAATTTTATAT |

**Table S1 Continued**

| **Accession** | **AC(%)** | **DNA Sequence** |
| --- | --- | --- |
| SWL198 | 97.80 | CCACAACTGTTCGCGTCCTGCTGGTTCATTATCTGACCTGATTGCATTATTGCAGCTACGAGAAGCCCGTGGAAGGCCGGAAGATCAACTGGATGAAGGCCGGGATCCTCGAGGCCGACAGGGTCCTCACCGTCAGCCCCTACTACGCCGAGGAGCTCATCTCCGGCATCGCCAGGGGCTGCGAGCTCGACAACATCATGCGCCTCACCGGCATCACCGGCATCGTCAACGGCATGGACGTCAGCGAGTGGGACCCCAGCAGGGACAAGTACATCGCCGTGAAGTACGACGTGTCGACGGTGAGCTGGCTAGCTAGCTGATTCTGCTGCCTGGTCCTCCTGCTCATGCTGGTTCGGTTCTGACGCGGCAAGTGTACGTACGTGCGTGCGACGGTGGTGTGGTGTCCGGTTCAGGCCGTGGAGGCCAAGGCGCTGAACAAGGAGGCGCTGCAGGCGGAGGTCGGGCTCCCGGTGGACCGGAACATCCCGCTGGTGGCGTTCATCGGCAGGCTGGAAGAGCAGAAGGGACCCGACGTCATGGCGGCCGCCATCCCGCAGCTCATGGAGATGGTGGAGGACGTGCAGATCGTTCTGCTGGTACGTGTGCGCCGCCCGCCACCCGGCTACTACATGCGTGTATCGTTCGTTCTACTGGAACATGCGTGTGAGCAACGCGATGGATAATGCTGCAGGGCACGGGCAAGAAGAAGTTCGAGCGCATGCTCATGAGCGCCGAGGAGAAGTTCCCAGGCAAGGTGCGCGCCGTGGTCAAGTTCAACGCGGCGCTGGCGCACCACATCATGGCCGGCGCCGACGTGCTCGCCGTCACCAGCCGCTTCGAGCCCTGCGGCCTCATCCAGCTGCAGGGGATGCGATACGGAACGGTACGAGAGAGAAAAAAAAACATCCTGAATCCTGACGAGAGGGACAGAGACAGATTGATTATGAATGCTTCATCGATTTGAATTGATTGATCGATGTCTCCCGCTGCGACTCTTGCAGCCCTGCGCCTGCGCGTCCACCGGTGGACTCGTCGACACCATCATCGAAGGCAAGACCGGGTTCCACATGGGCCGCCTCAGCGTCGACGTAAGCCTACCTCTGCCATGTTCTTTCTTCTTTCTTTCTGTATGTATGTATGTATGTACGAATCAGCACCGCCATTCTTGTTTCGTCGTCCTCTCTTCCCAGTGCAACGTCGTGGAGCCGGCGGACGTCAAGAAGGTGGCCACCACCTTGCAGCGCGCCATCAAGGTGGTCGGCACGCCGGCGTACGAGGAGATGGTGAGGAACTGCATGATCCAGGATCTCTCCTGGAAGGTACGTACGCCCGCCCCGCCAGAGCAGAGCGCCAAGATCGATCGACCGACCGACCACACGTACGCGCCTCGCTCCTGTCGCTGACCGTGGTTTAATTTGCGAAATGCGCAGGGCCCTGCCAAGAACTGGGAGAACGTGCTGCTCAGCCTCGGGGTCGCTGGCGGCGAGCCAGGGGTCGAAGGCGAGGAGATCGCGCCGCTCGCCAAGGAGAACGTGGCCGCGCCCTGAAGAGTTCGGCCTGCAGGGCCCCTGATCTCGCGCGTGGTGCAAAGATGTTGGGACATCTTCTTATATATGCTGTTTCGTTTATGTGATATGGACAAGTATGTGTAGCTGCTTGCTTGTGCTAGTGTAATATAGTGTAGTGGTGGCCAGTGGCACAACCTAATAAGCGCATGAACTAATTGCTTGCGTGTGTAGTTAAGTACCGATCGGTAATTTTATAT |
| SWL200 | 98.15 | CCACAACTGTTCGCGTCCTGCTGGTTCATTATCTGACCTGATTGCATTATTGCAGCTACGAGAAGCCCGTGGAAGGCCGGAAGATCAACTGGATGAAGGCCGGGATCCTCGAGGCCGACAGGGTCCTCACCGTCAGCCCCTACTACGCCGAGGAGCTCATCTCCGGCATCGCCAGGGGCTGCGAGCTCGACAACATCATGCGCCTCACCGGCATCACCGGCATCGTCAACGGCATGGACGTCAGCGAGTGGGACCCCAGCAGGGACAAGTACATCGCCGTGAAGTACGACGTGTCGACGGTGAGCTGGCTAGCTAGCTGATTCTGCTGCCTGGTCCTCCTGCTCATGCTGGTTCGGTTCTGACGCGGCAAGTGTACGTACGTGCGTGCGACGGTGGTGTGGTGTCCGGTTCAGGCCGTGGAGGCCAAGGCGCTGAACAAGGAGGCGCTGCAGGCGGAGGTCGGGCTCCCGGTGGACCGGAACATCCCGCTGGTGGCGTTCATCGGCAGGCTGGAAGAGCAGAAGGGACCCGACGTCATGGCGGCCGCCATCCCGCAGCTCATGGAGATGGTGGAGGACGTGCAGATCGTTCTGCTGGTACGTGTGCGCCGCCCGCCACCCGGCTACTACATGCGTGTATCGTTCGTTCTACTGGAACATGCGTGTGAGCAACGCGATGGATAATGCTGCAGGGCACGGGCAAGAAGAAGTTCGAGCGCATGCTCATGAGCGCCGAGGAGAAGTTCCCAGGCAAGGTGCGCGCCGTGGTCAAGTTCAACGCGGCGCTGGCGCACCACATCATGGCCGGCGCCGACGTGCTCGCCGTCACCAGCCGCTTCGAGCCCTGCGGCCTCATCCAGCTGCAGGGGATGCGATACGGAACGGTACGAGAGAGAAAAAAAAACATCCTGAATCCTGACGAGAGGGACAGAGACAGATTGATTATGAATGCTTCATCGATTTGAATTGATTGATCGATGTCTCCCGCTGCGACTCTTGCAGCCCTGCGCCTGCGCGTCCACCGGTGGACTCGTCGACACCATCATCGAAGGCAAGACCGGGTTCCACATGGGCCGCCTCAGCGTCGACGTAAGCCTACCTCTGCCATGTTCTTTCTTCTTTCTTTCTGTATGTATGTATGTATGTACGAATCAGCACCGCCATTCTTGTTTCGTCGTCCTCTCTTCCCAGTGCAACGTCGTGGAGCCGGCGGACGTCAAGAAGGTGGCCACCACCTTGCAGCGCGCCATCAAGGTGGTCGGCACGCCGGCGTACGAGGAGATGGTGAGGAACTGCATGATCCAGGATCTCTCCTGGAAGGTACGTACGCCCGCCCCGCCAGAGCAGAGCGCCAAGATCGATCGACCGACCGACCACACGTACGCGCCTCGCTCCTGTCGCTGACCGTGGTTTAATTTGCGAAATGCGCAGGGCCCTGCCAAGAACTGGGAGAACGTGCTGCTCAGCCTCGGGGTCGCTGGCGGCGAGCCAGGGGTCGAAGGCGAGGAGATCGCGCCGCTCGCCAAGGAGAACGTGGCCGCGCCCTGAAGAGTTCGGCCTGCAGGGCCCCTGATCTCGCGCGTGGTGCAAAGATGTTGGGACATCTTCTTATATATGCTGTTTCGTTTATGTGATATGGACAAGTATGTGTAGCTGCTTGCTTGTGCTAGTGTAATATAGTGTAGTGGTGGCCAGTGGCACAACCTAATAAGCGCATGAACTAATTGCTTGCGTGTGTAGTTAAGTACCGATCGGTAATTTTATAT |

**Table S1 Continued**

| **Accession** | **AC(%)** | **DNA Sequence** |
| --- | --- | --- |
| SWL201 | 98.58 | CCACAACTGTTCGCGTCCTGCTGGTTCATTATCTGACCTGATTGCATTATTGCAGCTACGAGAAGCCCGTGGAAGGCCGGAAGATCAACTGGATGAAGGCCGGGATCCTCGAGGCCGACAGGGTCCTCACCGTCAGCCCCTACTACGCCGAGGAGCTCATCTCCGGCATCGCCAGGGGCTGCGAGCTCGACAACATCATGCGCCTCACCGGCATCACCGGCATCGTCAACGGCATGGACGTCAGCGAGTGGGACCCCAGCAGGGACAAGTACATCGCCGTGAAGTACGACGTGTCGACGGTGAGCTGGCTAGCTAGCTGATTCTGCTGCCTGGTCCTCCTGCTCATGCTGGTTCGGTTCTGACGCGGCAAGTGTACGTACGTGCGTGCGACGGTGGTGTGGTGTCCGGTTCAGGCCGTGGAGGCCAAGGCGCTGAACAAGGAGGCGCTGCAGGCGGAGGTCGGGCTCCCGGTGGACCGGAACATCCCGCTGGTGGCGTTCATCGGCAGGCTGGAAGAGCAGAAGGGACCCGACGTCATGGCGGCCGCCATCCCGCAGCTCATGGAGATGGTGGAGGACGTGCAGATCGTTCTGCTGGTACGTGTGCGCCGCCCGCCACCCGGCTACTACATGCGTGTATCGTTCGTTCTACTGGAACATGCGTGTGAGCAACGCGATGGATAATGCTGCAGGGCACGGGCAAGAAGAAGTTCGAGCGCATGCTCATGAGCGCCGAGGAGAAGTTCCCAGGCAAGGTGCGCGCCGTGGTCAAGTTCAACGCGGCGCTGGCGCACCACATCATGGCCGGCGCCGACGTGCTCGCCGTCACCAGCCGCTTCGAGCCCTGCGGCCTCATCCAGCTGCAGGGGATGCGATACGGAACGGTACGAGAGAGAAAAAAAAACATCCTGAATCCTGACGAGAGGGACAGAGACAGATTGATTATGAATGCTTCATCGATTTGAATTGATTGATCGATGTCTCCCGCTGCGACTCTTGCAGCCCTGCGCCTGCGCGTCCACCGGTGGACTCGTCGACACCATCATCGAAGGCAAGACCGGGTTCCACATGGGCCGCCTCAGCGTCGACGTAAGCCTACCTCTGCCATGTTCTTTCTTCTTTCTTTCTGTATGTATGTATGTATGTACGAATCAGCACCGCCATTCTTGTTTCGTCGTCCTCTCTTCCCAGTGCAACGTCGTGGAGCCGGCGGACGTCAAGAAGGTGGCCACCACCTTGCAGCGCGCCATCAAGGTGGTCGGCACGCCGGCGTACGAGGAGATGGTGAGGAACTGCATGATCCAGGATCTCTCCTGGAAGGTACGTACGCCCGCCCCGCCAGAGCAGAGCGCCAAGATCGATCGACCGACCGACCACACGTACGCGCCTCGCTCCTGTCGCTGACCGTGGTTTAATTTGCGAAATGCGCAGGGCCCTGCCAAGAACTGGGAGAACGTGCTGCTCAGCCTCGGGGTCGCTGGCGGCGAGCCAGGGGTCGAAGGCGAGGAGATCGCGCCGCTCGCCAAGGAGAACGTGGCCGCGCCCTGAAGAGTTCGGCCTGCAGGGCCCCTGATCTCGCGCGTGGTGCAAAGATGTTGGGACATCTTCTTATATATGCTGTTTCGTTTATGTGATATGGACAAGTATGTGTAGCTGCTTGCTTGTGCTAGTGTAATATAGTGTAGTGGTGGCCAGTGGCACAACCTAATAAGCGCATGAACTAATTGCTTGCGTGTGTAGTTAAGTACCGATCGGTAATTTTATAT |
| SWL202 | 97.83 | CCACAACTGTTCGCGTCCTGCTGGTTCATTATCTGACCTGATTGCATTATTGCAGCTACGAGAAGCCCGTGGAAGGCCGGAAGATCAACTGGATGAAGGCCGGGATCCTCGAGGCCGACAGGGTCCTCACCGTCAGCCCCTACTACGCCGAGGAGCTCATCTCCGGCATCGCCAGGGGCTGCGAGCTCGACAACATCATGCGCCTCACCGGCATCACCGGCATCGTCAACGGCATGGACGTCAGCGAGTGGGACCCCAGCAGGGACAAGTACATCGCCGTGAAGTACGACGTGTCGACGGTGAGCTGGCTAGCTAGCTGATTCTGCTGCCTGGTCCTCCTGCTCATGCTGGTTCGGTTCTGACGCGGCAAGTGTACGTACGTGCGTGCGACGGTGGTGTGGTGTCCGGTTCAGGCCGTGGAGGCCAAGGCGCTGAACAAGGAGGCGCTGCAGGCGGAGGTCGGGCTCCCGGTGGACCGGAACATCCCGCTGGTGGCGTTCATCGGCAGGCTGGAAGAGCAGAAGGGACCCGACGTCATGGCGGCCGCCATCCCGCAGCTCATGGAGATGGTGGAGGACGTGCAGATCGTTCTGCTGGTACGTGTGCGCCGCCCGCCACCCGGCTACTACATGCGTGTATCGTTCGTTCTACTGGAACATGCGTGTGAGCAACGCGATGGATAATGCTGCAGGGCACGGGCAAGAAGAAGTTCGAGCGCATGCTCATGAGCGCCGAGGAGAAGTTCCCAGGCAAGGTGCGCGCCGTGGTCAAGTTCAACGCGGCGCTGGCGCACCACATCATGGCCGGCGCCGACGTGCTCGCCGTCACCAGCCGCTTCGAGCCCTGCGGCCTCATCCAGCTGCAGGGGATGCGATACGGAACGGTACGAGAGAGAAAAAAAAACATCCTGAATCCTGACGAGAGGGACAGAGACAGATTGATTATGAATGCTTCATCGATTTGAATTGATTGATCGATGTCTCCCGCTGCGACTCTTGCAGCCCTGCGCCTGCGCGTCCACCGGTGGACTCGTCGACACCATCATCGAAGGCAAGACCGGGTTCCACATGGGCCGCCTCAGCGTCGACGTAAGCCTACCTCTGCCATGTTCTTTCTTCTTTCTTTCTGTATGTATGTATGTATGTACGAATCAGCACCGCCATTCTTGTTTCGTCGTCCTCTCTTCCCAGTGCAACGTCGTGGAGCCGGCGGACGTCAAGAAGGTGGCCACCACCTTGCAGCGCGCCATCAAGGTGGTCGGCACGCCGGCGTACGAGGAGATGGTGAGGAACTGCATGATCCAGGATCTCTCCTGGAAGGTACGTACGCCCGCCCCGCCAGAGCAGAGCGCCAAGATCGATCGACCGACCGACCACACGTACGCGCCTCGCTCCTGTCGCTGACCGTGGTTTAATTTGCGAAATGCGCAGGGCCCTGCCAAGAACTGGGAGAACGTGCTGCTCAGCCTCGGGGTCGCTGGCGGCGAGCCAGGGGTCGAAGGCGAGGAGATCGCGCCGCTCGCCAAGGAGAACGTGGCCGCGCCCTGAAGAGTTCGGCCTGCAGGGCCCCTGATCTCGCGCGTGGTGCAAAGATGTTGGGACATCTTCTTATATATGCTGTTTCGTTTATGTGATATGGACAAGTATGTGTAGCTGCTTGCTTGTGCTAGTGTAATATAGTGTAGTGGTGGCCAGTGGCACAACCTAATAAGCGCATGAACTAATTGCTTGCGTGTGTAGTTAAGTACCGATCGGTAATTTTATAT |

**Table S1 Continued**

| **Accession** | **AC(%)** | **DNA Sequence** |
| --- | --- | --- |
| SWL231 | 98.05 | CCACAACTGTTCGCGTCCTGCTGGTTCATTATCTGACCTGATTGCATTATTGCAGCTACGAGAAGCCCGTGGAAGGCCGGAAGATCAACTGGATGAAGGCCGGGATCCTCGAGGCCGACAGGGTCCTCACCGTCAGCCCCTACTACGCCGAGGAGCTCATCTCCGGCATCGCCAGGGGCTGCGAGCTCGACAACATCATGCGCCTCACCGGCATCACCGGCATCGTCAACGGCATGGACGTCAGCGAGTGGGACCCCAGCAGGGACAAGTACATCGCCGTGAAGTACGACGTGTCGACGGTGAGCTGGCTAGCTAGCTGATTCTGCTGCCTGGTCCTCCTGCTCATGCTGGTTCGGTTCTGACGCGGCAAGTGTACGTACGTGCGTGCGACGGTGGTGTGGTGTCCGGTTCAGGCCGTGGAGGCCAAGGCGCTGAACAAGGAGGCGCTGCAGGCGGAGGTCGGGCTCCCGGTGGACCGGAACATCCCGCTGGTGGCGTTCATCGGCAGGCTGGAAGAGCAGAAGGGACCCGACGTCATGGCGGCCGCCATCCCGCAGCTCATGGAGATGGTGGAGGACGTGCAGATCGTTCTGCTGGTACGTGTGCGCCGCCCGCCACCCGGCTACTACATGCGTGTATCGTTCGTTCTACTGGAACATGCGTGTGAGCAACGCGATGGATAATGCTGCAGGGCACGGGCAAGAAGAAGTTCGAGCGCATGCTCATGAGCGCCGAGGAGAAGTTCCCAGGCAAGGTGCGCGCCGTGGTCAAGTTCAACGCGGCGCTGGCGCACCACATCATGGCCGGCGCCGACGTGCTCGCCGTCACCAGCCGCTTCGAGCCCTGCGGCCTCATCCAGCTGCAGGGGATGCGATACGGAACGGTACGAGAGAGAAAAAAAAACATCCTGAATCCTGACGAGAGGGACAGAGACAGATTGATTATGAATGCTTCATCGATTTGAATTGATTGATCGATGTCTCCCGCTGCGACTCTTGCAGCCCTGCGCCTGCGCGTCCACCGGTGGACTCGTCGACACCATCATCGAAGGCAAGACCGGGTTCCACATGGGCCGCCTCAGCGTCGACGTAAGCCTACCTCTGCCATGTTCTTTCTTCTTTCTTTCTGTATGTATGTATGTATGTACGAATCAGCACCGCCATTCTTGTTTCGTCGTCCTCTCTTCCCAGTGCAACGTCGTGGAGCCGGCGGACGTCAAGAAGGTGGCCACCACCTTGCAGCGCGCCATCAAGGTGGTCGGCACGCCGGCGTACGAGGAGATGGTGAGGAACTGCATGATCCAGGATCTCTCCTGGAAGGTACGTACGCCCGCCCCGCCAGAGCAGAGCGCCAAGATCGATCGACCGACCGACCACACGTACGCGCCTCGCTCCTGTCGCTGACCGTGGTTTAATTTGCGAAATGCGCAGGGCCCTGCCAAGAACTGGGAGAACGTGCTGCTCAGCCTCGGGGTCGCTGGCGGCGAGCCAGGGGTCGAAGGCGAGGAGATCGCGCCGCTCGCCAAGGAGAACGTGGCCGCGCCCTGAAGAGTTCGGCCTGCAGGGCCCCTGATCTCGCGCGTGGTGCAAAGATGTTGGGACATCTTCTTATATATGCTGTTTCGTTTATGTGATATGGACAAGTATGTGTAGCTGCTTGCTTGTGCTAGTGTAATATAGTGTAGTGGTGGCCAGTGGCACAACCTAATAAGCGCATGAACTAATTGCTTGCGTGTGTAGTTAAGTACCGATCGGTAATTTTTATAT |
| SWL258 | 97.86 | CCACAACTGTTCGCGTCCTGCTGGTTCATTATCTGACCTGATTGCATTATTGCAGCTACGAGAAGCCCGTGGAAGGCCGGAAGATCAACTGGATGAAGGCCGGGATCCTCGAGGCCGACAGGGTCCTCACCGTCAGCCCCTACTACGCCGAGGAGCTCATCTCCGGCATCGCCAGGGGCTGCGAGCTCGACAACATCATGCGCCTCACCGGCATCACCGGCATCGTCAACGGCATGGACGTCAGCGAGTGGGACCCCAGCAGGGACAAGTACATCGCCGTGAAGTACGACGTGTCGACGGTGAGCTGGCTAGCTAGCTGATTCTGCTGCCTGGTCCTCCTGCTCATGCTGGTTCGGTTCTGACGCGGCAAGTGTACGTACGTGCGTGCGACGGTGGTGTGGTGTCCGGTTCAGGCCGTGGAGGCCAAGGCGCTGAACAAGGAGGCGCTGCAGGCGGAGGTCGGGCTCCCGGTGGACCGGAACATCCCGCTGGTGGCGTTCATCGGCAGGCTGGAAGAGCAGAAGGGACCCGACGTCATGGCGGCCGCCATCCCGCAGCTCATGGAGATGGTGGAGGACGTGCAGATCGTTCTGCTGGTACGTGTGCGCCGCCCGCCACCCGGCTACTACATGCGTGTATCGTTCGTTCTACTGGAACATGCGTGTGAGCAACGCGATGGATAATGCTGCAGGGCACGGGCAAGAAGAAGTTCGAGCGCATGCTCATGAGCGCCGAGGAGAAGTTCCCAGGCAAGGTGCGCGCCGTGGTCAAGTTCAACGCGGCGCTGGCGCACCACATCATGGCCGGCGCCGACGTGCTCGCCGTCACCAGCCGCTTCGAGCCCTGCGGCCTCATCCAGCTGCAGGGGATGCGATACGGAACGGTACGAGAGAGAAAAAAAAACATCCTGAATCCTGACGAGAGGGACAGAGACAGATTGATTATGAATGCTTCATCGATTTGAATTGATTGATCGATGTCTCCCGCTGCGACTCTTGCAGCCCTGCGCCTGCGCGTCCACCGGTGGACTCGTCGACACCATCATCGAAGGCAAGACCGGGTTCCACATGGGCCGCCTCAGCGTCGACGTAAGCCTACCTCTGCCATGTTCTTTCTTCTTTCTTTCTGTATGTATGTATGTATGTACGAATCAGCACCGCCATTCTTGTTTCGTCGTCCTCTCTTCCCAGTGCAACGTCGTGGAGCCGGCGGACGTCAAGAAGGTGGCCACCACCTTGCAGCGCGCCATCAAGGTGGTCGGCACGCCGGCGTACGAGGAGATGGTGAGGAACTGCATGATCCAGGATCTCTCCTGGAAGGTACGTACGCCCGCCCCGCCAGAGCAGAGCGCCAAGATCGATCGACCGACCGACCACACGTACGCGCCTCGCTCCTGTCGCTGACCGTGGTTTAATTTGCGAAATGCGCAGGGCCCTGCCAAGAACTGGGAGAACGTGCTGCTCAGCCTCGGGGTCGCTGGCGGCGAGCCAGGGGTCGAAGGCGAGGAGATCGCGCCGCTCGCCAAGGAGAACGTGGCCGCGCCCTGAAGAGTTCGGCCTGCAGGGCCCCTGATCTCGCGCGTGGTGCAAAGATGTTGGGACATCTTCTTATATATGCTGTTTCGTTTATGTGATATGGACAAGTATGTGTAGCTGCTTGCTTGTGCTAGTGTAATATAGTGTAGTGGTGGCCAGTGGCACAACCTAATAAGCGCATGAACTAATTGCTTGCGTGTGTAGTTAAGTACCGATCGGTAATTTTATAT |

**Table S1 Continued**

| **Accession** | **AC(%)** | **DNA Sequence** |
| --- | --- | --- |
| SWL269 | 98.35 | CCACAACTGTTCGCGTCCTGCTGGTTCATTATCTGACCTGATTGCATTATTGCAGCTACGAGAAGCCCGTGGAAGGCCGGAAGATCAACTGGATGAAGGCCGGGATCCTCGAGGCCGACAGGGTCCTCACCGTCAGCCCCTACTACGCCGAGGAGCTCATCTCCGGCATCGCCAGGGGCTGCGAGCTCGACAACATCATGCGCCTCACCGGCATCACCGGCATCGTCAACGGCATGGACGTCAGCGAGTGGGACCCCAGCAGGGACAAGTACATCGCCGTGAAGTACGACGTGTCGACGGTGAGCTGGCTAGCTAGCTGATTCTGCTGCCTGGTCCTCCTGCTCATGCTGGTTCGGTTCTGACGCGGCAAGTGTACGTACGTGCGTGCGACGGTGGTGTGGTGTCCGGTTCAGGCCGTGGAGGCCAAGGCGCTGAACAAGGAGGCGCTGCAGGCGGAGGTCGGGCTCCCGGTGGACCGGAACATCCCGCTGGTGGCGTTCATCGGCAGGCTGGAAGAGCAGAAGGGACCCGACGTCATGGCGGCCGCCATCCCGCAGCTCATGGAGATGGTGGAGGACGTGCAGATCGTTCTGCTGGTACGTGTGCGCCGCCCGCCACCCGGCTACTACATGCGTGTATCGTTCGTTCTACTGGAACATGCGTGTGAGCAACGCGATGGATAATGCTGCAGGGCACGGGCAAGAAGAAGTTCGAGCGCATGCTCATGAGCGCCGAGGAGAAGTTCCCAGGCAAGGTGCGCGCCGTGGTCAAGTTCAACGCGGCGCTGGCGCACCACATCATGGCCGGCGCCGACGTGCTCGCCGTCACCAGCCGCTTCGAGCCCTGCGGCCTCATCCAGCTGCAGGGGATGCGATACGGAACGGTACGAGAGAGAAAAAAAAACATCCTGAATCCTGACGAGAGGGACAGAGACAGATTGATTATGAATGCTTCATCGATTTGAATTGATTGATCGATGTCTCCCGCTGCGACTCTTGCAGCCCTGCGCCTGCGCGTCCACCGGTGGACTCGTCGACACCATCATCGAAGGCAAGACCGGGTTCCACATGGGCCGCCTCAGCGTCGACGTAAGCCTACCTCTGCCATGTTCTTTCTTCTTTCTTTCTGTATGTATGTATGTATGTACGAATCAGCACCGCCATTCTTGTTTCGTCGTCCTCTCTTCCCAGTGCAACGTCGTGGAGCCGGCGGACGTCAAGAAGGTGGCCACCACCTTGCAGCGCGCCATCAAGGTGGTCGGCACGCCGGCGTACGAGGAGATGGTGAGGAACTGCATGATCCAGGATCTCTCCTGGAAGGTACGTACGCCCGCCCCGCCAGAGCAGAGCGCCAAGATCGATCGACCGACCGACCACACGTACGCGCCTCGCTCCTGTCGCTGACCGTGGTTTAATTTGCGAAATGCGCAGGGCCCTGCCAAGAACTGGGAGAACGTGCTGCTCAGCCTCGGGGTCGCTGGCGGCGAGCCAGGGGTCGAAGGCGAGGAGATCGCGCCGCTCGCCAAGGAGAACGTGGCCGCGCCCTGAAGAGTTCGGCCTGCAGGGCCCCTGATCTCGCGCGTGGTGCAAAGATGTTGGGACATCTTCTTATATATGCTGTTTCGTTTATGTGATATGGACAAGTATGTGTAGCTGCTTGCTTGTGCTAGTGTAATATAGTGTAGTGGTGGCCAGTGGCACAACCTAATAAGCGCATGAACTAATTGCTTGCGTGTGTAGTTAAGTACCGATCGGTAATTTTATAT |
| SWL271 | 97.57 | CCACAACTGTTCGCGTCCTGCTGGTTCATTATCTGACCTGATTGCATTATTGCAGCTACGAGAAGCCCGTGGAAGGCCGGAAGATCAACTGGATGAAGGCCGGGATCCTCGAGGCCGACAGGGTCCTCACCGTCAGCCCCTACTACGCCGAGGAGCTCATCTCCGGCATCGCCAGGGGCTGCGAGCTCGACAACATCATGCGCCTCACCGGCATCACCGGCATCGTCAACGGCATGGACGTCAGCGAGTGGGACCCCAGCAGGGACAAGTACATCGCCGTGAAGTACGACGTGTCGACGGTGAGCTGGCTAGCTAGCTGATTCTGCTGCCTGGTCCTCCTGCTCATGCTGGTTCGGTTCTGACGCGGCAAGTGTACGTACGTGCGTGCGACGGTGGTGTGGTGTCCGGTTCAGGCCGTGGAGGCCAAGGCGCTGAACAAGGAGGCGCTGCAGGCGGAGGTCGGGCTCCCGGTGGACCGGAACATCCCGCTGGTGGCGTTCATCGGCAGGCTGGAAGAGCAGAAGGGACCCGACGTCATGGCGGCCGCCATCCCGCAGCTCATGGAGATGGTGGAGGACGTGCAGATCGTTCTGCTGGTACGTGTGCGCCGCCCGCCACCCGGCTACTACATGCGTGTATCGTTCGTTCTACTGGAACATGCGTGTGAGCAACGCGATGGATAATGCTGCAGGGCACGGGCAAGAAGAAGTTCGAGCGCATGCTCATGAGCGCCGAGGAGAAGTTCCCAGGCAAGGTGCGCGCCGTGGTCAAGTTCAACGCGGCGCTGGCGCACCACATCATGGCCGGCGCCGACGTGCTCGCCGTCACCAGCCGCTTCGAGCCCTGCGGCCTCATCCAGCTGCAGGGGATGCGATACGGAACGGTACGAGAGAGAAAAAAAAACATCCTGAATCCTGACGAGAGGGACAGAGACAGATTGATTATGAATGCTTCATCGATTTGAATTGATTGATCGATGTCTCCCGCTGCGACTCTTGCAGCCCTGCGCCTGCGCGTCCACCGGTGGACTCGTCGACACCATCATCGAAGGCAAGACCGGGTTCCACATGGGCCGCCTCAGCGTCGACGTAAGCCTACCTCTGCCATGTTCTTTCTTCTTTCTTTCTGTATGTATGTATGTATGTACGAATCAGCACCGCCATTCTTGTTTCGTCGTCCTCTCTTCCCAGTGCAACGTCGTGGAGCCGGCGGACGTCAAGAAGGTGGCCACCACCTTGCAGCGCGCCATCAAGGTGGTCGGCACGCCGGCGTACGAGGAGATGGTGAGGAACTGCATGATCCAGGATCTCTCCTGGAAGGTACGTACGCCCGCCCCGCCAGAGCAGAGCGCCAAGATCGATCGACCGACCGACCACACGTACGCGCCTCGCTCCTGTCGCTGACCGTGGTTTAATTTGCGAAATGCGCAGGGCCCTGCCAAGAACTGGGAGAACGTGCTGCTCAGCCTCGGGGTCGCTGGCGGCGAGCCAGGGGTCGAAGGCGAGGAGATCGCGCCGCTCGCCAAGGAGAACGTGGCCGCGCCCTGAAGAGTTCGGCCTGCAGGGCCCCTGATCTCGCGCGTGGTGCAAAGATGTTGGGACATCTTCTTATATATGCTGTTTCGTTTATGTGATATGGACAAGTATGTGTAGCTGCTTGCTTGTGCTAGTGTAATATAGTGTAGTGGTGGCCAGTGGCACAACCTAATAAGCGCATGAACTAATTGCTTGCGTGTGTAGTTAAGTACCGATCGGTAATTTTATAT |

**Table S1 Continued**

| **Accession** | **AC(%)** | **DNA Sequence** |
| --- | --- | --- |
| SWL273 | 97.80 | CCACAACTGTTCGCGTCCTGCTGGTTCATTATCTGACCTGATTGCATTATTGCAGATACGAGAAGCCCGTGGAAGCCGGAAGATCAACTGGAGAAGGCCGGGATCCTCGAGGCCGACAGGGTCCTCACCGTCAGCCCCTACTACGCCGAGGAGCTCATCTCCGGCATCGCCAGGGGCTGCGAGCTCGACAACATCATGCGCCTCACCGGCATCACCGGCATCGTCAACGGCATGGACGTCAGCGAGTGGGACCCCAGCAGGGACAAGTACATCGCCGTGAAGTACGACGTGTCGACGGTGAGCTGGCTAGCTAGCTGATTCTGCTGCCTGGTCCTCCTGCTCATGCTGGTTCGGTTCTGACGCGGCAAGTGTACGTACGTGCGTGCGACGGTGGTGTGGTGTCCGGTTCAGGCCGTGGAGGCCAAGGCGCTGAACAAGGAGGCGCTGCAGGCGGAGGTCGGGCTCCCGGTGGACCGGAACATCCCGCTGGTGGCGTTCATCGGCAGGCTGGAAGAGCAGAAGGGACCCGACGTCATGGCGGCCGCCATCCCGCAGCTCATGGAGATGGTGGAGGACGTGCAGATCGTTCTGCTGGTACGTGTGCGCCGCCCGCCACCCGGCTACTACATGCGTGTATCGTTCGTTCTACTGGAACATGCGTGTGAGCAACGCGATGGATAATGCTGCAGGGCACGGGCAAGAAGAAGTTCGAGCGCATGCTCATGAGCGCCGAGGAGAAGTTCCCAGGCAAGGTGCGCGCCGTGGTCAAGTTCAACGCGGCGCTGGCGCACCACATCATGGCCGGCGCCGACGTGCTCGCCGTCACCAGCCGCTTCGAGCCCTGCGGCCTCATCCAGCTGCAGGGGATGCGATACGGAACGGTACGAGAGAGAAAAAAAAACATCCTGAATCCTGACGAGAGGGACAGAGACAGATTGATTATGAATGCTTCATCGATTTGAATTGATTGATCGATGTCTCCCGCTGCGACTCTTGCAGCCCTGCGCCTGCGCGTCCACCGGTGGACTCGTCGACACCATCATCGAAGGCAAGACCGGGTTCCACATGGGCCGCCTCAGCGTCGACGTAAGCCTACCTCTGCCATGTTCTTTCTTCTTTCTTTCTGTATGTATGTATGTATGTACGAATCAGCACCGCCATTCTTGTTTCGTCGTCCTCTCTTCCCAGTGCAACGTCGTGGAGCCGGCGGACGTCAAGAAGGTGGCCACCACCTTGCAGCGCGCCATCAAGGTGGTCGGCACGCCGGCGTACGAGGAGATGGTGAGGAACTGCATGATCCAGGATCTCTCCTGGAAGGTACGTACGCCCGCCCCGCCAGAGCAGAGCGCCAAGATCGATCGACCGACCGACCACACGTACGCGCCTCGCTCCTGTCGCTGACCGTGGTTTAATTTGCGAAATGCGCAGGGCCCTGCCAAGAACTGGGAGAACGTGCTGCTCAGCCTCGGGGTCGCTGGCGGCGAGCCAGGGGTCGAAGGCGAGGAGATCGCGCCGCTCGCCAAGGAGAACGTGGCCGCGCCCTGAAGAGTTCGGCCTGCAGGGCCCCTGATCTCGCGCGTGGTGCAAAGATGTTGGGACATCTTCTTATATATGCTGTTTCGTTTATGTGATATGGACAAGTATGTGTAGCTGCTTGCTTGTGCTAGTGTAATATAGTGTAGTGGTGGCCAGTGGCACAACCTAATAAGCGCATGAACTAATTGCTTGCGTGTGTAGTTAAGTACCGATCGGTAATTTTATAT |
| SWL276 | 99.60 | CCACAACTGTTCGCGTCCTGCTGGTTCATTATCTGACCTGATTGCATTATTGCAGCTACGAGAAGCCCGTGGAAGGCCGGAAGATCAACTGGATGAAGGCCGGGATCCTCGAGGCCGACAGGGTCCTCACCGTCAGCCCCTACTACGCCGAGGAGCTCATCTCCGGCATCGCCAGGGGCTGCGAGCTCGACAACATCATGCGCCTCACCGGCATCACCGGCATCGTCAACGGCATGGACGTCAGCGAGTGGGACCCCAGCAGGGACAAGTACATCGCCGTGAAGTACGACGTGTCGACGGTGAGCTGGCTAGCTAGCTGATTCTGCTGCCTGGTCCTCCTGCTCATGCTGGTTCGGTTCTGACGCGGCAAGTGTACGTACGTGCGTGCGACGGTGGTGTGGTGTCCGGTTCAGGCCGTGGAGGCCAAGGCGCTGAACAAGGAGGCGCTGCAGGCGGAGGTCGGGCTCCCGGTGGACCGGAACATCCCGCTGGTGGCGTTCATCGGCAGGCTGGAAGAGCAGAAGGGACCCGACGTCATGGCGGCCGCCATCCCGCAGCTCATGGAGATGGTGGAGGACGTGCAGATCGTTCTGCTGGTACGTGTGCGCCGCCCGCCACCCGGCTACTACATGCGTGTATCGTTCGTTCTACTGGAACATGCGTGTGAGCAACGCGATGGATAATGCTGCAGGGCACGGGCAAGAAGAAGTTCGAGCGCATGCTCATGAGCGCCGAGGAGAAGTTCCCAGGCAAGGTGCGCGCCGTGGTCAAGTTCAACGCGGCGCTGGCGCACCACATCATGGCCGGCGCCGACGTGCTCGCCGTCACCAGCCGCTTCGAGCCCTGCGGCCTCATCCAGCTGCAGGGGATGCGATACGGAACGGTACGAGAGAGAAAAAAAAACATCCTGAATCCTGACGAGAGGGACAGAGACAGATTGATTATGAATGCTTCATCGATTTGAATTGATTGATCGATGTCTCCCGCTGCGACTCTTGCAGCCCTGCGCCTGCGCGTCCACCGGTGGACTCGTCGACACCATCATCGAAGGCAAGACCGGGTTCCACATGGGCCGCCTCAGCGTCGACGTAAGCCTACCTCTGCCATGTTCTTTCTTCTTTCTTTCTGTATGTATGTATGTATGTACGAATCAGCACCGCCATTCTTGTTTCGTCGTCCTCTCTTCCCAGTGCAACGTCGTGGAGCCGGCGGACGTCAAGAAGGTGGCCACCACCTTGCAGCGCGCCATCAAGGTGGTCGGCACGCCGGCGTACGAGGAGATGGTGAGGAACTGCATGATCCAGGATCTCTCCTGGAAGGTACGTACGCCCGCCCCGCCAGAGCAGAGCGCCAAGATCGATCGACCGACCGACCACACGTACGCGCCTCGCTCCTGTCGCTGACCGTGGTTTAATTTGCGAAATGCGCAGGGCCCTGCCAAGAACTGGGAGAACGTGCTGCTCAGCCTCGGGGTCGCTGGCGGCGAGCCAGGGGTCGAAGGCGAGGAGATCGCGCCGCTCGCCAAGGAGAACGTGGCCGCGCCCTGAAGAGTTCGGCCTGCAGGGCCCCTGATCTCGCGCGTGGTGCAAAGATGTTGGGACATCTTCTTATATATGCTGTTTCGTTTATGTGATATGGACAAGTATGTGTAGCTGCTTGCTTGTGCTAGTGTAATATAGTGTAGTGGTGGCCAGTGGCACAACCTAATAAGCGCATGAACTAATTGCTTGCGTGTGTAGTTAAGTACCGATCGGTAATTTTATAT |

**Table S1 Continued**

| **Accession** | **AC(%)** | **DNA Sequence** |
| --- | --- | --- |
| SWL277 | 98.51 | CCACAACTGTTCGCGTCCTGCTGGTTCATTATCTGACCTGATTGCATTATTGCAGCTACGAGAAGCCCGTGGAAGGCCGGAAGATCAACTGGATGAAGGCCGGGATCCTCGAGGCCGACAGGGTCCTCACCGTCAGCCCCTACTACGCCGAGGAGCTCATCTCCGGCATCGCCAGGGGCTGCGAGCTCGACAACATCATGCGCCTCACCGGCATCACCGGCATCGTCAACGGCATGGACGTCAGCGAGTGGGACCCCAGCAGGGACAAGTACATCGCCGTGAAGTACGACGTGTCGACGGTGAGCTGGCTAGCTAGCTGATTCTGCTGCCTGGTCCTCCTGCTCATGCTGGTTCGGTTCTGACGCGGCAAGTGTACGTACGTGCGTGCGACGGTGGTGTGGTGTCCGGTTCAGGCCGTGGAGGCCAAGGCGCTGAACAAGGAGGCGCTGCAGGCGGAGGTCGGGCTCCCGGTGGACCGGAACATCCCGCTGGTGGCGTTCATCGGCAGGCTGGAAGAGCAGAAGGGACCCGACGTCATGGCGGCCGCCATCCCGCAGCTCATGGAGATGGTGGAGGACGTGCAGATCGTTCTGCTGGTACGTGTGCGCCGCCCGCCACCCGGCTACTACATGCGTGTATCGTTCGTTCTACTGGAACATGCGTGTGAGCAACGCGATGGATAATGCTGCAGGGCACGGGCAAGAAGAAGTTCGAGCGCATGCTCATGAGCGCCGAGGAGAAGTTCCCAGGCAAGGTGCGCGCCGTGGTCAAGTTCAACGCGGCGCTGGCGCACCACATCATGGCCGGCGCCGACGTGCTCGCCGTCACCAGCCGCTTCGAGCCCTGCGGCCTCATCCAGCTGCAGGGGATGCGATACGGAACGGTACGAGAGAGAAAAAAAAACATCCTGAATCCTGACGAGAGGGACAGAGACAGATTGATTATGAATGCTTCATCGATTTGAATTGATTGATCGATGTCTCCCGCTGCGACTCTTGCAGCCCTGCGCCTGCGCGTCCACCGGTGGACTCGTCGACACCATCATCGAAGGCAAGACCGGGTTCCACATGGGCCGCCTCAGCGTCGACGTAAGCCTACCTCTGCCATGTTCTTTCTTCTTTCTTTCTGTATGTATGTATGTATGTACGAATCAGCACCGCCATTCTTGTTTCGTCGTCCTCTCTTCCCAGTGCAACGTCGTGGAGCCGGCGGACGTCAAGAAGGTGGCCACCACCTTGCAGCGCGCCATCAAGGTGGTCGGCACGCCGGCGTACGAGGAGATGGTGAGGAACTGCATGATCCAGGATCTCTCCTGGAAGGTACGTACGCCCGCCCCGCCAGAGCAGAGCGCCAAGATCGATCGACCGACCGACCACACGTACGCGCCTCGCTCCTGTCGCTGACCGTGGTTTAATTTGCGAAATGCGCAGGGCCCTGCCAAGAACTGGGAGAACGTGCTGCTCAGCCTCGGGGTCGCTGGCGGCGAGCCAGGGGTCGAAGGCGAGGAGATCGCGCCGCTCGCCAAGGAGAACGTGGCCGCGCCCTGAAGAGTTCGGCCTGCAGGGCCCCTGATCTCGCGCGTGGTGCAAAGATGTTGGGACATCTTCTTATATATGCTGTTTCGTTTATGTGATATGGACAAGTATGTGTAGCTGCTTGCTTGTGCTAGTGTAATATAGTGTAGTGGTGGCCAGTGGCACAACCTAATAAGCGCATGAACTAATTGCTTGCGTGTGTAGTTAAGTACCGATCGGTAATTTTTATAT |
| SWL278 | 98.26 | CCACAACTGTTCGCGTCCTGCTGGTTCATTATCTGACCTGATTGCATTATTGCAGCTACGAGAAGCCCGTGGAAGGCCGGAAGATCAACTGGATGAAGGCCGGGATCCTCGAGGCCGACAGGGTCCTCACCGTCAGCCCCTACTACGCCGAGGAGCTCATCTCCGGCATCGCCAGGGGCTGCGAGCTCGACAACATCATGCGCCTCACCGGCATCACCGGCATCGTCAACGGCATGGACGTCAGCGAGTGGGACCCCAGCAGGGACAAGTACATCGCCGTGAAGTACGACGTGTCGACGGTGAGCTGGCTAGCTAGCTGATTCTGCTGCCTGGTCCTCCTGCTCATGCTGGTTCGGTTCTGACGCGGCAAGTGTACGTACGTGCGTGCGACGGTGGTGTGGTGTCCGGTTCAGGCCGTGGAGGCCAAGGCGCTGAACAAGGAGGCGCTGCAGGCGGAGGTCGGGCTCCCGGTGGACCGGAACATCCCGCTGGTGGCGTTCATCGGCAGGCTGGAAGAGCAGAAGGGACCCGACGTCATGGCGGCCGCCATCCCGCAGCTCATGGAGATGGTGGAGGACGTGCAGATCGTTCTGCTGGTACGTGTGCGCCGCCCGCCACCCGGCTACTACATGCGTGTATCGTTCGTTCTACTGGAACATGCGTGTGAGCAACGCGATGGATAATGCTGCAGGGCACGGGCAAGAAGAAGTTCGAGCGCATGCTCATGAGCGCCGAGGAGAAGTTCCCAGGCAAGGTGCGCGCCGTGGTCAAGTTCAACGCGGCGCTGGCGCACCACATCATGGCCGGCGCCGACGTGCTCGCCGTCACCAGCCGCTTCGAGCCCTGCGGCCTCATCCAGCTGCAGGGGATGCGATACGGAACGGTACGAGAGAGAAAAAAAAACATCCTGAATCCTGACGAGAGGGACAGAGACAGATTGATTATGAATGCTTCATCGATTTGAATTGATTGATCGATGTCTCCCGCTGCGACTCTTGCAGCCCTGCGCCTGCGCGTCCACCGGTGGACTCGTCGACACCATCATCGAAGGCAAGACCGGGTTCCACATGGGCCGCCTCAGCGTCGACGTAAGCCTACCTCTGCCATGTTCTTTCTTCTTTCTTTCTGTATGTATGTATGTATGTACGAATCAGCACCGCCATTCTTGTTTCGTCGTCCTCTCTTCCCAGTGCAACGTCGTGGAGCCGGCGGACGTCAAGAAGGTGGCCACCACCTTGCAGCGCGCCATCAAGGTGGTCGGCACGCCGGCGTACGAGGAGATGGTGAGGAACTGCATGATCCAGGATCTCTCCTGGAAGGTACGTACGCCCGCCCCGCCAGAGCAGAGCGCCAAGATCGATCGACCGACCGACCACACGTACGCGCCTCGCTCCTGTCGCTGACCGTGGTTTAATTTGCGAAATGCGCAGGGCCCTGCCAAGAACTGGGAGAACGTGCTGCTCAGCCTCGGGGTCGCTGGCGGCGAGCCAGGGGTCGAAGGCGAGGAGATCGCGCCGCTCGCCAAGGAGAACGTGGCCGCGCCCTGAAGAGTTCGGCCTGCAGGGCCCCTGATCTCGCGCGTGGTGCAAAGATGTTGGGACATCTTCTTATATATGCTGTTTCGTTTATGTGATATGGACAAGTATGTGTAGCTGCTTGCTTGTGCTAGTGTAATATAGTGTAGTGGTGGCCAGTGGCACAACCTAATAAGCGCATGAACTAATTGCTTGCGTGTGTAGTTAAGTACCGATCGGTAATTTTATAT |

**Table S1 Continued**

| **Accession** | **AC(%)** | **DNA Sequence** |
| --- | --- | --- |
| SWL283 | 97.90 | CCACAACTGTTCGCGTCCTGCTGGTTCATTATCTGACCTGATTGCATTATTGCAGCTACGAGAAGCCCGTGGAAGGCCGGAAGATCAACTGGATGAAGGCCGGGATCCTCGAGGCCGACAGGGTCCTCACCGTCAGCCCCTACTACGCCGAGGAGCTCATCTCCGGCATCGCCAGGGGCTGCGAGCTCGACAACATCATGCGCCTCACCGGCATCACCGGCATCGTCAACGGCATGGACGTCAGCGAGTGGGACCCCAGCAGGGACAAGTACATCGCCGTGAAGTACGACGTGTCGACGGTGAGCTGGCTAGCTAGCTGATTCTGCTGCCTGGTCCTCCTGCTCATGCTGGTTCGGTTCTGACGCGGCAAGTGTACGTACGTGCGTGCGACGGTGGTGTGGTGTCCGGTTCAGGCCGTGGAGGCCAAGGCGCTGAACAAGGAGGCGCTGCAGGCGGAGGTCGGGCTCCCGGTGGACCGGAACATCCCGCTGGTGGCGTTCATCGGCAGGCTGGAAGAGCAGAAGGGACCCGACGTCATGGCGGCCGCCATCCCGCAGCTCATGGAGATGGTGGAGGACGTGCAGATCGTTCTGCTGGTACGTGTGCGCCGCCCGCCACCCGGCTACTACATGCGTGTATCGTTCGTTCTACTGGAACATGCGTGTGAGCAACGCGATGGATAATGCTGCAGGGCACGGGCAAGAAGAAGTTCGAGCGCATGCTCATGAGCGCCGAGGAGAAGTTCCCAGGCAAGGTGCGCGCCGTGGTCAAGTTCAACGCGGCGCTGGCGCACCACATCATGGCCGGCGCCGACGTGCTCGCCGTCACCAGCCGCTTCGAGCCCTGCGGCCTCATCCAGCTGCAGGGGATGCGATACGGAACGGTACGAGAGAGAAAAAAAAACATCCTGAATCCTGACGAGAGGGACAGAGACAGATTGATTATGAATGCTTCATCGATTTGAATTGATTGATCGATGTCTCCCGCTGCGACTCTTGCAGCCCTGCGCCTGCGCGTCCACCGGTGGACTCGTCGACACCATCATCGAAGGCAAGACCGGGTTCCACATGGGCCGCCTCAGCGTCGACGTAAGCCTACCTCTGCCATGTTCTTTCTTCTTTCTTTCTGTATGTATGTATGTATGTACGAATCAGCACCGCCATTCTTGTTTCGTCGTCCTCTCTTCCCAGTGCAACGTCGTGGAGCCGGCGGACGTCAAGAAGGTGGCCACCACCTTGCAGCGCGCCATCAAGGTGGTCGGCACGCCGGCGTACGAGGAGATGGTGAGGAACTGCATGATCCAGGATCTCTCCTGGAAGGTACGTACGCCCGCCCCGCCAGAGCAGAGCGCCAAGATCGATCGACCGACCGACCACACGTACGCGCCTCGCTCCTGTCGCTGACCGTGGTTTAATTTGCGAAATGCGCAGGGCCCTGCCAAGAACTGGGAGAACGTGCTGCTCAGCCTCGGGGTCGCTGGCGGCGAGCCAGGGGTCGAAGGCGAGGAGATCGCGCCGCTCGCCAAGGAGAACGTGGCCGCGCCCTGAAGAGTTCGGCCTGCAGGGCCCCTGATCTCGCGCGTGGTGCAAAGATGTTGGGACATCTTCTTATATATGCTGTTTCGTTTATGTGATATGGACAAGTATGTGTAGCTGCTTGCTTGTGCTAGTGTAATATAGTGTAGTGGTGGCCAGTGGCACAACCTAATAAGCGCATGAACTAATTGCTTGCGTGTGTAGTTAAGTACCGATCGGTAATTTTATAT |
| SWL284 | 99.07 | CCACAACTGTTCGCGTCCTGCTGGTTCATTATCTGACCTGATTGCATTATTGCAGCTACGAGAAGCCCGTGGAAGGCCGGAAGATCAACTGGATGAAGGCCGGGATCCTCGAGGCCGACAGGGTCCTCACCGTCAGCCCCTACTACGCCGAGGAGCTCATCTCCGGCATCGCCAGGGGCTGCGAGCTCGACAACATCATGCGCCTCACCGGCATCACCGGCATCGTCAACGGCATGGACGTCAGCGAGTGGGACCCCAGCAGGGACAAGTACATCGCCGTGAAGTACGACGTGTCGACGGTGAGCTGGCTAGCTAGCTGATTCTGCTGCCTGGTCCTCCTGCTCATGCTGGTTCGGTTCTGACGCGGCAAGTGTACGTACGTGCGTGCGACGGTGGTGTGGTGTCCGGTTCAGGCCGTGGAGGCCAAGGCGCTGAACAAGGAGGCGCTGCAGGCGGAGGTCGGGCTCCCGGTGGACCGGAACATCCCGCTGGTGGCGTTCATCGGCAGGCTGGAAGAGCAGAAGGGACCCGACGTCATGGCGGCCGCCATCCCGCAGCTCATGGAGATGGTGGAGGACGTGCAGATCGTTCTGCTGGTACGTGTGCGCCGCCCGCCACCCGGCTACTACATGCGTGTATCGTTCGTTCTACTGGAACATGCGTGTGAGCAACGCGATGGATAATGCTGCAGGGCACGGGCAAGAAGAAGTTCGAGCGCATGCTCATGAGCGCCGAGGAGAAGTTCCCAGGCAAGGTGCGCGCCGTGGTCAAGTTCAACGCGGCGCTGGCGCACCACATCATGGCCGGCGCCGACGTGCTCGCCGTCACCAGCCGCTTCGAGCCCTGCGGCCTCATCCAGCTGCAGGGGATGCGATACGGAACGGTACGAGAGAGAAAAAAAAACATCCTGAATCCTGACGAGAGGGACAGAGACAGATTGATTATGAATGCTTCATCGATTTGAATTGATTGATCGATGTCTCCCGCTGCGACTCTTGCAGCCCTGCGCCTGCGCGTCCACCGGTGGACTCGTCGACACCATCATCGAAGGCAAGACCGGGTTCCACATGGGCCGCCTCAGCGTCGACGTAAGCCTACCTCTGCCATGTTCTTTCTTCTTTCTTTCTGTATGTATGTATGTATGTACGAATCAGCACCGCCATTCTTGTTTCGTCGTCCTCTCTTCCCAGTGCAACGTCGTGGAGCCGGCGGACGTCAAGAAGGTGGCCACCACCTTGCAGCGCGCCATCAAGGTGGTCGGCACGCCGGCGTACGAGGAGATGGTGAGGAACTGCATGATCCAGGATCTCTCCTGGAAGGTACGTACGCCCGCCCCGCCAGAGCAGAGCGCCAAGATCGATCGACCGACCGACCACACGTACGCGCCTCGCTCCTGTCGCTGACCGTGGTTTAATTTGCGAAATGCGCAGGGCCCTGCCAAGAACTGGGAGAACGTGCTGCTCAGCCTCGGGGTCGCTGGCGGCGAGCCAGGGGTCGAAGGCGAGGAGATCGCGCCGCTCGCCAAGGAGAACGTGGCCGCGCCCTGAAGAGTTCGGCCTGCAGGGCCCCTGATCTCGCGCGTGGTGCAAAGATGTTGGGACATCTTCTTATATATGCTGTTTCGTTTATGTGATATGGACAAGTATGTGTAGCTGCTTGCTTGTGCTAGTGTAATATAGTGTAGTGGTGGCCAGTGGCACAACCTAATAAGCGCATGAACTAATTGCTTGCGTGTGTAGTTAAGTACCGATCGGTAATTTTATAT |

**Table S1 Continued**

| **Accession** | **AC(%)** | **DNA Sequence** |
| --- | --- | --- |
| SWL285 | 98.44 | CCACAACTGTTCGCGTCCTGCTGGTTCATTATCTGACCTGATTGCATTATTGCAGCTACGAGAAGCCCGTGGAAGGCCGGAAGATCAACTGGATGAAGGCCGGGATCCTCGAGGCCGACAGGGTCCTCACCGTCAGCCCCTACTACGCCGAGGAGCTCATCTCCGGCATCGCCAGGGGCTGCGAGCTCGACAACATCATGCGCCTCACCGGCATCACCGGCATCGTCAACGGCATGGACGTCAGCGAGTGGGACCCCAGCAGGGACAAGTACATCGCCGTGAAGTACGACGTGTCGACGGTGAGCTGGCTAGCTAGCTGATTCTGCTGCCTGGTCCTCCTGCTCATGCTGGTTCGGTTCTGACGCGGCAAGTGTACGTACGTGCGTGCGACGGTGGTGTGGTGTCCGGTTCAGGCCGTGGAGGCCAAGGCGCTGAACAAGGAGGCGCTGCAGGCGGAGGTCGGGCTCCCGGTGGACCGGAACATCCCGCTGGTGGCGTTCATCGGCAGGCTGGAAGAGCAGAAGGGACCCGACGTCATGGCGGCCGCCATCCCGCAGCTCATGGAGATGGTGGAGGACGTGCAGATCGTTCTGCTGGTACGTGTGCGCCGCCCGCCACCCGGCTACTACATGCGTGTATCGTTCGTTCTACTGGAACATGCGTGTGAGCAACGCGATGGATAATGCTGCAGGGCACGGGCAAGAAGAAGTTCGAGCGCATGCTCATGAGCGCCGAGGAGAAGTTCCCAGGCAAGGTGCGCGCCGTGGTCAAGTTCAACGCGGCGCTGGCGCACCACATCATGGCCGGCGCCGACGTGCTCGCCGTCACCAGCCGCTTCGAGCCCTGCGGCCTCATCCAGCTGCAGGGGATGCGATACGGAACGGTACGAGAGAGAAAAAAAAACATCCTGAATCCTGACGAGAGGGACAGAGACAGATTGATTATGAATGCTTCATCGATTTGAATTGATTGATCGATGTCTCCCGCTGCGACTCTTGCAGCCCTGCGCCTGCGCGTCCACCGGTGGACTCGTCGACACCATCATCGAAGGCAAGACCGGGTTCCACATGGGCCGCCTCAGCGTCGACGTAAGCCTACCTCTGCCATGTTCTTTCTTCTTTCTTTCTGTATGTATGTATGTATGTACGAATCAGCACCGCCATTCTTGTTTCGTCGTCCTCTCTTCCCAGTGCAACGTCGTGGAGCCGGCGGACGTCAAGAAGGTGGCCACCACCTTGCAGCGCGCCATCAAGGTGGTCGGCACGCCGGCGTACGAGGAGATGGTGAGGAACTGCATGATCCAGGATCTCTCCTGGAAGGTACGTACGCCCGCCCCGCCAGAGCAGAGCGCCAAGATCGATCGACCGACCGACCACACGTACGCGCCTCGCTCCTGTCGCTGACCGTGGTTTAATTTGCGAAATGCGCAGGGCCCTGCCAAGAACTGGGAGAACGTGCTGCTCAGCCTCGGGGTCGCTGGCGGCGAGCCAGGGGTCGAAGGCGAGGAGATCGCGCCGCTCGCCAAGGAGAACGTGGCCGCGCCCTGAAGAGTTCGGCCTGCAGGGCCCCTGATCTCGCGCGTGGTGCAAAGATGTTGGGACATCTTCTTATATATGCTGTTTCGTTTATGTGATATGGACAAGTATGTGTAGCTGCTTGCTTGTGCTAGTGTAATATAGTGTAGTGGTGGCCAGTGGCACAACCTAATAAGCGCATGAACTAATTGCTTGCGTGTGTAGTTAAGTACCGATCGGTAATTTTATAT |
| SWL286 | 98.34 | CCACAACTGTTCGCGTCCTGCTGGTTCATTATCTGACCTGATTGCATTATTGCAGCTACGAGAAGCCCGTGGAAGGCCGGAAGATCAACTGGATGAAGGCCGGGATCCTCGAGGCCGACAGGGTCCTCACCGTCAGCCCCTACTACGCCGAGGAGCTCATCTCCGGCATCGCCAGGGGCTGCGAGCTCGACAACATCATGCGCCTCACCGGCATCACCGGCATCGTCAACGGCATGGACGTCAGCGAGTGGGACCCCAGCAGGGACAAGTACATCGCCGTGAAGTACGACGTGTCGACGGTGAGCTGGCTAGCTAGCTGATTCTGCTGCCTGGTCCTCCTGCTCATGCTGGTTCGGTTCTGACGCGGCAAGTGTACGTACGTGCGTGCGACGGTGGTGTGGTGTCCGGTTCAGGCCGTGGAGGCCAAGGCGCTGAACAAGGAGGCGCTGCAGGCGGAGGTCGGGCTCCCGGTGGACCGGAACATCCCGCTGGTGGCGTTCATCGGCAGGCTGGAAGAGCAGAAGGGACCCGACGTCATGGCGGCCGCCATCCCGCAGCTCATGGAGATGGTGGAGGACGTGCAGATCGTTCTGCTGGTACGTGTGCGCCGCCCGCCACCCGGCTACTACATGCGTGTATCGTTCGTTCTACTGGAACATGCGTGTGAGCAACGCGATGGATAATGCTGCAGGGCACGGGCAAGAAGAAGTTCGAGCGCATGCTCATGAGCGCCGAGGAGAAGTTCCCAGGCAAGGTGCGCGCCGTGGTCAAGTTCAACGCGGCGCTGGCGCACCACATCATGGCCGGCGCCGACGTGCTCGCCGTCACCAGCCGCTTCGAGCCCTGCGGCCTCATCCAGCTGCAGGGGATGCGATACGGAACGGTACGAGAGAGAAAAAAAAACATCCTGAATCCTGACGAGAGGGACAGAGACAGATTGATTATGAATGCTTCATCGATTTGAATTGATTGATCGATGTCTCCCGCTGCGACTCTTGCAGCCCTGCGCCTGCGCGTCCACCGGTGGACTCGTCGACACCATCATCGAAGGCAAGACCGGGTTCCACATGGGCCGCCTCAGCGTCGACGTAAGCCTACCTCTGCCATGTTCTTTCTTCTTTCTTTCTGTATGTATGTATGTATGTACGAATCAGCACCGCCATTCTTGTTTCGTCGTCCTCTCTTCCCAGTGCAACGTCGTGGAGCCGGCGGACGTCAAGAAGGTGGCCACCACCTTGCAGCGCGCCATCAAGGTGGTCGGCACGCCGGCGTACGAGGAGATGGTGAGGAACTGCATGATCCAGGATCTCTCCTGGAAGGTACGTACGCCCGCCCCGCCAGAGCAGAGCGCCAAGATCGATCGACCGACCGACCACACGTACGCGCCTCGCTCCTGTCGCTGACCGTGGTTTAATTTGCGAAATGCGCAGGGCCCTGCCAAGAACTGGGAGAACGTGCTGCTCAGCCTCGGGGTCGCTGGCGGCGAGCCAGGGGTCGAAGGCGAGGAGATCGCGCCGCTCGCCAAGGAGAACGTGGCCGCGCCCTGAAGAGTTCGGCCTGCAGGGCCCCTGATCTCGCGCGTGGTGCAAAGATGTTGGGACATCTTCTTATATATGCTGTTTCGTTTATGTGATATGGACAAGTATGTGTAGCTGCTTGCTTGTGCTAGTGTAATATAGTGTAGTGGTGGCCAGTGGCACAACCTAATAAGCGCATGAACTAATTGCTTGCGTGTGTAGTTAAGTACCGATCGGTAATTTTATAT |

**Table S1 Continued**

| **Accession** | **AC(%)** | **DNA Sequence** |
| --- | --- | --- |
| SWL287 | 98.15 | CCACAACTGTTCGCGTCTGCTGGTTCATTATCTGACCTGATTGCATTATTGCAGTACGAGAAGCCCGTGGAAGGCCGGAAGATCAACTGGATGAAGGCCGGGATCCTCGAGGCCGACAGGGTCCTCACCGTCAGCCCCTACTACGCCGAGGAGCTCATCTCCGGCATCGCCAGCGGCTGCGAGCTCGACAACATCATGCGCCTCACCGGCATCACCGGCATCGTCAACGGCATGGACGTCAGCGAGTGGGACCCCAGCAGGGACAAGTACATCGCCGTGAAGTACGACGTGTCGACGGTGAGCTGGCTAGCTAGCTGATTCTGCTGCCTGGTCCTCCTGCTCATGCTGGTTCGGTTCTGACGCGGCAAGTGTACGTACGTGCGTGCGACGGTGGTGTGGTGTCCGGTTCAGGCCGTGGAGGCCAAGGCGCTGAACAAGGAGGCGCTGCAGGCGGAGGTCGGGCTCCCGGTGGACCGGAACATCCCGCTGGTGGCGTTCATCGGCAGGCTGGAAGAGCAGAAGGGACCCGACGTCATGGCGGCCGCCATCCCGCAGCTCATGGAGATGGTGGAGGACGTGCAGATCGTTCTGCTGGTACGTGTGCGCCGCCCGCCACCCGGCTACTACATGCGTGTATCGTTCGTTCTACTGGAACATGCGTGTGAGCAACGCGATGGATAATGCTGCAGGGCACGGGCAAGAAGAAGTTCGAGCGCATGCTCATGAGCGCCGAGGAGAAGTTCCCAGGCAAGGTGCGCGCCGTGGTCAAGTTCAACGCGGCGCTGGCGCACCACATCATGGCCGGCGCCGACGTGCTCGCCGTCACCAGCCGCTTCGAGCCCTGCGGCCTCATCCAGCTGCAGGGGATGCGATACGGAACGGTACGAGAGAGAAAAAAAAACATCCTGAATCCTGACGAGAGGGACAGAGACAGATTGATTATGAATGCTTCATCGATTTGAATTGATTGATCGATGTCTCCCGCTGCGACTCTTGCAGCCCTGCGCCTGCGCGTCCACCGGTGGACTCGTCGACACCATCATCGAAGGCAAGACCGGGTTCCACATGGGCCGCCTCAGCGTCGACGTAAGCCTACCTCTGCCATGTTCTTTCTTCTTTCTTTCTGTATGTATGTATGTATGTACGAATCAGCACCGCCATTCTTGTTTCGTCGTCCTCTCTTCCCAGTGCAACGTCGTGGAGCCGGCGGACGTCAAGAAGGTGGCCACCACCTTGCAGCGCGCCATCAAGGTGGTCGGCACGCCGGCGTACGAGGAGATGGTGAGGAACTGCATGATCCAGGATCTCTCCTGGAAGGTACGTACGCCCGCCCCGCCAGAGCAGAGCGCCAAGATCGATCGACCGACCGACCACACGTACGCGCCTCGCTCCTGTCGCTGACCGTGGTTTAATTTGCGAAATGCGCAGGGCCCTGCCAAGAACTGGGAGAACGTGCTGCTCAGCCTCGGGGTCGCTGGCGGCGAGCCAGGGGTCGAAGGCGAGGAGATCGCGCCGCTCGCCAAGGAGAACGTGGCCGCGCCCTGAAGAGTTCGGCCTGCAGGGCCCCTGATCTCGCGCGTGGTGCAAAGATGTTGGGACATCTTCTTATATATGCTGTTTCGTTTATGTGATATGGACAAGTATGTGTAGCTGCTTGCTTGTGCTAGTGTAATATAGTGTAGTGGTGGCCAGTGGCACAACCTAATAAGCGCATGAACTAATTGCTTGCGTGTGTAGTTAAGTACCGATCGGTAATTTTATAT |
| SWL290 | 98.16 | CCACAACTGTTCGCGTCCTGCTGGTTCATTATCTGACCTGATTGCATTATTGCAGCTACGAGAAGCCCGTGGAAGGCCGGAAGATCAACTGGATGAAGGCCGGGATCCTCGAGGCCGACAGGGTCCTCACCGTCAGCCCCTACTACGCCGAGGAGCTCATCTCCGGCATCGCCAGGGGCTGCGAGCTCGACAACATCATGCGCCTCACCGGCATCACCGGCATCGTCAACGGCATGGACGTCAGCGAGTGGGACCCCAGCAGGGACAAGTACATCGCCGTGAAGTACGACGTGTCGACGGTGAGCTGGCTAGCTAGCTGATTCTGCTGCCTGGTCCTCCTGCTCATGCTGGTTCGGTTCTGACGCGGCAAGTGTACGTACGTGCGTGCGACGGTGGTGTGGTGTCCGGTTCAGGCCGTGGAGGCCAAGGCGCTGAACAAGGAGGCGCTGCAGGCGGAGGTCGGGCTCCCGGTGGACCGGAACATCCCGCTGGTGGCGTTCATCGGCAGGCTGGAAGAGCAGAAGGGACCCGACGTCATGGCGGCCGCCATCCCGCAGCTCATGGAGATGGTGGAGGACGTGCAGATCGTTCTGCTGGTACGTGTGCGCCGCCCGCCACCCGGCTACTACATGCGTGTATCGTTCGTTCTACTGGAACATGCGTGTGAGCAACGCGATGGATAATGCTGCAGGGCACGGGCAAGAAGAAGTTCGAGCGCATGCTCATGAGCGCCGAGGAGAAGTTCCCAGGCAAGGTGCGCGCCGTGGTCAAGTTCAACGCGGCGCTGGCGCACCACATCATGGCCGGCGCCGACGTGCTCGCCGTCACCAGCCGCTTCGAGCCCTGCGGCCTCATCCAGCTGCAGGGGATGCGATACGGAACGGTACGAGAGAGAAAAAAAAACATCCTGAATCCTGACGAGAGGGACAGAGACAGATTGATTATGAATGCTTCATCGATTTGAATTGATTGATCGATGTCTCCCGCTGCGACTCTTGCAGCCCTGCGCCTGCGCGTCCACCGGTGGACTCGTCGACACCATCATCGAAGGCAAGACCGGGTTCCACATGGGCCGCCTCAGCGTCGACGTAAGCCTACCTCTGCCATGTTCTTTCTTCTTTCTTTCTGTATGTATGTATGTATGTACGAATCAGCACCGCCATTCTTGTTTCGTCGTCCTCTCTTCCCAGTGCAACGTCGTGGAGCCGGCGGACGTCAAGAAGGTGGCCACCACCTTGCAGCGCGCCATCAAGGTGGTCGGCACGCCGGCGTACGAGGAGATGGTGAGGAACTGCATGATCCAGGATCTCTCCTGGAAGGTACGTACGCCCGCCCCGCCAGAGCAGAGCGCCAAGATCGATCGACCGACCGACCACACGTACGCGCCTCGCTCCTGTCGCTGACCGTGGTTTAATTTGCGAAATGCGCAGGGCCCTGCCAAGAACTGGGAGAACGTGCTGCTCAGCCTCGGGGTCGCTGGCGGCGAGCCAGGGGTCGAAGGCGAGGAGATCGCGCCGCTCGCCAAGGAGAACGTGGCCGCGCCCTGAAGAGTTCGGCCTGCAGGGCCCCTGATCTCGCGCGTGGTGCAAAGATGTTGGGACATCTTCTTATATATGCTGTTTCGTTTATGTGATATGGACAAGTATGTGTAGCTGCTTGCTTGTGCTAGTGTAATATAGTGTAGTGGTGGCCAGTGGCACAACCTAATAAGCGCATGAACTAATTGCTTGCGTGTGTAGTTAAGTACCGATCGGTAATTTTATAT |

**Table S1 Continued**

| **Accession** | **AC(%)** | **DNA Sequence** |
| --- | --- | --- |
| SWL294 | 98.42 | CCACAACTGTTCGCGTCCTGCTGGTTCATTATCTGACCTGATTGCATTATTGCAGCTACGAGAAGCCCGTGGAAGGCCGGAAGATCAACTGGATGAAGGCCGGGATCCTCGAGGCCGACAGGGTCCTCACCGTCAGCCCCTACTACGCCGAGGAGCTCATCTCCGGCATCGCCAGGGGCTGCGAGCTCGACAACATCATGCGCCTCACCGGCATCACCGGCATCGTCAACGGCATGGACGTCAGCGAGTGGGACCCCAGCAGGGACAAGTACATCGCCGTGAAGTACGACGTGTCGACGGTGAGCTGGCTAGCTAGCTGATTCTGCTGCCTGGTCCTCCTGCTCATGCTGGTTCGGTTCTGACGCGGCAAGTGTACGTACGTGCGTGCGACGGTGGTGTGGTGTCCGGTTCAGGCCGTGGAGGCCAAGGCGCTGAACAAGGAGGCGCTGCAGGCGGAGGTCGGGCTCCCGGTGGACCGGAACATCCCGCTGGTGGCGTTCATCGGCAGGCTGGAAGAGCAGAAGGGACCCGACGTCATGGCGGCCGCCATCCCGCAGCTCATGGAGATGGTGGAGGACGTGCAGATCGTTCTGCTGGTACGTGTGCGCCGCCCGCCACCCGGCTACTACATGCGTGTATCGTTCGTTCTACTGGAACATGCGTGTGAGCAACGCGATGGATAATGCTGCAGGGCACGGGCAAGAAGAAGTTCGAGCGCATGCTCATGAGCGCCGAGGAGAAGTTCCCAGGCAAGGTGCGCGCCGTGGTCAAGTTCAACGCGGCGCTGGCGCACCACATCATGGCCGGCGCCGACGTGCTCGCCGTCACCAGCCGCTTCGAGCCCTGCGGCCTCATCCAGCTGCAGGGGATGCGATACGGAACGGTACGAGAGAGAAAAAAAAACATCCTGAATCCTGACGAGAGGGACAGAGACAGATTGATTATGAATGCTTCATCGATTTGAATTGATTGATCGATGTCTCCCGCTGCGACTCTTGCAGCCCTGCGCCTGCGCGTCCACCGGTGGACTCGTCGACACCATCATCGAAGGCAAGACCGGGTTCCACATGGGCCGCCTCAGCGTCGACGTAAGCCTACCTCTGCCATGTTCTTTCTTCTTTCTTTCTGTATGTATGTATGTATGTACGAATCAGCACCGCCATTCTTGTTTCGTCGTCCTCTCTTCCCAGTGCAACGTCGTGGAGCCGGCGGACGTCAAGAAGGTGGCCACCACCTTGCAGCGCGCCATCAAGGTGGTCGGCACGCCGGCGTACGAGGAGATGGTGAGGAACTGCATGATCCAGGATCTCTCCTGGAAGGTACGTACGCCCGCCCCGCCAGAGCAGAGCGCCAAGATCGATCGACCGACCGACCACACGTACGCGCCTCGCTCCTGTCGCTGACCGTGGTTTAATTTGCGAAATGCGCAGGGCCCTGCCAAGAACTGGGAGAACGTGCTGCTCAGCCTCGGGGTCGCTGGCGGCGAGCCAGGGGTCGAAGGCGAGGAGATCGCGCCGCTCGCCAAGGAGAACGTGGCCGCGCCCTGAAGAGTTCGGCCTGCAGGGCCCCTGATCTCGCGCGTGGTGCAAAGATGTTGGGACATCTTCTTATATATGCTGTTTCGTTTATGTGATATGGACAAGTATGTGTAGCTGCTTGCTTGTGCTAGTGTAATATAGTGTAGTGGTGGCCAGTGGCACAACCTAATAAGCGCATGAACTAATTGCTTGCGTGTGTAGTTAAGTACCGATCGGTAATTTTATAT |
| SWL310 | 99.10 | CCACAACTGTTCGCGTCCTGCTGGTTCATTATCTGACCTGATTGCATTATTGCAGCTACGAGAAGCCCGTGGAAGGCCGGAAGATCAACTGGATGAAGGCCGGGATCCTCGAGGCCGACAGGGTCCTCACCGTCAGCCCCTACTACGCCGAGGAGCTCATCTCCGGCATCGCCAGGGGCTGCGAGCTCGACAACATCATGCGCCTCACCGGCATCACCGGCATCGTCAACGGCATGGACGTCAGCGAGTGGGACCCCAGCAGGGACAAGTACATCGCCGTGAAGTACGACGTGTCGACGGTGAGCTGGCTAGCTAGCTGATTCTGCTGCCTGGTCCTCCTGCTCATGCTGGTTCGGTTCTGACGCGGCAAGTGTACGTACGTGCGTGCGACGGTGGTGTGGTGTCCGGTTCAGGCCGTGGAGGCCAAGGCGCTGAACAAGGAGGCGCTGCAGGCGGAGGTCGGGCTCCCGGTGGACCGGAACATCCCGCTGGTGGCGTTCATCGGCAGGCTGGAAGAGCAGAAGGGACCCGACGTCATGGCGGCCGCCATCCCGCAGCTCATGGAGATGGTGGAGGACGTGCAGATCGTTCTGCTGGTACGTGTGCGCCGCCCGCCACCCGGCTACTACATGCGTGTATCGTTCGTTCTACTGGAACATGCGTGTGAGCAACGCGATGGATAATGCTGCAGGGCACGGGCAAGAAGAAGTTCGAGCGCATGCTCATGAGCGCCGAGGAGAAGTTCCCAGGCAAGGTGCGCGCCGTGGTCAAGTTCAACGCGGCGCTGGCGCACCACATCATGGCCGGCGCCGACGTGCTCGCCGTCACCAGCCGCTTCGAGCCCTGCGGCCTCATCCAGCTGCAGGGGATGCGATACGGAACGGTACGAGAGAGAAAAAAAAACATCCTGAATCCTGACGAGAGGGACAGAGACAGATTGATTATGAATGCTTCATCGATTTGAATTGATTGATCGATGTCTCCCGCTGCGACTCTTGCAGCCCTGCGCCTGCGCGTCCACCGGTGGACTCGTCGACACCATCATCGAAGGCAAGACCGGGTTCCACATGGGCCGCCTCAGCGTCGACGTAAGCCTACCTCTGCCATGTTCTTTCTTCTTTCTTTCTGTATGTATGTATGTATGTACGAATCAGCACCGCCATTCTTGTTTCGTCGTCCTCTCTTCCCAGTGCAACGTCGTGGAGCCGGCGGACGTCAAGAAGGTGGCCACCACCTTGCAGCGCGCCATCAAGGTGGTCGGCACGCCGGCGTACGAGGAGATGGTGAGGAACTGCATGATCCAGGATCTCTCCTGGAAGGTACGTACGCCCGCCCCGCCAGAGCAGAGCGCCAAGATCGATCGACCGACCGACCACACGTACGCGCCTCGCTCCTGTCGCTGACCGTGGTTTAATTTGCGAAATGCGCAGGGCCCTGCCAAGAACTGGGAGAACGTGCTGCTCAGCCTCGGGGTCGCTGGCGGCGAGCCAGGGGTCGAAGGCGAGGAGATCGCGCCGCTCGCCAAGGAGAACGTGGCCGCGCCCTGAAGAGTTCGGCCTGCAGGGCCCCTGATCTCGCGCGTGGTGCAAAGATGTTGGGACATCTTCTTATATATGCTGTTTCGTTTATGTGATATGGACAAGTATGTGTAGCTGCTTGCTTGTGCTAGTGTAATATAGTGTAGTGGTGGCCAGTGGCACAACCTAATAAGCGCATGAACTAATTGCTTGCGTGTGTAGTTAAGTACCGATCGGTAATTTTATAT |

**Table S1 Continued**

| **Accession** | **AC(%)** | **DNA Sequence** |
| --- | --- | --- |
| SWL312 | 97.71 | CCACAACTGTTCGCGTCCTGCTGGTTCATTATCTGACCTGATTGCATTATTGCAGCTACGAGAAGCCCGTGGAAGGCCGGAAGATCAACTGGATGAAGGCCGGGATCCTCGAGGCCGACAGGGTCCTCACCGTCAGCCCCTACTACGCCGAGGAGCTCATCTCCGGCATCGCCAGGGGCTGCGAGCTCGACAACATCATGCGCCTCACCGGCATCACCGGCATCGTCAACGGCATGGACGTCAGCGAGTGGGACCCCAGCAGGGACAAGTACATCGCCGTGAAGTACGACGTGTCGACGGTGAGCTGGCTAGCTAGCTGATTCTGCTGCCTGGTCCTCCTGCTCATGCTGGTTCGGTTCTGACGCGGCAAGTGTACGTACGTGCGTGCGACGGTGGTGTGGTGTCCGGTTCAGGCCGTGGAGGCCAAGGCGCTGAACAAGGAGGCGCTGCAGGCGGAGGTCGGGCTCCCGGTGGACCGGAACATCCCGCTGGTGGCGTTCATCGGCAGGCTGGAAGAGCAGAAGGGACCCGACGTCATGGCGGCCGCCATCCCGCAGCTCATGGAGATGGTGGAGGACGTGCAGATCGTTCTGCTGGTACGTGTGCGCCGCCCGCCACCCGGCTACTACATGCGTGTATCGTTCGTTCTACTGGAACATGCGTGTGAGCAACGCGATGGATAATGCTGCAGGGCACGGGCAAGAAGAAGTTCGAGCGCATGCTCATGAGCGCCGAGGAGAAGTTCCCAGGCAAGGTGCGCGCCGTGGTCAAGTTCAACGCGGCGCTGGCGCACCACATCATGGCCGGCGCCGACGTGCTCGCCGTCACCAGCCGCTTCGAGCCCTGCGGCCTCATCCAGCTGCAGGGGATGCGATACGGAACGGTACGAGAGAGAAAAAAAAACATCCTGAATCCTGACGAGAGGGACAGAGACAGATTGATTATGAATGCTTCATCGATTTGAATTGATTGATCGATGTCTCCCGCTGCGACTCTTGCAGCCCTGCGCCTGCGCGTCCACCGGTGGACTCGTCGACACCATCATCGAAGGCAAGACCGGGTTCCACATGGGCCGCCTCAGCGTCGACGTAAGCCTACCTCTGCCATGTTCTTTCTTCTTTCTTTCTGTATGTATGTATGTATGTACGAATCAGCACCGCCATTCTTGTTTCGTCGTCCTCTCTTCCCAGTGCAACGTCGTGGAGCCGGCGGACGTCAAGAAGGTGGCCACCACCTTGCAGCGCGCCATCAAGGTGGTCGGCACGCCGGCGTACGAGGAGATGGTGAGGAACTGCATGATCCAGGATCTCTCCTGGAAGGTACGTACGCCCGCCCCGCCAGAGCAGAGCGCCAAGATCGATCGACCGACCGACCACACGTACGCGCCTCGCTCCTGTCGCTGACCGTGGTTTAATTTGCGAAATGCGCAGGGCCCTGCCAAGAACTGGGAGAACGTGCTGCTCAGCCTCGGGGTCGCTGGCGGCGAGCCAGGGGTCGAAGGCGAGGAGATCGCGCCGCTCGCCAAGGAGAACGTGGCCGCGCCCTGAAGAGTTCGGCCTGCAGGGCCCCTGATCTCGCGCGTGGTGCAAAGATGTTGGGACATCTTCTTATATATGCTGTTTCGTTTATGTGATATGGACAAGTATGTGTAGCTGCTTGCTTGTGCTAGTGTAATATAGTGTAGTGGTGGCCAGTGGCACAACCTAATAAGCGCATGAACTAATTGCTTGCGTGTGTAGTTAAGTACCGATCGGTAATTTTATAT |
| SWL324 | 98.34 | CCACAACTGTTCGCGTCCTGCTGGTTCATTATCTGACCTGATTGCATTATTGCAGCTACGAGAAGCCCGTGGAAGGCCGGAAGATCAACTGGATGAAGGCCGGGATCCTCGAGGCCGACAGGGTCCTCACCGTCAGCCCCTACTACGCCGAGGAGCTCATCTCCGGCATCGCCAGGGGCTGCGAGCTCGACAACATCATGCGCCTCACCGGCATCACCGGCATCGTCAACGGCATGGACGTCAGCGAGTGGGACCCCAGCAGGGACAAGTACATCGCCGTGAAGTACGACGTGTCGACGGTGAGCTGGCTAGCTAGCTGATTCTGCTGCCTGGTCCTCCTGCTCATGCTGGTTCGGTTCTGACGCGGCAAGTGTACGTACGTGCGTGCGACGGTGGTGTGGTGTCCGGTTCAGGCCGTGGAGGCCAAGGCGCTGAACAAGGAGGCGCTGCAGGCGGAGGTCGGGCTCCCGGTGGACCGGAACATCCCGCTGGTGGCGTTCATCGGCAGGCTGGAAGAGCAGAAGGGACCCGACGTCATGGCGGCCGCCATCCCGCAGCTCATGGAGATGGTGGAGGACGTGCAGATCGTTCTGCTGGTACGTGTGCGCCGCCCGCCACCCGGCTACTACATGCGTGTATCGTTCGTTCTACTGGAACATGCGTGTGAGCAACGCGATGGATAATGCTGCAGGGCACGGGCAAGAAGAAGTTCGAGCGCATGCTCATGAGCGCCGAGGAGAAGTTCCCAGGCAAGGTGCGCGCCGTGGTCAAGTTCAACGCGGCGCTGGCGCACCACATCATGGCCGGCGCCGACGTGCTCGCCGTCACCAGCCGCTTCGAGCCCTGCGGCCTCATCCAGCTGCAGGGGATGCGATACGGAACGGTACGAGAGAGAAAAAAAAACATCCTGAATCCTGACGAGAGGGACAGAGACAGATTGATTATGAATGCTTCATCGATTTGAATTGATTGATCGATGTCTCCCGCTGCGACTCTTGCAGCCCTGCGCCTGCGCGTCCACCGGTGGACTCGTCGACACCATCATCGAAGGCAAGACCGGGTTCCACATGGGCCGCCTCAGCGTCGACGTAAGCCTACCTCTGCCATGTTCTTTCTTCTTTCTTTCTGTATGTATGTATGTATGTACGAATCAGCACCGCCATTCTTGTTTCGTCGTCCTCTCTTCCCAGTGCAACGTCGTGGAGCCGGCGGACGTCAAGAAGGTGGCCACCACCTTGCAGCGCGCCATCAAGGTGGTCGGCACGCCGGCGTACGAGGAGATGGTGAGGAACTGCATGATCCAGGATCTCTCCTGGAAGGTACGTACGCCCGCCCCGCCAGAGCAGAGCGCCAAGATCGATCGACCGACCGACCACACGTACGCGCCTCGCTCCTGTCGCTGACCGTGGTTTAATTTGCGAAATGCGCAGGGCCCTGCCAAGAACTGGGAGAACGTGCTGCTCAGCCTCGGGGTCGCTGGCGGCGAGCCAGGGGTCGAAGGCGAGGAGATCGCGCCGCTCGCCAAGGAGAACGTGGCCGCGCCCTGAAGAGTTCGGCCTGCAGGGCCCCTGATCTCGCGCGTGGTGCAAAGATGTTGGGACATCTTCTTATATATGCTGTTTCGTTTATGTGATATGGACAAGTATGTGTAGCTGCTTGCTTGTGCTAGTGTAATATAGTGTAGTGGTGGCCAGTGGCACAACCTAATAAGCGCATGAACTAATTGCTTGCGTGTGTAGTTAAGTACCGATCGGTAATTTTATAT |

**Table S1 Continued**

| **Accession** | **AC(%)** | **DNA Sequence** |
| --- | --- | --- |
| SWL326 | 97.90 | CCACAACTGTTCGCGTCCTGCTGGTTCATTATCTGACCTGATTGCATTATTGCAGCTACGAGAAGCCCGTGGAAGGCCGGAAGATCAACTGGATGAAGGCCGGGATCCTCGAGGCCGACAGGGTCCTCACCGTCAGCCCCTACTACGCCGAGGAGCTCATCTCCGGCATCGCCAGGGGCTGCGAGCTCGACAACATCATGCGCCTCACCGGCATCACCGGCATCGTCAACGGCATGGACGTCAGCGAGTGGGACCCCAGCAGGGACAAGTACATCGCCGTGAAGTACGACGTGTCGACGGTGAGCTGGCTAGCTAGCTGATTCTGCTGCCTGGTCCTCCTGCTCATGCTGGTTCGGTTCTGACGCGGCAAGTGTACGTACGTGCGTGCGACGGTGGTGTGGTGTCCGGTTCAGGCCGTGGAGGCCAAGGCGCTGAACAAGGAGGCGCTGCAGGCGGAGGTCGGGCTCCCGGTGGACCGGAACATCCCGCTGGTGGCGTTCATCGGCAGGCTGGAAGAGCAGAAGGGACCCGACGTCATGGCGGCCGCCATCCCGCAGCTCATGGAGATGGTGGAGGACGTGCAGATCGTTCTGCTGGTACGTGTGCGCCGCCCGCCACCCGGCTACTACATGCGTGTATCGTTCGTTCTACTGGAACATGCGTGTGAGCAACGCGATGGATAATGCTGCAGGGCACGGGCAAGAAGAAGTTCGAGCGCATGCTCATGAGCGCCGAGGAGAAGTTCCCAGGCAAGGTGCGCGCCGTGGTCAAGTTCAACGCGGCGCTGGCGCACCACATCATGGCCGGCGCCGACGTGCTCGCCGTCACCAGCCGCTTCGAGCCCTGCGGCCTCATCCAGCTGCAGGGGATGCGATACGGAACGGTACGAGAGAGAAAAAAAAACATCCTGAATCCTGACGAGAGGGACAGAGACAGATTGATTATGAATGCTTCATCGATTTGAATTGATTGATCGATGTCTCCCGCTGCGACTCTTGCAGCCCTGCGCCTGCGCGTCCACCGGTGGACTCGTCGACACCATCATCGAAGGCAAGACCGGGTTCCACATGGGCCGCCTCAGCGTCGACGTAAGCCTACCTCTGCCATGTTCTTTCTTCTTTCTTTCTGTATGTATGTATGTATGTACGAATCAGCACCGCCATTCTTGTTTCGTCGTCCTCTCTTCCCAGTGCAACGTCGTGGAGCCGGCGGACGTCAAGAAGGTGGCCACCACCTTGCAGCGCGCCATCAAGGTGGTCGGCACGCCGGCGTACGAGGAGATGGTGAGGAACTGCATGATCCAGGATCTCTCCTGGAAGGTACGTACGCCCGCCCCGCCAGAGCAGAGCGCCAAGATCGATCGACCGACCGACCACACGTACGCGCCTCGCTCCTGTCGCTGACCGTGGTTTAATTTGCGAAATGCGCAGGGCCCTGCCAAGAACTGGGAGAACGTGCTGCTCAGCCTCGGGGTCGCTGGCGGCGAGCCAGGGGTCGAAGGCGAGGAGATCGCGCCGCTCGCCAAGGAGAACGTGGCCGCGCCCTGAAGAGTTCGGCCTGCAGGGCCCCTGATCTCGCGCGTGGTGCAAAGATGTTGGGACATCTTCTTATATATGCTGTTTCGTTTATGTGATATGGACAAGTATGTGTAGCTGCTTGCTTGTGCTAGTGTAATATAGTGTAGTGGTGGCCAGTGGCACAACCTAATAAGCGCATGAACTAATTGCTTGCGTGTGTAGTTAAGTACCGATCGGTAATTTTATAT |
| SWL328 | 96.66 | CCACAACTGTTCGCGTCCTGCTGGTTCATTATCTGACCTGATTGCATTATTGCAGCTACGAGAAGCCCGTGGAAGGCCGGAAGATCAACTGGATGAAGGCCGGGATCCTCGAGGCCGACAGGGTCCTCACCGTCAGCCCCTACTACGCCGAGGAGCTCATCTCCGGCATCGCCAGGGGCTGCGAGCTCGACAACATCATGCGCCTCACCGGCATCACCGGCATCGTCAACGGCATGGACGTCAGCGAGTGGGACCCCAGCAGGGACAAGTACATCGCCGTGAAGTACGACGTGTCGACGGTGAGCTGGCTAGCTAGCTGATTCTGCTGCCTGGTCCTCCTGCTCATGCTGGTTCGGTTCTGACGCGGCAAGTGTACGTACGTGCGTGCGACGGTGGTGTGGTGTCCGGTTCAGGCCGTGGAGGCCAAGGCGCTGAACAAGGAGGCGCTGCAGGCGGAGGTCGGGCTCCCGGTGGACCGGAACATCCCGCTGGTGGCGTTCATCGGCAGGCTGGAAGAGCAGAAGGGACCCGACGTCATGGCGGCCGCCATCCCGCAGCTCATGGAGATGGTGGAGGACGTGCAGATCGTTCTGCTGGTACGTGTGCGCCGCCCGCCACCCGGCTACTACATGCGTGTATCGTTCGTTCTACTGGAACATGCGTGTGAGCAACGCGATGGATAATGCTGCAGGGCACGGGCAAGAAGAAGTTCGAGCGCATGCTCATGAGCGCCGAGGAGAAGTTCCCAGGCAAGGTGCGCGCCGTGGTCAAGTTCAACGCGGCGCTGGCGCACCACATCATGGCCGGCGCCGACGTGCTCGCCGTCACCAGCCGCTTCGAGCCCTGCGGCCTCATCCAGCTGCAGGGGATGCGATACGGAACGGTACGAGAGAGAAAAAAAAACATCCTGAATCCTGACGAGAGGGACAGAGACAGATTGATTATGAATGCTTCATCGATTTGAATTGATTGATCGATGTCTCCCGCTGCGACTCTTGCAGCCCTGCGCCTGCGCGTCCACCGGTGGACTCGTCGACACCATCATCGAAGGCAAGACCGGGTTCCACATGGGCCGCCTCAGCGTCGACGTAAGCCTACCTCTGCCATGTTCTTTCTTCTTTCTTTCTGTATGTATGTATGTATGTACGAATCAGCACCGCCATTCTTGTTTCGTCGTCCTCTCTTCCCAGTGCAACGTCGTGGAGCCGGCGGACGTCAAGAAGGTGGCCACCACCTTGCAGCGCGCCATCAAGGTGGTCGGCACGCCGGCGTACGAGGAGATGGTGAGGAACTGCATGATCCAGGATCTCTCCTGGAAGGTACGTACGCCCGCCCCGCCAGAGCAGAGCGCCAAGATCGATCGACCGACCGACCACACGTACGCGCCTCGCTCCTGTCGCTGACCGTGGTTTAATTTGCGAAATGCGCAGGGCCCTGCCAAGAACTGGGAGAACGTGCTGCTCAGCCTCGGGGTCGCTGGCGGCGAGCCAGGGGTCGAAGGCGAGGAGATCGCGCCGCTCGCCAAGGAGAACGTGGCCGCGCCCTGAAGAGTTCGGCCTGCAGGGCCCCTGATCTCGCGCGTGGTGCAAAGATGTTGGGACATCTTCTTATATATGCTGTTTCGTTTATGTGATATGGACAAGTATGTGTAGCTGCTTGCTTGTGCTAGTGTAATATAGTGTAGTGGTGGCCAGTGGCACAACCTAATAAGCGCATGAACTAATTGCTTGCGTGTGTAGTTAAGTACCGATCGGTAATTTTATAT |

**Table S1 Continued**

| **Accession** | **AC(%)** | **DNA Sequence** |
| --- | --- | --- |
| SWL329 | 98.84 | CCACAACTGTTCGCGTCCTGCTGGTTCATTATCTGACCTGATTGCATTATTGCAGCTACGAGAAGCCCGTGGAAGGCCGGAAGATCAACTGGATGAAGGCCGGGATCCTCGAGGCCGACAGGGTCCTCACCGTCAGCCCCTACTACGCCGAGGAGCTCATCTCCGGCATCGCCAGGGGCTGCGAGCTCGACAACATCATGCGCCTCACCGGCATCACCGGCATCGTCAACGGCATGGACGTCAGCGAGTGGGACCCCAGCAGGGACAAGTACATCGCCGTGAAGTACGACGTGTCGACGGTGAGCTGGCTAGCTAGCTGATTCTGCTGCCTGGTCCTCCTGCTCATGCTGGTTCGGTTCTGACGCGGCAAGTGTACGTACGTGCGTGCGACGGTGGTGTGGTGTCCGGTTCAGGCCGTGGAGGCCAAGGCGCTGAACAAGGAGGCGCTGCAGGCGGAGGTCGGGCTCCCGGTGGACCGGAACATCCCGCTGGTGGCGTTCATCGGCAGGCTGGAAGAGCAGAAGGGACCCGACGTCATGGCGGCCGCCATCCCGCAGCTCATGGAGATGGTGGAGGACGTGCAGATCGTTCTGCTGGTACGTGTGCGCCGCCCGCCACCCGGCTACTACATGCGTGTATCGTTCGTTCTACTGGAACATGCGTGTGAGCAACGCGATGGATAATGCTGCAGGGCACGGGCAAGAAGAAGTTCGAGCGCATGCTCATGAGCGCCGAGGAGAAGTTCCCAGGCAAGGTGCGCGCCGTGGTCAAGTTCAACGCGGCGCTGGCGCACCACATCATGGCCGGCGCCGACGTGCTCGCCGTCACCAGCCGCTTCGAGCCCTGCGGCCTCATCCAGCTGCAGGGGATGCGATACGGAACGGTACGAGAGAGAAAAAAAAACATCCTGAATCCTGACGAGAGGGACAGAGACAGATTGATTATGAATGCTTCATCGATTTGAATTGATTGATCGATGTCTCCCGCTGCGACTCTTGCAGCCCTGCGCCTGCGCGTCCACCGGTGGACTCGTCGACACCATCATCGAAGGCAAGACCGGGTTCCACATGGGCCGCCTCAGCGTCGACGTAAGCCTACCTCTGCCATGTTCTTTCTTCTTTCTTTCTGTATGTATGTATGTATGTACGAATCAGCACCGCCATTCTTGTTTCGTCGTCCTCTCTTCCCAGTGCAACGTCGTGGAGCCGGCGGACGTCAAGAAGGTGGCCACCACCTTGCAGCGCGCCATCAAGGTGGTCGGCACGCCGGCGTACGAGGAGATGGTGAGGAACTGCATGATCCAGGATCTCTCCTGGAAGGTACGTACGCCCGCCCCGCCAGAGCAGAGCGCCAAGATCGATCGACCGACCGACCACACGTACGCGCCTCGCTCCTGTCGCTGACCGTGGTTTAATTTGCGAAATGCGCAGGGCCCTGCCAAGAACTGGGAGAACGTGCTGCTCAGCCTCGGGGTCGCTGGCGGCGAGCCAGGGGTCGAAGGCGAGGAGATCGCGCCGCTCGCCAAGGAGAACGTGGCCGCGCCCTGAAGAGTTCGGCCTGCAGGGCCCCTGATCTCGCGCGTGGTGCAAAGATGTTGGGACATCTTCTTATATATGCTGTTTCGTTTATGTGATATGGACAAGTATGTGTAGCTGCTTGCTTGTGCTAGTGTAATATAGTGTAGTGGTGGCCAGTGGCACAACCTAATAAGCGCATGAACTAATTGCTTGCGTGTGTAGTTAAGTACCGATCGGTAATTTTATAT |
| SWL333 | 99.21 | CCACAACTGTTCGCGTCCTGCTGGTTCATTATCTGACCTGATTGCATTATTGCAGCTACGAGAAGCCCGTGGAAGGCCGGAAGATCAACTGGATGAAGGCCGGGATCCTCGAGGCCGACAGGGTCCTCACCGTCAGCCCCTACTACGCCGAGGAGCTCATCTCCGGCATCGCCAGGGGCTGCGAGCTCGACAACATCATGCGCCTCACCGGCATCACCGGCATCGTCAACGGCATGGACGTCAGCGAGTGGGACCCCAGCAGGGACAAGTACATCGCCGTGAAGTACGACGTGTCGACGGTGAGCTGGCTAGCTAGCTGATTCTGCTGCCTGGTCCTCCTGCTCATGCTGGTTCGGTTCTGACGCGGCAAGTGTACGTACGTGCGTGCGACGGTGGTGTGGTGTCCGGTTCAGGCCGTGGAGGCCAAGGCGCTGAACAAGGAGGCGCTGCAGGCGGAGGTCGGGCTCCCGGTGGACCGGAACATCCCGCTGGTGGCGTTCATCGGCAGGCTGGAAGAGCAGAAGGGACCCGACGTCATGGCGGCCGCCATCCCGCAGCTCATGGAGATGGTGGAGGACGTGCAGATCGTTCTGCTGGTACGTGTGCGCCGCCCGCCACCCGGCTACTACATGCGTGTATCGTTCGTTCTACTGGAACATGCGTGTGAGCAACGCGATGGATAATGCTGCAGGGCACGGGCAAGAAGAAGTTCGAGCGCATGCTCATGAGCGCCGAGGAGAAGTTCCCAGGCAAGGTGCGCGCCGTGGTCAAGTTCAACGCGGCGCTGGCGCACCACATCATGGCCGGCGCCGACGTGCTCGCCGTCACCAGCCGCTTCGAGCCCTGCGGCCTCATCCAGCTGCAGGGGATGCGATACGGAACGGTACGAGAGAGAAAAAAAAACATCCTGAATCCTGACGAGAGGGACAGAGACAGATTGATTATGAATGCTTCATCGATTTGAATTGATTGATCGATGTCTCCCGCTGCGACTCTTGCAGCCCTGCGCCTGCGCGTCCACCGGTGGACTCGTCGACACCATCATCGAAGGCAAGACCGGGTTCCACATGGGCCGCCTCAGCGTCGACGTAAGCCTACCTCTGCCATGTTCTTTCTTCTTTCTTTCTGTATGTATGTATGTATGTACGAATCAGCACCGCCATTCTTGTTTCGTCGTCCTCTCTTCCCAGTGCAACGTCGTGGAGCCGGCGGACGTCAAGAAGGTGGCCACCACCTTGCAGCGCGCCATCAAGGTGGTCGGCACGCCGGCGTACGAGGAGATGGTGAGGAACTGCATGATCCAGGATCTCTCCTGGAAGGTACGTACGCCCGCCCCGCCAGAGCAGAGCGCCAAGATCGATCGACCGACCGACCACACGTACGCGCCTCGCTCCTGTCGCTGACCGTGGTTTAATTTGCGAAATGCGCAGGGCCCTGCCAAGAACTGGGAGAACGTGCTGCTCAGCCTCGGGGTCGCTGGCGGCGAGCCAGGGGTCGAAGGCGAGGAGATCGCGCCGCTCGCCAAGGAGAACGTGGCCGCGCCCTGAAGAGTTCGGCCTGCAGGGCCCCTGATCTCGCGCGTGGTGCAAAGATGTTGGGACATCTTCTTATATATGCTGTTTCGTTTATGTGATATGGACAAGTATGTGTAGCTGCTTGCTTGTGCTAGTGTAATATAGTGTAGTGGTGGCCAGTGGCACAACCTAATAAGCGCATGAACTAATTGCTTGCGTGTGTAGTTAAGTACCGATCGGTAATTTTATAT |

**Table S1 Continued**

| **Accession** | **AC(%)** | **DNA Sequence** |
| --- | --- | --- |
| SWL335 | 99.37 | CCACAACTGTTCGCGTCCTGCTGGTTCATTATCTGACCTGATTGCATTATTGCAGCTACGAGAAGCCCGTGGAAGGCCGGAAGATCAACTGGATGAAGGCCGGGATCCTCGAGGCCGACAGGGTCCTCACCGTCAGCCCCTACTACGCCGAGGAGCTCATCTCCGGCATCGCCAGGGGCTGCGAGCTCGACAACATCATGCGCCTCACCGGCATCACCGGCATCGTCAACGGCATGGACGTCAGCGAGTGGGACCCCAGCAGGGACAAGTACATCGCCGTGAAGTACGACGTGTCGACGGTGAGCTGGCTAGCTAGCTGATTCTGCTGCCTGGTCCTCCTGCTCATGCTGGTTCGGTTCTGACGCGGCAAGTGTACGTACGTGCGTGCGACGGTGGTGTGGTGTCCGGTTCAGGCCGTGGAGGCCAAGGCGCTGAACAAGGAGGCGCTGCAGGCGGAGGTCGGGCTCCCGGTGGACCGGAACATCCCGCTGGTGGCGTTCATCGGCAGGCTGGAAGAGCAGAAGGGACCCGACGTCATGGCGGCCGCCATCCCGCAGCTCATGGAGATGGTGGAGGACGTGCAGATCGTTCTGCTGGTACGTGTGCGCCGCCCGCCACCCGGCTACTACATGCGTGTATCGTTCGTTCTACTGGAACATGCGTGTGAGCAACGCGATGGATAATGCTGCAGGGCACGGGCAAGAAGAAGTTCGAGCGCATGCTCATGAGCGCCGAGGAGAAGTTCCCAGGCAAGGTGCGCGCCGTGGTCAAGTTCAACGCGGCGCTGGCGCACCACATCATGGCCGGCGCCGACGTGCTCGCCGTCACCAGCCGCTTCGAGCCCTGCGGCCTCATCCAGCTGCAGGGGATGCGATACGGAACGGTACGAGAGAGAAAAAAAAACATCCTGAATCCTGACGAGAGGGACAGAGACAGATTGATTATGAATGCTTCATCGATTTGAATTGATTGATCGATGTCTCCCGCTGCGACTCTTGCAGCCCTGCGCCTGCGCGTCCACCGGTGGACTCGTCGACACCATCATCGAAGGCAAGACCGGGTTCCACATGGGCCGCCTCAGCGTCGACGTAAGCCTACCTCTGCCATGTTCTTTCTTCTTTCTTTCTGTATGTATGTATGTATGTACGAATCAGCACCGCCATTCTTGTTTCGTCGTCCTCTCTTCCCAGTGCAACGTCGTGGAGCCGGCGGACGTCAAGAAGGTGGCCACCACCTTGCAGCGCGCCATCAAGGTGGTCGGCACGCCGGCGTACGAGGAGATGGTGAGGAACTGCATGATCCAGGATCTCTCCTGGAAGGTACGTACGCCCGCCCCGCCAGAGCAGAGCGCCAAGATCGATCGACCGACCGACCACACGTACGCGCCTCGCTCCTGTCGCTGACCGTGGTTTAATTTGCGAAATGCGCAGGGCCCTGCCAAGAACTGGGAGAACGTGCTGCTCAGCCTCGGGGTCGCTGGCGGCGAGCCAGGGGTCGAAGGCGAGGAGATCGCGCCGCTCGCCAAGGAGAACGTGGCCGCGCCCTGAAGAGTTCGGCCTGCAGGGCCCCTGATCTCGCGCGTGGTGCAAAGATGTTGGGACATCTTCTTATATATGCTGTTTCGTTTATGTGATATGGACAAGTATGTGTAGCTGCTTGCTTGTGCTAGTGTAATATAGTGTAGTGGTGGCCAGTGGCACAACCTAATAAGCGCATGAACTAATTGCTTGCGTGTGTAGTTAAGTACCGATCGGTAATTTTATAT |
| SWL337 | 98.60 | CCACAACTGTTCGCGTCCTGCTGGTTCATTATCTGACCTGATTGCATTATTGCAGCTACGAGAAGCCCGTGGAAGGCCGGAAGATCAACTGGATGAAGGCCGGGATCCTCGAGGCCGACAGGGTCCTCACCGTCAGCCCCTACTACGCCGAGGAGCTCATCTCCGGCATCGCCAGGGGCTGCGAGCTCGACAACATCATGCGCCTCACCGGCATCACCGGCATCGTCAACGGCATGGACGTCAGCGAGTGGGACCCCAGCAGGGACAAGTACATCGCCGTGAAGTACGACGTGTCGACGGTGAGCTGGCTAGCTAGCTGATTCTGCTGCCTGGTCCTCCTGCTCATGCTGGTTCGGTTCTGACGCGGCAAGTGTACGTACGTGCGTGCGACGGTGGTGTGGTGTCCGGTTCAGGCCGTGGAGGCCAAGGCGCTGAACAAGGAGGCGCTGCAGGCGGAGGTCGGGCTCCCGGTGGACCGGAACATCCCGCTGGTGGCGTTCATCGGCAGGCTGGAAGAGCAGAAGGGACCCGACGTCATGGCGGCCGCCATCCCGCAGCTCATGGAGATGGTGGAGGACGTGCAGATCGTTCTGCTGGTACGTGTGCGCCGCCCGCCACCCGGCTACTACATGCGTGTATCGTTCGTTCTACTGGAACATGCGTGTGAGCAACGCGATGGATAATGCTGCAGGGCACGGGCAAGAAGAAGTTCGAGCGCATGCTCATGAGCGCCGAGGAGAAGTTCCCAGGCAAGGTGCGCGCCGTGGTCAAGTTCAACGCGGCGCTGGCGCACCACATCATGGCCGGCGCCGACGTGCTCGCCGTCACCAGCCGCTTCGAGCCCTGCGGCCTCATCCAGCTGCAGGGGATGCGATACGGAACGGTACGAGAGAGAAAAAAAAACATCCTGAATCCTGACGAGAGGGACAGAGACAGATTGATTATGAATGCTTCATCGATTTGAATTGATTGATCGATGTCTCCCGCTGCGACTCTTGCAGCCCTGCGCCTGCGCGTCCACCGGTGGACTCGTCGACACCATCATCGAAGGCAAGACCGGGTTCCACATGGGCCGCCTCAGCGTCGACGTAAGCCTACCTCTGCCATGTTCTTTCTTCTTTCTTTCTGTATGTATGTATGTATGTACGAATCAGCACCGCCATTCTTGTTTCGTCGTCCTCTCTTCCCAGTGCAACGTCGTGGAGCCGGCGGACGTCAAGAAGGTGGCCACCACCTTGCAGCGCGCCATCAAGGTGGTCGGCACGCCGGCGTACGAGGAGATGGTGAGGAACTGCATGATCCAGGATCTCTCCTGGAAGGTACGTACGCCCGCCCCGCCAGAGCAGAGCGCCAAGATCGATCGACCGACCGACCACACGTACGCGCCTCGCTCCTGTCGCTGACCGTGGTTTAATTTGCGAAATGCGCAGGGCCCTGCCAAGAACTGGGAGAACGTGCTGCTCAGCCTCGGGGTCGCTGGCGGCGAGCCAAGGGTCGAAGGCTAGGAGATCGCGCCGCTCGCCAAGGAGAACGTGGCCGCGCCCTGAAGAGTTCGGCCTGCAGGGCCCCTGATCTCGCGCGTGGTGCAAAGATGTTGGGACATCTTCTTATATATGCTGTTTCGTTTATGTGATATGGACAAGTATGTGTAGCTGCTTGCTTGTGCTAGTGTAATATAGTGTAGTGGTGGCCAGTGGCACAACCTAATAAGCGCATGAACTAATTGCTTGCGTGTGTAGTTAAGTACCGATCGGTAATTTTATAT |

**Table S1 Continued**

| **Accession** | **AC(%)** | **DNA Sequence** |
| --- | --- | --- |
| SWL339 | 99.60 | CCACAACTGTTCGCGTCCTGCTGGTTCATTATCTGACCTGATTGCATTATTGCAGCTACGAGAAGCCCGTGGAAGGCCGGAAGATCAACTGGATGAAGGCCGGGATCCTCGAGGCCGACAGGGTCCTCACCGTCAGCCCCTACTACGCCGAGGAGCTCATCTCCGGCATCGCCAGGGGCTGCGAGCTCGACAACATCATGCGCCTCACCGGCATCACCGGCATCGTCAACGGCATGGACGTCAGCGAGTGGGACCCCAGCAGGGACAAGTACATCGCCGTGAAGTACGACGTGTCGACGGTGAGCTGGCTAGCTAGCTGATTCTGCTGCCTGGTCCTCCTGCTCATGCTGGTTCGGTTCTGACGCGGCAAGTGTACGTACGTGCGTGCGACGGTGGTGTGGTGTCCGGTTCAGGCCGTGGAGGCCAAGGCGCTGAACAAGGAGGCGCTGCAGGCGGAGGTCGGGCTCCCGGTGGACCGGAACATCCCGCTGGTGGCGTTCATCGGCAGGCTGGAAGAGCAGAAGGGACCCGACGTCATGGCGGCCGCCATCCCGCAGCTCATGGAGATGGTGGAGGACGTGCAGATCGTTCTGCTGGTACGTGTGCGCCGCCCGCCACCCGGCTACTACATGCGTGTATCGTTCGTTCTACTGGAACATGCGTGTGAGCAACGCGATGGATAATGCTGCAGGGCACGGGCAAGAAGAAGTTCGAGCGCATGCTCATGAGCGCCGAGGAGAAGTTCCCAGGCAAGGTGCGCGCCGTGGTCAAGTTCAACGCGGCGCTGGCGCACCACATCATGGCCGGCGCCGACGTGCTCGCCGTCACCAGCCGCTTCGAGCCCTGCGGCCTCATCCAGCTGCAGGGGATGCGATACGGAACGGTACGAGAGAGAAAAAAAAACATCCTGAATCCTGACGAGAGGGACAGAGACAGATTGATTATGAATGCTTCATCGATTTGAATTGATTGATCGATGTCTCCCGCTGCGACTCTTGCAGCCCTGCGCCTGCGCGTCCACCGGTGGACTCGTCGACACCATCATCGAAGGCAAGACCGGGTTCCACATGGGCCGCCTCAGCGTCGACGTAAGCCTACCTCTGCCATGTTCTTTCTTCTTTCTTTCTGTATGTATGTATGTATGTACGAATCAGCACCGCCATTCTTGTTTCGTCGTCCTCTCTTCCCAGTGCAACGTCGTGGAGCCGGCGGACGTCAAGAAGGTGGCCACCACCTTGCAGCGCGCCATCAAGGTGGTCGGCACGCCGGCGTACGAGGAGATGGTGAGGAACTGCATGATCCAGGATCTCTCCTGGAAGGTACGTACGCCCGCCCCGCCAGAGCAGAGCGCCAAGATCGATCGACCGACCGACCACACGTACGCGCCTCGCTCCTGTCGCTGACCGTGGTTTAATTTGCGAAATGCGCAGGGCCCTGCCAAGAACTGGGAGAACGTGCTGCTCAGCCTCGGGGTCGCTGGCGGCGAGCCAGGGGTCGAAGGCGAGGAGATCGCGCCGCTCGCCAAGGAGAACGTGGCCGCGCCCTGAAGAGTTCGGCCTGCAGGGCCCCTGATCTCGCGCGTGGTGCAAAGATGTTGGGACATCTTCTTATATATGCTGTTTCGTTTATGTGATATGGACAAGTATGTGTAGCTGCTTGCTTGTGCTAGTGTAATATAGTGTAGTGGTGGCCAGTGGCACAACCTAATAAGCGCATGAACTAATTGCTTGCGTGTGTAGTTAAGTACCGATCGGTAATTTTATAT |
| SWL343 | 98.58 | CCACAACTGTTCGCGTCCTGCTGGTTCATTATCTGACCTGATTGCATTATTGCAGCTACGAGAAGCCCGTGGAAGGCCGGAAGATCGTAACCCACAAACTGGATGAAGGCCGGGATCCTCGAGGCCGACAGGGTCCTCACCGTCAGCCCCTACTACGCCGAGGAGCTCATCTCCGGCATCGCCAGGGGCTGCGAGCTCGACAACATCATGCGCCTCACCGGCATCACCGGCATCGTCAACGGCATGGACGTCAGCGAGTGGGACCCCAGCAGGGACAAGTACATCGCCGTGAAGTACGACGTGTCGACGGTGAGCTGGCTAGCTAGCTGATTCTGCTGCCTGGTCCTCCTGCTCATGCTGGTTCGGTTCTGACGCGGCAAGTGTACGTACGTGCGTGCGACGGTGGTGTGGTGTCCGGTTCAGGCCGTGGAGGCCAAGGCGCTGAACAAGGAGGCGCTGCAGGCGGAGGTCGGGCTCCCGGTGGACCGGAACATCCCGCTGGTGGCGTTCATCGGCAGGCTGGAAGAGCAGAAGGGACCCGACGTCATGGCGGCCGCCATCCCGCAGCTCATGGAGATGGTGGAGGACGTGCAGATCGTTCTGCTGGTACGTGTGCGCCGCCCGCCACCCGGCTACTACATGCGTGTATCGTTCGTTCTACTGGAACATGCGTGTGAGCAACGCGATGGATAATGCTGCAGGGCACGGGCAAGAAGAAGTTCGAGCGCATGCTCATGAGCGCCGAGGAGAAGTTCCCAGGCAAGGTGCGCGCCGTGGTCAAGTTCAACGCGGCGCTGGCGCACCACATCATGGCCGGCGCCGACGTGCTCGCCGTCACCAGCCGCTTCGAGCCCTGCGGCCTCATCCAGCTGCAGGGGATGCGATACGGAACGGTACGAGAGAGAAAAAAAAACATCCTGAATCCTGACGAGAGGGACAGAGACAGATTGATTATGAATGCTTCATCGATTTGAATTGATTGATCGATGTCTCCCGCTGCGACTCTTGCAGCCCTGCGCCTGCGCGTCCACCGGTGGACTCGTCGACACCATCATCGAAGGCAAGACCGGGTTCCACATGGGCCGCCTCAGCGTCGACGTAAGCCTACCTCTGCCATGTTCTTTCTTCTTTCTTTCTGTATGTATGTATGTATGTACGAATCAGCACCGCCATTCTTGTTTCGTCGTCCTCTCTTCCCAGTGCAACGTCGTGGAGCCGGCGGACGTCAAGAAGGTGGCCACCACCTTGCAGCGCGCCATCAAGGTGGTCGGCACGCCGGCGTACGAGGAGATGGTGAGGAACTGCATGATCCAGGATCTCTCCTGGAAGGTACGTACGCCCGCCCCGCCAGAGCAGAGCGCCAAGATCGATCGACCGACCGACCACACGTACGCGCCTCGCTCCTGTCGCTGACCGTGGTTTAATTTGCGAAATGCGCAGGGCCCTGCCAAGAACTGGGAGAACGTGCTGCTCAGCCTCGGGGTCGCTGGCGGCGAGCCAGGGGTCGAAGGCGAGGAGATCGCGCCGCTCGCCAAGGAGAACGTGGCCGCGCCCTGAAGAGTTCGGCCTGCAGGGCCCCTGATCTCGCGCGTGGTGCAAAGATGTTGGGACATCTTCTTATATATGCTGTTTCGTTTATGTGATATGGACAAGTATGTGTAGCTGCTTGCTTGTGCTAGTGTAATATAGTGTAGTGGTGGCCAGTGGCACAACCTAATAAGCGCATGAACTAATTGCTTGCGTGTGTAGTTAAGTACCGATCGGTAATTTTATAT |

**Table S1 Continued**

| **Accession** | **AC(%)** | **DNA Sequence** |
| --- | --- | --- |
| SWL345 | 99.18 | CCACAACTGTTCGCGTCCTGCTGGTTCATTATCTGACCTGATTGCATTATTGCAGCTACGAGAAGCCCGTGGAAGGCCGGAAGATCAACTGGATGAAGGCCGGGATCCTCGAGGCCGACAGGGTCCTCACCGTCAGCCCCTACTACGCCGAGGAGCTCATCTCCGGCATCGCCAGGGGCTGCGAGCTCGACAACATCATGCGCCTCACCGGCATCACCGGCATCGTCAACGGCATGGACGTCAGCGAGTGGGACCCCAGCAGGGACAAGTACATCGCCGTGAAGTACGACGTGTCGACGGTGAGCTGGCTAGCTAGCTGATTCTGCTGCCTGGTCCTCCTGCTCATGCTGGTTCGGTTCTGACGCGGCAAGTGTACGTACGTGCGTGCGACGGTGGTGTGGTGTCCGGTTCAGGCCGTGGAGGCCAAGGCGCTGAACAAGGAGGCGCTGCAGGCGGAGGTCGGGCTCCCGGTGGACCGGAACATCCCGCTGGTGGCGTTCATCGGCAGGCTGGAAGAGCAGAAGGGACCCGACGTCATGGCGGCCGCCATCCCGCAGCTCATGGAGATGGTGGAGGACGTGCAGATCGTTCTGCTGGTACGTGTGCGCCGCCCGCCACCCGGCTACTACATGCGTGTATCGTTCGTTCTACTGGAACATGCGTGTGAGCAACGCGATGGATAATGCTGCAGGGCACGGGCAAGAAGAAGTTCGAGCGCATGCTCATGAGCGCCGAGGAGAAGTTCCCAGGCAAGGTGCGCGCCGTGGTCAAGTTCAACGCGGCGCTGGCGCACCACATCATGGCCGGCGCCGACGTGCTCGCCGTCACCAGCCGCTTCGAGCCCTGCGGCCTCATCCAGCTGCAGGGGATGCGATACGGAACGGTACGAGAGAGAAAAAAAAACATCCTGAATCCTGACGAGAGGGACAGAGACAGATTGATTATGAATGCTTCATCGATTTGAATTGATTGATCGATGTCTCCCGCTGCGACTCTTGCAGCCCTGCGCCTGCGCGTCCACCGGTGGACTCGTCGACACCATCATCGAAGGCAAGACCGGGTTCCACATGGGCCGCCTCAGCGTCGACGTAAGCCTACCTCTGCCATGTTCTTTCTTCTTTCTTTCTGTATGTATGTATGTATGTACGAATCAGCACCGCCATTCTTGTTTCGTCGTCCTCTCTTCCCAGTGCAACGTCGTGGAGCCGGCGGACGTCAAGAAGGTGGCCACCACCTTGCAGCGCGCCATCAAGGTGGTCGGCACGCCGGCGTACGAGGAGATGGTGAGGAACTGCATGATCCAGGATCTCTCCTGGAAGGTACGTACGCCCGCCCCGCCAGAGCAGAGCGCCAAGATCGATCGACCGACCGACCACACGTACGCGCCTCGCTCCTGTCGCTGACCGTGGTTTAATTTGCGAAATGCGCAGGGCCCTGCCAAGAACTGGGAGAACGTGCTGCTCAGCCTCGGGGTCGCTGGCGGCGAGCCAGGGGTCGAAGGCGAGGAGATCGCGCCGCTCGCCAAGGAGAACGTGGCCGCGCCCTGAAGAGTTCGGCCTGCAGGGCCCCTGATCTCGCGCGTGGTGCAAAGATGTTGGGACATCTTCTTATATATGCTGTTTCGTTTATGTGATATGGACAAGTATGTGTAGCTGCTTGCTTGTGCTAGTGTAATATAGTGTAGTGGTGGCCAGTGGCACAACCTAATAAGCGCATGAACTAATTGCTTGCGTGTGTAGTTAAGTACCGATCGGTAATTTTATAT |
| SWL348 | 98.82 | CCACAACTGTTCGCGTCCTGCTGGTTCATTATCTGACCTGATTGCATTATTGCAGCTACGAGAAGCCCGTGGAAGGCCGGAAGATCAACTGGATGAAGGCCGGGATCCTCGAGGCCGACAGGGTCCTCACCGTCAGCCCCTACTACGCCGAGGAGCTCATCTCCGGCATCGCCAGGGGCTGCGAGCTCGACAACATCATGCGCCTCACCGGCATCACCGGCATCGTCAACGGCATGGACGTCAGCGAGTGGGACCCCAGCAGGGACAAGTACATCGCCGTGAAGTACGACGTGTCGACGGTGAGCTGGCTAGCTAGCTGATTCTGCTGCCTGGTCCTCCTGCTCATGCTGGTTCGGTTCTGACGCGGCAAGTGTACGTACGTGCGTGCGACGGTGGTGTGGTGTCCGGTTCAGGCCGTGGAGGCCAAGGCGCTGAACAAGGAGGCGCTGCAGGCGGAGGTCGGGCTCCCGGTGGACCGGAACATCCCGCTGGTGGCGTTCATCGGCAGGCTGGAAGAGCAGAAGGGACCCGACGTCATGGCGGCCGCCATCCCGCAGCTCATGGAGATGGTGGAGGACGTGCAGATCGTTCTGCTGGTACGTGTGCGCCGCCCGCCACCCGGCTACTACATGCGTGTATCGTTCGTTCTACTGGAACATGCGTGTGAGCAACGCGATGGATAATGCTGCAGGGCACGGGCAAGAAGAAGTTCGAGCGCATGCTCATGAGCGCCGAGGAGAAGTTCCCAGGCAAGGTGCGCGCCGTGGTCAAGTTCAACGCGGCGCTGGCGCACCACATCATGGCCGGCGCCGACGTGCTCGCCGTCACCAGCCGCTTCGAGCCCTGCGGCCTCATCCAGCTGCAGGGGATGCGATACGGAACGGTACGAGAGAGAAAAAAAAACATCCTGAATCCTGACGAGAGGGACAGAGACAGATTGATTATGAATGCTTCATCGATTTGAATTGATTGATCGATGTCTCCCGCTGCGACTCTTGCAGCCCTGCGCCTGCGCGTCCACCGGTGGACTCGTCGACACCATCATCGAAGGCAAGACCGGGTTCCACATGGGCCGCCTCAGCGTCGACGTAAGCCTACCTCTGCCATGTTCTTTCTTCTTTCTTTCTGTATGTATGTATGTATGTACGAATCAGCACCGCCATTCTTGTTTCGTCGTCCTCTCTTCCCAGTGCAACGTCGTGGAGCCGGCGGACGTCAAGAAGGTGGCCACCACCTTGCAGCGCGCCATCAAGGTGGTCGGCACGCCGGCGTACGAGGAGATGGTGAGGAACTGCATGATCCAGGATCTCTCCTGGAAGGTACGTACGCCCGCCCCGCCAGAGCAGAGCGCCAAGATCGATCGACCGACCGACCACACGTACGCGCCTCGCTCCTGTCGCTGACCGTGGTTTAATTTGCGAAATGCGCAGGGCCCTGCCAAGAACTGGGAGAACGTGCTGCTCAGCCTCGGGGTCGCTGGCGGCGAGCCAGGGGTCGAAGGCGAGGAGATCGCGCCGCTCGCCAAGGAGAACGTGGCCGCGCCCTGAAGAGTTCGGCCTGCAGGGCCCCTGATCTCGCGCGTGGTGCAAAGATGTTGGGACATCTTCTTATATATGCTGTTTCGTTTATGTGATATGGACAAGTATGTGTAGCTGCTTGCTTGTGCTAGTGTAATATAGTGTAGTGGTGGCCAGTGGCACAACCTAATAAGCGCATGAACTAATTGCTTGCGTGTGTAGTTAAGTACCGATCGGTAATTTTATAT |

**Table S1 Continued**

| **Accession** | **AC(%)** | **DNA Sequence** |
| --- | --- | --- |
| SWL359 | 98.46 | CCACAACTGTTCGCGTCCTGCTGGTTCATTATCTGACCTGATTGCATTATTGCAGCTACGAGAAGCCCGTGGAAGGCCGGAAGATCAACTGGATGAAGGCCGGGATCCTCGAGGCCGACAGGGTCCTCACCGTCAGCCCCTACTACGCCGAGGAGCTCATCTCCGGCATCGCCAGGGGCTGCGAGCTCGACAACATCATGCGCCTCACCGGCATCACCGGCATCGTCAACGGCATGGACGTCAGCGAGTGGGACCCCAGCAGGGACAAGTACATCGCCGTGAAGTACGACGTGTCGACGGTGAGCTGGCTAGCTAGCTGATTCTGCTGCCTGGTCCTCCTGCTCATGCTGGTTCGGTTCTGACGCGGCAAGTGTACGTACGTGCGTGCGACGGTGGTGTGGTGTCCGGTTCAGGCCGTGGAGGCCAAGGCGCTGAACAAGGAGGCGCTGCAGGCGGAGGTCGGGCTCCCGGTGGACCGGAACATCCCGCTGGTGGCGTTCATCGGCAGGCTGGAAGAGCAGAAGGGACCCGACGTCATGGCGGCCGCCATCCCGCAGCTCATGGAGATGGTGGAGGACGTGCAGATCGTTCTGCTGGTACGTGTGCGCCGCCCGCCACCCGGCTACTACATGCGTGTATCGTTCGTTCTACTGGAACATGCGTGTGAGCAACGCGATGGATAATGCTGCAGGGCACGGGCAAGAAGAAGTTCGAGCGCATGCTCATGAGCGCCGAGGAGAAGTTCCCAGGCAAGGTGCGCGCCGTGGTCAAGTTCAACGCGGCGCTGGCGCACCACATCATGGCCGGCGCCGACGTGCTCGCCGTCACCAGCCGCTTCGAGCCCTGCGGCCTCATCCAGCTGCAGGGGATGCGATACGGAACGGTACGAGAGAGAAAAAAAAACATCCTGAATCCTGACGAGAGGGACAGAGACAGATTGATTATGAATGCTTCATCGATTTGAATTGATTGATCGATGTCTCCCGCTGCGACTCTTGCAGCCCTGCGCCTGCGCGTCCACCGGTGGACTCGTCGACACCATCATCGAAGGCAAGACCGGGTTCCACATGGGCCGCCTCAGCGTCGACGTAAGCCTACCTCTGCCATGTTCTTTCTTCTTTCTTTCTGTATGTATGTATGTATGTACGAATCAGCACCGCCATTCTTGTTTCGTCGTCCTCTCTTCCCAGTGCAACGTCGTGGAGCCGGCGGACGTCAAGAAGGTGGCCACCACCTTGCAGCGCGCCATCAAGGTGGTCGGCACGCCGGCGTACGAGGAGATGGTGAGGAACTGCATGATCCAGGATCTCTCCTGGAAGGTACGTACGCCCGCCCCGCCAGAGCAGAGCGCCAAGATCGATCGACCGACCGACCACACGTACGCGCCTCGCTCCTGTCGCTGACCGTGGTTTAATTTGCGAAATGCGCAGGGCCCTGCCAAGAACTGGGAGAACGTGCTGCTCAGCCTCGGGGTCGCTGGCGGCGAGCCAGGGGTCGAAGGCGAGGAGATCGCGCCGCTCGCCAAGGAGAACGTGGCCGCGCCCTGAAGAGTTCGGCCTGCAGGGCCCCTGATCTCGCGCGTGGTGCAAAGATGTTGGGACATCTTCTTATATATGCTGTTTCGTTTATGTGATATGGACAAGTATGTGTAGCTGCTTGCTTGTGCTAGTGTAATATAGTGTAGTGGTGGCCAGTGGCACAACCTAATAAGCGCATGAACTAATTGCTTGCGTGTGTAGTTAAGTACCGATCGGTAATTTTATAT |
| SWL362 | 98.68 | CCACAACTGTTCGCGTCCTGCTGGTTCATTATCTGACCTGATTGCATTATTGCAGCTACGAGAAGCCCGTGGAAGGCCGGAAGATCAACTGGATGAAGGCCGGGATCCTCGAGGCCGACAGGGTCCTCACCGTCAGCCCCTACTACGCCGAGGAGCTCATCTCCGGCATCGCCAGGGGCTGCGAGCTCGACAACATCATGCGCCTCACCGGCATCACCGGCATCGTCAACGGCATGGACGTCAGCGAGTGGGACCCCAGCAGGGACAAGTACATCGCCGTGAAGTACGACGTGTCGACGGTGAGCTGGCTAGCTAGCTGATTCTGCTGCCTGGTCCTCCTGCTCATGCTGGTTCGGTTCTGACGCGGCAAGTGTACGTACGTGCGTGCGACGGTGGTGTGGTGTCCGGTTCAGGCCGTGGAGGCCAAGGCGCTGAACAAGGAGGCGCTGCAGGCGGAGGTCGGGCTCCCGGTGGACCGGAACATCCCGCTGGTGGCGTTCATCGGCAGGCTGGAAGAGCAGAAGGGACCCGACGTCATGGCGGCCGCCATCCCGCAGCTCATGGAGATGGTGGAGGACGTGCAGATCGTTCTGCTGGTACGTGTGCGCCGCCCGCCACCCGGCTACTACATGCGTGTATCGTTCGTTCTACTGGAACATGCGTGTGAGCAACGCGATGGATAATGCTGCAGGGCACGGGCAAGAAGAAGTTCGAGCGCATGCTCATGAGCGCCGAGGAGAAGTTCCCAGGCAAGGTGCGCGCCGTGGTCAAGTTCAACGCGGCGCTGGCGCACCACATCATGGCCGGCGCCGACGTGCTCGCCGTCACCAGCCGCTTCGAGCCCTGCGGCCTCATCCAGCTGCAGGGGATGCGATACGGAACGGTACGAGAGAGAAAAAAAAACATCCTGAATCCTGACGAGAGGGACAGAGACAGATTGATTATGAATGCTTCATCGATTTGAATTGATTGATCGATGTCTCCCGCTGCGACTCTTGCAGCCCTGCGCCTGCGCGTCCACCGGTGGACTCGTCGACACCATCATCGAAGGCAAGACCGGGTTCCACATGGGCCGCCTCAGCGTCGACGTAAGCCTACCTCTGCCATGTTCTTTCTTCTTTCTTTCTGTATGTATGTATGTATGTACGAATCAGCACCGCCATTCTTGTTTCGTCGTCCTCTCTTCCCAGTGCAACGTCGTGGAGCCGGCGGACGTCAAGAAGGTGGCCACCACCTTGCAGCGCGCCATCAAGGTGGTCGGCACGCCGGCGTACGAGGAGATGGTGAGGAACTGCATGATCCAGGATCTCTCCTGGAAGGTACGTACGCCCGCCCCGCCAGAGCAGAGCGCCAAGATCGATCGACCGACCGACCACACGTACGCGCCTCGCTCCTGTCGCTGACCGTGGTTTAATTTGCGAAATGCGCAGGGCCCTGCCAAGAACTGGGAGAACGTGCTGCTCAGCCTCGGGGTCGCTGGCGGCGAGCCAGGGGTCGAAGGCGAGGAGATCGCGCCGCTCGCCAAGGAGAACGTGGCCGCGCCCTGAAGAGTTCGGCCTGCAGGGCCCCTGATCTCGCGCGTGGTGCAAAGATGTTGGGACATCTTCTTATATATGCTGTTTCGTTTATGTGATATGGACAAGTATGTGTAGCTGCTTGCTTGTGCTAGTGTAATATAGTGTAGTGGTGGCCAGTGGCACAACCTAATAAGCGCATGAACTAATTGCTTGCGTGTGTAGTTAAGTACCGATCGGTAATTTTATAT |

**Table S1 Continued**

| **Accession** | **AC(%)** | **DNA Sequence** |
| --- | --- | --- |
| SWL370 | 99.57 | CCACAACTGTTCGCGTCCTGCTGGTTCATTATCTGACCTGATTGCATTATTGCAGCTACGAGAAGCCCGTGGAAGGCCGGAAGATCAACTGGATGAAGGCCGGGATCCTCGAGGCCGACAGGGTCCTCACCGTCAGCCCCTACTACGCCGAGGAGCTCATCTCCGGCATCGCCAGGGGCTGCGAGCTCGACAACATCATGCGCCTCACCGGCATCACCGGCATCGTCAACGGCATGGACGTCAGCGAGTGGGACCCCAGCAGGGACAAGTACATCGCCGTGAAGTACGACGTGTCGACGGTGAGCTGGCTAGCTAGCTGATTCTGCTGCCTGGTCCTCCTGCTCATGCTGGTTCGGTTCTGACGCGGCAAGTGTACGTACGTGCGTGCGACGGTGGTGTGGTGTCCGGTTCAGGCCGTGGAGGCCAAGGCGCTGAACAAGGAGGCGCTGCAGGCGGAGGTCGGGCTCCCGGTGGACCGGAACATCCCGCTGGTGGCGTTCATCGGCAGGCTGGAAGAGCAGAAGGGACCCGACGTCATGGCGGCCGCCATCCCGCAGCTCATGGAGATGGTGGAGGACGTGCAGATCGTTCTGCTGGTACGTGTGCGCCGCCCGCCACCCGGCTACTACATGCGTGTATCGTTCGTTCTACTGGAACATGCGTGTGAGCAACGCGATGGATAATGCTGCAGGGCACGGGCAAGAAGAAGTTCGAGCGCATGCTCATGAGCGCCGAGGAGAAGTTCCCAGGCAAGGTGCGCGCCGTGGTCAAGTTCAACGCGGCGCTGGCGCACCACATCATGGCCGGCGCCGACGTGCTCGCCGTCACCAGCCGCTTCGAGCCCTGCGGCCTCATCCAGCTGCAGGGGATGCGATACGGAACGGTACGAGAGAGAAAAAAAAACATCCTGAATCCTGACGAGAGGGACAGAGACAGATTGATTATGAATGCTTCATCGATTTGAATTGATTGATCGATGTCTCCCGCTGCGACTCTTGCAGCCCTGCGCCTGCGCGTCCACCGGTGGACTCGTCGACACCATCATCGAAGGCAAGACCGGGTTCCACATGGGCCGCCTCAGCGTCGACGTAAGCCTACCTCTGCCATGTTCTTTCTTCTTTCTTTCTGTATGTATGTATGTATGTACGAATCAGCACCGCCATTCTTGTTTCGTCGTCCTCTCTTCCCAGTGCAACGTCGTGGAGCCGGCGGACGTCAAGAAGGTGGCCACCACCTTGCAGCGCGCCATCAAGGTGGTCGGCACGCCGGCGTACGAGGAGATGGTGAGGAACTGCATGATCCAGGATCTCTCCTGGAAGGTACGTACGCCCGCCCCGCCAGAGCAGAGCGCCAAGATCGATCGACCGACCGACCACACGTACGCGCCTCGCTCCTGTCGCTGACCGTGGTTTAATTTGCGAAATGCGCAGGGCCCTGCCAAGAACTGGGAGAACGTGCTGCTCAGCCTCGGGGTCGCTGGCGGCGAGCCAGGGGTCGAAGGCGAGGAGATCGCGCCGCTCGCCAAGGAGAACGTGGCCGCGCCCTGAAGAGTTCGGCCTGCAGGGCCCCTGATCTCGCGCGTGGTGCAAAGATGTTGGGACATCTTCTTATATATGCTGTTTCGTTTATGTGATATGGACAAGTATGTGTAGCTGCTTGCTTGTGCTAGTGTAATATAGTGTAGTGGTGGCCAGTGGCACAACCTAATAAGCGCATGAACTAATTGCTTGCGTGTGTAGTTAAGTACCGATCGGTAATTTTATAT |
| SWL371 | 99.44 | CCACAACTGTTCGCGTCCTGCTGGTTCATTATCTGACCTGATTGCATTATTGCAGCTACGAGAAGCCCGTGGAAGGCCGGAAGATCAACTGGATGAAGGCCGGGATCCTCGAGGCCGACAGGGTCCTCACCGTCAGCCCCTACTACGCCGAGGAGCTCATCTCCGGCATCGCCAGGGGCTGCGAGCTCGACAACATCATGCGCCTCACCGGCATCACCGGCATCGTCAACGGCATGGACGTCAGCGAGTGGGACCCCAGCAGGGACAAGTACATCGCCGTGAAGTACGACGTGTCGACGGTGAGCTGGCTAGCTAGCTGATTCTGCTGCCTGGTCCTCCTGCTCATGCTGGTTCGGTTCTGACGCGGCAAGTGTACGTACGTGCGTGCGACGGTGGTGTGGTGTCCGGTTCAGGCCGTGGAGGCCAAGGCGCTGAACAAGGAGGCGCTGCAGGCGGAGGTCGGGCTCCCGGTGGACCGGAACATCCCGCTGGTGGCGTTCATCGGCAGGCTGGAAGAGCAGAAGGGACCCGACGTCATGGCGGCCGCCATCCCGCAGCTCATGGAGATGGTGGAGGACGTGCAGATCGTTCTGCTGGTACGTGTGCGCCGCCCGCCACCCGGCTACTACATGCGTGTATCGTTCGTTCTACTGGAACATGCGTGTGAGCAACGCGATGGATAATGCTGCAGGGCACGGGCAAGAAGAAGTTCGAGCGCATGCTCATGAGCGCCGAGGAGAAGTTCCCAGGCAAGGTGCGCGCCGTGGTCAAGTTCAACGCGGCGCTGGCGCACCACATCATGGCCGGCGCCGACGTGCTCGCCGTCACCAGCCGCTTCGAGCCCTGCGGCCTCATCCAGCTGCAGGGGATGCGATACGGAACGGTACGAGAGAGAAAAAAAAACATCCTGAATCCTGACGAGAGGGACAGAGACAGATTGATTATGAATGCTTCATCGATTTGAATTGATTGATCGATGTCTCCCGCTGCGACTCTTGCAGCCCTGCGCCTGCGCGTCCACCGGTGGACTCGTCGACACCATCATCGAAGGCAAGACCGGGTTCCACATGGGCCGCCTCAGCGTCGACGTAAGCCTACCTCTGCCATGTTCTTTCTTCTTTCTTTCTGTATGTATGTATGTATGTACGAATCAGCACCGCCATTCTTGTTTCGTCGTCCTCTCTTCCCAGTGCAACGTCGTGGAGCCGGCGGACGTCAAGAAGGTGGCCACCACCTTGCAGCGCGCCATCAAGGTGGTCGGCACGCCGGCGTACGAGGAGATGGTGAGGAACTGCATGATCCAGGATCTCTCCTGGAAGGTACGTACGCCCGCCCCGCCAGAGCAGAGCGCCAAGATCGATCGACCGACCGACCACACGTACGCGCCTCGCTCCTGTCGCTGACCGTGGTTTAATTTGCGAAATGCGCAGGGCCCTGCCAAGAACTGGGAGAACGTGCTGCTCAGCCTCGGGGTCGCTGGCGGCGAGCCAGGGGTCGAAGGCGAGGAGATCGCGCCGCTCGCCAAGGAGAACGTGGCCGCGCCCTGAAGAGTTCGGCCTGCAGGGCCCCTGATCTCGCGCGTGGTGCAAAGATGTTGGGACATCTTCTTATATATGCTGTTTCGTTTATGTGATATGGACAAGTATGTGTAGCTGCTTGCTTGTGCTAGTGTAATATAGTGTAGTGGTGGCCAGTGGCACAACCTAATAAGCGCATGAACTAATTGCTTGCGTGTGTAGTTAAGTACCGATCGGTAATTTTATAT |

**Table S1 Continued**

| **Accession** | **AC(%)** | **DNA Sequence** |
| --- | --- | --- |
| SWL378 | 98.63 | CCACAACTGTTCGCGTCCTGCTGGTTCATTATCTGACCTGATTGCATTATTGCAGCTACGAGAAGCCCGTGGAAGGCCGGAAGATCAACTGGATGAAGGCCGGGATCCTCGAGGCCGACAGGGTCCTCACCGTCAGCCCCTACTACGCCGAGGAGCTCATCTCCGGCATCGCCAGGGGCTGCGAGCTCGACAACATCATGCGCCTCACCGGCATCACCGGCATCGTCAACGGCATGGACGTCAGCGAGTGGGACCCCAGCAGGGACAAGTACATCGCCGTGAAGTACGACGTGTCGACGGTGAGCTGGCTAGCTAGCTGATTCTGCTGCCTGGTCCTCCTGCTCATGCTGGTTCGGTTCTGACGCGGCAAGTGTACGTACGTGCGTGCGACGGTGGTGTGGTGTCCGGTTCAGGCCGTGGAGGCCAAGGCGCTGAACAAGGAGGCGCTGCAGGCGGAGGTCGGGCTCCCGGTGGACCGGAACATCCCGCTGGTGGCGTTCATCGGCAGGCTGGAAGAGCAGAAGGGACCCGACGTCATGGCGGCCGCCATCCCGCAGCTCATGGAGATGGTGGAGGACGTGCAGATCGTTCTGCTGGTACGTGTGCGCCGCCCGCCACCCGGCTACTACATGCGTGTATCGTTCGTTCTACTGGAACATGCGTGTGAGCAACGCGATGGATAATGCTGCAGGGCACGGGCAAGAAGAAGTTCGAGCGCATGCTCATGAGCGCCGAGGAGAAGTTCCCAGGCAAGGTGCGCGCCGTGGTCAAGTTCAACGCGGCGCTGGCGCACCACATCATGGCCGGCGCCGACGTGCTCGCCGTCACCAGCCGCTTCGAGCCCTGCGGCCTCATCCAGCTGCAGGGGATGCGATACGGAACGGTACGAGAGAGAAAAAAAAACATCCTGAATCCTGACGAGAGGGACAGAGACAGATTGATTATGAATGCTTCATCGATTTGAATTGATTGATCGATGTCTCCCGCTGCGACTCTTGCAGCCCTGCGCCTGCGCGTCCACCGGTGGACTCGTCGACACCATCATCGAAGGCAAGACCGGGTTCCACATGGGCCGCCTCAGCGTCGACGTAAGCCTACCTCTGCCATGTTCTTTCTTCTTTCTTTCTGTATGTATGTATGTATGTACGAATCAGCACCGCCATTCTTGTTTCGTCGTCCTCTCTTCCCAGTGCAACGTCGTGGAGCCGGCGGACGTCAAGAAGGTGGCCACCACCTTGCAGCGCGCCATCAAGGTGGTCGGCACGCCGGCGTACGAGGAGATGGTGAGGAACTGCATGATCCAGGATCTCTCCTGGAAGGTACGTACGCCCGCCCCGCCAGAGCAGAGCGCCAAGATCGATCGACCGACCGACCACACGTACGCGCCTCGCTCCTGTCGCTGACCGTGGTTTAATTTGCGAAATGCGCAGGGCCCTGCCAAGAACTGGGAGAACGTGCTGCTCAGCCTCGGGGTCGCTGGCGGCGAGCCAGGGGTCGAAGGCGAGGAGATCGCGCCGCTCGCCAAGGAGAACGTGGCCGCGCCCTGAAGAGTTCGGCCTGCAGGGCCCCTGATCTCGCGCGTGGTGCAAAGATGTTGGGACATCTTCTTATATATGCTGTTTCGTTTATGTGATATGGACAAGTATGTGTAGCTGCTTGCTTGTGCTAGTGTAATATAGTGTAGTGGTGGCCAGTGGCACAACCTAATAAGCGCATGAACTAATTGCTTGCGTGTGTAGTTAAGTACCGATCGGTAATTTTATAT |
| SWL385 | 99.07 | CCACAACTGTTCGCGTCCTGCTGGTTCATTATCTGACCTGATTGCATTATTGCAGCTACGAGAAGCCCGTGGAAGGCCGGAAGATCAACTGGATGAAGGCCGGGATCCTCGAGGCCGACAGGGTCCTCACCGTCAGCCCCTACTACGCCGAGGAGCTCATCTCCGGCATCGCCAGGGGCTGCGAGCTCGACAACATCATGCGCCTCACCGGCATCACCGGCATCGTCAACGGCATGGACGTCAGCGAGTGGGACCCCAGCAGGGACAAGTACATCGCCGTGAAGTACGACGTGTCGACGGTGAGCTGGCTAGCTAGCTGATTCTGCTGCCTGGTCCTCCTGCTCATGCTGGTTCGGTTCTGACGCGGCAAGTGTACGTACGTGCGTGCGACGGTGGTGTGGTGTCCGGTTCAGGCCGTGGAGGCCAAGGCGCTGAACAAGGAGGCGCTGCAGGCGGAGGTCGGGCTCCCGGTGGACCGGAACATCCCGCTGGTGGCGTTCATCGGCAGGCTGGAAGAGCAGAAGGGACCCGACGTCATGGCGGCCGCCATCCCGCAGCTCATGGAGATGGTGGAGGACGTGCAGATCGTTCTGCTGGTACGTGTGCGCCGCCCGCCACCCGGCTACTACATGCGTGTATCGTTCGTTCTACTGGAACATGCGTGTGAGCAACGCGATGGATAATGCTGCAGGGCACGGGCAAGAAGAAGTTCGAGCGCATGCTCATGAGCGCCGAGGAGAAGTTCCCAGGCAAGGTGCGCGCCGTGGTCAAGTTCAACGCGGCGCTGGCGCACCACATCATGGCCGGCGCCGACGTGCTCGCCGTCACCAGCCGCTTCGAGCCCTGCGGCCTCATCCAGCTGCAGGGGATGCGATACGGAACGGTACGAGAGAGAAAAAAAAACATCCTGAATCCTGACGAGAGGGACAGAGACAGATTGATTATGAATGCTTCATCGATTTGAATTGATTGATCGATGTCTCCCGCTGCGACTCTTGCAGCCCTGCGCCTGCGCGTCCACCGGTGGACTCGTCGACACCATCATCGAAGGCAAGACCGGGTTCCACATGGGCCGCCTCAGCGTCGACGTAAGCCTACCTCTGCCATGTTCTTTCTTCTTTCTTTCTGTATGTATGTATGTATGTACGAATCAGCACCGCCATTCTTGTTTCGTCGTCCTCTCTTCCCAGTGCAACGTCGTGGAGCCGGCGGACGTCAAGAAGGTGGCCACCACCTTGCAGCGCGCCATCAAGGTGGTCGGCACGCCGGCGTACGAGGAGATGGTGAGGAACTGCATGATCCAGGATCTCTCCTGGAAGGTACGTACGCCCGCCCCGCCAGAGCAGAGCGCCAAGATCGATCGACCGACCGACCACACGTACGCGCCTCGCTCCTGTCGCTGACCGTGGTTTAATTTGCGAAATGCGCAGGGCCCTGCCAAGAACTGGGAGAACGTGCTGCTCAGCCTCGGGGTCGCTGGCGGCGAGCCAGGGGTCGAAGGCGAGGAGATCGCGCCGCTCGCCAAGGAGAACGTGGCCGCGCCCTGAAGAGTTCGGCCTGCAGGGCCCCTGATCTCGCGCGTGGTGCAAAGATGTTGGGACATCTTCTTATATATGCTGTTTCGTTTATGTGATATGGACAAGTATGTGTAGCTGCTTGCTTGTGCTAGTGTAATATAGTGTAGTGGTGGCCAGTGGCACAACCTAATAAGCGCATGAACTAATTGCTTGCGTGTGTAGTTAAGTACCGATCGGTAATTTTATAT |

**Table S1 Continued**

| **Accession** | **AC(%)** | **DNA Sequence** |
| --- | --- | --- |
| SWL386 | 97.96 | CCACAACTGTTCGCGTCCTGCTGGTTCATTATCTGACCTGATTGCATTATTGCAGCTACGAGAAGCCCGTGGAAGGCCGGAAGATCAACTGGATGAAGGCCGGGATCCTCGAGGCCGACAGGGTCCTCACCGTCAGCCCCTACTACGCCGAGGAGCTCATCTCCGGCATCGCCAGGGGCTGCGAGCTCGACAACATCATGCGCCTCACCGGCATCACCGGCATCGTCAACGGCATGGACGTCAGCGAGTGGGACCCCAGCAGGGACAAGTACATCGCCGTGAAGTACGACGTGTCGACGGTGAGCTGGCTAGCTAGCTGATTCTGCTGCCTGGTCCTCCTGCTCATGCTGGTTCGGTTCTGACGCGGCAAGTGTACGTACGTGCGTGCGACGGTGGTGTGGTGTCCGGTTCAGGCCGTGGAGGCCAAGGCGCTGAACAAGGAGGCGCTGCAGGCGGAGGTCGGGCTCCCGGTGGACCGGAACATCCCGCTGGTGGCGTTCATCGGCAGGCTGGAAGAGCAGAAGGGACCCGACGTCATGGCGGCCGCCATCCCGCAGCTCATGGAGATGGTGGAGGACGTGCAGATCGTTCTGCTGGTACGTGTGCGCCGCCCGCCACCCGGCTACTACATGCGTGTATCGTTCGTTCTACTGGAACATGCGTGTGAGCAACGCGATGGATAATGCTGCAGGGCACGGGCAAGAAGAAGTTCGAGCGCATGCTCATGAGCGCCGAGGAGAAGTTCCCAGGCAAGGTGCGCGCCGTGGTCAAGTTCAACGCGGCGCTGGCGCACCACATCATGGCCGGCGCCGACGTGCTCGCCGTCACCAGCCGCTTCGAGCCCTGCGGCCTCATCCAGCTGCAGGGGATGCGATACGGAACGGTACGAGAGAGAAAAAAAAACATCCTGAATCCTGACGAGAGGGACAGAGACAGATTGATTATGAATGCTTCATCGATTTGAATTGATTGATCGATGTCTCCCGCTGCGACTCTTGCAGCCCTGCGCCTGCGCGTCCACCGGTGGACTCGTCGACACCATCATCGAAGGCAAGACCGGGTTCCACATGGGCCGCCTCAGCGTCGACGTAAGCCTACCTCTGCCATGTTCTTTCTTCTTTCTTTCTGTATGTATGTATGTATGTACGAATCAGCACCGCCATTCTTGTTTCGTCGTCCTCTCTTCCCAGTGCAACGTCGTGGAGCCGGCGGACGTCAAGAAGGTGGCCACCACCTTGCAGCGCGCCATCAAGGTGGTCGGCACGCCGGCGTACGAGGAGATGGTGAGGAACTGCATGATCCAGGATCTCTCCTGGAAGGTACGTACGCCCGCCCCGCCAGAGCAGAGCGCCAAGATCGATCGACCGACCGACCACACGTACGCGCCTCGCTCCTGTCGCTGACCGTGGTTTAATTTGCGAAATGCGCAGGGCCCTGCCAAGAACTGGGAGAACGTGCTGCTCAGCCTCGGGGTCGCTGGCGGCGAGCCAGGGGTCGAAGGCGAGGAGATCGCGCCGCTCGCCAAGGAGAACGTGGCCGCGCCCTGAAGAGTTCGGCCTGCAGGGCCCCTGATCTCGCGCGTGGTGCAAAGATGTTGGGACATCTTCTTATATATGCTGTTTCGTTTATGTGATATGGACAAGTATGTGTAGCTGCTTGCTTGTGCTAGTGTAATATAGTGTAGTGGTGGCCAGTGGCACAACCTAATAAGCGCATGAACTAATTGCTTGCGTGTGTAGTTAAGTACCGATCGGTAATTTTATAT |
| SMV002 | 77.72 | CCACAACTGTTCGCGTCCTGCTGGTTCATTATCTGACCTTGATTGCATTGCAGCTACGAGAAGCCCGTGGAAGGCCGGAAGATCAACTGGATGAAGGCCGGGATCCTCGAGGCCGACAGGGTCCTCACCGTCAGCCCCTACTACGCCGAGGAGCTCATCTCCGGCATCGCCAGGGGCTGCGAGCTCGACAACATCATGCGCCTCACCGGCATCACCGGCATCGTCAACGGCATGGACGTCAGCGAGTGGGACCCCAGCAGGGACAAGTACATCGCCGTGAAGTACGACGTGTCGACGGTGAGCTGGCTAGCTAGCTGATTCTGCTGCCTGGTCCTCCTGCTCATGCTGGTTCGGTTCTGACGCGGCAAGTGTACGTACGTGCGTGCGACGGTGGTGTGGTGTCCGGTTCAGGCCGTGGAGGCCAAGGCGCTGAACAAGGAGGCGCTGCAGGCGGAGGTCGGGCTCCCGGTGGACCGGAACATCCCGCTGGTGGCGTTCATCGGCAGGCTGGAAGAGCAGAAGGGCCCCGACGTCATGGCGGCCGCCATCCCGCAGCTCATGGAGATGGTGGAGGACGTGCAGATCGTTCTGCTGGTACGTGTGCGCCGGCCGCCACCCGGCTACTACATGCGTGTATCGTTCTACTGGAACATACGTGTGAGCAACGCGATGGATAATGCTGCAGGGCACGGGCAAGAAGAAGTTCGAGCGCATGCTCATGAGCGCCGAGGAGAAGTTCCCAGGCAAGGTGCGCGCCGTGGTCAAGTTCAACGCGGCGCTGGCGCACCACATCATGGCCGGCGCCGACGTGCTCGCCGTCACCAGCCGCTTCGAGCCCTGCGGCCTCATCCAGCTGCAGGGGATGCGATACGGAACGGTACGAGAGAGAAAAAAAAACATCCTGAATCCTGACGAGAGGGACAGAGACAGATTGATTATGAATGCTTCATCGATTTGAATTGATTGATCGATGTCTCCCGCTGCGACTCTTGCAGCCCTGCGCCTGCGCGTCCACCGGTGGACTCGTCGACACCATCATCGAAGGCAAGACCGGGTTCCACATGGGCCGCCTCAGCGTCGACGTAAGCCTACCTCTGCCATGTTCTTTCTTCTTTCTTTCTGTATGTATGTATGTATGTACGAATCAGCACCGCCATTCTTGTTTCGTCGTCCTCTCTTCCCAGTGCAACGTCGTGGAGCCGGCGGACGTCAAGAAGGTGGCCACCACCTTGCAGCGCGCCATCAAGGTGGTCGGCACGCCGGCGTACGAGGAGATGGTGAGGAACTGCATGATCCAGGATCTCTCCTGGAAGGTACGTACGCCCGCCCCGCCAGAGCAGAGCGCCAAGATCGATCGATCGACCGACCACACGTACGCGCCTCGCTCTTGTCGCTGACCGTGGTTTAATTTGCGAAATGCGCAGGGCCCTGCCAAGAACTGGGAGAACGTGCTGCTCAGCCTCGGGGTCGCCGGCGGCGAGCCAGGGGTTGAAGGCGAGGAGATCGCGCCGCTCGCCAAGGAGAACGTGGCCGCGCCCTGAAGAGTTCGGCCTGCAGGGCCCCTGATCTCGCGCGTGGTGCAAAGATGTTGGGACATCTTCTTATATATGCTGTTTCGTTTATGTGATATGGACAAGTATGTGTAGATGCTTGCTTGTGCTAGTGTAATGTAGTGTAGTGGTGGCCAGTGGCACAACCTAATAAGCGCATGAACTAATTGCTTGCGTGTGTAGTTAAGTACCGATCGGTAATTTTATAT |

**Table S1 Continued**

| **Accession** | **AC(%)** | **DNA Sequence** |
| --- | --- | --- |
| SMV003 | 78.17 | CCACAACTGTTCGCGTCCTGCTGGTTCATTATCTGACCTGATTGCATTATTGCAGCTACGAGAAGCCCGTGGAAGGCCGGAAGATCAACTGGATGAAGGCCGGGATCCTCGAGGCCGACAGGGTCCTCACCGTCAGCCCCTACTACGCCGAGGAGCTCATCTCCGGCATCGCCAGGGGCTGCGAGCTCGACAACATCATGCGCCTCACCGGCATCACCGGCATCGTCAACGGCATGGACGTCAGCGAGTGGGACCCCAGCAGGGACAAGTACATCGCCGTGAAGTACGACGTGTCGACGGTGAGCTGGCTAGCTAGCTGATTCTGCTGCCTGGTCCTCCTGCTCATGCTGGTTCGGTTCTGACGCGGCAAGTGTACGTACGTGCGTGCGACGGTGGTGTGGTGTCCGGTTCAGGCCGTGGAGGCCAAGGCGCTGAACAAGGAGGCGCTGCAGGCGGAGGTCGGGCTCCCGGTGGACCGGAACATCCCGCTGGTGGCGTTCATCGGCAGGCTGGAAGAGCAGAAGGGACCCGACGTCATGGCGGCCGCCATCCCGCAGCTCATGGAGATGGTGGAGGACGTGCAGATCGTTCTGCTGGTACGTGTGCGCCGCCCGCCACCCGGCTACTACATGCGTGTATCGTTCTACTGGAACATACGTGTGAGCAACGCGATGGATAATGCTGCAGGGCACGGGCAAGAAGAAGTTCGAGCGCATGCTCATGAGCGCCGAGGAGAAGTTCCCAGGCAAGGTGCGCGCCGTGGTCAAGTTCAACGCGGCGCTGGCGCACCACATCATGGCCGGCGCCGACGTGCTCGCCGTCACCAGCCGCTTCGAGCCCTGCGGCCTCATCCAGCTGCAGGGGATGCGATACGGAACGGTACGAGAGAGAAAAAAAAACATCCTGAATCCTGACGAGAGGGACAGAGACAGATTGATTATGAATGCTTCATCGATTTGAATTGATTGATCGATGTCTCCCGCTGCGACTCTTGCAGCCCTGCGCCTGCGCGTCCACCGGTGGACTCGTCGACACCATCATCGAAGGCAAGACCGGGTTCCACATGGGCCGCCTCAGCGTCGACGTAAGCCTAGCTCTGCCATGATCTTTCTTCTTTCTGTATGTATGTATGTATGAATCAGCACCGCCATTCTTGTTTCGTCGTCCTCTCTTCCCAGTGCAACGTCGTGGAGCCGGCGGACGTCAAGAAGGTGGCCACCACCTTGCAGCGCGCCATCAAGGTGGTCGGCACGCCGGCGTACGAGGAGATGGTGAGGAACTGCATGATCCAGGATCTCTCCTGGAAGGTACGTACGCCCGCCCCGCCAGAGCAGAGCGCCAAGCCGCCAAGATTGATCGATCGACCGACCACACGTACGCGCCTCGCTCCTGTCGCTGACCGTGGTTTAATTTGCGAAATGCGCAGGGCCCTGCCAAGAACTGGGAGAACGTGCTGCTCAGCCTCGGGGTCGCCGGCGGCGAGCCAGGGGTCGAAGGCGAGGAGATCGCGCCGCTCGCCAAGGAGAACGTGGCCGCGCCCTGAAGAGTTCGGCCTGCAGGGCCCCTGATCTCGCGCGTGGTGCAAAGATGTTGGGACATCTTCTTATATATGCTGTTTCGTTTATGTGATATGGACAAGTATGTGTAGATGCTTGCTTGTGCTAGTGTAATGTAGTGTAGTGGTGGCCAGTGGCACAACCTAATAAGCGCATGAACTAATTGCTTGCGTGTGTAGTTAAGTACCGATCGGTAATTTTATAT |
| SMV004 | 72.27 | CCACAACTGTTCGCGTCCTGCTGGTTCATTATCTGACCTTGATTGCATTGCAGCTACGAGAAGCCCGTGGAAGGCCGGAAGATCAACTGGATGAAGGCCGGGATCCTCGAGGCCGACAGGGTCCTCACCGTCAGCCCCTACTACGCCGAGGAGCTCATCTCCGGCATCGCCAGGGGCTGCGAGCTCGACAACATCATGCGCCTCACCGGCATCACCGGCATCGTCAACGGCATGGACGTCAGCGAGTGGGACCCCAGCAGGGACAAGTACATCGCCGTGAAGTACGACGTGTCGACGGTGAGCTGGCTAGCTAGCTGATTCTGCTGCCTGGTCCTCCTGCTCATGCTGGTTCGGTTCTGACGCGGCGAGTGTACGTACGTGCGTGCGACGGTGGTGTGGTGTCCGGTTCAGGCCGTGGAGGCCAAGGCGCTGAACAAGGAGGCGCTGCAGGCGGAGGTCGGGCTCCCGGTGGACCGGAACATCCCGCTGGTGGCGTTCATCGGCAGGCTGGAAGAGCAGAAGGGACCCGACGTCATGGCGGCCGCCATCCCGCAGCTCATGGAGATGGTGGAGGACGTGCAGATCGTTCTGCTGGTACGTGTGCGCCGCCCGCCACCCGGCTACTACATGCGTGTATCGTTCTACTGGAACATACGTGTGAGCAACGCGATGGATAATGCTGCAGGGCACGGGCAAGAAGAAGTTCGAGCGCATGCTCATGAGCGCCGAGGAGAAGTTCCCAGGCAAGGTGCGCGCCGTGGTCAAGTTCAACGCGGCGCTGGCGCACCACATCATGGCCGGCGCCGACGTGCTCGCCGTCACCAGCCGCTTCGAGCCCTGCGGCCTCATCCAGCTGCAGGGGATGCGATACGGAACGGTACGAGAGAGAAAAAAAAACATCCTGAATCCTGACGAGAGGGACAGAGACAGATTGATTATGAATGCTTCATCGATTTGAATTGATTGATCGATGTCTCCCGCTGCGACTCTTGCAGCCCTGCGCCTGCGCGTCCACCGGTGGACTCGTCGACACCATCATCGAAGGCAAGACCGGGTTCCACATGGGCCGCCTCAGCGTCGACGTAAGCCTAGCTCTGCCATGATCTTTCTTCTTTCTGTATGTATGTATGTATGAATCAGCACCGCCGTTCTTGTTTCGTCGTCCTCTCTTCCCAGTGCAACGTCGTGGAGCCGGCGGACGTCAAGAAGGTGGCCACCACCTTGCAGCGCGCCATCAAGGTGGTCGGCACGCCGGCGTACGAGGAGATGGTGAGGAACTGCATGATCCAGGATCTCTCCTGGAAGGTACGTACGCCCGCCCCGCCAGAGCAGAGCGCCAAGATCGATCGACCGACCGACCACACGTACGCGCCTCGCTCCTGTCGCTGACCGTGGTTTAATTTGCGAAATGCGCAGGGCCCTGCCAAGAACTGGGAGAACGTGCTGCTCAGCCTCGGGGTCGCCGGCGGCGAGCCAGGGGTCGAAGGCGAGGAGATCGCGCCGCTCGCCAAGGAGAACGTGGCCGCGCCCTGAAGAGTTCGGCCTGCAGGCCCCCTGATCTCGCGCGTGGTGCAAACATGTTGGGACATCTTCTTATATATGCTGTTTCGTTTATGTGATATGGACAAGTATGTGTAGCTGCTTGCTTGTGCTAGTGTAATATAATAGTGTAGTGGTGGCCAGTGGCACAACCTAATAAGCGCATGAACTAATTGCTTGCGTGTGTAGTTAAGTACCGATCGGTAATTTTATAT |

**Table S1 Continued**

| **Accession** | **AC(%)** | **DNA Sequence** |
| --- | --- | --- |
| SMV005 | 79.68 | CCACAACTGTTCGCGTCCTGCTGGTTCATTATCTGACCTGATTGCATTATTGCAGCTACGAGAAGCCCGTGGAAGGCCGGAAGATCAACTGGATGAAGGCCGGGATCCTCGAGGCCGACAGGGTCCTCACCGTCAGCCCCTACTACGCCGAGGAGCTCATCTCCGGCATCGCCAGGGGCTGCGAGCTCGACAACATCATGCGCCTCACCGGCATCACCGGCATCGTCAACGGCATGGACGTCAGCGAGTGGGACCCCAGCAGGGACAAGTACATCGCCGTGAAGTACGACGTGTCGACGGTGAGCTGGCTAGCTAGCTGATTCTGCTGCCTGGTCCTCCTGCTCATGCTGGTTCGGTTCTGACGCGGCAAGTGTACGTACGTGCGTGCGACGGTGGTGTGGTGTCCGGTTCAGGCCGTGGAGGCCAAGGCGCTGAACAAGGAGGCGCTGCAGGCGGAGGTCGGGCTCCCGGTGGACCGGAACATCCCGCTGGTGGCGTTCATCGGCAGGCTGGAAGAGCAGAAGGGACCCGACGTCATGGCGGCCGCCATCCCGCAGCTCATGGAGATGGTGGAGGACGTGCAGATCGTTCTGCTGGTACGTGTGCGCCGCCCGCCACCCGGCTACTACATGCGTGTATCGTTCTACTGGAACATACGTGTGAGCAACGCGATGGATAATGCTGCAGGGCACGGGCAAGAAGAAGTTCGAGCGCATGCTCATGAGCGCCGAGGAGAAGTTCCCAGGCAAGGTGCGCGCCGTGGTCAAGTTCAACGCGGCGCTGGCGCACCACATCATGGCCGGCGCCGACGTGCTCGCCGTCACCAGCCGCTTCGAGCCCTGCGGCCTCATCCAGCTGCAGGGGATGCGATACGGAACGGTACGAGAGAGAAAAAAAAACATCCTGAATCCTGACGAGAGGGACAGAGACAGATTGATTATGAATGCTTCATCGATTTGAATTGATTGATCGATGTCTCCCGCTGCGACTCTTGCAGCCCTGCGCCTGCGCGTCCACCGGTGGACTCGTCGACACCATCATCGAAGGCAAGACCGGGTTCCACATGGGCCGCCTCAGCGTCGACGTAAGCCTAGCTCTGCCATGATCTTTCTTCTTTCTGTATGTATGTATGTATGAATCAGCACCGCCATTCTTGTTTCGTCGTCCTCTCTTCCCAGTGCAACGTCGTGGAGCCGGCGGACGTCAAGAAGGTGGCCACCACCTTGCAGCGCGCCATCAAGGTGGTCGGCACGCCGGCGTACGAGGAGATGGTGAGGAACTGCATGATCCAGGATCTCTCCTGGAAGGTACGTACGCCCGCCCCGCCAGAGCAGAGCGCCAAGCCGCCAAGATTGATCGATCGACCGACCACACGTACGCGCCTCGCTCCTGTCGCTGACCGTGGTTTAATTTGCGAAATGCGCAGGGCCCTGCCAAGAACTGGGAGAACGTGCTGCTCAGCCTCGGGGTCGCCGGCGGCGAGCCAGGGGTCGAAGGCGAGGAGATCGCGCCGCTCGCCAAGGAGAACGTGGCCGCGCCCTGAAGAGTTCGGCCTGCAGGGCCCCTGATCTCGCGCGTGGTGCAAAGATGTTGGGACATCTTCTTATATATGCTGTTTCGTTTATGTGATATGGACAAGTATGTGTAGATGCTTGCTTGTGCTAGTGTAATGTAGTGTAGTGGTGGCCAGTGGCACAACCTAATAAGCGCATGAACTAATTGCTTGCGTGTGTAGTTAAGTACCGATCGGTAATTTTATAT |
| SMV006 | 78.23 | CCACAACTGTTCGCGTCCTGCTGGTTCATTATCTGACCTGATTGCATTATTGCAGCTACGAGAAGCCCGTGGAAGGCCGGAAGATCAACTGGATGAAGGCCGGGATCCTCGAGGCCGACAGGGTCCTCACCGTCAGCCCCTACTACGCCGAGGAGCTCATCTCCGGCATCGCCAGGGGCTGCGAGCTCGACAACATCATGCGCCTCACCGGCATCACCGGCATCGTCAACGGCATGGACGTCAGCGAGTGGGACCCCAGCAGGGACAAGTACATCGCCGTGAAGTACGACGTGTCGACGGTGAGCTGGCTAGCTAGCTGATTCTGCTGCCTGGTCCTCCTGCTCATGCTGGTTCGGTTCTGACGCGGCAAGTGTACGTACGTGCGTGCGACGGTGGTGTGGTGTCCGGTTCAGGCCGTGGAGGCCAAGGCGCTGAACAAGGAGGCGCTGCAGGCGGAGGTCGGGCTCCCGGTGGACCGGAACATCCCGCTGGTGGCGTTCATCGGCAGGCTGGAAGAGCAGAAGGGACCCGACGTCATGGCGGCCGCCATCCCGCAGCTCATGGAGATGGTGGAGGACGTGCAGATCGTTCTGCTGGTACGTGTGCGCCGCCCGCCACCCGGCTACTACATGCGTGTATCGTTCTACTGGAACATACGTGTGAGCAACGCGATGGATAATGCTGCAGGGCACGGGCAAGAAGAAGTTCGAGCGCATGCTCATGAGCGCCGAGGAGAAGTTCCCAGGCAAGGTGCGCGCCGTGGTCAAGTTCAACGCGGCGCTGGCGCACCACATCATGGCCGGCGCCGACGTGCTCGCCGTCACCAGCCGCTTCGAGCCCTGCGGCCTCATCCAGCTGCAGGGGATGCGATACGGAACGGTACGAGAGAGAAAAAAAAACATCCTGAATCCTGACGAGAGGGACAGAGACAGATTGATTATGAATGCTTCATCGATTTGAATTGATTGATCGATGTCTCCCGCTGCGACTCTTGCAGCCCTGCGCCTGCGCGTCCACCGGTGGACTCGTCGACACCATCATCGAAGGCAAGACCGGGTTCCACATGGGCCGCCTCAGCGTCGACGTAAGCCTAGCTCTGCCATGATCTTTCTTCTTTCTGTATGTATGTATGTATGAATCAGCACCGCCATTCTTGTTTCGTCGTCCTCTCTTCCCAGTGCAACGTCGTGGAGCCGGCGGACGTCAAGAAGGTGGCCACCACCTTGCAGCGCGCCATCAAGGTGGTCGGCACGCCGGCGTACGAGGAGATGGTGAGGAACTGCATGATCCAGGATCTCTCCTGGAAGGTACGTACGCCCGCCCCGCCAGAGCAGAGCGCCAAGCCGCCAAGATTGATCGATCGACCGACCACACGTACGCGCCTCGCTCCTGTCGCTGACCGTGGTTTAATTTGCGAAATGCGCAGGGCCCTGCCAAGAACTGGGAGAACGTGCTGCTCAGCCTCGGGGTCGCCGGCGGCGAGCCAGGGGTCGAAGGCGAGGAGATCGCGCCGCTCGCCAAGGAGAACGTGGCCGCGCCCTGAAGAGTTCGGCCTGCAGGGCCCCTGATCTCGCGCGTGGTGCAAAGATGTTGGGACATCTTCTTATATATGCTGTTTCGTTTATGTGATATGGACAAGTATGTGTAGATGCTTGCTTGTGCTAGTGTAATGTAGTGTAGTGGTGGCCAGTGGCACAACCTAATAAGCGCATGAACTAATTGCTTGCGTGTGTAGTTAAGTACCGATCGGTAATTTTATAT |

**Table S1 Continued**

| **Accession** | **AC(%)** | **DNA Sequence** |
| --- | --- | --- |
| SMV015 | 71.16 | CCACAACTGTTCGCGTCCTGCTGGTTCATTATCTGACCTGATTGCATTATTGCAGCTACGAGAAGCCCGTGGAAGGCCGGAAGATCAACTGGATGAAGGCCGGGATCCTCGAGGCCGACAGGGTCCTCACCGTCAGCCCCTACTACGCCGAGGAGCTCATCTCCGGCATCGCCAGGGGCTGCGAGCTCGACAACATCATGCGCCTCACCGGCATCACCGGCATCGTCAACGGCATGGACGTCAGCGAGTGGGACCCCAGCAGGGACAAGTACATCGCCGTGAAGTACGACGTGTCGACGGTGAGCTGGCTAGCTAGCTGATTCTGCTGCCTGGTCCTCCTGCTCATCATGCTGGTTCGGTACTGACGCGGCAAGTGTACGTACGTGCGTGCGACGGTGGTGTCCGGTTCAGGCCGTGGAGGCCAAGGCGCTGAACAAGGAGGCGCTGCAGGCGGAGGTCGGGCTCCCGGTGGACCGGAACATCCCGCTGGTGGCGTTCATCGGCAGGCTGGAAGAGCAGAAGGGCCCCGACGTCATGGCGGCCGCCATCCCGCAGCTCATGGAGATGGTGGAGGACGTGCAGATCGTTCTGCTGGTACGTGTGCGCCGCCCGCCACCCGGCTACTACATGCGTGTATCGTTCGTTCTACTGGAACATGCGTGTGAGCAACGCGATGGATAATGCTGCAGGGCACGGGCAAGAAGAAGTTCGAGCGCATGCTCATGAGCGCCGAGGAGAAGTTCCCACGCAAGGTGCGCGCCGTGGTCAAGTTCAACGCGGCGCTGGCGCACCACATCATGGCCGGCGCCGACGTGCTCGCCGTCACCAGCCGCTTCGAGCCCTGCGGCCTCATCCAGCTGCAGGGGATGCGATACGGAACGGTACGAGAGAGAAAAAAAAACATCCTGAATCCTGACGAGAGGGACAGAGACAGATTGATTATGAATGCTTCATCGATTTGAATTGATTGATCGATGTCTCCCGCTGCGACTCTTGCAGCCCTGCGCCTGCGCGTCCACCGGTGGACTCGTCGACACCATCATCGAAGGCAAGACCGGGTTCCACATGGGCCGCCTCAGCGTCGACGTAAGCCTACCTCTGCCATGTTCTTTCTTCTTTCTTTCTGTATGTATGTATGTATGTACGAATCAGCACCGCCATTCTTGTTTCGTCGTCCTCTCTTCCCAGTGCAACGTCGTGGAGCCGGCGGACGTCAAGAAGGTGGCCACCACCTTGCAGCGCGCCATCAAGGTGGTCGGCACGCCGGCGTACGAGGAGATGGTGAGGAACTGCATGATCCAGGATCTCTCCTGGAAGGTACGTACGCCCGCCCCGCCAGAGCAGAGCGCCAAGATCGATCGACCGACCGACCACACGTACGCGCCTCGCTCCTGTCGCTGACCGTGGTTTAATTTGCGAAATGCGCAGGGCCCTGCCAAGAACTGGGAGAACGTGCTGCTCAGCCTCGGGGTCGCTGGCGGCGAGCCAGGGGTCGAAGGCGAGGAGATCGCGCCGCTCGCCAAGGAGAACGTGGCCGCGCCCTGAAGAGTTCGGCCTGCAGGGCCCCTGATCTCGCGCGTGGTGCAAAGATGTTGGGACATCTTCTTATATATGCTGTTTCGTTTATGTGATATGGACAAGTATGTGTAGCTGCTTGCTTGTGCTAGTGTAATATAGTGTAGTGGTGGCCAGTGGCACAACCTAATAAGCGCATGAACTAATTGCTTGCGTGTGTAGTTAAGTACCGATCGGTAATTTTATAT |
| CMV054 | 79.91 | CCACAACTGTTCGCGTCCTGCTGGTTCATTATCTGACCTGGATTGCATTGCAGCTACGAGAAGCCCGTGGAAGGCCGGAAGATCAACTGGATGAAGGCCGGGATCCTCGAGGCCGACAGGGTCCTCACCGTCAGCCCCTACTACGCCGAGGAGCTCATCTCCGGCATCGCCAGGGGCTGCGAGCTCGACAACATCATGCGCCTCACCGGCATCACCGGCATCGTCAACGGCATGGACGTCAGCGAGTGGGACCCCAGCAGGGACAAGTACATCGCCGTGAAGTACGACGTGTCGACGGTGAGCTGGCTAGCTAGCTGATTCTGCTGCCTGGTCCTCCTGCTCATGCTGGTTCGGTTCTGACGCGGCGAGTGTACGTACGTGCGTGCGACGGTGGTGTGGTGTCCGGTTCAGGCCGTGGAGGCCAAGGCGCTGCAGGCGGAGGTCGGGCTCCCGGTGGACCGGAACATCCCGCTGGTGGCGTTCATCGGCAGGCTGGAAGAGCAGAAGGGACCCGACGTCATGGCGGCCGCCATCCCGCAGCTCATGGAGATGGTGGAGGACGTGCAGATCGTTCTGCTGGTACGTGTGCGCCGCCCGCCACCCGGCTACTACATGCGTGTATCGTTCTACTGGAACATACGTGTGAGCAACGCGATGGATAATGCTGCAGGGCACGGGCAAGAAGAAGTTCGAGCGCATGCTCATGAGCGCCGAGGAGAAGTTCCCAGGCAAGGTGCGCGCCGTGGTCAAGTTCAACGCGGCGCTGGCGCACCACATCATGGCCGGCGCCGACGTGCTCGCCGTCACCAGCCGCTTCGAGCCCTGCGGCCTCATCCAGCTGCAGGGGATGCGATACGGAACGGTACGAGAGAGAAAAAAAAACATCCTGAATCCTGACGAGAGGGACAGAGACAGATTGATTATGAATGCTTCATCGATTTGAATTGATTGATCGATGTCTCCCGCTGCGACTCTTGCAGCCCTGCGCCTGCGCGTCCACCGGTGGACTCGTCGACACCATCATCGAAGGCAAGACCGGGTTCCACATGGGCCGCCTCAGCGTCGACGTAAGCCTAGCTCTGCCATGATCTTTCTTCTTTCTGTATGTATGTATGTATGAATCAGCACCGCCGTTCTTGTTTCGTCGTCCTCTCTTCCCAGTGCAACGTCGTGGAGCCGGCGGACGTCAAGAAGGTGGCCACCACCTTGCAGCGCGCCATCAAGGTGGTCGGCACGCCGGCGTACGAGGAGATGGTGAGGAACTGCATGATCCAGGATCTCTCCTGGAAGGTACGTACGCCCGCCCCGCCAGAGCAGAGCGCCAAGATCGATCGACCGACCGACCACACGTACGCGCCTCGCTCCTGTCGCTGACCGTGGTTTAATTTGCGAAATGCGCAGGGCCCTGCCAAGAACTGGGAGAACGTGCTGCTCAGCCTCGGGGTCGCCGGCGGCGAGCCAGGGGTCGAAGGCGAGGAGATCGCGCCGCTCGCCAAGGAGAACGTGGCCGCGCCCTGAAGAGTTCGGCCTGCAGGCCCCCTGATCTCGCGCGTGGTGCAAACATGTTGGGACATCTTCTTATATATGCTGTTTCGTTTATGTGATATGGACAAGTATGTGTAGCTGCTTGCTTGTGCTAGTGTAATATAATAGTGTAGTGGTGGCCAGTGGCACAACCTAATAAGCGCATGAACTAATTGCTTGCGTGTGTAGTTAAGTACCGATCGGTAATTTTATAT |

**Table S1 Continued**

| **Accession** | **AC(%)** | **DNA Sequence** |
| --- | --- | --- |
| CMV065 | 73.00 | CCACAACTGTTCGCGTCCTGCTGGTTCATTATCTGACCTTGATTGCATTGCAGCTACGAGAAGCCCGTGGAAGGCCGGAAGATCAACTGGATGAAGGCCGGGATCCTCGAGGCCGACAGGGTCCTCACCGTCAGCCCCTACTACGCCGAGGAGCTCATCTCCGGCATCGCCAGGGGCTGCGAGCTCGACAACATCATGCGCCTCACCGGCATCACCGGCATCGTCAACGGCATGGACGTCAGCGAGTGGGACCCCAGCAGGGACAAGTACATCGCCGTGAAGTACGACGTGTCGACGGTGAGCTGGCTAGCTAGCTGATTCTGCTGCCTGGTCCTCCTGCTCATGCTGGTTCGGTTCTGACGCGGCGAGTGTACGTACGTGCGTGCGACGGTGGTGTGGTGTCCGGTTCAGGCCGTGGAGGCCAAGGCGCTGAACAAGGAGGCGCTGCAGGCGGAGGTCGGGCTCCCGGTGGACCGGAACATCCCGCTGGTGGCGTTCATCGGCAGGCTGGAAGAGCAGAAGGGCCCCGACGTCATGGCGGCCGCCATCCCGCAGCTCATGGAGATGGTGGAGGACGTGCAGATCGTTCTGCTGGTACGTGTGCGCCGCCCGCCACCCGGCTACTACATGCGTGTATCGTTCGTTCTACTGGAACATGCGTGTGAGCAACGCGATGGATAATGCTGCAGGGCACGGGCAAGAAGAAGTTCGAGCGCATGCTCATGAGCGCCGAGGAGAAGTTCCCAGGCAAGGTGCGCGCCGTGGTCAAGTTCAACGCGGCGCTGGCGCACCACATCATGGCCGGCGCCGACGTGCTCGCCGTCACCAGCCGCTTCGAGCCCTGCGGCCTCATCCAGCTGCAGGGGATGCGATACGGAACGGTACGAGAGAGAAAAAAAAACATCCTGAATCCTGACGAGAGGGACAGAGACAGATTGATTATGAATGCTTCATCGATTTGAATTGATTGATCGATGTCTCCCGCTGCGACTCTTGCAGCCCTGCGCCTGCGCGTCCACCGGTGGACTCGTCGACACCATCATCGAAGGCAAGACCGGGTTCCACATGGGCCGCCTCAGCGTCGACGTAAGCCTACCTCTGCCATGTTCTTTCTTCTTTCTTTCTGTATGTATGTATGTATGTACGAATCAGCACCGCCATTCTTGTTTCGTCGTCCTCTCTTCCCAGTGCAACGTCGTGGAGCCGGCGGACGTCAAGAAGGTGGCCACCACCTTGCAGCGCGCCATCAAGGTGGTCGGCACGCCGGCGTACGAGGAGATGGTGAGGAACTGCATGATCCAGGATCTCTCCTGGAAGGTACGTACGCCCGCCCCGCCAGAGCAGAGCGCCAAGATCGATCGACCGACCGACCACACGTACGCGCCTCGCTCCTGTCGCTGACCGTGGTTTAATTTGCGAAATGCGCAGGGCCCTGCCAAGAACTGGGAGAACGTGCTGCTCAGCCTCGGGGTCGCCGGCGGCGAGCCAGGGGTCGAAGGCGAGGAGATCGCGCCGCTCGCCAAGGAGAACGTGGCCGCGCCCTGAAGAGTTCGGCCTGCAGGGCCCCTGATCTCGCGCGTGGTGCAAAGATGTTGGGACATCTTCTTATATATGCTGTTTCGTTTATGTGATATGGACAAGTATGTGTAGATGCTTGCTTGTGGTAGTGTAATGTAGTGTAGTGGTGGCCAGTGGCACAACCTAATAAGCGCATGAACTAATTGCTTGCGTGTGTAGTTAAGTACCGATCGGTAATTTTATAT |
| CMV075 | 81.46 | CCACAACTGTTCGCGTCCTGCTGGTTCATTATCTGACCTTGATTGCATTGCAGCTACGAGAAGCCCGTGGAAGGCCGGAAGATCAACTGGATGAAGGCCGGGATCCTCGAGGCCGACAGGGTCCTCACCGTCAGCCCCTACTACGCCGAGGAGCTCATCTCCGGCATCGCCAGGGGCTGCGAGCTCGACAACATCATGCGCCTCACCGGCATCACCGGCATCGTCAACGGCATGGACGTCAGCGAGTGGGACCCCAGCAGGGACAAGTACATCGCCGTGAAGTACGACGTGTCGACGGTGAGCTGGCTAGCTAGCTGATTCTGCTGCCTGGTCCTCCTGCTCATGCTGGTTCGGTTCTGACGCGGCGAGTGTACGTACGTGCGTGCGACGGTGGTGTGGTGTCCGGTTCAGGCCGTGGAGGCCAAGGCGCTGAACAAGGAGGCGCTGCAGGCGGAGGTCGGGCTCCCGGTGGACCGGAACATCCCGCTGGTGGCGTTCATCGGCAGGCTGGAAGAGCAGAAGGGACCCGACGTCATGGCGGCCGCCATCCCGCAGCTCATGGAGATGGTGGAGGACGTGCAGATCGTTCTGCTGGTACGTGTGCGCCGCCCGCCACCCGGCTACTACATGCGTGTATCGTTCTACTGGAACATACGTGTGAGCAACGCGATGGATAATGCTGCAGGGCACGGGCAAGAAGAAGTTCGAGCGCATGCTCATGAGCGCCGAGGAGAAGTTCCCAGGCAAGGTGCGCGCCGTGGTCAAGTTCAACGCGGCGCTGGCGCACCACATCATGGCCGGCGCCGACGTGCTCGCCGTCACCAGCCGCTTCGAGCCCTGCGGCCTCATCCAGCTGCAGGGGATGCGATACGGAACGGTACGAGAGAGAAAAAAAAACATCCTGAATCCTGACGAGAGGGACAGAGACAGATTGATTATGAATGCTTCATCGATTTGAATTGATTGATCGATGTCTCCCGCTGCGACTCTTGCAGCCCTGCGCCTGCGCGTCCACCGGTGGACTCGTCGACACCATCATCGAAGGCAAGACCGGGTTCCACATGGGCCGCCTCAGCGTCGACGTAAGCCTAGCTCTGCCATGATCTTTCTTCTTTCTGTATGTATGTATGTATGAATCAGCACCGCCGTTCTTGTTTCGTCGTCCTCTCTTCCCAGTGCAACGTCGTGGAGCCGGCGGACGTCAAGAAGGTGGCCACCACCTTGCAGCGCGCCATCAAGGTGGTCGGCACGCCGGCGTACGAGGAGATGGTGAGGAACTGCATGATCCAGGATCTCTCCTGGAAGGTACGTACGCCCGCCCCGCCAGAGCAGAGCGCCAAGATCGATCGACCGACCGACCACACGTACGCGCCTCGCTCCTGTCGCTGACCGTGGTTTAATTTGCGAAATGCGCAGGGCCCTGCCAAGAACTGGGAGAACGTGCTGCTCAGCCTCGGGGTCGCCGGCGGCGAGCCAGGGGTCGAAGGCGAGGAGATCGCGCCGCTCGCCAAGGAGAACGTGGCCGCGCCCTGAAGAGTTCGGCCTGCAGGCCCCCTGATCTCGCGCGTGGTGCAAACATGTTGGGACATCTTCTTATATATGCTGTTTCGTTTATGTGATATGGACAAGTATGTGTAGCTGCTTGCTTGTGCTAGTGTAATATAATAGTGTAGTGGTGGCCAGTGGCACAACCTAATAAGCGCATGAACTAATTGCTTGCGTGTGTAGTTAAGTACCGATCGGTAATTTTATAT |

**Table S1 Continued**

| **Accession** | **AC(%)** | **DNA Sequence** |
| --- | --- | --- |
| SML090 | 83.22 | CCACAACTGTTCGCGTCCTGCTGGTTCATTATCTGACCTGATTGCATTATTGCAGCTACGAGAAGCCCGTGGAAGGCCGGAAGATCAACTGGATGAAGGCCGGGATCCTCGAGGCCGACAGGGTCCTCACCGTCAGCCCCTACTACGCCGAGGAGCTCATCTCCGGCATCGCCAGGGGCTGCGAGCTCGACAACATCATGCGCCTCACCGGCATCACCGGCATCGTCAACGGCATGGACGTCAGCGAGTGGGACCCCAGCAGGGACAAGTACATCGCCGTGAAGTACGACGTGTCGACGGTGAGCTGGCTAGCTCTGATTCTGCTGCCTGGTCCTCCTGCTCATCATGCTGGTTCGGTACTGACGCGGCAAGTGTACGTACGTGCGTGCGACGGTGGTGTCCGGTTCAGGCCGTGGAGGCCAAGGCGCTGAACAAGGAGGCGCTGCAGGCGGAGGTCGGGCTCCCGGTGGACCGGAACATCCCGCTGGTGGCGTTCATCGGCAGGCTGGAAGAGCAGAAGGGCCCCGACGTCATGGCGGCCGCCATCCCGCAGCTCATGGAGATGGTGGAGGACGTGCAGATCGTTCTGCTGGTACGTGTGCGCCGGCCGCCACCCGGCTACTACATGCGTGTATCGTTCGTTCTACTGGAACATGCGTGTGAGCAACGCGATGGATAATGCTGCAGGGCACGGGCAAGAAGAAGTTCGAGCGCATGCTCATGAGCGCCGAGGAGAAGTTCCCAGGCAAGGTGCGCGCCGTGGTCAAGTTCAACGCGGCGCTGGCGCACCACATCATGGCCGGCGCCGACGTGCTCGCCGTCACCAGCCGCTTCGAGCCCTGCGGCCTCATCCAGCTGCAGGGGATGCGATACGGAACGGTACGAGAGAGAAAAAAAAACATCCTGAATCCTGACGAGAGGGACAGAGACAGATTGATTATGAATGCTTCATCGATTTGAATTGATTGATCGATGTCTCCCGCTGCGACTCTTGCAGCCCTGCGCCTGCGCGTCCACCGGTGGACTCGTCGACACCATCATCGAAGGCAAGACCGGGTTCCACATGGGCCGCCTCAGCGTCGACGTAAGCCTAGCTCTGCCATGTTCTTTCTTCTTTCTTTCTGTATGTATGTATGAATCAGCACCGCCGTTCTTGTTTCGTCGTCGTCCTCTCTTCCCAGTGTAACGTCGTGGAGCCGGCGGACGTCAAGAAGGTGGCCACCACATTGCAGCGCGCCATCAAGGTGGTCGGCACGCCGGCGTACGAGGAGATGGTGAGGAACTGCATGATCCAGGATCTCTCCTGGAAGGTACGTACGCCCGCCCCGCCCCGCCCCGCCAGAGCAGAGCGCCAAGATCGACCGATCGACCGACCACACGTACGCGCCTCGCTCCTGTCGCTGACCGTGGTTTAATTTGCGAAATGCGCAGGGCCCTGCCAAGAACTGGGAGAACGTGCTGCTCAGCCTCGGGGTCGCCGGCGGCGAGCCAGGGGTCGAAGGCGAGGAGATCGCGCCGCTCGCCAAGGAGAACGTGGCCGCGCCCTGAAGAGTTCGGCCTGCAGGGCCCCTGATCTCGCGCGTGGTGCAAAGATGTTGGGACATCTTCTTATATATGCTGTTTCGTTTATGTGATATGGACAAGTATGTGTAGCTGCTTGCTTGTGCTAGTGTAATGTAGTGTAGTGGTGGCCAGTGGCACAACCTAATAAGCGCATGAACTAATTGCTTGCGTGTGTAGTTAAGTACCGATCGGTAATTTTATAT |
| SML136 | 62.62 | CCACAACTGTTCGCGTCCTGCTGGTTCATTATCTGACCTGATTGCATTATTGCAGCTACGAGAAGCCCGTGGAAGGCCGGAAGATCAACTGGATGAAGGCCGGGATCCTCGAGGCCGACAGGGTCCTCACCGTCAGCCCCTACTACGCCGAGGAGCTCATCTCCGGCATCGCCAGGGGCTGCGAGCTCGACAACATCATGCGCCTCACCGGCATCACCGGCATCGTCAACGGCATGGACGTCAGCGAGTGGGACCCCAGCAGGGACAAGTACATCGCCGTGAAGTACGACGTGTCGACGGTGAGCTGGCTAGCTCTGATTCTGCTGCCTGGTCCTCCTGCTCATCATGCTGGTTCGGTACTGACGCGGCAAGTGTACGTACGTGCGTGCGACGGTGGTGTCCGGTTCAGGCCGTGGAGGCCAAGGCGCTGAACAAGGAGGCGCTGCAGGCGGAGGTCGGGCTCCCGGTGGACCGGAACATCCCGCTGGTGGCGTTCATCGGCAGGCTGGAAGAGCAGAAGGGCCCCGACGTCATGGCGGCCGCCATCCCGCAGCTCATGGAGATGGTGGAGGACGTGCAGATCGTTCTGCTGGTACGTGTGCGCCGGCCGCCACCCGGCTACTACATGCGTGTATCGTTCGTTCTACTGGAACATGCGTGTGAGCAACGCGATGGATAATGCTGCAGGGCACGGGCAAGAAGAAGTTCGAGCGCATGCTCATGAGCGCCGAGGAGAAGTTCCCAGGCAAGGTGCGCGCCGTGGTCAAGTTCAACGCGGCGCTGGCGCACCACATCATGGCCGGCGCCGACGTGCTCGCCGTCACCAGCCGCTTCGAGCCCTGCGGCCTCATCCAGCTGCAGGGGATGCGATACGGAACGGTACGAGAGAGAAAAAAAAACATCCTGAATCCTGACGAGAGGGACAGAGACAGATTGATTATGAATGCTTCATCGATTTGAATTGATTGATCGATGTCTCCCGCTGCGACTCTTGCAGCCCTGCGCCTGCGCGTCCACCGGTGGACTCGTCGACACCATCATCGAAGGCAAGACCGGGTTCCACATGGGCCGCCTCAGCGTCGACGTAAGCCTAGCTCTGCCATGTTCTTTCTTCTTTCTTTCTGTATGTATGTATGAATCAGCACCGCCGTTCTTGTTTCGTCGTCTTCCTCTCTTCCCAGTGTAACGTCGTGGAGCCGGCGGACGTCAAGAAGGTGGCCACCACATTGCAGCGCGCCATCAAGGTGGTCGGCACGCCGGCGTACGAGGAGATGGTGAGGAACTGCATGATCCAGGATCTCTCCTGGAAGGTACGTACGCCCGCCCCGCCCCGCCCCGCCAGAGCAGAGCGCCAAGATCGACCGATCGACCGACCACACGTACGCGCCTCGCTCCTGTCGCTGACCGTGGTTTAATTTGCGAAATGCGCAGGGCCCTGCCAAGAACTGGGAGAACGTGCTGCTCAGCCTCGGGGTCGCCGGCGGCGAGCCAGGGGTCGAAGGCGAGGAGATCGCGCCGCTCGCCAAGGAGAACGTGGCCGCGCCCTGAAGAGTTCGGCCTGCAGGGCCCCTGATCTCGCGCGTGGTGCAAAGATGTTGGGACATCTTCTTATATATGCTGTTTCGTTTATGTGATATGGACAAGTATGTGTAGCTGCTTGCTTGTGCTAGTGTAATATAGTGTAGTGGTGGCCAGTGGCACAACCTAATAAGCGCATGAACTAATTGCTTGCGTGTGTAGTTAAGTACCGATCGGTAATTTTATAT |

**Table S1 Continued**

| **Accession** | **AC(%)** | **DNA Sequence** |
| --- | --- | --- |
| SML137 | 75.75 | CCACAACTGTTCGCGTCCTGCTGGTTCATTATCTGACCTGATTGCATTATTGCAGCTACGAGAAGCCCGTGGAAGGCCGGAAGATCAACTGGATGAAGGCCGGGATCCTCGAGGCCGACAGGGTCCTCACCGTCAGCCCCTACTACGCCGAGGAGCTCATCTCCGGCATCGCCAGGGGCTGCGAGCTCGACAACATCATGCGCCTCACCGGCATCACCGGCATCGTCAACGGCATGGACGTCAGCGAGTGGGACCCCAGCAGGGACAAGTACATCGCCGTGAAGTACGACGTGTCGACGGTGAGCTGGCTAGCTAGCTGATTCTGCTGCCTGGTCCTCCTGCTCATGCTGGTTCGGTTCTGACGCGGCAAGTGTACGTACGTGCGTGCGACGGTGGTGTGGTGTCCGGTTCAGGCCGTGGAGGCCAAGGCGCTGAACAAGGAGGCGCTGCAGGCGGAGGTCGGGCTCCCGGTGGACCGGAACATCCCGCTGGTGGCGTTCATCGGCAGGCTGGAAGAGCAGAAGGGACCCGACGTCATGGCGGCCGCCATCCCGCAGCTCATGGAGATGGTGGAGGACGTGCAGATCGTTCTGCTGGTACGTGTGCGCCGCCCGCCACCCGGCTACTACATGCGTGTATCGTTCGTTCTACTGGAACATGCGTGTGAGCAACGCGATGGATAATGCTGCAGGGCACGGGCAAGAAGAAGTTCGAGCGCATGCTCATGAGCGCCGAGGAGAAGTTCCCAGGCAAGGTGCGCGCCGTGGTCAAGTTCAACGCGGCGCTGGCGCACCACATCATGGCCGGCGCCGACGTGCTCGCCGTCACCAGCCGCTTCGAGCCCTGCGGCCTCATCCAGCTGCAGGGGATGCGATACGGAACGGTACGAGAGAGAAAAAAAAACATCCTGAATCCTGACGAGAGGGACAGAGACAGATTGATTATGAATGCTTCATCGATTTGAATTGATTGATCGATGTCTCCCGCTGCGACTCTTGCAGCCCTGCGCCTGCGCGTCCACCGGTGGACTCGTCGACACCATCATCGAAGGCAAGACCGGGTTCCACATGGGCCGCCTCAGCGTCGACGTAAGCCTACCTCTGCCATGTTCTTTCTTCTTTCTTTCTGTATGTATGTATGTATGTACGAATCAGCACCGCCATTCTTGTTTCGTCGTCCTCTCTTCCCAGTGCAACGTCGTGGAGCCGGCGGACGTCAAGAAGGTGGCCACCACCTTGCAGCGCGCCATCAAGGTGGTCGGCACGCCGGCGTACGAGGAGATGGTGAGGAACTGCATGATCCAGGATCTCTCCTGGAAGGTACGTACGCCCGCCCCGCCAGAGCAGAGCGCCAAGATCGATCGACCGACCGACCACACGTACGCGCCTCGCTCCTGTCGCTGACCGTGGTTTAATTTGCGAAATGCGCAGGGCCCTGCCAAGAACTGGGAGAACGTGCTGCTCAGCCTCGGGGTCGCTGGCGGCGAGCCAGGGGTCGAAGGCGAGGAGATCGCGCCGCTCGCCAAGGAGAACGTGGCCGCGCCCTGAAGAGTTCGGCCTGCAGGGCCCCTGATCTCGCGCGTGGTGCAAAGATGTTGGGACATCTTCTTATATATGCTGTTTCGTTTATGTGATATGGACAAGTATGTGTAGCTGCTTGCTTGTGCTAGTGTAATATAGTGTAGTGGTGGCCAGTGGCACAACCTAATAAGCGCATGAACTAATTGCTTGCGTGTGTAGTTAAGTACCGATCGGTAATTTTATAT |
| SML173 | 77.62 | CCACAACTGTTCGCGTCCTGCTGGTTCATTATCTGACCTTGATTGCATTGCAGCTACGAGAAGCCCGTGGAAGGCCGGAAGATCAACTGGATGAAGGCCGGGATCCTCGAGGCCGACAGGGTCCTCACCGTCAGCCCCTACTACGCCGAGGAGCTCATCTCCGGCATCGCCAGGGGCTGCGAGCTCGACAACATCATGCGCCTCACCGGCATCACCGGCATCGTCAACGGCATGGACGTCAGCGAGTGGGACCCCAGCAGGGACAAGTACATCGCCGTGAAGTACGACGTGTCGACGGTGAGCTGGCTAGCTAGCTGATTCTGCTGCCTGGTCCTCCTGCTCATGCTGGTTCGGTTCTGACGCGGCAAGTGTACGTACGTGCGTGCGACGGTGGTGTGGTGTCCGGTTCAGGCCGTGGAGGCCAAGGCGCTGAACAAGGAGGCGCTGCAGGCGGAGGTCGGGCTCCCGGTGGACCGGAACATCCCGCTGGTGGCGTTCATCGGCAGGCTGGAAGAGCAGAAGGGCCCCGACGTCATGGCGGCCGCCATCCCGCAGCTCATGGAGATGGTGGAGGACGTGCAGATCGTTCTGCTGGTACGTGTGCGCCGGCCGCCACCCGGCTACTACATGCGTGTATCGTTCTACTGGAACATACGTGTGAGCAACGCGATGGATAATGCTGCAGGGCACGGGCAAGAAGAAGTTCGAGCGCATGCTCATGAGCGCCGAGGAGAAGTTCCCAGGCAAGGTGCGCGCCGTGGTCAAGTTCAACGCGGCGCTGGCGCACCACATCATGGCCGGCGCCGACGTGCTCGCCGTCACCAGCCGCTTCGAGCCCTGCGGCCTCATCCAGCTGCAGGGGATGCGATACGGAACGGTACGAGAGAGAAAAAAAAACATCCTGAATCCTGACGAGAGGGACAGAGACAGATTGATTATGAATGCTTCATCGATTTGAATTGATTGATCGATGTCTCCCGCTGCGACTCTTGCAGCCCTGCGCCTGCGCGTCCACCGGTGGACTCGTCGACACCATCATCGAAGGCAAGACCGGGTTCCACATGGGCCGCCTCAGCGTCGACGTAAGCCTACCTCTGCCATGTTCTTTCTTCTTTCTTTCTGTATGTATGTATGTATGTACGAATCAGCACCGCCATTCTTGTTTCGTCGTCCTCTCTTCCCAGTGCAACGTCGTGGAGCCGGCGGACGTCAAGAAGGTGGCCACCACCTTGCAGCGCGCCATCAAGGTGGTCGGCACGCCGGCGTACGAGGAGATGGTGAGGAACTGCATGATCCAGGATCTCTCCTGGAAGGTACGTACGCCCGCCCCGCCAGAGCAGAGCGCCAAGATCGATCGATCGACCGACCACACGTACGCGCCTCGCTCTTGTCGCTGACCGTGGTTTAATTTGCGAAATGCGCAGGGCCCTGCCAAGAACTGGGAGAACGTGCTGCTCAGCCTCGGGGTCGCCGGCGGCGAGCCAGGGGTTGAAGGCGAGGAGATCGCGCCGCTCGCCAAGGAGAACGTGGCCGCGCCCTGAAGAGTTCGGCCTGCAGGGCCCCTGATCTCGCGCGTGGTGCAAAGATGTTGGGACATCTTCTTATATATGCTGTTTCGTTTATGTGATATGGACAAGTATGTGTAGATGCTTGCTTGTGCTAGTGTAATGTAGTGTAGTGGTGGCCAGTGGCACAACCTAATAAGCGCATGAACTAATTGCTTGCGTGTGTAGTTAAGTACCGATCGGTAATTTTATAT |

**Table S1 Continued**

| **Accession** | **AC(%)** | **DNA Sequence** |
| --- | --- | --- |
| SML280 | 83.44 | CCACAACTGTTCGCGTCCTGCTGGTTCATTATCTGACCTGATTGCATTATTGCAGCTACGAGAAGCCCGTGGAAGGCCGGAAGATCAACTGGATGAAGGCCGGGATCCTCGAGGCCGACAGGGTCCTCACCGTCAGCCCCTACTACGCCGAGGAGCTCATCTCCGGCATCGCCAGGGGCTGCGAGCTCGACAACATCATGCGCCTCACCGGCATCACCGGCATCGTCAACGGCATGGACGTCAGCGAGTGGGACCCCAGCAGGGACAAGTACATCGCCGTGAAGTACGACGTGTCGACGGTGAGCTGGCTAGCTAGCTGATTCTGCTGCCTGGTCCTCCTGCTCATGCTGGTTCGGTTCTGACGCGGCAAGTGTACGTACGTGCGTGCGACGGTGGTGTGGTGTCCGGTTCAGGCCGTGGAGGCCAAGGCGCTGAACAAGGAGGCGCTGCAGGCGGAGGTCGGGCTCCCGGTGGACCGGAACATCCCGCTGGTGGCGTTCATCGGCAGGCTGGAAGAGCAGAAGGGACCCGACGTCATGGCGGCCGCCATCCCGCAGCTCATGGAGATGGTGGAGGACGTGCAGATCGTTCTGCTGGTACGTGTGCGCCGCCCGCCACCCGGCTACTACATGCGTGTATCGTTCTACTGGAACATACGTGTGAGCAACGCGATGGATAATGCTGCAGGGCACGGGCAAGAAGAAGTTCGAGCGCATGCTCATGAGCGCCGAGGAGAAGTTCCCAGGCAAGGTGCGCGCCGTGGTCAAGTTCAACGCGGCGCTGGCGCACCACATCATGGCCGGCGCCGACGTGCTCGCCGTCACCAGCCGCTTCGAGCCCTGCGGCCTCATCCAGCTGCAGGGGATGCGATACGGAACGGTACGAGAGAGAAAAAAAAACATCCTGAATCCTGACGAGAGGGACAGAGACAGATTGATTATGAATGCTTCATCGATTTGAATTGATTGATCTATGTCTCCCGCTGCGACTCTTGCAGCCCTGCGCCTGCGCGTCCACCGGTGGACTCGTCGACACCATCATCGAAGGCAAGACCGGGTTCCACATGGGCCGCCTCAGCGTCGACGTAAGCCTAGCTCTGCCATGATCTTTCTTCTTTCTGTATGTATGTATGTATGAATCAGCACCGCCATTCTTGTTTCGTCGTCCTCTCTTCCCAGTGCAACGTCGTGGAGCCGGCGGACGTCAAGAAGGTGGCCACCACCTTGCAGCGCGCCATCAAGGTGGTCGGCACGCCGGCGTACGAGGAGATGGTGAGGAACTGCATGATCCAGGATCTCTCCTGGAAGGTACGTACGCCCGCCCCGCCAGAGCAGAGCGCCAAGCCGCCAAGATTGATCGATCGACCGACCACACGTACGCGCCTCGCTCCTGTCGCTGACCGTGGTTTAATTTGCGAAATGCGCAGGGCCCTGCCAAGAACTGGGAGAACGTGCTGCTCAGCCTCGGGGTCGCCGGCGGCGAGCCAGGGGTCGAAGGCGAGGAGATCGCGCCGCTCGCCAAGGAGAACGTGGCCGCGCCCTGAAGAGTTCGGCCTGCAGGGCCCCTGATCTCGCGCGTGGTGCAAAGATGTTGGGACATCTTCTTATATATGCTGTTTCGTTTATGTGATATGGACAAGTATGTGTAGATGCTTGCTTGTGCTAGTGTAATGTAGTGTAGTGGTGGCCAGTGGCACAACCTAATAAGCGCATGAACTAATTGCTTGCGTGTGTAGTTAAGTACCGATCGGTAATTTTATAT |
| SML306 | 82.80 | CCACAACTGTTCGCGTCCTGCTGGTTCATTATCTGACCTGATTGCATTATTGCAGCTACGAGAAGCCCGTGGAAGGCCGGAAGATCAACTGGATGAAGGCCGGGATCCTCGAGGCCGACAGGGTCCTCACCGTCAGCCCCTACTACGCCGAGGAGCTCATCTCCGGCATCGCCAGGGGCTGCGAGCTCGACAACATCATGCGCCTCACCGGCATCACCGGCATCGTCAACGGCATGGACGTCAGCGAGTGGGACCCCAGCAGGGACAAGTACATCGCCGTGAAGTACGACGTGTCGACGGTGAGCTGGCTAGCTAGCTGATTCTGCTGCCTGGTCCTCCTGCTCATGCTGGTTCGGTTCTGACGCGGCAAGTGTACGTACGTGCGTGCGACGGTGGTGTGGTGTCCGGTTCAGGCCGTGGAGGCCAAGGCGCTGAACAAGGAGGCGCTGCAGGCGGAGGTCGGGCTCCCGGTGGACCGGAACATCCCGCTGGTGGCGTTCATCGGCAGGCTGGAAGAGCAGAAGGGACCCGACGTCATGGCGGCCGCCATCCCGCAGCTCATGGAGATGGTGGAGGACGTGCAGATCGTTCTGCTGGTACGTGTGCGCCGCCCGCCACCCGGCTACTACATGCGTGTATCGTTCTACTGGAACATACGTGTGAGCAACGCGATGGATAATGCTGCAGGGCACGGGCAAGAAGAAGTTCGAGCGCATGCTCATGAGCGCCGAGGAGAAGTTCCCAGGCAAGGTGCGCGCCGTGGTCAAGTTCAACGCGGCGCTGGCGCACCACATCATGGCCGGCGCCGACGTGCTCGCCGTCACCAGCCGCTTCGAGCCCTGCGGCCTCATCCAGCTGCAGGGGATGCGATACGGAACGGTACGAGAGAGAAAAAAAAACATCCTGAATCCTGACGAGAGGGACAGAGACAGATTGATTATGAATGCTTCATCGATTTGAATTGATTGATCTATGTCTCCCGCTGCGACTCTTGCAGCCCTGCGCCTGCGCGTCCACCGGTGGACTCGTCGACACCATCATCGAAGGCAAGACCGGGTTCCACATGGGCCGCCTCAGCGTCGACGTAAGCCTAGCTCTGCCATGATCTTTCTTCTTTCTGTATGTATGTATGTATGAATCAGCACCGCCATTCTTGTTTCGTCGTCCTCTCTTCCCAGTGCAACGTCGTGGAGCCGGCGGACGTCAAGAAGGTGGCCACCACCTTGCAGCGCGCCATCAAGGTGGTCGGCACGCCGGCGTACGAGGAGATGGTGAGGAACTGCATGATCCAGGATCTCTCCTGGAAGGTACGTACGCCCGCCCCGCCAGAGCAGAGCGCCAAGCCGCCAAGATTGATCGATCGACCGACCACACGTACGCGCCTCGCTCCTGTCGCTGACCGTGGTTTAATTTGCGAAATGCGCAGGGCCCTGCCAAGAACTGGGAGAACGTGCTGCTCAGCCTCGGGGTCGCCGGCGGCGAGCCAGGGGTCGAAGGCGAGGAGATCGCGCCGCTCGCCAAGGAGAACGTGGCCGCGCCCTGAAGAGTTCGGCCTGCAGGGCCCCTGATCTCGCGCGTGGTGCAAAGATGTTGGGACATCTTCTTATATATGCTGTTTCGTTTATGTGATATGGACAAGTATGTGTAGCTGCTTGCTTGTGCTAGTGTAATAAGTGTAGTGGTGGCCAGTGGCACAACCTAATAAGCGCATGAACTAATTGCTTGCGTGTGTAGTTAAGTACCGATCGGTAATTTTATAT |

**Table S1 Continued**

| **Accession** | **AC(%)** | **DNA Sequence** |
| --- | --- | --- |
| SML383 | 81.10 | CCACAACTGCTCGCGTCCTGCTGGTTCATTATCTGACCTTGATTGCATTGCAGCTACGAGAAGCCCGTGGAAGGCCGGAAGATCAACTGGATGAAGGCCGGGATCCTCGAGGCCGACAGGGTCCTCACCGTGAGCCCCTACTACGCCGAGGAGCTCATCTCCGGCATCGCCAGGGGCTGCGAGCTCGACAACATCATGCGCCTCACCGGCATCACCGGCATCGTCAACGGCATGGACGTCAGCGAGTGGGACCCCAGCAGGGACAAGTACATCGCCGTGAAGTACGACGTGTCGACGGTGAGCTGGCTAGCTGATTCTGCTGCCTGGTCCTCCTGCTCATGCTGGTTCGGTTCTGACGCGGCAAGTGTACGTACGTGCGTGCGACGGCGGTGTGGTGTCCGGTTCAGGCCGTGGAGGCCAAGGCGCTGAACAAGGAGGCGCTGCAGGCGGAGGTCGGGCTCCCGGTGGACCGGAACATCCCGCTGGTGGCGTTCATCGGCAGGCTGGAAGAGCAGAAGGGACCCGACGTCATGGCGGCCGCCATCCCGCAGCTCATGGAGATGGTGGAGGACGTGCAGATCGTTCTGCTGGTACGTGTGCGCCGCCCGCCACCCGGCTACTACATGCGTGTATCGTTCTACTGGAACATACGTGTGAGCAACGCGATGGATAATGCTGCAGGGCACGGGCAAGAAGAAGTTCGAGCGCATGCTCATGAGCGCCGAGGAGAAGTTCCCAGGCAAGGTGCGCGCCGTGGTCAAGTTCAACGCGGCGCTGGCGCACCACATCATGGCCGGCGCCGACGTGCTCGCCGTCACCAGCCGCTTCGAGCCCTGCGGCCTCATCCAGCTGCAGGGGATGCGATACGGAACGGTACGAGAGAGAAAAAAAAACATCCTGAATCCTGACGAGAGGGACAGAGACAGATTGATTATGAATGCTTCATCGATTTGAATTGATTGATCGATGTCTCCCGCTGCGACTCTTGCAGCCCTGCGCCTGCGCGTCCACCGGTGGACTCGTCGACACCATCATCGAAGGCAAGACCGGGTTCCACATGGGCCGCCTCAGCGTCGACGTAAGCCTACCTCTGCCATGTTCTTTCTTCTTTCTTTCTGTATGTATGTATGTATGTACGAATCAGCACCGCCATTCTTGTTTCGTCGTCCTCTCTTCCCAGTGCAACGTCGTGGAGCCGGCGGACGTCAAGAAGGTGGCCACCACCTTGCAGCGCGCCATCAAGGTGGTCGGCACGCCGGCGTACGAGGAGATGGTGAGGAACTGCATGATCCAGGATCTCTCCTGGAAGGTACGTACGCCCGCCCCGCCAGAGCAGAGCGCCAAGATCGATCGACCGACCGACCACACGTACGCGCCTCGCTCCTGTCGCTGACCGTGGTTTAATTTGCGAAATGCGCAGGGCCCTGCCAAGAACTGGGAGAACGTGCTGCTCAGCCTCGGGGTCGCTGGCGGCGAGCCAGGGGTCGAAGGCGAGGAGATCGCGCCGCTCGCCAAGGAGAACGTGGCCGCGCCCTGAAGAGTTCGGCCTGCAGGGCCCCTGATCTCGCGCGTGGTGCAAAGATGTTGGGACATCTTCTTATATATGCTGTTTCGTTTATGTGATATGGACAAGTATGTGTAGCTGCTTGCTTGTGCTAGTGTAATATAGTGTAGTGGTGGCCAGTGGCACAACCTAATAAGCGCATGAACTAATTGCTTGCGTGTGTAGTTAAGTACCGATCGGTAATTTTATAT |
| SHL391 | 84.52 | CCACAACTGTTCGCGTCCTGCTGGTTCATTATCTGACCTGGATTGCATTGCAGCTACGAGAAGCCCGTGGAAGGCCGGAAGATCAACTGGATGAAGGCCGGGATCCTCGAGGCCGACAGGGTCCTCACCGTCAGCCCCTACTACGCCGAGGAGCTCATCTCCGGCATCGCCAGGGGCTGCGAGCTCGACAACATCATGCGCCTCACCGGCATCACCGGCATCGTCAACGGCATGGACGTCAGCGAGTGGGACCCCAGCAGGGACAAGTACATCGCCGTGAAGTACGACGTGTCGACGGTGAGCTGGCTAGCTAGCTGATTCTGCTGCCTGGTCCTCCTGCTCATGCTGGTTCGGTTCTGACGCGGCAAGTGTACGTACGTGCGTGCGACGGTGGTGTGGTGTCCGGTTCAGGCCGTGGAGGCCAAGGCGCTGAACAAGGAGGCGCTGCAGGCGGAGGTCGGGCTCCCGGTGGACCGGAACATCCCGCTGGTGGCGTTCATCGGCAGGCTGGAAGAGCAGAAGGGACCCGACGTCATGGCGGCCGCCATCCCGCAGCTCATGGAGATGGTGGAGGACGTGCAGATCGTTCTGCTGGTACGTGTGCGCCGCCCGCCACCCGGCTACTACATGCGTGTATCGTTCTACTGGAACATACGTGTGAGCAACGCGATGGATAATGCTGCAGGGCACGGGCAAGAAGAAGTTCGAGCGCATGCTCATGAGCGCCGAGGAGAAGTTCCCAGGCAAGGTGCGCGCCGTGGTCAAGTTCAACGCGGCGCTGGCGCACCACATCATGGCCGGCGCCGACGTGCTCGCCGTCACCAGCCGCTTCGAGCCCTGCGGCCTCATCCAGCTGCAGGGGATGCGATACGGAACGGTACGAGAGAGAAAAAAAAACATCCTGAATCCTGACGAGAGGGACAGAGACAGATTGATTATGAATGCTTCATCGATTTGAATTGATTGATCGATGTCTCCCGCTGCGACTCTTGCAGCCCTGCGCCTGCGCGTCCACCGGTGGACTCGTCGACACCATCATCGAAGGCAAGACCGGGTTCCACATGGGCCGCCTCAGCGTCGACGTAAGCCTACCTCTGCCATGTTCTTTCTTCTTTCTTTCTGTATGTATGTATGTATGTACGAATCAGCACCGCCATTCTTGTTTCGTCGTCCTCTCTTCCCAGTGCAACGTCGTGGAGCCGGCGGACGTCAAGAAGGTGGCCACCACCTTGCAGCGCGCCATCAAGGTGGTCGGCACGCCGGCGTACGAGGAGATGGTGAGGAACTGCATGATCCAGGATCTCTCCTGGAAGGTACGTACGCCCGCCCCGCCAGAGCAGAGCGCCAAGATCGATCGACCGACCGACCACACGTACGCGCCTCGCTCCTGTCGCTGACCGTGGTTTAATTTGCGAAATGCGCAGGGCCCTGCCAAGAACTGGGAGAACGTGCTGCTCAGCCTCGGGGTCGCCGGCGGCGAGCCAGGGGTCGAAGGCGAGGAGATCGCGCCGCTCGCCAAGGAGAACGTGGCCGCGCCCTGAAGAGTTCGGCCTGCAGGGCCCCTGATCTCGCGCGTGGTGCAAAGATGTTGGGACATCTTCTTATATATGCTGTTTCGTTTATGTGATATGGACAAGTATGTGTAGATGCTTGCTTGTGCTAGTGTAATATAGTGTAGTGGTGGCCAGTGGCACAACCTAATAAGCGCATGAACTAATTGCTTGCGTGTGTAGTTAAGTACCGATCGGTAATTTTATAT |

**Table S1 Continued**

| **Accession** | **AC(%)** | **DNA Sequence** |
| --- | --- | --- |
| SHL403 | 84.27 | CCACAACTGTTCGCGTCCTGCTGGTTCATTATCTGACCTTGATTGCATTGCAGCTACGAGAAGCCCGTGGAAGGCCGGAAGATCAACTGGATGAAGGCCGGGATCCTCGAGGCCGACAGGGTCCTCACCGTCAGCCCCTACTACGCCGAGGAGCTCATCTCCGGCATCGCCAGGGGCTGCGAGCTCGACAACATCATGCGCCTCACCGGCATCACCGGCATCGTCAACGGCATGGACGTCAGCGAGTGGGACCCCAGCAGGGACAAGTACATCGCCGTGAAGTACGACGTGTCGACGGTGAGCTGGCTAGCTAGCTGATTCTGCTGCCTGGTCCTCCTGCTCATGCTGGTTCGGTTCTGACGCGGCGAGTGTACGTACGTGCGTGCGACGGTGGTGTGGTGTCCGGTTCAGGCCGTGGAGGCCAAGGCGCTGAACAAGGAGGCGCTGCAGGCGGAGGTCGGGCTCCCGGTGGACCGGAACATCCCGCTGGTGGCGTTCATCGGCAGGCTGGAAGAGCAGAAGGGCCCCGACGTCATGGCGGCCGCCATCCCGCAGCTCATGGAGATGGTGGAGGACGTGCAGATCGTTCTGCTGGTACGTGTGCGCCGCCCGCCACCCGGCTACTACATGCGTGTATCGTTCGTTCTACTGGAACATGCGTGTGAGCAACGCGATGGATAATGCTGCAGGGCACGGGCAAGAAGAAGTTCGAGCGCATGCTCATGAGCGCCGAGGAGAAGTTCCCAGGCAAGGTGCGCGCCGTGGTCAAGTTCAACGCGGCGCTGGCGCACCACATCATGGCCGGCGCCGACGTGCTCGCCGTCACCAGCCGCTTCGAGCCCTGCGGCCTCATCCAGCTGCAGGGGATGCGATACGGAACGGTACGAGAGAGAAAAAAAAACATCCTGAATCCTGACGAGAGGGACAGAGACAGATTGATTATGAATGCTTCATCGATTTGAATTGATTGATCGATGTCTCCCGCTGCGACTCTTGCAGCCCTGCGCCTGCGCGTCCACCGGTGGACTCGTCGACACCATCATCGAAGGCAAGACCGGGTTCCACATGGGCCGCCTCAGCGTCGACGTAAGCCTACCTCTGCCATGTTCTTTCTTCTTTCTTTCTGTATGTATGTATGTATGTACGAATCAGCACCGCCATTCTTGTTTCGTCGTCCTCTCTTCCCAGTGCAACGTCGTGGAGCCGGCGGACGTCAAGAAGGTGGCCACCACCTTGCAGCGCGCCATCAAGGTGGTCGGCACGCCGGCGTACGAGGAGATGGTGAGGAACTGCATGATCCAGGATCTCTCCTGGAAGGTACGTACGCCCGCCCCGCCAGAGCAGAGCGCCAAGATCGATCGACCGACCGACCACACGTACGCGCCTCGCTCCTGTCGCTGACCGTGGTTTAATTTGCGAAATGCGCAGGGCCCTGCCAAGAACTGGGAGAACGTGCTGCTCAGCCTCGGGGTCGCCGGCGGCGAGCCAGGGGTCGAAGGCGAGGAGATCGCGCCGCTCGCCAAGGAGAACGTGGCCGCGCCCTGAAGAGTTCGGCCTGCAGGGCCCCTGATCTCGCGCGTGGTGCAAAGATGTTGGGACATCTTCTTATATATGCTGTTTCGTTTATGTGATATGGACAAGTATGTGTAGATGCTTGCTTGTGCTAGTGTAATATAGTGTAGTGGTGGCCAGTGGCACAACCTAATAAGCGCATGAACTAATTGCTTGCGTGTGTAGTTAAGTACCGATCGGTAATTTTATAT |
| SHL408 | 82.60 | CCACAACTGTTCGCGTCCTGCTGGTTCATTATCTGACCTGGATTGCATTGCAGCTACGAGAAGCCCGTGGAAGGCCGGAAGATCAACTGGATGAAGGCCGGGATCCTCGAGGCCGACAGGGTCCTCACCGTCAGCCCCTACTACGCCGAGGAGCTCATCTCCGGCATCGCCAGGGGCTGCGAGCTCGACAACATCATGCGCCTCACCGGCATCACCGGCATCGTCAACGGCATGGACGTCAGCGAGTGGGACCCCAGCAGGGACAAGTACATCGCCGTGAAGTACGACGTGTCGACGGTGAGCTGGCTGGCTAGCTGATTCTGCTGCCTGGTCCTCCTGCTCATGCTGGTTCGGTTCTGACGCGGCGAGTGTACGTACGTGCGTGCGACGGTGGTGTGGTGTCCGGTTCAGGCCGTGGAGGCCAAGGCGCTGAACAAGGAGGCGCTGCAGGCGGAGGTCGGGCTCCCGGTGGACCGGAACATCCCGCTGGTGGCGTTCATCGGCAGGCTGGAAGAGCAGAAGGGCCCCGACGTCATGGCGGCCGCCATCCCGCAGCTCATGGAGATGGTGGAGGACGTGCAGATCGTTCTGCTGGTACGTGTGCGCCGCCCGCCACCCGGCTACTACATGCGTGTATCGTTCTACTGGAACATACGTGTGAGCAACGCGATGGATAATGCTGCAGGGCACGGGCAAGAAGAAGTTCGAGCGCATGCTCATGAGCGCCGAGGAGAAGTTCCCAGGCAAGGTGCGCGCCGTGGTCAAGTTCAACGCGGCGCTGGCGCACCACATCATGGCCGGCGCCGACGTGCTCGCCGTCACCAGCCGCTTCGAGCCCTGCGGCCTCATCCAGCTGCAGGGGATGCGATACGGAACGGTACGAGAGAGAAAAAAAAACATCCTGAATCCTGACGAGAGGGACAGAGACAGATTGATTATGAATGCTTCATCGATTTGAATTGATTGATCGATGTCTCCCGCTGCGACTCTTGCAGCCCTGCGCCTGCGCGTCCACCGGTGGACTCGTCGACACCATCATCGAAGGCAAGACCGGGTTCCACATGGGCCGCCTCAGCGTCGACGTAAGCCTACCTCTGCCATGTTCTTTCTTCTTTCTTTCTGTATGTATGTATGTATGTACGAATCAGCACCGCCATTCTTGTTTCGTCGTCCTCTCTTCCCAGTGCAACGTCGTGGAGCCGGCGGACGTCAAGAAGGTGGCCACCACCTTGCAGCGCGCCATCAAGGTGGTCGGCACGCCGGCGTACGAGGAGATGGTGAGGAACTGCATGATCCAGGATCTCTCCTGGAAGGTACGTACGCCCGCCCCGCCAGAGCAGAGCGCCAAGATCGATCGATCGACCGACCACACGTACGCGCCTCGCTCTTGTCGCTGACCGTGGTTTAATTTGCGAAATGCGCAGGGCCCTGCCAAGAACTGGGAGAACGTGCTGCTCAGCCTCGGGGTCGCCGGCGGCGAGCCAGGGGTTGAAGGCGAGGAGATCGCGCCGCTCGCCAAGGAGAACGTGGCCGCGCCCTGAAGAGTTCGGCCTGCAGGGCCCCTGATCTCGCGCGTGGTGCAAAGATGTTGGGACATCTTCTTATATATGCTGTTTCGTTTATGTGATATGGACAAGTATGTGTAGATGCTTGCTTGTGCTAGTGTAATGTAGTGTAGTGGTGGCCAGTGGCACAACCTAATAAGCGCATGAACTAATTGCTTGCGTGTGTAGTTAAGTACCGATCGGTAATTTTATAT |

AC: amylopectin content ; The amylopectin content was determined according to the National Standards of the People’s Republic of China, GB 7648-87 (China Standard Press 1987).
